# Oral Presentations

**DOI:** 10.1155/1995/27578

**Published:** 1995


### F001

BILIARY CANCER OF THE CONFLUENCE: COMBINED MODALITY THERAPY

V.Dufek, J.Petrtýl, P.Klener, R.Brůha

First Medical Department, Charles University, Prague, Czech Republic

Bifurcation cancer (BC) is one of the most dismal GIT malignancies because of the difficulties in early diagnosis and treatment. Both endoscopic stenting or palliative surgery improve the survival conditions; the median survival, however, does not exceed 16 months. This study was performed to assess the feasibility of combined modality therapy in BC. Since Jan. 1990 to Feb. 1994, 61 consecutive jaundiced pts with BC (38 females, 23 males, mean age 56,9 years) were entered into this procpective study. In 36 pts the endoscopic drainage (pastic 11F stents) alone was performed; in 25 subjects the drainage of the biliary tree was done by a transhepatic approach using 24F metalic self-expanding stents. The stenting was in 29 pts completed by regional chemotherapy or by intraluminal radiotherapy (iridium-192 wires). Endoscopic stenting (two stents) was successful in 78% out of cases, the transhepatic one reached 96%. Median survival in pts, treated by stenting alone, was (in both types of stents) 236 days. Complications did not exceed 2,3%. In pts, treated by bilioduodenal endoprostheses and regional chemotherapy, or intraluminal radiotherapy the survival median reached 594 days (interval 160 to 713 days). No weighty side effects either local or systemis we were able to demonstrate. On the basis of our results is possible to conclude that in BC the combination of transhepatic insertion of metalic stents with subsequent regional chemotherapy and intralumiminal radiotherapy is the methos of choice at this time.

### F002

**K-ras Mutations in Klatskin-Tumors and in Klatskin-Mimicking Lesions**

**JR Izbicki, KL Prenzel, A Niendorf, WT Knoefel, A Gebhardt, SB Hosch, B Passlick, D Evans, X Rogiers, CE Broelsch**
**Depts. of Surgery and Pathology, University of Hamburg, Hamburg, Germany**

**Introduction:** A significant percentage of suspected Klatskin lesions turn out to be fibrosing cholangitis only after resection. Biliary tree probing to analyze the bile could potentially show different degrees of expression of oncogenes and help in decision-making. We therefore analyzed K-ras mutations in Klatskin and Klatskin-mimicking lesions.

**Material:** Pathologic specimens from 31 consecutive patients that had undergone resection of the extrahepatic biliary tree for suspicion of a malignant tumor were included. DNA was extracted from a total number of 39 paraffin blocks. In order to detect mutations in the K-ras gene, a modification of the PCR-based method of Kahn et al (Oncogene 6:1079-83; 1991) was employed.

**Results:** 27 of the lesion were Klatskin tumors, 4 lesions were only mimicking a malignant lesion and turned out to be fibrosing cholangitis in the final pathological report. The K-ras gene could be amplified from 37 of 39 blocks. A mutated K-ras codon 12 was detected only in one Klatskin tumor.

**Conclusions:** Mutations of K-ras codon 12 is extremely rare in Klatskin tumors. It is therefore not different from expression in mimicking lesions. Analysis of bile will thus not help to differentiate these two entities before surgery. Complete resection of the lesion in question will remain the only therapeutic option.

### F003

EXTRAHEPATIC CHOLANGIOCARCINOMA: HOMOGENEOUS DIAGNOSTIC AND THERAPEUTIC MANAGEMENT FOR 100 CASES.

S. Alfieri, G.B. Doglietto, G. Costamagna, F. Pacelli, D. Frontera, C. Carriero, G. De Vivo, P.F. Crucitti, F. Crucitti.
Department of Surgery - Catholic University - Rome - Italy

AIM of this study is to report short and long term results of surgical and endoscopic treatment of 100 patients with extrahepatic cholangiocarcinoma (CC) consecutively observed in our unit between November 1987 and May 1994. MATERIALS AND METHODS: there were 58 men and 42 women, with a mean age of 68 years. To evaluate origin, level and state of advancement of tumor spread, all patients routinely underwent US, CT-scan and ERCP. Associated major prexisting disease, advanced age and poor general conditions were never considered as contraindication of endoscopic procedure. After diagnostic procedures, according to Longmire classification, tumors were found to be in the poximal bile ducts in 66 patients, midportion in 13 and distal portion of the bile duct in 21 patients. With the exception of 4 patients, a preoperative drainrge by endoscopic placement of one or more endoprostheses has been always performed. Advanced aged (>75 years), poor general conditions, regional metastases, bilateral involvement of the hepatic ducts beyond the secondary branches, involvement of the main trunk of the hepatic artery, bilateral involvement of the portal vein branches or a combination of vascular involvement to one side of liver with the extensive cholangiographic involvement on the other, were considered contraindications to resection. On the basis of the above criteria, 27 patients out of 100 were considered suitable for resection of the tumor. At laparotomy, resectability was confirmed in 22 patients: 6 patients with a hilar CC, 3 and 13 patients with a middle and distal tumor respectively. Local excision of hilar tumor was performed in 4 cases, left hepatectomy was required in 2 (Type IIIb). Pancreatoduodenectomy was performed in 14 cases with a distal and midportion bile duct tumor. Local excision of choledocus were performed in 2 other patients. In all cases all loco-regional lymphatics and areolar tissue was excised. RESULTS: there were 6 post-operative morbidity (27%) with 2 postoperative death (9%). Macroscopic and microscopic tumor clearance was obtained in 16 patients. In the remaining 6 cases final hystology report revealed microscopic invasion of lymphonodes or perineural tissue. The overall mean survival was 22 months (range 3-73); 6 patients are still alive, disease free. The mean survival was 21, 7 and 25 months for patients with upper, middle and distal CC respectively. For the other 78 patients endoscopic drainage was the only treatment performed but in 11 cases PTBD was associated to complete endoscopic intrahepatic drainage. The morbidity rate and the direct procedure related mortality was of 13% and 1.3% respectively with a mean survival of 13.2 months (range1-24). CONCLUSION: from the present study is justifiable to conclude that long term survival and potential cure can be achieved, in selected cases, by radical surgical resection. Endoscopic insertion of single or multiple endoprostheses for patients unsuitable to surgery is a safe procedure and provides good palliation.

### F004

**COMBINATION OF INTRAOPERATIVE AND EXTERNAL BEAM RADIATION THERAPY WITH RESECTION OF PANCREATIC CANCER.**

G.B. DOGLIETTO, D. FRONTERA, M. BOSSOLA, G. VIOLA, G. ALFONSI, S. CASALE, F. CRUCITTI.
**DEPARTMENT OF SURGERY, CATHOLIC UNIVERSITY SCHOOL OF MEDICINE, ROME, ITALY.**

To improve local control, from March 1990 a combined multimodal treatment including surgical resection, intraoperative irradiation (IORT) and external beam radiation therapy (ERT) has been used in 17 patients with resectable carcinoma of the head of the pancreas. In all cases but 2 a subtotal pancreatectomy was performed. IORT has always been delivered to the tumor bed including celiac axis, portal vein, origin of superior mesenteric vessels with a dose of 10 Gy and an energy of 6 MeV. Postoperative ERT alone of the tumor bed (3 box technique; dose: 50 Gy) has been used in 9 patients (Group A); in another 8 cases a preoperative treatment (irradiation of the liver and the pancreas the day before surgery; dose: 5 Gy) has been added (Group B). In 5 cases postoperative ERT was not completed because of bad clinical condition of the patients. The mean time spent to deliver IORT (the patient is transported from the 9th to the 2nd floor) was 52 minutes. Postoperative major complications were observed in 4 patients (23.5 %) and mortality was 5.8 % (1 case).

In Group A one patient is alive, disease free, 4 months after operation; 7 patients died after a mean time of 10.6 months (min.: 5; max.: 19): all of them but one have had radiological evidence of liver metastasis after a mean time of 5.7 months (min.: 2; max.: 7); one patient developed peritoneal spread of tumor 14 months after surgery.

In Group B 3 patients are alive, disease free, respectively 3, 21 and 24 months after operation; 5 patients died after a mean time of 22 months (min.: 9; max.: 37): in 3 of them there was radiological evidence of liver metastasis after a mean time of 7.3 months (min.: 5; max.: 11); 2 patients developed local recurrence 16 and 29 months after surgery respectively.

Our experience suggests that combination of ERT and IORT in surgical resection of pancreatic cancer is safe, tolerable and offers a good local control. High frequency of liver metastases suggests that more aggressive treatment including preoperative ERT (as in Group B) and/or chemotherapy needs to be evaluated.

### F005

LATE RESULTS OF PARTIAL PANCREATODUODENECTOMY

A.G.Mylnikov, I.M.Buriev
A.V.Vishnevsky Institute of Surgery, Moscow, Russia

During the last 18 years 182 partial pancreatoduodenectomies (PPDE) for tumors of the pancreatoduodenal area (156) and chronic pancreatitis (26) were carried out. The follow-up study from 4 months to 12 years in 118 patients is considered. The average survival rate was 22,8 months for tumors of the pancreatic head with significant differences depended on morphological changes: for ductal adenocarcinoma - 18,6 months; carcinoid tumors - 50,5 months, rare tumors - 20 months. Survival in patients with periampullary carcinomas was 34,4, months for papillary tumors, 37 months for distal bile duct tumors and 37,6 month for duodenal ones. The five-year survival rate was 8,8% for ductal adenocarcinoma (10 patients - 29,9% - still alive; 33,3% for pancreatic head carcinoid (83,3% still alive); 16,7% for rare pancreatic head tumors (33,3% alive); 22,5% for papillary tumors, 42,9% for distal bile duct tumors and 14,3% for duodenal tumors (32,5%, 42,9%, 42,9% of patients still alive accordingly). 23 patients from neoplastic group requared secondary operations due to complicated tumor reccurence (6) or late complications or consequences of PPDE (17). The average survival rate for patients operated on for chronic cephalic pancreatitis was 42,9 months ranged from 10 months to 11,5 years, 82,4% of patients alive. The quality of life was good in 82,4%, satisfactory only in 17,6%, bad results were not observrd. Secondary operations were performed in 11,8% from this group of patients. Thus the long-term survival after PPDE is better for patients with periampullary tumors and surprisingly, for pancreatic head carcinoid than for men suffering from ductal adenocarcinoma of the pancreatic head; we think also that PPDE is the good procedure for treatment of severe chronic cephalic pancreatitis.

### F006

PROGNOSTIC RELEVANCE OF PLOIDY AND S-PHASE IN RESECTED CARCINOMA OF THE PANCREAS

H. Nekarda, J.D. Roder, K. Becker*, A. Rossmann, J. R. Siewert
Department of Surgery and *Institut for Pathology, Technische Universität München, Klinikum rechts der Isar

Objective: The prognosis of patients with ductal carcinoma of the pancreas compares unfavorably to other GI tumors. Analysis of prognostic factors is therefore essential to identify patients who might benefit from adjuvant therapy.
Materials and methods: The data of 69 patients who had a resection of a ductal carcinoma of the pancreas between 1982 and 1990 were documented prospectively. A R0 resection was achieved in 32/69 (46.4%) patients. Multiple stepwise regression analysis (Cox model) of the pT-, pN-, pM-categories, grading, size, perineural invasion, ploidy, and S-phase of the tumor was performed selectively for the total patient population and in these 32 patients respectively. Ploidy and S-phase of the tumor were assessed using flow cytometry and the 'Multicycle' software package. Median survival time for all patients was 13 months with a 5-year survival rate of 10.1%.
Results: The aneuploidy rate was 68.1% for the total patient population and 56.7% for the subgroup of R0-resected patients. By multivariate analysis an independent prognostic impact was proven for S-phase and tumor grading. Survival was significantly longer in the 24 patients who had a S-phase below 8.0% as compared to those 6 patients with a S-phase above 8.0% (median survival 17.5 months versus 6.5 months). Median survival in the 18 patients with well or moderately differentiated tumors was 23 months as compared to 7.5 months in the 12 patients with poorly differentiated tumors ($p<0.05$).
Conclusion: Tumor differentiation and S-phase independently influenced the prognosis of patients with carcinoma of the pancreas. These parameters may be helpful to identify those patients who benefit from adjuvant therapy.

### F007

**SURGICAL EXCISION OF HEPATOCELLULAR CARCINOMA (HCC) IN CIRRHOTIC PATIENTS. RESULTS OF A PROSPECTIVE STUDY**

C.Gouillat, D.Manganas, G.Saguier, R.Duque Campos, Ph.Bérard

Département de Chirurgie, Hôtel Dieu Lyon France

We initiated a prospective study to assess the results of surgical resection of HCC in European cirrhotic patients selected using criteria inspired from the Japonese ones.
From 1986 to 1991, 37 patients ranging in age from 47 to 84 years were included in the study. Evidence of associated viral chronic hepatitis was demonstrated in 18 patients. The mean values of bilirubin level and Indocyanin Green Retention Rate were 20±12 Umol/l (range : 6-53) and 29±15% (12-69) respectively.
The mean tumor diameter was 5.3±2.6 cm (range : 2-11). Resection was performed with the aim to spare the non tumoral parenchyma. Depending upon the tumor diameter and location an anatomic hepatectomy was performed in 12 patients and an ultrasonically-guided tumorectomy in 25. Seventeen patients received an average of 3±1 (2-5) blood units during surgery. Four patients (11%) died postoperatively including 2 from hepatic failure. The overall survival rates at 1, 2, 3, 4 and 5 years were 65%, 49%, 37%, 26% and 18% respectively. The cumulative recurrence rate was 32% at one year, 60% at 2 years, 70% at 3 years and 93% at 4 years. Univariate analysis failed to demonstrate any prognostic factor.
It is concluded that HCC can be safely resected in selected french cirrhotic patients. Resection results in a 18% 5 years survival rate and the tumor recurrence rate is close to 100% after 4 years.

### F008

**PRIMARY SARCOMA OF THE LIVER - REPORT ABOUT 10 SURGICALLY TREATED PATIENTS**

C. Zornig, M. Peiper, X. Rogiers, ChE Broelsch.
Dept. of General Surgery, University Hospital Hamburg-Eppendorf, Germany

Primary sarcoma of the liver is a very rare neoplasm (0,1% of all hepatic tumors). A rather large series of surgically treated patients is presented.

From 1985 until 1994 10 patients with primary sarcomas of the liver have been operated on in our department. Data about the clinical presentation, treatment and histological classification were registered. The follow-up is complete.

There were 7 women and 3 men with an average age of 41 (19-76) years. 5 hemihepatectomies, 4 segmentectomies and 1 transplantation was performed. Wide margins (R0) could be achieved in 7 patients even in case of multifocal growth or extended resections for infiltration of other organs. Six different histological types were found. Two tumors were graded G1, 4 G2 and 4 G3. There were no synchronous lymph node or distant metastases.

After a median follow-up of 40 (4-95) months 7 patients are tumor free (6 of the R0 resections). The patient with multifocal growth and transplantation develloped lung metastases and a recurrence in the graft after 14 months and lives with tumor. Two patients with initial marginal resection (R1) had local recurrences, further operations and chemotherapy but died because of the tumor disease. The third patient with R1 resection has no evidence of disease 4 months after the operation.

Even in case of undifferentiated tumors and/or infiltration of surrounding organs in all but one cases long-term survival could be achieved if the sarcoma initially was resected with wide margins (R0).

### F009

**CURATIVE RESECTION OF LIVER METASTASES AND POSTOPERATIVE ADJUVANT REGIONAL CHEMOTHERAPY-RESULTS OF A PROSPECTIVE RANDOMIZED TRIAL.**

B. Leibl, M. Ulrich, M. Butters, R. Bittner.
Marienhospital Stuttgart, Chirurgische Klinik, Germany

From 4/90 to 11/94 27 patients were enrolled into a prospective randomized trial after curative resection of liver metastases from a former colorectal carcinoma. The aim of the study is to judge the efficiancy of a postoperative adjuvant regional chemotherapy (group A, 3 courses i.a. 5FU/folinic acid).

**RESULTS**

| | A | B |
|---|---|---|
| n=number of patients | 14 | 13 |
| age (median, years) | 68 | 67 |
| | (36-78) | (52-86) |
| procedures (prim. res.) | | |
| segmentectomy | 11 | 9 |
| hemihepatectomy left | 1 | 1 |
| hemihepatectomy right | 2 | 3 |
| number of metastases res. | | |
| sing./2-4 | 6/8 | 7/6 |
| postop. mortality | 0 | 0 |
| curative re-res. (liver) | 3 | 1 |
| follow-up | 24 | 18.5 |
| (median, mo.) | (3-56) | (2-41) |

There were 7 cases in group A and 8 cases in group B with intra-/and or extrahepatic recurrence. 2 patients in each group died because of tumor progression.

Hepatic resection is a safe method for treatment of hepatic secondaries; until now we could not see any difference between the two groups.

### F010

**INTRAPERITONEAL GALLSTONE**
**( Experimental Study )**

**H. Sonmez, O. Demircan, O. Yagmur, F. Doran***
**Cukurova University School of Medicine, Department of General Surgery and Pathology*, Adana-TURKEY**

**The newest treatment modality for the management of gallstone disease is laparoscopic cholecystectomy. In our hospital laparoscopic cholecystectomy has been performing since September 1992. During the procedure if gallbladder is perforated, gallstones may fall into the peritoneal cavity. Sometimes we can't be able to remove these stones from the abdomen. Our study's aim was to evaluate effects of free gallstones in the peritoneal cavity. The rats were used in the study which were divided into 2 equal groups according to the gallstones that were used. Group-1 (n=20): Gallstones were washed with 0.9% NaCl three times. Group-2 (n=20): We used the gallstone as we took it out from the gallbladder. These stones were taken out from the patients who were undergone cholecystectomy for chronic gallstone cholecystitis. These gallstones were given into the peritoneal cavity of the rats in steril condition. After 6 weeks, rats were sacrificed. Gallstones and adhesions were determined and graded. Subsequently these adhesions were examinated histologically. There were no difference of adhesion scores between the groups. Histologic examination of the adhesions were showed diffuse inflammatory cells. In conclusion, intraperitoneal free gallstones are not innocent.**

### F011

**CHOLESTEROL GALL STONE DISSOLUTION USING MTBE: STUDY OF EFFICACY, SAFETY AND PREVENTION OF RECURRENCE**

**BY**
**MYT.RASHED(MD), R.ZAHER(MD), AA.SOLIMAN(MD), ELSAID HASSAN(MD), S.ELSHEIKH***
**Alexandria University, Hepatobiliary & Radiology * Unit Alex. Egypt .**

23 patients 4 males and 19 females(mean age 38.6 years), with symptomatic noncomplicated cholesterol gall stone(s) were treated by percutaneous transhepatic direct contact MTBE dissolution. All patients were evaluated clinically and ultrasonographically before and after dissolution. Also CT scan evaluation was done before dissolution to detect any calcification andto assure good gallblader bed. Patients were classified into two groups:group 1(12 patients including two patients with residual stones), received ursodeoxycholic acid for threemonths after dissolution and group 2 (eleven patients) did not receive ursodeoxycholic acid therapy. In 21 patients first time gallbladder puncture was successful (91%), and in two obese patients second punctures (5 and 2 months later were successful using stiffer catheter. Average dissolution time was 8.47 hours. Gall stone dissolution rate ranged from 0.98 to 3.4 cm/hour. Yhe rate of dissolution was increased with the increase in stone number and stone burden. Complete stone dissolution was achieved in 21 patients (91.3%0), residual gall bladder sediment not casting posterior shadow on ultrasound examination was detected in 3 patients (13.04%). Residual stone fragments escaped dissolution in two patients(8.69%0). Side effects of the procedure were minimal and no major complications occurred. Results. of three years follow up showed only one genuine recurrence (10%) out of 10 patients who received prophylactic post dissolution therapy in group 1, while 5 out of the 11 patients in group 11 showed recurrence (45.45%). Direct contact MTBE cholesterol gall stone dissolution issafe and effective alternative forsurgery. Post dissolution Ursodeoxycholic acid therapy for 3 months is highly effective in preventing recurrence of stones .

### F012

**Can gallbladder pathology predispose to colorectal carcinoma?**

P. Karydakis, S. Pierrakakis, N. Economou, G. Douridas, J. Marinakis, G. Antsaklis.
Department of Surgery, Sismanoglion General Hospital, Athens - Greece.

In the last 15-year period it was suggested the possibility of a correlation between choelecystectomy and colorectal carcinoma, never the less the several studies have been varied and in some cases contradictory.
The object of our study is to give a statistic contribution to this controversial problem including in our group of patients not only the already cholecystectomized, but also the ones with cholelithiasis to look for an eventual correlation between gallblader pathology and colorectal cancer. For this ponrpose we have analysed 459 patients (253 men and 206 women) 129 of which were suffering from gallbladder pathology that underwent an operation for colorectal cancer from 1981 to 1994.
We found a higher incidence of right colonic cancer especiallay in the women among the patients suffering from gallbladder pathology.

### F013

CHOLELITHIASIS : A NUTRITIONAL AND EPIDEMIOLOGIC STUDY.

E.D. Papavassiliou, M. Radou-Sfiri, D.A. Zachariadis, L.S. Kalli,
S. Boutzouvis, N.A. Papanagiotou.
NIMTS Hospital, Athens, Greece.

Cholelithiasis has many risk factors. Among others high calorie intake, fatty foods and refined sugars have been considered predisposing factors, while dietary fibers and possibly small amounts of alcohol may be protective. In this study we evaluated the dietary intake over a large number of foods in patients with documented cholelithiasis, to access their potential role in the pathogenesis of this disease.

METHODS : The diagnosis was established by ultrasound. The dietary habits of the patients were assessed by a detailed nutritional questionnaire. 73 patients were studied (age 66.4 years, range 11.3; body weight 73.3 kg, range 11.6; 23 men and 50 women). Of them 41% had chronic constipation, 30.1% arterial blood hypertension, 26.0% peptic ulcer disease, 24.6% diabetes mellitus, 15% hypercholesterolemia and 15% diverticular colon disease. 43.8% used cardiovascular medicines and 19.1% mild antidepressants. 19.1% were smokers.

RESULTS :

| Food consump. | Daily (%) | Weekly (%) | Occasionally (%) | Food consump. | Daily (%) | Weekly (%) | Occasionally (%) |
|---|---|---|---|---|---|---|---|
| Starchy foods | 10.9 | 63.0 | 26.0 | Animal fat | 6.8 | 53.4 | 39.7 |
| Milk | 47.9 | 27.3 | 24.6 | Olive oil | 84.9 | 13.6 | 1.3 |
| Milk prod. | 58.9 | 36.9 | 4.1 | Eggs | 6.8 | 43.8 | 49.3 |
| Vegetables | 30.1 | 64.3 | 5.4 | Sugar | 28.7 | 35.6 | 35.6 |
| Fruits | 68.4 | 31.5 | 0 | Coffee | 64.3 | 15.0 | 20.5 |
| Legumes | 0 | 59.9 | 41 | Wine | 16.4 | 13.6 | 69.8 |
| Beef | 12.3 | 75.3 | 12.3 | Beer | 2.7 | 24.6 | 72.6 |
| Chicken or fish | 4.1 | 87.6 | 9.5 | Whisky | 1.3 | 5.4 | 93.1 |

CONCLUSIONS : Beef, chicken or fish, fruits, vegetables, olive oil, starchy foods and milk products were the preferable foods, while legumes, alcohol and eggs, were avoided. Advanced-age diseases were present in high incidence.

### F014

CLINICO-PATHOLOGICAL OUTCOME OF INTRAPERITONEALY RETAINED GALL STONES WITH DIFFERENT CHARACTERISTICS.

I. Alacayir, M.A. Yerdel, U. Kusbasioglu, U. Gur, A.G. Turkcapar, N. Aras
Dept. of Surgery, Ankara University. Med. School

Rupture of the gallbladder with spillage of its contents is not an infrequent occurrence during laparoscopic cholecystectomy (LC) and recovery of all lost stones is impossible. Nevertheless, the natural course of these "left behind" stones is not known. This study was undertaken to assess the probable effects of intraperitonealy retained gall-stones in a mouse model. Group I served as the control group (simple laparotomy, n:10). Groups II, III and IV (n:10 in each group) were study groups. "Intact-sterile-cholesterol" (Group II), "crushed-sterile-cholesterol" (Group III), and "crushed-infected-pigment" (Group IV) gall-stones aseptically retrieved from 3 different patients were implanted to the peritoneal cavity of the animals. Animals were sacrificed 6 and 12 weeks after the operations. Cultures and tissue samples were obtained. No animal was lost and no micro/macroscopic abnormality was observed in Groups I and II and cultures remained negative. In Group III, adhesions surrounding the fragmented stone particles were evident at 12th week and no mortality was encountered. Histopathology revealed fibroblastic reaction and cultures remained negative in Group III. In Group IV, 4 animals died because of intraabdominal sepsis before their sacrifice. Remaining mice showed severe adhesions with localized abscesses and plastron was evident in all surviving mice at the 12th week. Severe inflammatory reaction and localized peritonitis with positive culture and dense fibrinoid reaction was evident in Group IV. Intraperitonealy retained gall-stones remain inert and do not cause even microscopical peritoneal reaction unless they are crushed to fragments or infected. It is for this group of patients that laparotomy for total stone clearance is probably not justifiable. Better stone retrieval techniques or even laparotomy may be worthwhile to consider in patients with crushed and particularly infected stones.

### F015

EFFECT OF OBSTRUCTIVE JAUNDICE ON BACTERIAL TRANSLOCATION OF THE GIS TRACT IN RATS.

Ö. Alabaz, H. Demiryürek, A. Yaman,
F. Doran, O. Demircan, E.U. Erkoçak.
Department of Surgery, University of Çukurova, Adana, TÜRKİYE.

Bacterial translocation which can result in systemic infections and septicemia are observed after shock, burns, intestinal obstruction, trauma, radiotheraphy, endotoxemia and extrahepatic cholestasis.
An experimental study was designed to investigate the changes at the intestinal compartment, during obstructive jaundice in rats. Sixty Wistar-Albino rats were divided into 3 equal groups. I.Control group, II.Sham ligation group, III.Choledoch ligation group. All rats were killed in fourteenth day and cultures were taken from mesenteric lymph nodes and colonic lumen. Biopsies were taken from terminal ileum for histopathological examination and the results were compared with the control group.
Bacterial translocation at the mesenteric node was found 60%(12/20) in choledoch ligation group, 30%(6/20) in sham ligation group and no found(0/20) in control group. In choledoch ligation group was seen the mucosal disruption with intraluminal over-colonization of the bacteria.
In conclusion, the reason for bacterial translocation was thought to be the disruption of mucosal integrity in obstructive jaundice.

### F016

***EFFECT OF MANNITOL ON BACTERIAL TRANSLOCATION IN OBSTRUCTIVE JAUNDICED RATS***

***Yıldıray YÜZER, Tayanç ÖNCEL*, Deniz FER**, Işıl ÇOKER**, Sinan ERSİN*, Ahmet ÇOKER, Ali MENTEŞ***

***The HPB Surgery Unit, Department of Surgery*, Aegean University Faculty of Medicine and Department of Biochemistry**, SSK Tepecik Hospital, Izmir, TURKEY***

An experimental study was designed to investigate intestinal permeability, bacterial population changes and the metabolic consequences due to experimental obstructive jaundice in rats. Twenty male Wistar rats were devided into three groups; Group 1: Control (n=6), Group 2: experimental jaundice group (n=7), Group 3: rats treated with 2 ml 20% mannito intraperitonealy after the establishment of experimental jaundice (n=7).

Intestinal permeability was determined by Tc99 disappearance curves that was injected into the ileum. Aspiration materails taken from the distal ileum were cultered for aerobic and anaerobic bacteria and bacterial counts were assessed. Urine urea nitrogen content, and serum ammonia levels were determined besides tests reflecting the livers functional status. A hystopathologic investigation was carried out on samples taken from the ileum of rats. The jaundiced rats showed increased blood ammonia ($p<0.05$), increased urinary urea nitrogen excretion ($p<0.05$), increased population of both the gram negative and the gram positive bacteria ($p<0.01$), and submocosal oedema was more common ($p<0.05$) when compared to the controls. After the injection of mannitol, there was a statistically significant decrease observed in urinary urea nitrogen excretion, bacterial population levels and submucosal oedema amount. The serum bilirubin and serum ammonia levels had decreased but the amount of change was not statistically significant when compared to the jaundiced group of rats.

Our findings suggest that intestinal compartment plays an important role on the complex changes that arises during the course of obstructive jaundice. More efficient therapeutic efforts targeting the intestinal compartment may be of benefit during the preoperative preparation period of patients with obstructive jaundice.

### F017

PROSPECTIVE STUDY OF SEPTIC RESPONSE FOLLOWING BILIARY MANIPULATION: INCIDENCE AND CYTOKINE CORRELATION

C.S. Ho, O. Rotstein, E. Yeung
Department of Radiology, The Toronto Hospital & The University of Toronto, Toronto, Canada

To prospectively study the incidence of sepsis following percutaneous biliary drainage (PBD) on patients with obstructive jaundice and to correlate this with alteration of blood cytokine levels. Forty patients with obstructive jaundice had PBD and were monitored clinically for their response to sepsis. The clinical response was categorized according to clearly-defined criteria. Tumor Necrosis Factor (TNF) are determined by measuring lysis of the TNF-sensitive cell line. Both the clinical responses and the blood cytokine level were measured at predetermined time intervals in the first 24 hours after PBD. Thirty-three of 40 patients had serial blood sampling for cytokine; the other 7 patients who refused serial blood sampling were included to reflect the actual incidence of sepsis. One of the 40 patients developed a severe septic response with 15 fold elevation of cytokine 90 minutes after PBD. She had pre-existing cholangitis due to blockage of an indwelling endoprosthesis inserted endoscopically. Pus was found in bile. The incidence of septic response following PBD in patients with obstructive jaundice is low: The severe septic response in one patient was correlated with significant rise of TNF. The study was supported by a grant from Sanofi-Winthrop.

### F018

FUNCTION OF INTESTINAL LOOP INTERPOSED IN BILIARY SURGERY.

G.Salgarello, M.Cagossi
Department of Surgery, S.H.Catholic University, Rome, Italy.

The postoperative function of intestinal loop interposed for the replacement of the hepato-choledodal tract was evaluated in four patients using manometry even in the course of physiological and pharmacological stimulation; at the same time the gastrinemic profile was detected.

Authors gave evidence that:

1- basically a poor motor activity was present in the interposed loop;

2- sudden wave bursts, 15-20 mmHg in amplitude and 10|min. in frequence, happeared without propulsive effects non related with respiratory moovements;

3- following mechanical stimulus a periodic wave series, 10|min. in frequence, lasting almost 60 seconds, happeared;

4- a meat soup intake caused an exponential increase of gastrin during the first 60 minutes followed by a reversal toward baseline in the subsequent hour; while a progressive increase in the motor activity of the loop was elicited just 25 minutes after the oral intake with a real increase of the interposed loop tonus;

5- finally the administration of neostigmine gave rise to bursts connected with the increased motor activity after 10 minutes.

These statements could be obtained using endoluminal drainages inserted during the operation.

The evidences reported suggested a good preservation of the functional capacity of the interposed loop even if it was allocated in a different situation caracterized by poor mechanical stimulus which caused a basic minor motor activity.

Thenelse the loop worked in an incoordinate neuro-humoral environment.

### F019

THE BILIARY TRACT DRAINAGE PROCEDURES FOR MALIGNANT OBSTRUCTION.

Michael E.Kontes, S.Pinis, P.Gousis, M.Rallis, G.Androulakis.
4th Surgical Department, University of Athens, Greece.

This study deals with the Common bile duct drainage procedures due to biliary tract malignant obstruction.
We studied 65 patients, 31 males, 34 females whose ages ranged from 43 to 90 years old (Mean age was 67 years).
These patients were treated in the last 5 years (July 1989 - November 1994) in our department.
The indications for operation were:
Tumour of the head of the pancreas in 54 patients.
Periampullary neoplasm in 4 patients.
Common bile duct tumour in 2 patients.
Gallbladder tumour in 3 patients.
Klatskin tumour in 2 patients.

We used a variety of drainage procedures among Common bile duct or Gallbladder or Common hepatic duct and duoedenum or jejunum or stomach on the other hand.
Twentynine of our patients had a gastrojejunostomy as well.
Postoperatively 9 patients died.
Three patients had bile leakage and underwent a reoperation . One patient had a malfunctioning gastrojejunostomy and had also a reoperation.
As a conclusion a drainage procedure is the solution of choice for malignant obstruction, especially for those patients whose obstructing tumour (more frequently pancreatic head) excision is infeasible.

### F020

**MALIGNANT BILIARY OBSTRUCTION: REVIEW OF 66 PATIENTS.**

**A.Calvo, F.Martín, R.García, I.Moreno, A.López, L.Lozano, J.Guadarrama, T.Butrón, M.Lomas, F.Botella.**

**Department of Surgery B (Prof. M.R.Vilariño).12 de Octubre General Hospital.Madrid.Spain.**

**PURPOSE:** To report, our experince with malignant biliary obstruction in a retrospective review.
**MATERIALS AND METHODS:** In a review of 66 patients, 53 underwent to paliative treatment including surgical(45) or endoscopic treatment(8), the other 13 were considered unfit for surgery because of poor general health, metastasic disease or advanced age.
**RESULTS:** Palliative bypass was performed in 25 patients, external drainage(Kehr) in 9 patients, transtumoral intubation whit plastic endoprosthesis in 4 and 7 patients had no procedure other than an exploratory laparotomy. Endoscopic treatment were by percutaneous placement of Wallstent endoprosthesis.
Thirty day mortality was 29.25% in surgical treatment and 1.5% in percutaneous biliary stenting. Minor postoperative complications were 16% in surgical treatment and 12.5% in billiary stenting. Postoperative staying in surgical treatment was 16.2 days, in percutaneous placement of endoprosthesis was 4 days. There was no difference in median survival.
**CONCLUSIONS:** Wallstent endoprosthesis offers a non-surgical method for releaving jaundice of malignant aetiology, especially in those patients who have a high operative mortality risk, with lower postoperative staying, lower mortality and lower postoperative complications.

### F021

GALLBLADDER CANCER. OUR EXPERIENCE.
A RETROSPECTIVE STUDY OF THE LAST 5 YEARS.

M.Diaz Tie, T.Butrón, M.J. Castillo, A. Garcia Carranza, O. Garcia Villar, J. Garcia Borda, M. Garcia Padrós, R. Ramos, M. Lomas.

I Department of General and Digestive Surgery

Doce de Octubre Hospital. Madrid. Spain.

Primary gallbladder cancer is the most common malignant lesions of the biliary tract and the results of its treatment are dismal. We report our experience in the last 5 years.
MATERIAL AND METHODS: From January of 1989 to June of 1994 we treated 796 patients with gallbladder pathology, 2% (16 cases) gallbladder cancers were found with a female to male ratio of 7:1. There were classified following Nevin clasification.
RESULTS: The mean age was 64 years. Abdominal pain was the symptom more frecuent (87.5%). Cholelitiasis was present in 10 cases (68.7%). Accurate preoperative diagnosis was established in 5 cases (31.2%), preoperative in 7 cases (44%) and postoperative in 4 cases (25%). All were operated on performing Cholecystectomy in 5 patients (31.2%) Cholecystectomy with local wedge resection of the liver and lymphadenectomy in 4 cases (25%), Cholecystectomy with palliative gastrointestinal or biliary tract diversion ( hepatico-yeyunostomy, T-tube) in 3 cases, gastrointestinal diversion in 1 and Laparotomy alone in 3 cases with postoperative palliative stenting in one of them. The histology was adenocarcinoma in 87.5%, papillary carcinoma in 6.25% and squamous carcinoma in 6.25%. The staging was : stage II (Nevin ) 6.25%, stage III 25%, stage IV 12.5% and stage V 56.25%. The survival was of 3 months for patients in stage V, two patients with stage IV alive after 2 and 8 months from surgery. A 2 year survival was noted in patients in stage III and a 5 years survival in the only patient with stage II.
CONCLUSIONS: Gallbladder cancer is a seldom pathology with relatively nonspecific syntoms. Preoperative diagnosis often is done in advanced stages. There are relacionship between the degree of invasion of the layers of the gallbladder and the survival. Surgery could be curative in earlier stages.

### F022

CHOLELITHIASIS,GALLSTONE SIZE AND PATHOGENESIS OF PRIMARY GALLBLADDER ADENOCARCINOMA

I.Damtsios,P.Hatzigakis,S.Hantzisalatas,S.Dimas,H.Kafetzis, D.Kamilaris,S.Panayeas

Surgery Department-Polyclinic Hospital of Athens

This study was performed to asses the role of cholelithiasis in the pathogenesis of primary gallbladder adenocarcinoma.In a series of 1640 consecutive and unselected cholecystectomies that were carried out during the last 12 years, we have found 15 cases of primary gallbladder cancer histologically proven and we have studied the relationship between cholelithiasis and cancer.The age of the 15 patients with the carcinoma ranged between 58 and 82 years (average 69 years) and 4 of them were male,namely a M:F ratio 1:2,8. In 1606 out of 1640 cases of cholecystectomies there was cholelithiasis with a mean age of 71 years and a M:F ratio 1:3.In most of the cases with only cholelithiasis the size of the gallstones was smaller than 2.0cm.The histological type of the tumors in 13 cases was adenocarcinoma,in 1 leiomyosarcoma and in the last one Hodgkin´s lymphoma.Except for the patient with the leiomyosarcoma the other 14 had gallstones.In 3 of the patients with the adenocarcinoma the tumor was located in the gallbladder´s neck and the gallstones were multible with size from 1.0 to 2,5cm in diameter.In another 9 patients the tumor was located in the body of the gallbladder with one or two very large gallstones (diameter of 2,7 to 4,2cm).The location of the tumor in the remaining 3 patients was indeterminable and in the 1st of them with the lymphoma the size of the gallstones was very small(less than 0,5cm),in the 2nd one with adenocarcinoma there was a single very large gallstone, whereas the 3rd was the patient with the leiomyosarcoma and without associated cholelithiasis.In conclusion the results of this study support our hypothesis that the cholelithiasis plays a role in the pathogenesis of primary gallbladder adenocarcinoma and probably persons with large gallstones are of increased risk to develop this type of cancer.

### F023

ENDOSCOPIC BILIARY STENTING IN OBSTRUCTIVE JAUNDICE DUE TO METASTATIC CANCER

PPLO Coene, GNJ Tytgat, H Obertop, K Huibregtse. Depts of Gastroenterology and Surgery, Academic Medical Center, Amsterdam, The Netherlands.

Obstructive jaundice secondary to metastatic cancer is a dismal sign. Treatment is palliative and consists of biliary drainage. The efficacy of endoscopic drainage was evaluated in 124 consecutive patients with metastatic obstructive jaundice, representing 10% of all malignant biliary strictures. The mean age was 64 years (range 32-91), male/female ratio 0.97. Extrahepatic metastatic strictures comprised a heterogeous group of primary tumors: colorectal carcinoma 42, breast carcinoma 21, gastric carcinoma 17, pulmonary carcinoma 10, cervix carcinoma 7, lymphoma 6, hypernephroma 5, prostate carcinoma 4, hepatoma 3, esophageal carcinoma 2, unkown 7. Liver metastases were present in 24 patients (19.5%).
Results: Endoscopc retrograde cholangiography visualized a stenosis of the distal common bile duct (CBD) in 35%, the mid CBD in 18% and the hilum in 47%. Placement of a 10 Fg straight endoprosthesis was succesful in 123 patients (99%), with subsequent relief of jaundice and itching in 93% of 30-day survivors. Procedure-related cholangitis was encountered in 13 patients (11%), 30-day mortality was 25%. Median stent patency was 203 days, incidence of clogging 30%. The total number of stents required was directly correlated with length of patient survival (mean 1.3). The overall median survival was 91 days (mean 178 days, range 2-2,319), terminal clinical features were fever in 23% and jaundice in 69%. Patients with metastatic spread to the liver survived 67 days versus 113 days without liver metastases ($p<0.05$). Site of obstruction ($p=0.48$) and the primary origin of malignancy ($p=0.31$) had no bearing on survival. Adjuvant therapy was given to 12 patients (radiotherapy 5, chemotherapy 7); median survival of these patients was 355 days compared to 81 days without therapy.
Conclusion: Endoscopic biliary stenting can provide good palliation in patients with extrahepatic obstructive jaundice secondary to metastatic cancer.

### F024

SURGICAL MANAGEMENT OF PROXIMAL BILE DUCT TUMORS.

J.Poulantzas,M.Lorentziadis,S.Prigouris,S.Stylogiannis, L.Iosifidis,C.Kontaxis.
4th Depart. of Surg."Evangelismos"Hosp.& 1st Depart.of Surg "General Hospital" of Athens.

Conclusions:1)Resection of the tumor in the few cases where it is indicated is the only hope for longtime survival and cure and this is the case of well or moderately well differentiated carcinoma.2)From the palliative procedures employed, intrahepatic left hepaticojejunal anastomosis seems to offer better results than central hepaticojejunostomy while when intubation is indicated,internal intraluminal drainage has to be prefered from external via a T-tube or transhepatic intubation, because of the better quality of life it offers.

### F025

IS RADICAL SURGERY IN ADVANCED GALLBLADDER CARCINOMA JUSTIFIED ?

C. Bloechle, J.R. Izbicki, B. Passlick, R. Kuehn, X. Rogiers, C.E. Broelsch
Depart. of Surgery, University Hospital Eppendorf, Hamburg, Germany

Advanced gallbladder carcinoma is associated with a dismal long-term prognosis. The aim of this study was to evaluate the effectiveness of radical surgery in advanced stages of gallbladder carcinoma.
The course of 66 patients, that underwent surgery for advanced gallbladder carcinoma, was evaluated. 14% of the patients had stage II, 29% stage III, and 57% stage IV tumors. In 27 patients curative treatment was thought to be feasible: 12 patients underwent cholecystectomy and lymphadenectomy of the hepatoduodenal ligament. 17 patients underwent cholecystectomy combined with segment IV/V liver resection and lymphadenectomy. 10 patients underwent right extended hemihepatectomy. R0 tumor resection was achieved in 6 patients with cholecystectomy and lymphadenectomy, in 14 patients with cholecystectomy combined with segment IV/V liver resection and lymphadenectomy, and in all patients with right extended hemihepatectomy. R1 tumor resections were performed in 9 patients. Mean follow-up was 15.4 months (range 3 to 90 months).

Overall periopertive mortality was 1.5%, and morbidity was 20%. In R0 resections mean survival was 23.3, 25.0, and 26.3 months of the patients, who underwent cholecystectomy and lymphadenectomy, cholecystectomy combined with segment IV/V liver resection and lymphadenectomy, and right extended hemihepatectomy, respectively. After 24 months 46.4% of the patients with R0 resection were still alive compared to none of the patients with residual tumor. In the patients with R0 resection no difference in survival was detected when pN0 status was compared with pN1a, while the degree of dedifferentiation (G2/G3) influenced survival.
If complete resection is achieved radical surgical procedures including segment IV/V liver resection and extended right hepatectomy improve significantly survival rates with accepatable morbidity and mortality rates.

### F026

INTRAHEPATIC CHOLANGIOCARCINOMA : A WELL RECOGNIZED TUMOR

A. Valverde, A. Sauvanet, J. Belghiti, V. Vilgrain*, J.F. Fléjou**.
Departments of Digestive Surgery, * Radiology and ** Anatomopathology, Hôpital Beaujon, F-92100 Clichy.

Intrahepatic cholangiocarcinoma (IHCC) is a rare liver tumor, developed from the intrahepatic bile duct. The aim of this study was to show the features of this tumor, which may be mistaken with metastases of an adenocarcinoma.
From 1986, we retrospectively rewiewed the clinical courses of 20 consecutive patients (7 men, 13 women) aged from 33 to 72 years (mean: 56) resected. No patient had jaundice. Alphafoetoprotein level was elevated only in one case (>20N). Mean size of the tumor was 11 cm (range: 3-22), with peritumoral nodes in 5. Intrahepatic portal venous stenosis was present in all cases but without tumor thrombus. The tumor was hypervascularised in 5 cases and contained cystic components in 2 cases. According to the percutaneous preoperative biopsy pathologic diagnosis was adenocarcinoma with sclerosing features in all cases. Between 1986 and 1990, 6/9 were preoperatively classified as an unknow origin. Since 1990, preoperative diagnosis of IHCC was suggested in all cases (11/11) by clinical, radiological and histological characteristics. All patients had major surgical procedure with curative resection of more than 3 segments. and combined adjuvant chemo-radiotherapy for 7 patients. Actuarial survival at 12, 24 and 30 months was respectively 73, 44 and 22 %.
Conclusion: 1) IHCC, which usually occurs as solitary and large sclerotic adenocarcinoma, have specific clinical, radiological and histological features; 2) these features allow earlier diagnosis and treatment; 3) prognosis remains poor in spite of large surgical resections and adjuvant therapy should be evaluated.

### F027

**ENDOSCOPIC/PERCUTANEOUS ENDOPROSTHESES OR BYPASS SURGERY FOR PALLIATION OF MALIGNANT OBSTRUCTIVE JAUNDICE ?**

**G. Nuzzo, G. Clemente, A. Stracqualursi**
**Department of Geriatric Surgery - A. Gemelli Med. School, Rome (Italy)**

**In the last years, endoscopic or percutaneous placement of endoprostheses has become an attractive alternative to bypass surgery for relieving obstructive jaundice in patients with unresectable biliary or pancreatic carcinoma. Some randomized studies (1,2) failed to show significative differences between biliary stenting and surgical bypass regarding survival, complications and costs, taking into account readmissions for blocked endoprosthesis and gastric outlet. Nevertheless, the dilemma of "stent or surgery" remains (3). In this report a retrospective comparison between surgical bypass and non-operative palliation was performed in 34 consecutive patients with malignant obstructive jaundice.**
**From Jan '92 to oct '94, 46 patients presenting with malignant obstructive jaundice were admitted to our Department. There were 28 males and 18 females with a mean age of 65 yrs. All patients underwent ultrasonography and TC: 21 pancreatic cancers, 18 biliary neoplasms, 5 tumours of the ampolla and 2 recurrences of gastrointestinal malignancies were diagnosed. 12 patients underwent radical surgery (9 pancreasectomies and 3 liver resections) and were not considered for this study. 9 patients underwent surgical bypass (choledoco-duodenostomy or hepatico-jejunostomy) without morbidity and mortality. A non-operative palliation was performed in 25 pts by placement of an endoprosthesis (19 by ERCP and 6 by PTC) with a 32% morbidity and 12% mortality; 12 patients showed, into 3 months, at least one episode of cholangitis from blocking of endoprosthesis; 4 of these required, successively, a surgical bypass for repeated episodes (3 to 7) of cholangitis. Postoperative course was complicated in 3 of these 4 patients and 1 patient died.**
**In this series, an high incidence of procedure-related complications, resulting in failure of palliation, was found following biliary stenting. So, the initial advantage of a shorter hospital-stay was reduced by repeated admission for blocked endoprosthesis. Furthermore, 4 patients required, at second time, bypass surgery with an high incidence of complications. In conclusion, if the primary goal of the palliative therapy is to improve the quality of survival, bypass surgery is more attractive as an initial treatment option. Biliary stenting can be recommended for patients in whom a much brief survival might be expected.**

**REFERENCES:**
**1) Bornmann P et al: Lancet 1986,jan 11: 69-71**
**2) Andersen JR et al: Gut 1989, 30: 1132-5**
**3) Hatfield ARV: Gut 1990, 31: 1339-40**

### F028

ADENOMATOUS PANETH CELL HYPERPLASIA IN GALLBLADDER EPITHELIUM - UNUSUAL CASE

V. Katić, J.Gligorijević, V.Živković, M.Matijaš M. Spalević

Faculty of Medicine, Niš, Yugoslavia

We report microscopical characteristics,possible pathogenesis and significance the adenomatous Paneth cell hyperplasia in gallbladder epithelium.
Woman,58-year old, was opperated due to prolonged obstruction of the common bile duct,induced by impacted gallstone.
On surgical specimens of cholecystectomia,stained with HE, AB-PAS (AB pH=2,5), HID-AB and Trichrome,chronic calculous cholecystitis with acute exacerbation,was observed.
In addition,the incomplete type of the intestinal metaplasia was found in the surface epithelium.In the glandular epithelium the adenomatous tubules were filled with cytoplasmic Paneth granules and with sparse goblet cells containing neutral mucins.
On the basis of the presence of lysozyme content in Paneth cells,with its anti-infectious and anti-tumorous role, we have concluded the following:

1. Paneth cell hyperplasia is the sequence of faulty differentiation of a common stem cell induced by acute bacterial infection.
2. Paneth cells have anti-infectious and anti-tumorous role at the beginning,but later they became dysplastic and some of them neoplastic.

### F029

PERFORATION OF THE GALLBLADDER IN PARASITIC CHOLECYSTITIS

*T. El-Sefi, [2]M.Y.T. Rashed, [3]I. Boghdadi
*Liver Institute, Menoufiya University, [2]Faculty of Medicine, Alexandria University, [3]Faculty of Medicine, Menoufiya University, EGYPT.

Gallbladder(G.B.) perforation is a relatively uncommon complication of a variety of traumatic, inflammatory and neoplastic conditions. However, it has been seldom reported in parasitic infestation of the biliary tree. We report 3 patients with G.B. perforation due to Fasciola hepatica in 2 and intestinal schistosomiasis in one.According to Neimeir's classification, one was type I (F.hep.), one was type II (Schistosomiasis), and the remaining one was type III in the form of cholecysto-colic fistula (F.hep.). Ultrasound and Computed tomography examinations were helpful in the preoperative diagnosis of G.B. perforation in types I and II cases.Type III case was discovered accidently during surgery for biliary fascioliasis. Multiple nests of bile stained fragments of fasciola flukes were retrieved from the peritoneal cavity and the G.B. in types I&III respectively. Histopathological examination of the G.B. revealed acute parasitic granulomas with foci of suppuration at the site of perforation in all cases in addition to a heavy deposition of Schistosoma mansoni eggs at the the site of perforation in the case of schistosomiasis.All cases were treated by cholecystectomy followed by praziquantel therapy. There was neither mortality nor major morbidity.
We conclude that heavy infestation with fasciola flukes and intestinal schistosomiasis is another risk factor for G.B. perforation and great awareness of such complication is needed particularly in endemic regions.

### F030

GANGRENE OF THE GALLBLADDER

V. Skountzos, G. Antonopoulos, G. Kyratzis, L. Papastamatiou
2nd Dpt. of Surgery "Apostle Paul" Hosp-KAT: ATHENS-HELLAS

Gallbladder gangrene consists a serious pathological condition mainly due to delayed diagnosis and treatment. In numerous cases the overwhelming course of acute cholecystitis causes necrosis within 24-36h, especially the acalculous type.

During the last 10 years 47 patients with gangrenous cholecystitis out of 198 cases of acute cholecystitis (23.7%) were treated. Sex distribution was men 1:1.2 female and mean age 65.8y (17-102y). On 10 of them (I.C.U. patients) gangrene was the early result of acalculous cholecystitis. A mean delay time of 36 hours from the onset of symptoms until admission was noticed in the other 37 cases. Diagnosis was based on clinical symptomatology and ultrasonographic findings, within less than 24 hours.All patients underwent emergency intervention. Cholecystectomy was achieved in all cases and in 3 of them suppurative cholangitis obliged to bile duct exploration. Another 12 patients required I.C.U. treatment postoperatively (12:37=32.4%) for 2-5 days.

There were no deaths in these series. Four out of the 10 I.C.U. patients died for reasons not connected to the disease or the operation. General morbidity rate was high (46.8%) while in 6 patients septic intraabdominal complications were developed: One subphrenic abscess drained under CTscan, 3 subhepatic collections -CT drainage 2, operative drainage 1 - and 2 bile leakages treated conservatively.

It is concluded that prompt recognition and early operative treatment of acute calculous cholecysitits, improves percentages of gallbladder gangrene. Necrosis however occurs as early result in cases of fulminant course of the infection involving circulation. The low percentage of acute acalculous type is due to our policy i.e. preventive cholecystectomy during emergency laparotomy in multi-injured patients, necessitating I.C.U. treatment.

### F031

DIAGNOSIS AND THERAPY OF CHOLOPERITONITIS

A.Koussidis,M,Apsokardou,G.Koussidis
Department of Surgery,General Hospital of Chios Greece.

The aim of this work is to show the urgent diagnosis and surgical treatment of choloperitonitis. This is caused by perforation of the gall bladder of the choledochal duct,and from the beginning of the small intestine.Patients are often brought in to hospital delayd,therefore their general condition has been worsened.The diagnosis is mainly based on clinical symptomatology and on the puncture of the abdomen which,in all cases,turns out gall bladder fluid and puss.

PATIENTS AND METHODS

From 1992 to 1993 four patients,suffering from heavy choloperitonitis,were operated on.The perforation of the gall bladder in one patient was due to the increase in pressure in the gall bladder from a large stone stuck in the doudenal part of the choledochal duct.In a second patient the perforation of the gall bladder was duc to the necrosis of the gall bladder's wall due to necrotic cholecystitis.The third patient presented perforation of the choledochus duct from necrosis of the choledochus duct wall due to acute cholangitis.The forth of the patients presented choloperitonitis from perforation of the initial part of the small intestine.The age of the patients ranged from 64 to 84.All had good postoperative progress and were dischaged between the 7th and 12th post operativ day.

CONCLUSION

Choloperitonitis is a very serious illness with a high rate of mortality.The good preoperative preparation,the diligent surgical treatment and the very good post operativ care,assure the survival of the patients with full recovery of their halth.

### F032

ACUTE CHOLECYSTITIS. THE SURGEON'S POINT OF VIEW THROUGH LAST DECADE.

P. Karydakis, S. Pierrakakis, E. Bobotis, G. Douridas, J. Marinakis, G. Antsaklis.
Surgical Department, Sismanoglion General Hospital, Athens - Greece.

Over the last two decades several changes have occured in the diagnosis and managment of acute cholecystitis. The purpose of this retrospective study was to point out the surgical philosophy concerning acute cholecystitis by demonstrating the differences between two groups of patients operated on during the last 13 years.
One hundred and eighty four operations for acute cholecystitis were performed from 1981 to 1986 ( group I ) and compared to 591 cases operated from 1988 to 1993 ( group II ). The following significant differences were observed in group II : a) an increase in the age of patients operated on for acute cholecystitis, b) a higher percendage of diabetics, c) an increase in the number of patients operated on within 72 hours of onset of symptoms, d) an increase in the incidence of acalculus cholecystitis and gangrenous changes in the gallbladder, e) a decrease of the average hospital stay by 1.2 days. The mortality was less than 3% with no significant difference between the two groups.
The results of this study support the safety and efficacy of early syrgery as a semielective operation for patients with uncomplicated acute cholecystitis.

### F033

### RISK FACTORS OF POSTOPERATIVE INFLAMMATORY COMPLICATIONS IN PATIENTS WITH ACUTE CHOLECYSTITIS

B.S.Briskin, Z.I.Savchenko, N.N.Khachatrian, O.G.Sosnovikova
Department of Surgery, Institute Semashko, Moscow, Russia

Operations upon the gallbladder and biliary tract in patients with acute cholecystitis are the most frequently performed procedures on the digestive system,that's why the problem of pyoinflammatory complications and their prevention is particularly important.Postoperative infections occured in 16% of the 180 patients with acute cholecystitis, just over half being wound infections.

The incidence of wound infection and other septic complications depends on,among other things,clinical risk factors such as the presence of diabetes, jaundice,the performance of choledochotomy, the presence of accompanying diseases.Patients over 70 years of age also have a higher incidence of postoperative complications.The risks of wound or infra-abdominal infection are significantly increased by the presence of bacteria in the bile.

We prospectively studied the influence of following laboratory risk factors: the condition of the immune system,interleukins,endocrine factors on the prognosis in patients with acute cholecystitis, their influence on the final outcome.

Our results showed reduced T-lymphocytes,T-helper cell percentages and functional activity, decreased function in natural killer cells more expressed in patients with complicated courses.Complicated forms of disease are characterised by high ACTH,insulin and low T3,T4 levels 3-5 days after operation.Quantification of T-lymphocyte proliferation in response to T4,cortisol is reduced in patients with complicated courses.

Patients with various clinical and laboratory risk factors are needed in the immunocorrective therapy to prevent the development of different pyoinflammatory postoperative complications.

Certain immunomodulators,such as thymogen,tactivin,thymalin,human immunoglobulin preparations for intravenous use, given to high risk groups of patients will be able to restore immunologic and endocrine competence. The choice of the most suitable immunomodulators for profylaxis depends on the condition of immune system and severity of inflammation.

With the use of immunomodulators in the complex treatment in patients with acute cholecystitis the results of therapy are improved,the incidence of postoperative complications is decreased,and the period of hospital-stay decreases by 3 days on the average.

### F034

### ACUTE CHOLECYSTITIS - HISTOPATOLOGICAL LESSIONS AND BACTERIOLOGY FINDINGS

V.Jeremic,M.Sukalo,M.Mitrović,D.Raderkovic,A.Karamarkovic

UNIVERSITY CLINICAL CENTER, CENTER FOR EMERGENCY SURGERY,BELGRADE

YUGOSLAVIA

Acute cholecystitis has become the most common indication for major abdominal surgery. The presence of bacteria in bile during acute cholecystitis is widely regarded as a secondary phenomerion, the bile is initially sterile and becomes infected after the onset of the inflammatory reaction. Close correlation was found between postitive bile cultures and the incidence of septic complications, which play an increasingly important role in the morbidity and mortality of biliary tract disease and biliary surgery.

### F035

### COMMON BILE DUCT EXPLORATION IN EMERGENCY OPERATIONS FOR ACUTE GALLBLADDER DISEASE.

G. Antsaklis, P. Karydakis, S. Pierrakakis, N. Economou, G. Douridas, J. Marinakis.
Surgical Department, Sismanoglion General Hospital, Athens - Greece.

The incidence of common bile duct (CBD) exploration during the last decade has been declined, due to the use of other non surgical methods, mainly on patients without acute disease of the extrahepatic biliary tract, who have been operated electively.

In emergency surgical treatment, in acute gallbladder disease, the percentage of CBD exploration has but little changed, in the last 10 years.

In a period of 8 years (1986 - 1993), in a total of 2.498 operations for extrahepatic biliary tract calculus disease, 761 patients (30,5%) were operated on urgently. There were 121 CBD explorations and positive findings revealed in 101 patients (83%).

Per-operative cholangiography has to be performed whenever possible, and is of great help in the decision for CBD exploration.

### F036

### EARLY SIGNS OF MULTIPLE ORGAN FAILURE AT SEPTIC CHOLANGITIS

E. Galperin, G. Akhaladze
I.M. Sechenov Moscow Medical Akademy, Russia

During 1987-93 clinical signs of multiple organ failure (MOF) were cosidered in 134 out of 348 pts with septic cholangitis. In 96 pts the earliest manifastation of MOF was a hyperkinetic state of central haemodynamics on sonocardiography with cardiac output (CO) 8.5+1.5 l/min and total vascular resistance (TVR) 985.0+315.2 din/cm/c. At CO 8.7 l/min and TVR 600 din/cm/s 8 out of 16 pts died, and at CO 11.2 l/min and TVR 573.9 din/cm/c - 5 out of 7.

Ultrasonic doppler investigation of portal blood flow (PBF) in 79 MOF pts with septic cholangitis we found a decreased fasting PBF (438.2+21.3 ml/min vs 900-1200 ml/min in a group of healthy volunteers) with a considerable decrease of the PBF index after a histamine load (0.7+0.12 vs 2.12+0.3 in healthy). These data were accompanied with a refractery responce of bilirubinemia to biliary tree decompression (mean value = -0.126+0.021 vs -0.9 in controls). Bile excretion in these pts was 215.0+130.7 ml/day.

Study of lymphocyte subpopulations in 28 pts with septic cholangitis rvealed that if in autoplasm CD11+=61.95+15.3, CD4+=18.5+3.1, CD8+=10.3+5.1 and B7+=14.1+2.7, ransfer of these cells into the neutral bovine serum albumin led to a considerable increase in quantity of superficial receptor expression on lymphfocyte membrane: CD11+=83.1+9.8, CD4+=32.3+ +5.8,CD8+=49.1+8.2, and B7+=32.3+5.3 (in all groups p). These data idicate, that depressed expression of lymphocytes' superficial receptors is caused by endotoxemie at septic cholangitis.

In conclusion, septic cholangitis leeds to MOF with early signs as a disturbance of central and organ hemodynamics and depression of lymphfocytes superficial receptor expression.

### F037

ENDOSCOPIC RETROGRADE CHOLANGIOGRAPHIC PATTERNS OF BILIARY FASCIOLIASIS

*T. El-Sefi, [2]M.Y.T. Rashed, [3]I. Boghdadi
*Liver Institute, Menoufiya University, [2]Faculty of Medicine, Alexandria University, [3]Faculty of Medicine, Menoufiya University, EGYPT.

The cholangiographic patterns of 14 patients with biliary fascioliasis were reviewed to define its characteristic features. The diagnosis was confirmed in all cases by retrieval of fasciola flukes from the biliary tree either endoscopically and/or surgically. In all patients, the bile ducts were dilated with one or more of the following cholangiographic patterns:
1) Whip-worm like filling defects (7/14).
2) Single or multiple leaf-shaped filling defects (4/14).
3) Irregular changing filling defects on serial films (3/14).
4) Jagged irregular bile ducts wall (5/14).
5) Segmental main bile duct stricture (2/14).

The gallbladder was infested in 9(64%) patients. The gallbladder filling patterns included:
1) Dry cracked earth appearance (4/9).
2) Numerous whip-worm like filling defects in the fundus or at the neck (2/9).
3) Vague ill-defined filling defects with jagged irregular wall (2/9).
4) Contracted small-sized gallbladder (1/9).

Associated stones were found in the bile ducts in 3 patients and in the G.B. in 2 patients.

In conclusion, these cholangiographic patterns should be considered as specific for the diagnosis of biliary fascioliasis.

### F038

**ENDOSCOPIC SPHINCTEROTOMY FOR THE TREATMENT OF POSTOPERATIVE BILIARY FISTULAS**

**Y. Tekant, O. Bilge, K. Acarlı, A. Alper, A. Emre, O. Arıoğul**

**Hepatopancreatobiliary Surgery Unit, University of Istanbul Medical Faculty, Istanbul, Turkey.**

**Postoperative biliary fistulas continues to be an important problem in liver surgery. Our results of endoscopic treatment of these fistulas are presented.**

**Between March and October 1994, 7 patients underwent endoscopic sphincterotomy for the treatment of postoperative biliary fistulas in the Hepatopancreatobiliary Surgery Unit of the University of Istanbul Medical Faculty in Istanbul, Turkey. Six of these patients were male and one was female with a mean age of 42 years (range19-54). Five patients had undergone liver surgery for hepatic hydatid disease, while one was operated on for alveolar echinococcosis of the liver and one for liver abscess. The mean daily output of the fistulas was 400 cc (range 200-900). Endoscopic sphincterotomy was performed between 5. and 8. postoperative days in 5 patients, and on the 30. and 39. days in the remaining two patients. Fistulous output decreased by 50% in the first two days after treatment in 6 patients (86%) and their fistulas resolved in a mean of 7 days (range 2-14 days), while no effect was observed in a patient treated on the 30. day.**

**In conclusion, endoscopic sphincterotomy may be an effective minimally invasive solution for the treatment of postoperative biliary fistulas.**

### F039

SPECIFICITY AND POSITIVE PREDICTIVE VALUE OF ECHOENDOSCOPY FOR COMMON BILE DUCT STONES. (A PROSPECTIVE STUDY FROM FRENCH SURGICAL RESEARCH ASSOCIATIONS).

Chipponi J, Montariol TH, Julienne P, Moka A., Charlier A, Hay JM..
Hôtel-Dieu, B.P. 69, 63003 CLERMONT-FERRAND FRANCE

The aim of this prospective multi-centre study was the evaluation of the performance of echoendoscopy (EE) compared to per-operative cholangiography (POC) in the exploration of common bile duct (CBD) stones before any surgical intervention.

From October 1992 until March 1994, 177 patients with symptomatic gallstone disease and a Huguier and Lacaine score of 3.50 or more were included in the study. All patients underwent an EE, a cholecystectomy, a POC and an instrumental exploratin of the CBD if indicated. The principal judgement criterion was the POC, with the finding of a stone during operation or at endoscopy being secondary criteria.

EE was performed in 175 cases (99 %), POC in 156 cases (88 %), and the 154 patients who underwent both investigations were analysed. Thirty patients had a stone in the CBD (20 %). There were 4 to 6 false positives (the CBD was not explored in 2 cases) and 4 false negatives for EE, whereas there was only 1 false positive for POC. The specificity was 0.95 for EE and 0.99 for POC, with positive predictive values for EE at 0.81, and for POC at 0.97. These two helpful indices did not depend on possible false negatives for the POC and so could not have been altered by the study method. A positive EE indicated an 81 % risk of CBD stones, whereas a positive POC carried a risk of 97 %.

EE is more feasible than POC, but the diagnostic value of POC is certainly superior to that of EE. POC therefore remains the recommended investigation for CBD stones, although the good results obtained with EE make it a useful pre-operative or second-line investigation.

### F040

**THE VALUE OF THE SIDE VIEW DUODENOSCOPY AND COMBINE RETROGRATE CHOLANGIOPANCREATOGRAPHY IN THE DETECTION OF THE DIVERTICULA OF THE SECOND PART OF THE DUODENUM**

Surgery Department-Polyclinic Hospital of Athens
I.Damtsios,P.Hatzigakis,K.Mustafa,S.Hantzisalatas,G.Kekos

Most of the duodenal diverticula are located in the second part of the duodenum and the determination of the precise position of them and the relationship between a duodenal diverticulum and the papilla of Vater is valuable in order to perform surgical prodecures in this area.The aim of this study was to asses the diagnostic value of the side view duodenoscopy and the retrograde cholangiopancreatography (ERCP) for establishing the relationship between the diverticula of the second part of the duodenum and the papilla of Vater.The study comprised 21 cases of diverticula of the descending part of the duodenum with a mean age of 67 years and male-female ratio 1,1:1.All the patients underwent lateral duodenoscopy and retrograde cholangiopancreatography and 6 of them additional X-ray examination of the upper gastrointestinal tract.In none of the 6 patients the barium meal examination revealed the precise position of the diverticula and the internal relation between the diverticulum and the papilla of Vater.On the contrary the side view duodenoscopy combined with retrograde cholangiopancreatography showed that in 15 patients the diverticula were peripapillary,in another 2 parapapillary and in 1 patient with two diverticula in the descending part of the duodenum,the first deverticulum was peripapillary and the second one was located distal to the papilla of Vater.In the remaining 3 cases the diverticula were located at the end of the descending part of the duodenum and were diagnosed and detected with X-ray examination and side view duodenoscopy.The conclusion of this study is that the side view duodenoscopy combined with retrograde cholangiopancreatography is the method of choise for establishing the relationship between a diverticulum of the second part of the duodenum and the papilla of Vater.

### F041

**LAPAROSCOPIC ENDOSONOGRAPHY IN THE DETECTION OF CBD STONES**

D. Lomanto, M.Nardovino*, M.Di Girolamo, A.Paganini**, M.Sottili, F.Carlei ***, E. Lezoche**
II Clinica Chirurgica University of Rome "La Sapienza"; * Hepato-Biliary Division, INI Canistro, (AQ); ** Patologia Chirurgica University of Ancona and *** Dept.of Medicina Sperimentale, University of L'Aquila.

Laparoscopic approach has become the *gold standard* to perform cholecystectomy (LC). This surgical procedure, however, needs that i.o. cholangiography (IOC) be performed simultaneously to better define the anatomy of the biliary ducts and the presence of bile ducts stones (11% of important informations) but, nevertheless the high sensitivity, in about 3% of pts, due to anatomically or technically reasons, was impossible to perform.Theferore, in false positive or in doubtful cases surgeon is obliged to perform an unjustified and risky bile duct exploration. To reduce and to better define intraoperative diagnosis we evaluate, during laparoscopic biliary surgery, the role of i.o. ultrasonography (IUS) advocating for the unquestionable advantages offered by its non-invasiveness, high spatial resolution and no-need of contrast medium injection. 60 patients (36F; 24M - Mean age 45 ± 16) with gallstones and no pre-op. evidence of CBD stone, underwent LC. After IOC, all patients were submitted to IUS. we utilize a 10 mm in diameter linear probe with a 7.5 MHz transducer side-mounted, 38 mm in lenght. Longitudinal and transverse scans was obtained through the umbilical or right subcostal approach. Mean duration of IUS was 4 min. As far as concern the definition of the anatomy of the biliary tree and the adjacent organs, IUS allowed an optimal visualization in 57 patients (95%), whereas in 3 pts (5%) the study was limited in one case due to anatomical abnormality and in two pts for multiple adhesions. In one patient, US allowed the visualization of the biliary tree that intraoperative cholangiography failed to demonstrate because of an extrinsic gallbladder compression and confirmed in 2 cases the presence of an hepatic angioma.
Both techniques clearly demonstrated extrahepatic bile duct stones in 4 cases (6,7%). In one case which was positive at intraoperative cholangiography the method allowed the biliary duct filling defect to be referred to an artifact caused by the presence of an air bubble. In conclusion, IUS proved to be a reliable technique in the intraoperative study of the biliary ducts morphology and can be considered as a procedure complementary to IOC.

### F042

**J. Doseděl, P. Sedláček, S. Černý, M. Cholt:** **Endoscopical treatment of complications after laparoscopic cholecystectomy**

Hospital of Merciful Sisters of St. Charles Boromeus in Prague

Czech Republic

### F043

ENDOSCOPIC RETROGRADE CHOLANGIOPANCREATOGRAPHY: A PERSONAL OR DISCIPLINE DEPENDENT PROCEDURE?

J.A.Paraskevopoulos, A.R.Dennison, H.Smart, W.E.G.Thomas
Endoscopy Unit, Royal Hallamshire Hospital, Sheffield, UK

The aim of the present study was to investigate whether there is a difference (diagnostic or interventional) in ERCP performed by clinicians of various disciplines. We have, therefore, reviewed 600 patients who had had ERCP, 300 by a physician and 300 by a surgeon, in our Unit over a 3 year period.
The mean age of the surgical patients was 63 years (range 9-96) and of the medical 59 years (range 17-98). There were 173 females (58%) in the surgical group compared to 155 (52%) in the medical group. The indications for ERCP were similar: pain 67 v 74, jaundice 121 v 141, previous pancreatitis 47 v 24, CBD stones 29 v 14, duct check 5 v 8, blocked stent 3 v 1 and other 28 v 38, surgical v medical, respectively. Diagnoses were also very similar: stones 104 v 92, CBD tumour 10 v 30, CBD dilatation 34 v 28, respectively. There was no significant difference in failure to perform ERCP, 24 v 26, respectively. The complication rate was also the same. There were however significant differences ($p<0.001$) in the procedures performed: 135 (45%) v 247 (82.3%), surgical v medical, respectively (Table 1).

These results indicate a different management approach. Whether this is a personal or discipline difference requires further study.

**Table 1.**

| Procedure | Surgeon | Physician |
|---|---|---|
| * Sphincterotomy | 99 | 117 |
| * Stent/stent change | 11 | 41 |
| * Stone extraction | 14 | 81 |
| * Nasobiliary tube | 11 | 8 |

### F044

**Percutaneous transhepatic expandable metallic endoprostheses in metastatic billiary obstraction.**

*I.S.Kaskarelis, K.Malagari, P.Abatzis, I.Minardos, M.Natsika, K.Roubis, Z.Fasoulas, S.Kouris.*
**Department of Radiology, Evangelismos Hosp. Athens Greece.**

**Purpose:** This study is to evaluate the efficiency of percutaneously inserted metallic endoprostheses in selected patients with metastatic biliary obstruction.
**Material and Method:** During 1994 we treated 14 patients. A total of 20 stents was placed (13 Wallstent, 4 Accuflex, 3 Gianturco). They were 11 men and 3 women, with a mean age of 65 years (age range 42 - 80). The causes of malignant bile obstruction were, pancreatic carcinoma (5), gallbladder carcinoma (4), stomach carcinoma (4), swannoma (1). The percutaneous transhepatic implantation of stents was performed in a one stage procedure (6 cases) or following P.T.B.D. (8 cases).
**Results:**
1.Successful stent inserting in all cases (14/14).
2.In 6 patients we implanted 2 stents.
3.Early complication in 2 cases (haemorrhage, treated conservatively).
4.The 30 day mortality rate was 7,8%.
5.Five patients of our study died with a survival average 88 days (25-183). The cause of their death was the primary disease, without evidence of stent obstruction.
6.In 2 patients we don' t have follow up more than 30 days.
7.Seven patients remain alive after an average follow up of 89,1 days (range 40-255 days) with no recurrence of obstruction so far.
**Conclusion:** Percutaneous placement of the metallic endoprostheses is effective in palliation of inoperable metastatic billiary tree obstruction, especially when other palliative methods have failed.

### F045

**PERCUTANEOUS STENTING FOR CHOLANGIOCARCINOMA RESULTS PRIOR TO EXPANDABLE METALLIC STENTS**

N Doctor, Helen Whiteway, A Salamat, J Dooley, R Dick, B Davidson.
Hepatobiliary Unit, Royal Free Hospital, London

Expandable metallic stents may provide superior palliation for symptomatic patients with cholangiocarcinoma(CCA). To allow proper evaluation we analysed the results with plastic stents prior to their introduction.
We studied 14 patients (10 male) aged 27-84 (mean 65.3) with hilar (64%) or distal (36%) CCA with tissue confirmation in 10 (biopsy(7), cytology(3)). All were symptomatic. Percutaneous stenting was successful in 11 (80%). The three failures had endoscopic stenting (1) or surgical bypass(2). In hospital complications occurred in 7 (50%). Cholangitis in 5 settled with antibiotics alone in 3, required stent change in 2 and was associated with renal failure leading to death in 1 patient (Overall mortality rate 7%). Haemobilia in 2 patients stopped spontaneously. Of the 13 patients leaving hospital with palliation of symptoms (93%), follow up information was available in 8 (6-40 months). Three have required stent replacement which has been successful endoscopically.
This study has provided contemporary data for comparison with expandible metallic stents for palliative treatment of cholangiocarcinoma.

### F046

**RESULTS OF ROUX-EN-Y HEPATICOJEJUNOSTOMY FOR BENIGN BILIARY STRICTURE**

Wig JD, Gupta NM, Mohabeer VT, Khanna SK
Department of Surgery, Postgraduate Institute of Medical Education and Research, Chandigarh, India, Pin-160 012.

Roux-en-Y hepaticojejunostomy remains the standard treatment for post-cholecystectomy bile duct strictures. The objective of the present study was to evaluate the results of biliojejunal anastomosis. Ten patients were studied (one male, 9 females), follow-up period ranged from 7 months to 7 years (mean 32.4 months). Parameters studied were LFT, US and barium meal study and radioisotope study using $^{99}Tc_m$ labelled BULDIA. One patient had symptoms of peptic ulcer, one had cholangitis and one patient had loose motions and loss of weight. LFT were normal (n = 9), one patient had elevated alkaline phosphatase and SGOT, SGPT. On US, dilated IHBR were seen in only one patient, in others no dilatation was seen. In one patient no tracer was seen in the gut even after 2 hours, while in others there was appearance of tracer in the bowel after 45 minutes. Overall 9 patients had excellent to good results. One patient had cholangitis but did not require any intervention - surgical or radiological. We conclude that hepaticojejunostomy is a good procedure for benign biliary disease and was associated with good results in our experience.

### F047

RESULTS OF RECONSTRUCTION OF BENIGN BILE DUCT STRICTURES

R. Čolović, D. Bilanović, V. Kalezić, M. Šukalo, S. Matić, M. Perišić

1st Surgical Clinic of the Institute for Digestive Diseases, Clinical Center of Serbia, Belgrade, Yugoslavia

### F048

**ASSESSMENT AND MANAGEMENT OF POST OPERATIVE BILIARY STRICTURES AND FISTULAE**

**T.K. Neelamekam, E. Brazil, S. Attwood, O. Traynor**

**Liver Unit, St. Vincent's Hospital, Elm Park, Dublin 4**

### F049

BENIGN BILIARY STRICTURES

M.Jeremić,M.Stojiljković,M.Stojanović,

V.Pejčić,A.Bogićević,Z.Rančić

Surgical clinic.Clinical center -Niš,Serbia, Yugoslavia

The aim of thisstudy is to analyze the results and experience of 31 cases of benign biliary stricture surgically treated in the period of 1983-1993.In this period 4o57 operations on biliary tract were performed.The frequency of operations for biliary strictures was o,76%.The causes of the strictures were postoperative stenosis after operations on biliary tract(23 or 74,19%),after gastric resection(4 or 12,9o%),after reconstructive operations of biliary tract(2 or 6,45%) and persistent gallstones in the biliary tract (2 or 6,45%).We performed:hepaticojejunostomy by Roux-en-Y in 23 cases (74,19%),hepaticojejunostomy by Lachey method in 5 cases (16,13%) and choledochoduodenostomy in 3 cases(9,67%).Follow-up og patients was from one to ten years.Recurrent cholagitis ocurred in two patients (one after choledochoduodenostomy and one after Lachey procedure).Biliary fistulas ocurred in two patients and one needed a reoperation.One stenosis of choledochoduodenostomy required a conversion to hepaticojejunostomy by Raux-en-Y.The postoperative mortality was 3,22%.One patient died due to septic shock with multiorgan failure.
In our opinion benign biliary stricture are an impotrant problem in biliary surgery which can be resolved with correct reconstructive surgical menagement.Hepaticojejunostomy by Roux-y is in the long-term the best reconstructive procedure.

### F050

HIGH MUCOSA-TO-MUCOSA BILIARY-ENTERAL ANASTOMOSIS WITHOUT STENTING FOR BENIGN (BISMUTH 3-5 TYPE) HEPATIC DUCT STRICTURE

E.Galperin, N. Kuzovlev
I.M. Sechenov Moscow Medical Academy, Russia

365 surgical repairs were performed for binign bile duct strictures (Bismuth I-5 types) during 1979-1993. Type 3 - 5 represent the most difficulty for the surgeon. For many years we used long term (2 years) transanastomotic ring stent. From 1886 we prefer mucosa-to-mucosa biliary-enteral anastomosis without stenting (was perfomed in 84 pts out of 135 and in recent years this ratio was 1:7).

71.2% pts had previously undergone surgery for iatrogenic injury or strictures, 72.6% had recurrent cholangitis, 7.1% - subphrenic and subhepatic abscesses, 26.2% - external biliary fistula.

Peforming high mucosa-to-mucosa biliary enteral anastomosis we tried to free hepatic ducts, above the stricture, wich was anastomosed with a 80-100 cm Roux-en-Y jejunal loop, using one-row of sutures with knots left outside.

No fatal outcome was recorded and the complications (12) were: subphrenic abscess -1, temporary biliary fistulae -4, intraabdominal bleeding -3, intestinal ileus -1). In long-term follow-up only one pt had recurrent stricture (8 years).

Our experience shows, that in benign biliary stricture (type 3 - 5) acurate dissection of bile ducts the above stricture scar using modern sutur materials diminishes the risk of stricture recurrence.

### F051

NON-OPERATIVE PERCUTANEOUS TREATMENT OF BENIGN, POST-SURGICAL BILIARY STRICTURES

M. Colledan**, M. Marinoni*, G. Paone**, M. Raule*
*Institute of General Surgery, University of Milano, Italy
**Liver Transplant Unit, Ospedale Maggiore I.R.C.C.S., Milano, Italy

Non-operative percutaneous procedures (NOPP) have been increasingly used in recent years for the tratment of benign, post-surgical biliary strictures (BS), with encouraging results, we report here our experience in this field. Nine patients, with age ranging from 18 months to 64 years, underwent NOPP for the treatment of BS at our institutions. In six patients the BS was at the site of a previous biliary anastomosis. These were: 1 choledoco-choledochostomy and 3 choledoco-jejunostomies in patients with liver transplantation and 3 choledocho-jejunostomies performed after pancreatoduodenectomy, at surgery for recurrent bile stones and for a surgical injury of the bile duct respectively. The remaining 2 patients had a stricture of the common hepatic duct and of the low common bile duct respectively, secondary to surgery for lithiasis. Four patients had large stones above the BS. All the patients underwent percutaneous transhepatic biliary drainage and repeated sessions of balloon dilation. The patients with stones underwent wash-out and balloon manipulation, preceeded by shock wave lythotripsy in 3 of them. An expandable metallic endoprosthesis was left in place in 2 patients while a plastic one in another. Three complications were observed: a pleural effusion in 2 patients, treated by thoracentesis, and the accidental intrahepatic rupture of a biliary catheter, treated by removal of the distal fragment. Infection of the catheter was demonstrated in all the patients by positive cultures and treated, although in no case it was clinically evident. No mortality was observed. In only 1 case the NOPP were unsuccessful: this was a 57 years old woman, treated for BS with stones, 41 months after a liver transplantation, who had already developed secondary sclerosing cholangitis and eventually required a retransplantation. In the remaining 8 patients NOPP were successful and no further treatment was required. One of them was lost to the follow-up after 2 years, another has a recurrent hycteric C hepatitis after 30 months, 6 patients are alive and well with good liver function with a mean follow-up of 3.1 years (range: 1 month-7 years). Our experience confirms the safety and effectiveness of NOPP for the treatment of BS. Although the cost of these procedures is high, they can be recommended, as a first-choice alternative to surgery, at least for high risk patients. Availability of a broad spectrum of instruments, devices and facilities is necessary to optimize results.

### F052

THE CONTRIBUTION TO DIAGNOSIS AND TREATMENT OF MIRIZZI SYNDROME (M.s.)

M.Ryska,J.Skála,F.Bělina,J.Doseděl

Surgery and gastroenterology departments, Charles University, Prague, Czech republic

In 1948 there was described uncommon cause of cholestasis by P.L.Mirizzi: extraductal partial compression of common hepatic bile duct due to stone in gallblader neck or in cystic duct or to surrounding inflammation, so called "syndrome anatomo - functionnel. Clinical signs are expressed by jaundice and recurrent cholangoitis. 4 types of M.s. syndrome are distinguished in literature. During last 6 years we treated 11 patients with M.s. All of them had M.s. diagnosed before operation. There is very important for surgeon to determine the place of bile duct obstruction and its nature before operation. Ultrasonography shows usually dilatation of intrahepatic bile ducts and normal choledochus. ERC alone or with PTC are able to inform exactly about stenosis as well as the character of it and about the present of cholecystobiliaric fistula. Six patients with M.s. are presented.

### F053

**SPONTANEOUS INTERNAL BILIARY FISTULAS: STUDY OF 13 CASES**

M.A.C. MACHADO, J. JUKEMURA, P. VOLPE, E.E. ABDO, S. PENTEADO, T. BACCHELLA, J.E.M. CUNHA, M.C.C. MACHADO, H.W. PINOTTI.
Department of Surgery - University of São Paulo, Brazil.

Spontaneous internal biliary fistulas represent a rare pathology, but an important one given the diagnostic difficulties and high operative mortality. This entity is rarely recognized preoperatively. Ultrasonography and intravenous cholangiogram merely demonstrate alterations of cholelithiasis while plain abdominal radiograph biliary gas may show evidence of enterobiliary fistula formation. The aim of this study is to describe the diagnostic features and therapeutical options in the management of spontaneous internal biliary fistula. We report our findings in 13 patients with spontaneous internal biliary fistula collected from bile tract surgery performed in our center over a eight-year period. Ten patients were women and three were men. The mean age was 55.2 years (range, 30 to 87 years). The etiology was cholelithiasis in all cases and the most frequent type of fistula was cholecystoduodenal. The incidence was 1.21% of the total number of cases. In each case the surgical management depended on the etiology, clinical manifestations and status of the patient. The mortality was 0% and morbidity was 23.1%. The complete clinical nonspecificity of the fistulas and their frequent association with choledocholithiasis are emphasized.

Spontaneous internal fistulas of the bile tract are severe complications of cholelithiasis. Late detection and treatment of diseases of the biliary system is the main cause of formation of the fistulas. Removal of the internal biliary fistula as well as correction of abnormal changes in the bile ducts is the object of the operative intervention.

### F054

**IATROGENIC BILE DUCT LESIONS FOLLOWING CHOLECYSTECTOMY-SURGICAL OR ENDOSCOPIC TREATMENT?**

S.B. Hosch[1], F. Thonke[2], W.T. Knoefel[1], K.F. Binmoeller[2], J.R. Izbicki[1], C.E. Broelsch[1]

Dept. of Surgery[1], and Dept. of Endoscopic Surgery[2], University of Hamburg, FRG

Introduction: Endoscopic and surgical approaches are established in the management of iatrogenic common bile duct (CBD) injuries following cholecystectomy. This retrospective study compares outcomes of surgically and endocopically managed patients.
Patients and methods: Included are 58 consecutive patients that were treated for iatrogenic bile duct lesions primarily either by surgery (n=17) or by endoscopic means (n=41) over the last 10 years. Primary surgical treatment was performed if on ERCP a total transsection (n=13) or non-negotiable stenosis (n=4) of the CBD was evident. Primary endoscopical treatment was performed in cases of bile leak (n=18), and negotiable stenosis (23). Follow up ranged from 2 to 125 months and was obtained by exams in the outpatient clinic or telephone interview using a questionnaire.
Results: Mean interval between cholecytectomy and treatment was 12 weeks (endoscopy) and 5 weeks (surgery). Endoscopic treatment consisted of stenting in 35 patients, Histoacryl sealing in 2 patients, and EPT combined with balloon dilatation in 4 patients. Surgical treatment consisted of end-to-end anastomosis in 3 patients, hepatico-jejunostomy in 13 patients, and oversewing in 1 patient. Primary endoscopic intervention was successful in 36 patients, and 5 patients required surgical therapy. Surgical treatment was successful in 15 patients, 1 required furhter endoscopic treatment, and 1 patient died of peritonitis.
Conclusions: Endoscopic treatment is justified in negotiable stenoses and leaks. Complete transsection of the CBD or non-negotiable stenoses will remain the domain of surgery.

### F055

CARDIOPULMONARY EFFECTS IN LAPAROSCOPIC CHOLECYSTECTOMY - A COMPARATIVE STUDY

G.S. Zografos, Kalliopi Athanassiadi, G. Athanasas, G. Androulakis
4th Surgical Department of Athens University, General Hospital of Piraeus - HELLAS

The purpose of this prospective study is to investigate the effects of abdominal CO2 insufflation on the arterial gases (pH,pCO2,pO2,SAT), heart rate and arterial pressure during laparoscopic cholecystectomy.
The material of the study consisted of 10 patients (3 male, 7 female) with a median age of 54 (range 39-67)years, who underwent laparoscopic cholecystectomy (group A) and 10 patients (4 male,6 female) with a median age of 50 (range 38-65) years, who underwent open cholecystectomy (group B). All patients had uncomplicated cholelithiasis.
Preoperatively they were all investigated on routine tests, an ECG and a pulmonary function testing. During the operation, changes in heart rate, arterial pressure and abdominal pressure were recorded. Arterial blood samples were taken preoperatively and 30 min after the induction of anestesia in both groups.
According to our results, there were no statistically significant differences observed concerning the pH, pCO2, pO2 and SAT measurements. ($p>0.01$)
We thus conclude that laparoscopic cholecystectomy is a safe surgical technique without cardiopulmonary effects in uncomplicated cases.

### F056

INFLUENCE OF PNEUMOPERITONEUM AND REVERSE-TRENDELENBURG POSITION ON THE VENOUS RETURN DURING LAPAROSCOPIC CHOLECYSTECTOMY.

M.A. Reymond, Y. Christen, D. Tassile, C.E. Klopfenstein, H. Bounameaux, Ph. Morel.
University Hospital of Geneva, Switzerland.

Background: It is now well known that pneumoperitoneum induces a venous stasis in the lower limbs. Two questions with clinical relevance were therefore asked: i) does decreasing the pneumoperitoneum from 15 to 11 mmHg induce a significant improve of the venous return from the lower limbs ? ii) does the reverse-Trendelenburg position impair this venous return ?
Methods: In 12 consecutive patients the venous hemodynamic effects of pneumoperitoneum (0,11,13 and 15 mmHg) and reverse-Trendelenburg position were studied during laparoscopic cholecystectomy. Femoral venous diameter and peak femoral venous velocity were measured with Duplex-sonography. Femoral venous pressure was also monitored.
Results: i) Decreasing the pneumoperitoneum from 15 to 11 mmHg increases significantly the peak femoral venous velocity ($p<0.05$). It also diminishes the venous pressure and the femoral venous diameter. ii) The reverse-Trendelenburg position has no significant influence but on pressure ($p<0.01$).
Conclusions: The surgeon should use the lowest possible pneumoperitoneum during the operation, and not use a routine pressure. The reverse-Trendelenburg position does not significantly increase venous stasis in the lower limbs. The consequences of these hemodynamic variations on thromboembolic complications remain to be elucidated.

### F057

PATHOPHYSIOLOGICAL AND CLINICAL ASPECTS OF LAPAROSCOPIC CHOLECYSTEKTOMY IN HIGH-RISK CARDIAC PATIENTS(NYHAII-III) - A CLINICAL, PROSPECTIV STUDY

H. Gebhardt, H. Schaube, D. Loose*, H. Wulf*
Dept. Gen. Surg. and * Anaesth., University Kiel

Patients displaying cardio-pulmonary diseases should profit most from the benefits of laparoscopic surgery. However, the intraoperativ risk of the $CO_2$-Pneumoperitoneum (PP) is still under discussion. For further evaluation we investigated the pathophysiological changes of the $CO_2$-PP in high risk patients using an intensive monitoring. **METHODS:** 11 patients with clinical signs of cardio-pulmonary insufficiency (NYHA II-III) were monitored for cardiac output (CO), right-ventricle ejection fraction (REF), systemic-vascular resistance (SVR), transmural right-atrial-(TAP) and peripher-venous pressure (PVP). Furthermore blood-gas analyzes were performed. **RESULTS:**

| t(min.) | CO(l/min) | REF(%) | SVR(dyness./cm) | TAP(mmHg) |
|---|---|---|---|---|
| 0 | 3,7+/-0,4 | 44+/-4 | 1294+/-239 | 12+/-44 |
| 5 | 32,+/-0,4 | 47+/-9 | 2247+/-221 | 9,3+/-3,2 |
| 15 | 4+/-0,6 | 39+/-9 | 2415+/-272 | 4,5+/-2,1 |
| 60 | 4,3+/-0,6 | 36+/-5 | 2319+/-398 | 4,3+/-2,5 |
| After desufflation of the $CO_2$-Pneumoperitoneum | | | | |
| 10 | 5+/-0,7 | 38+/-7 | 1549+/-285 | 17+/-4 |

**DISCUSSION:** $CO_2$-Pneumoperitoneum causes a significant cardiac load by an increased cardiac afterload, a decreased preload and a reduction of CO. In one case the REF was predicting i.op. worsening demanding conversion to open cholecystektomy. The other patients did not demonstrate further cardiac complications. Despite this serious cardiac load laparoscopic surgery can be safely performed in high risk cardiac patients as long as a substantial perioperativ monitoring is performed.

### F058

LESSONS LEARNED FROM OUR FIRST 200 CONSECUTIVE LAPAROSCOPIC CHOLOCYSTECTOMIES

P. Tzardis, V. Laopodis, S. Durakis, N. Karavas, Emm. Kriticos, P. Klonaris, Emm. Tierris
1st Surg Dept, Red Cross Hosp. Athens

During a 20 month period (April 1993-November 1994), 200 patients underwent laparoscopic cholecystectomy (L.C.) at the 1st Surgical Department of the Red Cross Hospital in Athens. One hundred and eighty two patients (182) had cholelithiasis, 10 bile shudge and 8 gallbladder polyps. Standard laparoscopic procedure was followed in all cases. Pneumoperitoneum was induced via a Verress needle in all cases except in three patients where the Hasson method was used. Intraoperative cholangiography was performed selectively in five cases, and intraoperative ERCP in one. No deaths were noted. Six cases were converted to open cholecystectomy due to severe inflammation and adhesions (3 cases), haemorrhage (2 cases) and inferior mesenteric vein injury (1 case). Complications were as follows: common bile duct transection (one) retained common bile duct stones), pulmonary $CO_2$ embolus (one) undetected pancreatic cancer (one), subcutaneous emphysema (two), bile leakage (three), inferior epigastric vessels trocar injury (one), biloma formation (one), wound infection (five), persistent subdiaphragmatic pain (ten), gall-bladder contents spillage (eight).

Mean hospitalization was 2.8 days, while all patients resumed work within 5 to 10 days postoperatively. Cosmetic results were characterized as very good by 97% of patients. Postoperative pain was easily controlled with mild analgesics. Technical considerations concerning the procedure were a) difficulties with induction of pneumonoperitoneum through the Verress needle, b) systematic use of haemo-static absorbable gauge in the gallbladder bed, and subhepatic rediwach drain, c) extraction of the gallbladder through the upper middline hole and d) percutaneous drainage of the gallbladder in cases of hydrops or empyema.

It is concluded that extreme caution should be exercised during the "learning curve" period of laparoscopic cholecystectomy. Complete awareness of the pitfalls and dangers of the procedure is important in order to minimize complications and particularly injury to the CBD.

### F059

PULMONAR FUNCTION EVALUATION PRE- AND POSTOPERATIVELY AFTER LAPAROSCOPIC CHOLECYSTECTOMY AND SUBCOSTAL INCISION

E. Crema, J H Santana, E L Dair, A A Silva, L F V Mesquita

Disciplina de Cirurgia do Aparelho Digestivo da Faculdade de Medicina do Triângulo Mineiro

Brazil

Forty-four patients, presenting with symptomatic cholelithiasis, mean age 47.43 years (ranging from 18 to 82 years), being 37 female. All of them underwent pulmonar function evaluation (spirometry) pre- and postoperatively (1st, 2nd and 3rd postoperative days). Values obtained were compared to the elective procedure used: Group I: 25 patients, laparoscopic cholecystectomies and Group II: 19 patientes, high subcostal transverse incision.

No significant differences were observed among patients of 2 groups, neither between both groups when values expected for forced vital capacity (FVC), volume expirated in 1 second (FEV1) and forced expirated flow 25-75% (FEF) of FVC were compared with respective preoperative values.

For Group I, preoperative mean value of CVF (79.39) compared to values at 1st (62.50), 2nd (64.25) and 3rd (70.55) postoperative days; preoperative mean value of FEV1 (84.65) compared to values at 1st (69.75), 2nd (65.00) and 3rd (78.88) postoperative days, it was observed reduction with no statistically significant differences.

For Group II, preoperative mean values of CVF (78.22) compared to values at 1st (45.33), 2nd (51.25) and 3rd (68.33) preoperative mean value of FEV1 (84.38) compared to 1st (49.66), 2nd (55.00) and 3rd (69.66) postoperative days, it was observed reduction (statiscally significant).

Results indicate lower pulmonar disfunction at 1st, 2nd and 3rd postoperative days of Group I (laparoscopic cholecystectomy) than Group II (subcostal incision).

### F060

LAPAROSCOPIC CHOLECYSTECTOMY. FOUR YEARS EXPERIENCE AND RESULTS

### F061

### THE ROLE OF RETROGRADE APPROACH IN LAPAROSCOPIC CHOLECYSTECTOMY

**Palanivelu C. MS.MCH.**, Rajan P.S., Mahesh Kumar, *Department of Surgical Gastroenterology, Coimbatore Medical College Hospital, Coimbatore-641 018, INDIA.*

Though the antigrade approach is easier in Laparoscopic Cholecystectomy, there certain circumstances retrograde fundus on gallbladder laparoscopically.

Though June 1991 to October 1994, 1250 patients with gallstones were treated by Laparoscopic Cholecystectomy. Wherever liver could not be retracted cranically with adequate exposure, retrograde cholecystectomy was performed by fundus first on approach for the following indications : A. Liver plastered to the chest wall - 14, inflammatory in 12 and developmental in 4, B. Malpositioned gallbladder - 4, Medioposition in 3 and Left lobe GB in 1, C. Firm Cirrhotic liver - 8, and D. Normal liver, cystic duct joining the LHD deep in the hilum.

Group A - when Liver plastered to chest wall, duodenum and omentum were retracted down and retrograde cholecystectomy was performed. By giving downward traction on the fundus, gallbladder was easily seperated from the liver. Group B - Liver was lifted upwards by retracting the quadrate lobe using dipping retracted as described in the French technique. All the patients had uneventful post operative recovery.

Retrograde cholecystectomy has got definite indications for safe and effective Laparoscopic Management of gall stone desease.

### F062

### DIFFICULTIES AND BENEFITS OF THE LOPAROSCOPIC CHOLECYSTECTOMY

**A.E.NICOLAU**, D.Venter, R.Mehic, Gh.Ionescu
Department of Surgery, Emergency Hospital, Bucharest, Romania

Our study reports on the first 120 laparoscopic cholecystectomies(LC)performed in our Department of Surgery between December 1993 and November 1994.It tries to point out the advantages of a such a surgical approach despite the difficulties inherent to any beginning.
Difficulties were represented by:modest technical equipment,surgeons'team inadeqnate training ,patients reduced adressability,impossibility to perform ERCP,difficult selection of cases and patients anatomicopathological specific features:17,5% acute cholecystitis,5% hydrops, 13,3% sclero-atrophic cholecystitis.Acute cholecystitis approach has been extended during the last few months.
The benefits of LC are yet obvious.The mean period of hospitalization was three days.We had trhee major complications(parietal abscess,respectively pulmonary embolism).
The was no conversion to open surgery.
We consider LC to be the elective surgery in cholecystitis and in some cases of acute cholecystitis and it is in our intention to extend this procedure to a growing number of patients.

### F063

### LAPAROSCOPIC CHOLECYSTECTOMY - OUR TWO YEARS EXPERIENCE

K.Gyftopoulos, I.Palasconis, E.Zacharopoulou, Ch.Paranomou, S.Artelaris, P.Andricopoulos.
Departement of Surgery, Aegion General Hospital, Greece.

The aim of this study was to analyse the results of 292 laparoscopic cholecystectomies performed in our Hospital during the period of Aug 92-Dec 94 and to present the medical and socio-economic advantages of this method.

A total of 259 patients were symptomatic and the main symptom was pain in the right upper quadrant (67%).There were 213 women(72%) and 79 men (28%).The mean age of the patients was 50 yrs (16-86).Sixty-seven pts.had been previously operated in the abdomen,with appendiccectomy and hysterectomy being the most common operatons.Diagnosis of cholelithiasis was established by means of Ultrasound.A check-up of biochemical parameters was performed in all pts.

The procedure was successful in 279 cases (95%).Major operative complications occurred in 7 pts and included: bleeding(1,7%),cystic duct leak(0,34%).No common bile duct injury occurred.Accidental perforation of the gallblader occurred in 93 pts(32%).13 cases were transformed into open operation.In8 cases(2,7%)gallstones fell into the peritoneal cavity and were removed by differrent means.The patient with the cystic duct leak had to be re-operated (0,34%)The mortality was zero (0)with a very low morbidity (4%).The mean operative time was 40´(25´-90).The mean hospitalization time was 2,7 days (1-10 d)and most patients were able to return to work in 7-10 days.

Laparoscopic cholecystectomy is constantly proving its efficiency as a successful method of treating cholelithiasis by means of Minimal Invasive Surgery.The main advantages are minimal trauma,less pain,quick recovery,which are at the patients and the State´s benefit.

### F064

### Elective Cholecystectomy - a minimal invasive Approach for Diagnostic Procedures and Therapy

E. Schweizer, P. Boll, H. Schaube

In elective cholecystectomy ultrasound is the primary diagnostic procedure of choice to detect gallstones and biliary lithiasis, but still intraoperative controlled trial (January 1989-August 1991) we evaluated a diagnostic scheme of defined risk factors (patients history, laboratory findings, ultrasonographic results) in comparison to intraoperative cholangiography in 384 patients. Concerning the evaluation of cholecystolithiasis sensitivity of US was 96%, preoperative cholangiography 88%. Bile duct stones could be detected by US in 77%, in peroperative cholangiography in 99%. Sensitivity of intraoperative cholangiography was 100%, specificity 90%. The incidence of one of the above mentioned risk factors correlated significantly (p 0,01, Chi-Square-Test) with the incidence of choledocholithiasis. The negative predictive value of the risk factors was 100%. All patients with bile duct stones had been detected.
Since September 1991 the above mentioned diagnostic setting was used in 775 patients. 142 of the patients were risk factor positive and underwent preoperative i.v.-cholangiography and/or ERCP prior to laparoscopic cholecystectomy. In 21 patients bile duct stones were present and could be removed prior to laparoscopic cholecystectomy. Incidence of bile duct stones was negative in all patients postoperatively.
In case of one positive risk factor preoperative cholangiography or ERCP should be recommended to avoid intraoperative cholangiography which is no longer necessary.

### F065

MINI CHOLOCYSTECTOMY

p.Capsambelis, K.Tsangaropoulos, E.Palli.A.Karaghiannis.G.Kastanis, A.Papadatou,S.Stravolemos
DEPARTMENT OF SURGERY, GENERAL HOSPITAL OF ZANTE, GREECE

The purpose of our study is to present our data, the results and in general the surgical experience from the performance of mini-cholocystectomy to patients with litiasis in the gall blader.
In a three-year period 1991-1994, 24 patients (8M-16W) have been through mini-cholocystectomy. The results are satisfactory. The operating time had an average of 60 min, the post-operating nursing time was 48 to 72 hours with no complications. In 12 of our sample we penetrated the surgical trauma with local anesthetic, in such a case the post-operating pain was completely tolerable, whereas in the rest small doses of Propoxyphen or Pethidine were necessary. The aesthetic results were very good.
CONCLUSION: The mini-cholocystectomy allows ease at surgical operation, diminues the nursing time, minimises the post operation pain, especially after the penetration of local anesthetic and gives a satisfactory aesthetic result. Also it is a good and secure alternative to the laparoscopic cholocystectomy.-

### F066

**SURGICAL OPTIONS IN TRAUMATIC INJURY TO THE EXTRAXEPATIC BILIARY TRACT**

Xeropotamos N, Baltoyannis G, Koulouras B, Cassioumis D.

Surgical Department, School of Medicine, Ioannina University, Ioannina Greece

Traumatic injury of the extrahepatic biliary tract is very uncommon. Recommendations for optimal operative management is controversial because on such small numbers of cases in the reported series.
Patient and Methods: 5 patients who sustained extrahepatic biliary tract injury during a 16-year period (1978-1994) and treated at the Surgical Department, School of Medicine, Ioannina University, Ioannina Greece included in this series. These patients represent 1,5% of all the operated traumatic intrabdominal injuries in the same period. The median age was 49 years (range 21-62 years), three patients were male and two female. All the cases were due to blunt injuries from motor vehicle accidents.
Results: The diagnosis of traumatic intraabdominal injury was made by DPL. Upon arrival at the hospital all but one patient were in shock because of associated injuries. One patient had traumatic cholecystectomy, one traumatic hemobilia of the gallbladder, one had near complete transection of common bile duct and two complete transection of the left hepatic duct and common bile duct respectively. Recognition of one near complete transection of common bile duct was made after the onset of obstructive jaundice because of stricture formation on the common bile duct. Operative management included: two cholocystectomies, one end-to-end duct repair, one Roux-en-Y choledocho-jejunostomy, and one choledocho-duodenostomy. All patients recovered uneventfully including the patient with the late recognition of the bile duct injury. In the patient with end-to-end repair a stricture of the left hepatic duct developed 4 months after the operation.
Conclusions: All central retroperitoneal haematomas should obligatorily be explored for recognition of possible ductal injury. Complete transection of the bile ducts are best managed by primary duct-enteric anastomosis.

### F067

MODERN APPROACH OF THE BILIARY TRACT DISEASE (LITHIASIS. CHOLECYSTITIS.CHOLANGITIS) TWO (2) YEARS CLINICAL EXPERIENCE OF 3D SURGICAL DPT OF UNIVERSITY OF ATHENS.

C.Fotiadis, G.Papastratis, D.Mandrekas, Th.Liakakos, A.Machairas, E.Karageorgos, M.Sechas.
Third Surgical Dpt of University of Athens.

The aim of this study is to describe our experience on cholecystitis, cholangitis and lithiasis of the biliary tract from 2/93 to 12/94. In a total sum of 145 admissions for biliary tract disease (92 women 63.44% - 53 men 36.56%) 108 patients were operated and 37 were treated conservatively (3 of them with ERCP). As for the technique, we had an open method in 58 cases (53.71%) and laparoscopy in the other 50 (46.29%).
Our operative findings were:
a. 90 cases with lithiasis of the gallbladder
b. 7 cases with embyema of the gallbladder
c. 5 cases with hydrops
d. 4 cases with choledocholithiasis
e. 1 case with abscess and
f. 1 case with Ca (carcinoma)
The mean time of hospitalization postoperative was 6 (six) days for the open method versus two (2) days for laparoscopy. The choise of surgeons between the two methods is equal, except for the difference of the nursing time postoperative. The most frequent cause of the biliary tract disease is lithiasis of the gallbladder and this is frequent among women of the 5th.6th.7th decade.

### F068

CHOLEDOCHAL CYSTS IN ADULT LIFE, AN ANALYSIS OF OWN MATERIAL AND METHODS OF SURGICAL TREATMENT

M.Jesipowicz, S.Rudzki, J.Jesipowicz, M.Matuszek
The First Department of General Surgery
Medical Academy in Lublin, Poland

The intra- and extrahepatic cystic dilatations of biliary ducts belong to rare defects of the duct system. It is a typically surgical problem of childhood. However in nearly 20% of patients the diagnosis is delayed until adulthood. The purpose of this chapter is to define a coherent approach to the surgical management of these patients according to pathology problem.
From the practical point of view it seems to be proper to accept the classification proposed by Tadani.
In the First Department of General Surgery 16 patients with choledochal cysts were treated in the last twenty years.
In 14 patients the cysts were excised together with ducts. Those biliary tracts were anastomosed by a Roux an Y jejunal loop.
In 2 patients with cysts of the hepato-pancreatic ampula, which were 10 cm in diameter, through-duodenal excision of the cyst together with ampulla was done and the choledochus and pancreatic ducts were implanted in to posterio-medial duodenal wall.
The follow up study showed a good conditions of all patients according to isotopic scanning techniques which should be used to value eventual stricture of anastomosis.

### F069

### THE OPERATIVE TREATMENT OF CHOLEDOCHAL CYSTS IN CHILDREN

M. Soutis, G. Mavridis, E. Papandreou, G. Sakellaris, D. Keramidas
2nd Dept of Pediatric Surgery, "Aghia Sophia" Children's Hospital, Athens, Greece

Cystic dilatation of the extrahepatic bile ducts is a rare congenital anomaly which should be treated surgically because of its serious complications. In this study we present our experience with this malformation in regard to clinical presentation and diagnosis and especially to surgical treatment and postoperative results. Thirteen operations on choledochal cysts out of 152 operations on the extrahepatic biliary tree were performed during a nine year period. Ten patients were females and 3 males aged between 2 months and 14 years. Clinical onset was atypical with vague abdominal pain in 8 patients; cholangitis was the mode of clinical presentation in 5 patients. Preoperative diagnosis was made by abdominal ultrasonography in all patients. In all but one patients excision of the extrahepatic biliary tree up to the common hepatic duct was performed and the bile drainage was reestablished by constructing a Roux-en-Y anastomosis between the common hepatic duct and a jejunal loop. Radical excision of this type is necessary in order to eradicate the dysplastic tissue, thus eliminating the risk of carcinoma development. In one patient local excision of a spherical cyst arising from the division of the common hepatic duct was performed. There were no complications during a follow-up period of 1-8 years and the patients' growth is within normal limits. It is concluded that choledochal cyst is a rare congenital anomaly representing about 8.5% of the extrahepatic biliary tree pathology in children; it has a female to male predominance; it is easily diagnosed; surgical excision of the anomaly followed by hepatico-jejunostomy is feasible and complications free.

### F070

### CYSTIC DILATATIONS OF THE COMMON BILE DUCT IN ADULTS

Gr.Kouraklis,E.Misiakos,A.Glinavou,G.Karatzas,J.Gogas
2nd Department of Propedeutic Surgery,Athens University Medical School, Greece

Cystic dilatations of the common bile duct are believed to be of congenital etiology with most cases presenting in childhood. The aim of this study is to present our experience with cystic dilatations of the bile duct in adults. During the last 20 years,10 patients with cystic dilations of the bile duct were treated in our Department. There were 5 men and 5 women with an age range of 35-81 years Clinical presentation consisted of right hypochondrial pain, nausea, nomiting and a history of obstructive jaundice. Diagnosis was established by ultrasound, cholangiography and ERCP in most cases. According to the Todani classification system, 5 patients had type I cysts, 4 had type II and one had type III. At the time of surgery, main associated diseases were:choledocholithiasis in 3 cases and cholangitis in 2 cases. One patient (type III) underwent endoscopic sphincterotomy;5 patients underwent cholecystectomy with internal drainage and 2 of them developed recurrent cholangitis postoperatively; 4 patients underwent excision of the cyst and a biliary-enteric bypass and developed no main complications. Patients remained in good health during long-term follow up. Cyst excision is the treatment of choice for adults in order to reduce postoperative morbidity and the potential risk of malignancy.

### F071

### CHOLECYSTECTOMY IN 114 PATIENTS OVER 80 YEARS OLD

S. Kakkos, J. Harkoftakis, D. Karavias, C. Tepetes, C. Vagenas, J. Androulakis.
Department of Surgery, University Hospital of Rion, Patras, Greece.

The main purpose of this study was to estimate morbidity, mortality and possible contributing factors in cases of cholecystectomy performed in patients over 80 years old. We studied retrospectively 114 consecutive patients over 80 years old, who underwent cholesystectomy, in our department, during the period 1987-1993. There were 50 men and 64 women with mean age of 83.5 years (S.D. 3.4, range 80 - 97). Forty five patients (39.5 %) had acute cholecystitis and 41 (36%) had choledocholithiasis. An urgent operation was carried out in 43 patients(37.7%). The control group consisted of 84 consecutive patients below 6 years old, 23 men and 61 women, with mean age of 45.4 years (S.D. 10.9, range 20 - 61), who underwent cholesystectomy during 1991. We examined the influence of age, sex, jaundice, physical sings, acute cholecystitis, exploration of CBD or choledocholithiasis, urgency of operation and preoperative medical problems on postoperative hospitalisation and morbidity, using regression analysis.
Morbidity was 22 % and mortality 2.6 %, compared with 9.5 % and 0 % respectively in the control group(p=0.034, and p=0.19 respectively). Only age, postoperative complications and CBD exploration had statistically significant influence ($p< 0.05$) on postoperative hospitalisation. Age, CBD exploration and also jaundice and medical problems had statistically significantly influenced morbidity.
In conclusion, cholecystectomy performed in patients over 80 years old, is a relatively safe procedure, despite the higher complication rate, compared to patients younger than 61.

### F072

### CHOLELITHIASIS AFTER GASTRIC SURGERY

M.R.Diaconescu,I.Simon,I.Costea,M.Zamfir

Fourth Department of Surgery. University of Medicine and Pharmacy, Iaşi, Romania

BACKGROUND. It has been suggested that operations of the stomach may predispose to subsequent development of cholelithiasis (CL).
PATIENTS AND METHODS. In order to verify this hypothesis we reviewed our experience in 20 patients(13 males, 7 females of medium age 53) with chronic or acute CL(in 4 cases with common bile duct(CBD) stones and cholangitis) which underwent previous gastric surgery. They represent 1,6 % from all biliary tract operations and respectively 2,1% from all gastric operations in the last 10 years.
RESULTS AND DISCUSSIONS. .The gastric operations performed 5-22 years ago were Billroth I(2 cases) and II(10 cases) partial gastrectomy, truncal vagotomy with pyloroplasty or antrectomy for peptic ulcer disease, hemigastrectomy and tumorectomy for benign gastric tumours(2 cases), subtotal enlarged gastrectomy for gastric cancer (one case), and finally gastrectomy for suspected gastric tumour(one case).
Statistical and clinical data as well as the diagnosis and intraoperative findings are analysed.
All but one of our cases underwent emergency or elective cholecistectomy ± CBD exploration.
CONCLUSION. The causal connection between CL and previous gastric surgery are obvious but anatomical modifications and alterations in the duodenum and CBD also with decreased bile acid synthesis may be incriminated.

### F073

### SELECTIVE SURGICAL TREATMENT FOR IATROGENIC HEMOBILIA

**B. Dousset*, A.Sauvanet, M. Bardoux, V. Vilgrain, J Belghiti**
**Departments of Surgery, Hôpital Beaujon and Cochin*, Paris, France**

Iatrogenic hemobilia following percutaneous liver biopsy (PLB), percutaneous transhepatic cholangiography (PTC) or percutaneous biliary drainage (PBD) now represents the first cause of hemobilia. To assess the place for surgery, we report our experience of 17 patients treated for severe iatrogenic hemobilia.

**Methods.** Between 1989 and 1993, 12 males and 5 females, aged 21 to 70 years were referred for hemobilia after PLB (n=10), PTC (n=4) or PBD (n=3). All patients required blood transfusion, ranging from 2 to 6 red cell units. Indications for PTC were hilar cholangiocarcinoma (n=1), and common bile duct stones (n=3) two of which following remote cholecystectomy. Indications for PBD consisted of pancreatic carcinoma (n=2) and hepatocellular carcinoma (n=1).

**Results.** *Hemobilia after PLB.* Selective angiography demonstrated an arterio-portal fistula in 4 patients, an arterio-biliary fistula in 2, a false aneurysm in 2, a vascular flask in 1 and was normal in 1 patient. Selective embolization was attempted in 9 patients and was successful in 7 cases (78%), 4 of whom required secondary cholecystectomy for hemocholecystitis (n=3) or ischemic cholecystitis (n=1). The 2 patients in whom selective catheterization proved impossible were treated by ligation of the right hepatic artery. The patient without recognizable vascular lesion was treated by cholecystectomy and retrograde irrigation of the biliary tree without artery ligation.. *Hemobilia after PTC.* All patients had dilated intrahepatic ducts prior to PTC. Selective angiogram revealed a vascular lesion which was successfully embolized in the 4 patients. Biliary decompression was secondarily achieved by endoscopic sphincterotomy + nasobiliary catheter (n=2), percutaneous transtumoral intubation + external biliary catheter (n=1), and surgical extraction of common duct stones + T-tube (n=1) because of associated hemocholecystitis. *Hemobilia after PBD.* Anterograde irrigation of the biliary tree in addition to reintroduction of a larger catheter 2 instances achieved to stop the bleeding and to decompress the bile ducts above the site of tumor obstruction. Both embolization and surgery were avoided in the 3 patients. None of the 17 patients died nor experienced recurrent hemobilia following treatment.

**Conclusion.** 1) Successful management of iatrogenic hemobilia is based upon arterial embolization of the intrahepatic bleeding site and biliary decompression in case of obstructive jaundice. 2) Indications for surgery are limited and include failure or complication of arterial embolization, acute hemocholecystitis and failed attempt at endoscopic or percutaneous biliary decompression.

### F074

### EXTERIORIZATION OF THE BLIND INTESTINAL END OF ROUX-Y BILIOJEJUNAL ANASTOMOSIS FOR FUTUR ENDOSCOPIC ACCESS TO THE ANASTOMOSIS

P. Giannopoulos, D. Babalis, A. Polydorou, A. Vezakis, C. Charitopoulos, S. Smirnis
Department of Surgery, Hippocration Hospital, Athens, Greece

The usuall restoration of biliary tract injuries with a Roux-Y bilioenteric anastomosis carries a risk of secondary stenosis or lithiasis above the stenosis. Up to recently these patients were managed operatively or by a percutaneous approach. Today, stabilization of the blind jejunum in the subcutaneous layer during initial operation allows exteriorization when neccessary, permitting endoscopic access to the anastomosis.
We applied this method in 3 patients who had undergone multiple biliary procedures due to iatrogenic trauma during open cholecystectomy and had developed intrahepatic lithiasis.
This method succeded in clearing all stones and patients are asymptomatic 19, 7 and 3 months respectively later. At the end the jejunum was closed and reburied subcutaneously for futur endoscopic access.
In conclusion we propose exteriorization of the Roux loop as a routine method for the Roux-Y reconstruction of benign biliary stenosis so that difficult and dangerous reoperations will be avoided.

### F075

### THERAPEUTIC SPLITTING FOR CHOLEDOCHOLITHIASIS

M. Gundlach, C.C.J. Pohland, A. Emmermann, C. Zornig, N. Sohendra, C.E. Brölsch
Dept. of Surgery, Univ. Hospital Eppendorf, University of Hamburg, Germany

**Introduction:** The management of common bile duct (CBD) stones has been debated for years. In a retrospective study the impact of preoperative, selective endoscopic cholangiography (ERC/P) and therapy was evaluated in 577 consecutive patients with symptomatic gallstones. **Patients and Results:** Because of clinical presentation, pathologic laboratory or a presumed pathology on ultrasound 128 patients were suggested to have a CBD stone and had preoperative ERC/P. 68 patients were found to have choledocholithiasis (11.8%). 19 ERC/P's were performed after cholecystectomy and stones were detected in 9 (1.6%), with a median follow-up time of 30 months (range 12-82 months). Clearance of the CBD was achieved with or without sphincterotomy in all patients. Morbidity was 2.0% after ERC/EPT and mortality was zero. Local and general complications were not increased neither intraoperatively nor postoperatively. **Conclusion:** Cholecystectomy can be performed safely without routine intraoperative cholangiography by means of the "therapeutical splitting". Conversion to open or laparoscopic bile duct surgery can be avoided. The selective use of preoperative ERC/P will clear the CBD of stones in 93% of patients. In symptomatic patients after cholecystectomy a clearance of the CBD was achieved in all cases with ERC/P. This approach facilitates a very low morbidity, mortality and high patient comfort in the treatment of the complicated gallstone disease, especially in regard to the long-term complications observed after surgical common bile duct exploration.

### F076

### TRANSDUODENAL SPHINCTEROPLASTY-STILL HAS A PLACE

Wig JD, Gupta NM, Behera A, Bose SM, Katariya RN, Khanna SK, Department of Surgery, Postgraduate Institute of Medical Education and Research, Chandigarh-160012, INDIA.

Frequently employed biliary drainage procedures for common bile duct calculi include choledochoduodenostomy and transduodenal sphincteroplasty (TDS). This is a retrospective analysis of 120 patients submitted to TDS - 31 male and 89 females, and their ages ranged from 21-78 years. The decision to perform TDS was taken during the operation in majority of cases. Inability to pass a No.3 dilator through the papilla was an indicator for TDS. The standard operative procedure was supraduodenal choledochal exploration with TDS after performing a short vertical duodenotomy over the papilla and leaving a T-tube. The patients presented with jaundice (38%), cholangitis (10%). There was previous history of jaundice and or fever in 20%. The indications for TDS were primary CBD stones (1.7%), multiple stones (40%), impacted ampullary stones (21%), ampullary stenosis (25%), residual stones/recurrent stones (12.5%). Postoperative complications included wound infection (10%), cholangitis (6.7%), residual stones (3%), hyperamylasaemia (4%). No further intervention was needed for residual stones. Two patients died (1.7%) - one from GI bleed and one from pancreatitis. In the follow-up period ranging from 2-8 years, no serious long-term complications have been encountered. This retrospective study shows that TDS is a safe and effective drainage procedure in the management of calculous biliary disease.

### F077

**COMBINED TREATMENT OF BILIAIRY LITHIASIS BY PREOPERATIVE ENDOSCOPIC RETROGRADE CHOLANGIOPANCREATOGRAPHY AND SUBSEQUENT CHOLECYSTECTOMY.**

*C.H.Huynh, A.Medhi, J.Van de Stadt, M.Cremer, M.Gelin.* Department of Digestive Surgery and Gastroenterology, Erasme hospital, Brussels, Belgium.

Between January 1992 and December 1993, 452 cholecystectomies were perfomed in our Institution. There were 321 females (71%) and 131 males (29%). Mean age was 55 years (range 23 - 91).
Two hundred ninety three patients (65%) presented with a chronic symptomatic cholelithiasis (group I) and 159 patients (35%) had a complicated cholelithiasis (118 cholecystitis, 18 pancreatitis, 19 cholangitis, 4 peritonitis) (group II). The common bile duct was investigated by ***endoscopic retrograde cholangiopancreatography (ERCP)*** each time a common bile duct stone was suspected (group I: 33%), and in acute situation (group II: 66%). Endoscopic sphincterotomy and lithiasis extraction from the main biliairy duct (MBD) were performed respectively in 33 patients (11%) and 14 patients (4.8%) in group I, and in 77 patients (48%) and 28 patients (17.6%) in group II. No morbidity was related to ERCP in this series.
***Laparoscopic cholecystectomy*** has been successfully performed in 334 patients (74%): the morbidity was 4.5% and mean hospital stay 3 days. ***Conversion to laparotomy*** was neces²sary in 33 patients (7%): the morbidity (12%) and mean hospital stay (8.7 days) were significantly higher. There was no difference between the two groups according to sex, previous surgery or indications.
***Cholecystectomy by laparotomy*** was performed as a first choice procedure in 85 patients (19%): the morbidity was 15% and mean hospital stay 9.8 days. Due to preoperative ERCP, peroperative lithiasis extraction from the MBD was necessary in only 1 case.
*Conclusion:* Preoperative ERCP significantly decreases peroperative extraction of lithiasis from the MBP during subsequent cholecystectomy. When a lithiasis in the MBD is suspected, the association of preoperative ERCP and laparoscopic cholecystectomy allows a shorter hospital stay with a lower morbidity rate.

### F078

**MANAGEMENT OF COMMON BILE DUCT STONES IN PATIENTS OVER THE AGE OF 75**

M. Suter , O. Martinet, H. Vuilleumier, M. Gillet
Department of Surgery, Centre Hospitalier Universitaire Vaudois
1011 Lausanne, Switzerland

The introduction of endoscopic sphincterotomy (ES) in 1974, and more recently the advent of laparoscopic techniques, have put into question the entire therapeutic concept for gallstone disease. Elective cholecystectomy is no longer the rule for asymptomatic cholelithiasis, especially in the elderly, and some authors have questioned the paradigm of routine cholecystectomy in the presence of common duct and gallbladder stones. For common duct stones (CDS), there is a strong shift from classic open choledocotomy to ES. The aim of this study was to evaluate the results of traditional common duct surgery in the elderly patients, in order to define an optimal therapeutic approach in this group.

Among the 656 patients who had common duct surgery in our department between 1976 and 1993, we reviewed the charts of 188 patients over the age of 75. Seventy-five patients (40 %) were operated on for acute cholecystitis. Seventy-nine (42 %) were jaundiced, 39 (21 %) had acute pancreatitis, and 45 (24 %) had angiocholitis. In 79 cases, there was no preoperative reason to suspect CDS. There were 107 emergency, and 71 elective operations. Hundred and twenty-three (66 %) patients had choledocotomy and T-drainage, 8 (4 %) had open transduodenal sphincterotomy, and 57 (30 %) had a bilio-enteric anastomosis. The overall mortality rate was 8,5 % (16 cases), and the morbidity reached 43 % (13 % major complications). Between the first and the second half of the study time, mortality fell from 14,8 % (14/95) to 2,1 % (p = 0,001). Severe associated disease (p = 0,02) and operative time over 150 minutes (p = 0,02) were significantly related to mortality.

In the elderly, common duct surgery is associated with substantial risks. In the absence of complication related to the gallbladder, CDS should be extracted by ES. Open surgery is reserved for cases in which ES fails to provide complete clearance of the common duct. Elective cholecystectomy (laparoscopic) can be performed secondarily if indicated. If cholecystectomy is required (i.e acute cholecystitis), open choledocotomy based on intraoperative cholangiography is justified, provided the patient has no severe associated disease and the operative time is not exceedingly prolonged. If clearance cannot be obtained promptly, a choledocoduodenal anastomosis or the placement of a T-drain followed by ES are safe alternatives. Ongoing studies will tell us which role laparoscopic common duct surgery has to play in this setting.

### F079

**COMMON BILE DUCT LITHIASIS :
SURGICAL TREATMENT OR ENDOSCOPIC SPHINCTEROTOMY ?
A randomized, controlled multicenter trial**

B. SUC, J. MICHEL*, Ch. PEILLON**, N. GONZALEZ*, G. FOURTANIER,* J. ESCAT*

The aim of this randomized prospective study was to compare surgical treatment (ST) with endoscopic sphincterotomy (ES) in the management of common bile duct lithiasis. Judgement criteria were decided to be firstly the post operative mortality and secondly retain stone rate, early or urgent second surgical procedure and mean hospital stay.
During five years, 28 centres included 220 patients in whom :
1) diagnosis of common bile lithiasis was considered as established,
2) active treatment was decided,
3) choice between surgical and endoscopic treatment was permissible.
Statistical analysis was by means of Chi 2 test, exact Fisher's test and ANOVA using StatView 4 software.
14 patients were excluded of the study and 206 records were analysed : 109 patients underwent ST and 97 ES. The two groups of patients were comparable (no statistical significant difference) concerning age, sex ratio, ASA score and indication for treatment. In the ST group, 74,5 % of patients underwent an operative cholangiography which found stones in 53 8 % and no stone in 20,8 % ; choledocotomy was performed and positive in 67 % of patients, performed and negative in 10,1 % of patients and not performed in 22,9 % of patients. Intra operative endoscopy was achieved in 52,8 % of patients. At the end of the procedure, an external biliary drainage was done in 61,1 % of patients, a biliary enteric anastomosis in 15,7 % , simple closure of choledocotomy in 10,2 %, a cholecystectomy only in 12,1 % and a surgical sphincterotomy in 0,9 %. In the ES group, papilla was always seen, retrograde catheterism was achieved in 95 % of patients and sphincterotomy in 82 %.
The difference of post operative mortality rate observed between the two groups was not statistically significant (0,9 % in the ST group, 3,1 % in the ES group). The difference of retain stone rate (8,3 % in the ST group, 21,6 % in the ES group) and of early or urgent second surgical procedure (1,8 % in the ST group, 18,6 % in the ES group) were statistically significant. The difference of the mean hospital stay (17,5 days in the ST group, 15,3 days in the ES group) was not statistically significant.

*Service de Chirurgie Digestive, Hôpital Rangueil, 31054 - TOULOUSE Cedex
**Service de Chirurgie Digestive, C.H.U. Ch. Nicolle, 76031 - ROUEN Cedex

### F080

**CHOLEDOCHODUODENOSTOMY AND SPHİNCTEROPLASTY İN THE TREATMENT OF CHOLEDOCHOLİTHİASİS**

N. Akyürek, E. Sözüer, O. Uslu, Y. Antaş, Y. Yeşilkaya

Univercity of Erciyes, Medical School,

Turkey

Gallbladder and bile duct diseases forms one of the groups of the most common diseases. There is a possibility of choledoc primary and seconder, stone to be found 12% of the subjects who were exposed to cholecystectomy. In the last fourteen years in our clinic, 156 patients were exposed to choledochoduodenostomy and 97 subjects to sphincteroplasty were examined retrospectively. Sixteen subjects who were bound to the T-tube drenage were primarly left out of the research. Eighty two (58.5%) subjects were women and fifty eight (41.5%) were men whose ages ranged between 18 to 85 at an approximate rate of 54.6. The order of symptoms seen in both groups were abdominal pain, nausea, vomiting, jaundice, sepsis and a fever mainly. In the choledochoduodenostomy subjects the approximate diameter of the choledoc was found as 23.5 milimeter, in the sphincteroplasty subjects 19.05 milimeter. The general morbidity rate was 20.9%, the mortality rate in the choledochoduodenostomy subjects was found as 4.6%, and in the sphincteroplasty subjects it was seen as 7.2%. The patients of choledochoduodenostomy stay in hospital for 9.3 days and the patients of sphincteroplasty were kept for about 10.1 days. Apart from the impact stone and stenosis of ampulla in the other explorations of the choledoc. There is not different between the choledochoduodenostomy and sphincteroplasty methods. But in the old aged risk group choledochoduodenostomy is easy, can be performed in a short time and is a trustworthy method.

**F081**

SURGICAL BYPASS FOR BENING OBSTRUCTIVE JAUNDICE. BENEFITS AND LIMITATIONS

D.Xypolytas, S. Nicolaides, P. Vrachnos, L. Papastamatiou
2nd Dept. of Surgery "Apostle Paul" Hosp.-KAT.ATHENS-HELLAS

Since 1888 when Riedel discribed choledochoduodenostomy as a bilio-digestive bypass (BDB) a wide range of bypass techniques for benign obstructive jaundice (BOJ) such as multiple choledocholithiasis, bile duct stricture, Oddi's stenosis, intrahepatic rupture of hydatid cyst (IRHC) etc. consisted the operative procedure of choice. Modern endoscopic or radiological interventional methods (E-RIM) restricted the operative indications but after the initial enthousiasm, clear limitations for each technique, as definite treatment are going to be established.

In the last 10-y period 122 patients with BOJ were treated. Prior cholecystectomy for lithiasis: 99 cases. Causes of BOJ: IRHC: 6 cases. Bile duct stricture: 4 cases. Oddi's stenosis: 9 cases. Lithiasis: 103 cases. Fourteen out of the last 103 patients underwent cholecystectomy and BDB. Another 10 cholecystomized patients with bile duct stricture or Oddi's stenosis, in wich E-RIM failed to solve their problem permanently, underwent also BDB, as well as the IRHC patients. Repeated or not endoscopy in 32, mostly elderly, patients with choledochal sludge or calculi were successfuly treated by endoscopy. In all other 71 cases a BDB was performed. Four elderly patients died by causes unrelated to their operative problem. Complication rate was rather high (10.6%)

It is concluded that a suitable and correct BDB achieves longterm efficiency for retained or reccurent calculi, while IRHC have to be reserved for elderly high risk patients, unilithiasic or with strictures-stenosis.

**F082**

**NITRIC OXIDE SYNTHASE ACTIVITY IN THE LIVER FOLLOWING INDUCTION OF GRAFT-VERSUS-HOST DISEASE AFTER COMBINED SMALL BOWEL AND BONE MARROW TRANSPLANTATION**

**F. Fändrich[1], J. Peters[2]**

**[1]Dept. of General & Thoracic Surgery, and**

**[2] Institute of Pathology, Kiel University, Kiel, Germany**

**INTRODCUTION:** It has been demonstrated that the liver plays a major role as a target organ in the course of bone marrow (BMTx) induced graft-versus-host disease (GVHD) and, to a smaller extent, after small bowel transplantation (SBTx). The immunological reactions involved are particularly characterized by the release of TNF-α and γ-interferon. Nitric oxide synthase (NOS) activation was shown to be elevated in response to such inflammatory stimulation. This study evaluated the expression of three NO isoenzymes, in particular brain (B), endothelial capillary (EC), and macrophage (Mac) NOS, and NADPH-diaphorase activity in the liver of recipient animals after simultaneous SBTx and donor-specific bone marrow infusion.

**METHODS:** A GvH-model was established by transplantation of DA or LEW parental cells/organs on F1-hybrids, a cross-breeding between DA(*RT1.*$^{avl}$) and LEW(*RT1.*$^{l}$) inbred rats: 1.DA(BM $5x10^7$cells+SB)-->F1, 2.DA(SB)-->F1,3.LEW(SB)-->F1,4.F1(SB)-->F1, n=6 animals/group. On day 7 and 14, APAAP-immunostaining with monoclonal antibodies specific for B-/EC-and Mac NOS and determination of the histochemical activation of NADPH-diaphorase was performed in adjacent liver tissues by microscopic evaluation.

**RESULTS:** There was a significant correlation between the manifestation of GvHD, periportal lymphocyte infiltration, and the increase of EC -and Mac -NOS and NADPH-d expression in livers of recipient animals as compared to syngeneic animals. B-NOS was unaffected. Simultaneous BM and SBTx increased the severity of GvHD and the NOS-activity.

**CONCLUSIONS:** NOS is proving to be an important mediator of homeostatic physiological processes and appears to contribute efficiently to the GvH related cytotoxicity induced in the host liver. Concepts to induce microchimerism by concomitant donor-specific BMTx may be hampered by the non-specific induction of NOS related cytotoxicity.

**F083**

INTRAOPERATIVE ASSESSMENT OF LIVER PERFUSION USING LASER DOPPLER FLOWMETRY (LDF) IN THE PORCINE MODEL

M. Cutress, A. Seifalian, L. Horgan, D. Moore, B. Davidson
Department Of Hepatobiliary Surgery & Liver Transplantation, Royal Free Hospital & School Of Medicine, London, U.K.

The hepatic microcirculation derives blood from the hepatic artery and portal vein which mixes at the level of the sinusoid. The objectives of the investigation were to: (1) determine surface and deep microvascular flow distribution; (2) assess liver perfusion during occlusion of: a) the splenic artery (SA), b) hepatic artery (HA) and c) portal vein (PV); (3) relate changes in liver microvascular flow to changes in total liver blood flow; (4) determine that liver microvascular flow is homogeneously distributed.

Laser Doppler flowmetry (LDF) was used to measure microvascular flow using surface and deep-needle probes. Electromagnetic flowmetry (EMF) applied on PV and HA measured total liver blood flow. We used 6 large white/landrace male pigs, weight 32±5kg (mean±SD) in our study.

There was a 31% distribution of microvascular flow on the surface layer and a 45% distribution deep within the liver, as measured at random sites by LDF. The effects of vascular occlusions are shown:

| Vascular Occlusion | Change in Total Liver Blood Flow (EMF) | Change in Liver Microvascular Flow (LDF) |
|---|---|---|
| SA | rise 1±0%, $p<0.01$ | rise* 10±9%, $p<0.01$ |
| HA | fall 13±8%, $p<0.02$ | fall 17±11%, $p<0.001$ |
| PV | fall 88±7%, $p<0.001$ | fall 48±20%, $p<0.001$ |

*significant in 5 out of 6 subjects.

In conclusion: (1) There exists a large variation in LDF recordings due to the distribution of microvascular flow and its continuous change. (2) LDF recordings indicate an increase in microvascular flow with occlusion of the SA, and larger decreases in microvascular flow with occlusions of the HA and PV. (3) These changes in microvascular flow reflect changes in total liver blood flow. (4) There were no significant differences in changes in surface LDF recordings compared with deep LDF recordings during vascular occlusions suggesting homogeneity of microvascular flow throughout the liver parenchyma.

**F084**

**PROLONGATION OF HAMSTER-TO-RAT LIVER XENOGRAFT SURVIVAL BY PRETRANSPLANT BLOOD TRANSFUSION COMBINED WITH FK 506.**

**B Nardo°*, LA Valdivia*, A Recordare°, AJ Demetris^, JJ Fung *, A Cavallari°, G Gozzetti°, TE Starzl***
**Department of Transplant Surgery* and Pathology^, University of Pittsburgh Medical Center, PA 15213, USA; 2nd Department of Surgery°, University of Bologna, Italy.**

It is known that donor-specific transfusion enhances graft survival in clinical and experimental organ allotransplantation. However, experimental trials using xenogeneic donor-specific transfusion, in order to improve xenograft survival, have resulted in sensitization of the recipients, with subsequent hyperacute rejection even in the presence of conventional immunosuppression. In this study we tested the effect of donor-specific transfusion combined with FK 506 on survival of hamster-to-rat liver xenografts. Golden syrian hamsters were the donors and Lewis rats the recipients of orthotopic liver transplants. Donor-specific transfusion in the form of 1 ml of hamster whole blood was given i.v. on day -7 to day -1. Liver grafting was done on day 0. Donor-specific transfusion alone induced high levels of anti-hamster antibodies on day 0, followed by hyperacute rejection. In the FK 506 group alone, increased survival to a mean of 26 days was observed. Finally, the combined treatment of donor-specific transfusion plus FK 506, further prolonged survival to a mean of 42 days. Hamster lymphocytes were detected on day 0 in the spleen of Lewis rats treated with donor-specific transfusion plus FK 506 but not in the spleen of rats treated with donor-specific transfusion alone. In conclusion, the combination of donor-specific transfusion plus FK 506 avoided the hyperacute rejection caused by donor-specific transfusion alone and resulted in a trend to longer liver xenograft survival. The presence of chimerism at the time of transplantation may be an advatage for longer graft survival. Becouse FK 506 is a T cell direct agent, this suggests that, xenogeneic sensitization, that lead to hyperacute rejection, requires T cell help. As in allotransplantation, donor-specific transfusion may be an advantage for clinical xenotransplantation of the liver provided that FK 506 is given.

### F085

**PERI-OPERATIVE RENAL TUBULAR DYSFUNCTION IN PATIENTS WITH OBSTRUCTIVE JAUNDICE AS INDICATED BY URINARY ENZYMOLOGICAL STUDIES**

D.N.Anderson+, C.P.Driver+, I.J.Broom* and P.H.Whiting*.
Departments of Surgery+ and Biochemistry* Aberdeen Royal Hospital's NHS Trust, Aberdeen, Scotland

Surgical intervention in obstructive jaundice has a significant post-operative mortality (13-37%), predominately attributed to the development of acute renal impairment ( 6-50%) which carries a mortality of 25-80%. The true incidence of proximal tubular failure may be underestimated or undiagnosed by conventional serum creatinine measurements.

The peri-operative urinary activities of the proximal renal tubular brush border enzymes gamma-glutamyl transferase (GGT) and alanine aminopeptidase (AAP) ,and the intracellular lysosomal hydrolase N-acetyl-B-D-glucosaminidase (NAG) were measured in 24 non-jaundiced patients having elective cholecystectomy (group 1). 31 jaundiced patients (group 2) and 19 patients undergoing surgery for relief of their obstructive jaundice (group 3).

Surgical intervention in group 1 had little effect on the urinary excretion of NAG, GGT and AAP ( 24±16 vs 30±28; 3.8±1.3 vs 4.1±3.8 and 1.1±0.7 vs 1.3±1.2 U/mmol creatinine respectively), yet in the jaundiced patients (group 2) both pre- and, in group 3, post-operative levels were significantly elevated (NAG 236±153 vs 328±182 $p<0.01$, GGT 6.6±2.5 vs 11.5±5.0 $p< 0.05$ and AAP 5.8±4.1 vs 10.1±6.1, $p< 0.01$). There was no correlation between the duration or degree of hyperbilirubinaemia and the detected enzymuria. Serum creatinine remained static following surgery in all groups.

Urinary enzymology highlights a proximal tubular dysfunction in patients with obstructive jaundice, compounded by surgical intervention. The measurement of serum creatinine lacks this sensitivity and is of limited value.

### F086

**ROLE OF NITRIC OXIDE IN ACUTE LIVER INJURY AND ASSOCIATED BACTERIAL TRANSLOCATION**

D. Adawi, F.B. Kasrawi, G. Molin, B. Jeppsson
Department of Surgery and Department of Food technology, Lund University, Sweden

Nitric oxide has a protective effect of the liver during endotoxemia and chronic inflammation. Its involvement in acute toxic liver injury is unknown. We therefore studied the effect of nitric oxide in D-galactosamine induced acute liver injury.

Liver injury was induced by intraperitoneal administration of D-galactosamine (1.1 gm/kg body wt.) Sprague-Dawley rats were used and divided into 4 groups: normal control, acute liver injury, acute liver injury + N-nitro-L-arginine methyl ester (L-NAME) and acute liver injury + L-NAME + L-arginine. Each group was studied at 3 time points 6, 12, 24 h after induction of liver injury. 6 h after liver injury Alkaline Phosphatases (ALP), bilirubin (bil), Aspartate Aminotransferase (ASAT) and Alanine Aminotransferase (ALAT) increased in the acute liver injury + L-NAME group compared to the acute liver injury control group (ALP 11±0.5 vs 8.6±0.6 $p<0.01$; bil 11.7±1.5 vs 7.1±1.3 $p<0.05$; ASAT 13.4±1.5 vs 8.6±1.1 $p<0.05$). The acute liver injury + L-NAME + L-arginine group showed reduced levels of ALP, bil, ASAT, ALAT compared to acute liver injury + L-NAME group (ALP 8.9±0.7 vs 11±0.5 $p<0.05$). 12 h after induction of liver injuries ALP , bil, ASAT, ALAT increased in the acute liver injury + L-NAME group compared to acute liver injury control group and decreased in the acute liver injury + L-NAME + L-arginine group compared to acute liver injury + L-NAME group with no significant difference. 24 h after liver injury, there was a significant increase in bil (24.83±4.79 vs 14.66±2.48 $p<0.05$) in the acute liver injury + L-NAME group compared to acute liver injury control group. The acute liver injury + L-NAME + L-arginine showed significant reduction in ALP (14.78±0.92 vs 18.9±1.99 $p<0.05$) compared to acute liver injury + L-NAME group. Bacterial translocation to arterial blood, portal blood, liver tissue and mesenteric lymph nodes increased at all time points in acute liver injury + L-NAME group compared to acute liver injury group with a significant difference in arterial blood at 24 h (148.3±70.4 CFU/ml vs 50±20.9 CFU/ml $p<0.05$) and decreased numbers in acute liver injury + L-NAME + L-arginine groups compared to acute liver injury + L-NAME groups (10±3.7 CFU/ml vs 148.3±70.4 CFU/ml $p<0.05$).

These results suggest a protective role of nitric oxide in acute liver injury induced by D-galactosamine and a reduction in bacterial translocation.

### F087

**THE EFFECT OF ANTI-CD4 MONOCLONAL ANTIBODY (MAb) AND CYCLOPHOSPHAMIDE ON THE SURVIVAL OF XENOGRAFTS OF FETAL PIG PANCREAS IN NON-OBESE DIABETIC (NOD) MICE.**

M. Koulmanda and T.E. Mandel.
Transplantation Unit, The Walter and Eliza Hall Institute of Medical Research, Melbourne, Australia.

Xenografts of islets may solve the severe shortage of donor tissue for transplantation in insulin dependent diabetes mellitus, but safe and effective immunosuppression to prevent rejection and recurrent disease in the graft is be required. We have shown that depletion of $CD4^+$ T cells in adult pre-diabetic female NOD/Lt mice with an anti-CD4 MAb (GK1.5) used peri-transplant (p/t) and weekly, prolongs survival of fetal pig pancreas (FPP) grafts up to 10wks. In this experiment we tested the effect of p/t and weekly immunosuppression with GK1.5 and Cyclophosphamide (CP) on graft survival.

Three groups each of 10 mice were grafted bilaterally under the renal capsule and 5 grafts per group were removed at 5, 8, 12 and 16 wks. Gp 1 received MTPBS; gp 2, 100mg/kg CP on day 0 and weekly; and gp 3 GK1.5 p/t and weekly plus CP on day 0 and weekly. The graft sites were fixed in Bouin's fluid, processed for light microscopy, and assessed for graft survival and extent and nature of infiltration. Peripheral blood was monitored by flow cytometry (FC) biweekly, and on wks 8 and 16 LN and spleen were also assayed for T cell subsets ($CD4^+$, $CD8^+$, $CD3^+$, $Thy1.2^+$) and for B cells ($sIg^+$).

Five wks post-transplant all grafts were rejected in gp 1, 2/5 grafts were present but heavily infiltrated in gp 2, but all grafts were present in gp 3. By wk 12 gp 3 grafts were still present without infiltrate and by wk 16, 50% were still present without infiltration. However due to CP toxicity we lost 50% of the animals making the CP treated gps very small. FC showed that B cells in gps 2 and 3 were reduced by 50% and CD4+ cells in gp 3 were reduced to 1% (normal 40%); both B and $CD4^+$ T cells remained depleted to the end of the experiment.

We conclude that CP treatment delays rejection minimally but CP plus anti-CD4 therapy can prevent rejection of FPP grafts in NOD mice for at least 16 wks.

### F088

**A FUNCTIONAL THROMBIN RECEPTOR IS CO-LOCALISED WITH FACTOR Xa RECEPTOR AND TISSUE FACTOR IN HUMAN PANCREATIC CARCINOMA CELLS.**

**A.K. Kakkar, V. Chinswangwatanakul, R.C.N. Williamson.**
**Royal Postgraduate Medical School, London.**

**Human pancreatic carcinoma is characterised by its aggressive nature and high incidence of thrombotic complications. Pancreatic carcinoma cells express cell surface tissue factor (TF), which in turn leads to factor Xa and thrombin generation. We have studied the expression of human thrombin receptor (HTR), factor Xa receptor (EPR-1) and tissue factor (TF) in 8 human pancreatic carcinoma cell lines, using rabbit anti-HTR and anti-TF polyclonal antibodies, and a mouse anti-EPR1 monoclonal antibody. All 8 cell lines showed clear membrane immunoreactivity for TF and EPR-1, with different degrees of expression, while HTR staining was localised to a distinct cytoplasmic region in each case. Localisation of all three antigens was confirmed with confocal microscopy. Stimulation of 2 cell lines (BXPC3 and PT45) with human thrombin increased calcium ion influx, as manifested by flourescent spectrometry. Incubation of the same two cell lines with human thrombin (2 units/ml for 6 hours) led to an increased secretion of urokinase plasminogen activator (UPA) from 5.15 to 10.1 ng/ml (BXPC3) and from 2.35 to 8.1 ng/ml (PT45). TF antigen secretion was unchanged. Specific cell surface receptor binding was confirmed using $^{125}I$-thrombin. This study shows the presence of a functional thrombin receptor co-localised with factor Xa receptor and TF in pancreatic carcinoma and suggests a direct link between the coagulation and fibrinolytic systems in cancer biology.**

### F089

**REVERSAL OF OBSTRUCTIVE JAUNDICE : EFFECTS ON HEMODYNAMIC RESPONSE TO HEMORRHAGIC SHOCK**

R.N. Younes, M.M. Itinoshe, D Birolini. Department of Surgery (LIM-62), University of Sao Paulo School of Medicine, Sao Paulo, Brazil

Jaundice is associated with increased perioperative morbidity and mortality rates. These complications are due in part to hemdynamic instability and poor tolerance to hypovolemia. The present study evaluates the effects of bile duct ligation (BDL) and reversal on hemodynamic response to hemorrhage and shock. Adult Wistar rats (n=39) were randomized into 4 groups: **Sham1** (n=10-sham operation for 7 days-shock), **Jaun** (n=10-BDL for 7 days, shock), **Rev** (n=10, BDL for 7 days, reversal for 7 days, shock), or **Sham2** (n=9, sham operation for 14 days, shock). BDL was performed under anesthesia using a vessel elastic loop, sutured to the abdominal wall. Jaundice was maintained for 7 days. Reversal was performed by pulling out the elasti vessel loop under anesthesia, and the rats were observed for another 7 days. Shock was induced by hemorrhage until MAP=50 mm Hg for 30 min. Following shock period, rats were observed for spontaneous MAP recovery. **RESULTS**: Bilirubin level (± SD)-pre-shock baseline

| | |
|---|---|
| Sham1 (n=10) | 0.4 ± 0.1 mg/dl |
| Jaun (n=10) | 7.5 ± 2.8 mg/dl (p<0.001) |
| Sham2 (n=9) | 0.2 ± 0.1 mg/dl |
| Rev (n=10) | 0.5 ± 0.3 mg/dl |

No significant differences on baseline MAP between groups (p=0.167). All groups beld similar volumes to reach MAP=50 mm Hg (p=0.421), but spontaneous recovery was blunted significantly slower in the jaundiced rats, compared to the other 3 groups (p=0.002). **CONCLUSIONS:** Jaundice did not affect the resistance to hemorrhage, but significantly decreased the ability for spontaneous recovery after shock. Reversal of jaundice after 7 days recovered hemodynamic response to shock in rats.

### F090

EXPERIMENTAL XENOTRANSPLANTATION OF FRESH ISOLATED AND CRYOPRESERVED PIG HEPATOCYTES IN CASES OF TOXIC LIVER FAILURE. COMPARISON OF TWO METHODS

N. Arkadopoulos, A. Papalois, M. Demonakou, A. Fotopoulos, B. Golematis, Th. Pataryas, J. Papadimitriou
2nd Department of Surgery, Department of Biology and 1st Department of Propaedeutic Surgery, University of Athens, Athens, Greece

Hepatocyte (Hc) xeno-transplantation (xeno-Tx) is a possible clinical application in the future, for patients with acute liver failure (ALF). The aim of the present study was to evaluate the efficacy of Hc xeno-Tx in cases of toxic acute liver failure (TALF), under different pre-Tx management of isolated Hcs. One single swine (female, 20 Kg) was used as donor of Hc. After total hepatectomy and 10 hours preservation of the liver in University of Wisconsin solution, Hcs were harvested using a modification of the portal vein collagenase perfusion technique (Type V, Sigma C-9263/1.3 mg/ml). Hcs viability was >90%. Thirty Lewis rats (200-300 g) were divided in three experimental groups: Group A (n=6): induction of TALF without further treatment. Group B (n=12): induction of TALF followed by xeno-Tx of $10^8$ Hc (fresh isolated) in 24 h. Group C (n=12): induction of TALF followed by xeno-Tx of $10^8$ cryopreserved (Cp) Hc (simply Cp at -20°C for a month) again in 24 h. Hcs were transplanted beneath the renal capsule and intrasplenically. TALF in all the groups was induced with a single dose (iv 20 mg/Kg) of N-Dimethylnitrosamine (N-DMNA). All rats (controls and recipients) were treated with cyclosporin A (CyA) 20 mg/Kg/day iv (days 0-15) and 10 mg/Kg/day (days 15-30). Total period of observation for surviving animals was 30 days. All rats in Group A died within 48 h. The survival rate in Group B was 6/12 (50%) and in Group C 5/12 (41.6%). The statistical comparison between Groups B and C showed no significant difference. Liver cells morphology in Hematoxyline/Eosin stain was intact.
We conclude that simply Cp Hcs are still viable to replace hepatic function and both methods have similar results.

### F091

LIVER GRAFTING IN RUSSIA - A NEW APPROACH TO END STAGE LIVER DISEASES TREATMENT

A.Eramishantsev, S.Gautier, O.Tsiroulnikova, O.Skipenko, A.Filin

Research Centre of Surgery, Moscow, Russia

According to new legislative abilities in donor organs supply in Russia 132 patients (123 adult - mean age 36,4± 1,0 years and 9 children - mean age 8,2± 1,6 years) with end stage liver disease were evaluated while selecting for orthotopic liver transplantation (OLT) during 1990-1994 years period. Sixty patients (45,5%) were listed for OLT. Fifteen OLT were performed in 14 patients including 1 retransplantation and 3 living related liver grafting without operative mortality. The indications were: unresectable hepatocellular carcinoma (HCC) in 4 cases, viral cirrhosis, primary biliary cirrhosis, primary sclerosing cholangitis in 2 each, Caroli's disease, biliary hypoplasia, alveolar echinococcosis and fulminant B-hepatitis in 1 each. One patient after OLT in HCC was retransplanted on the 13th day for severe graft rejection. The basic immunosuppression was double- or triple-drug protocol depending on Cy-A toxity. Seven - 44 mo survival was observed in 5 patients. While waiting 32 listed patients (53,3%) died in the shortage of donor organs. OLT in Russia is a new possibility for end stage liver diseases treatment. The high mortality rate in waiting list compels to check the indications for OLT as early as possible.

### F092

**AUXILIARY ORTHOTOPIC LIVER TRANSPLANTATION. A NEW THERAPEUTICAL OPTION FOR ACUTE LIVER FAILURE ?**

K.J. Oldhafer, G. Gubernatis, H.J. Maschek, H.J. Schlitt, H. Lang, B. Rodeck, R. Pichlmayr
Hannover Medical School, Hannover, Germany

Auxiliary partial orthotopic liver transplantation (APOLT) has been developed to enable the native liver to regenerate in acute liver failure and to avoid the risks of long-term immunosuppressive therapy. We present our experience with APOLT in patients with acute liver failure. **Patients:** So far, 4 APOLT procedures have been performed in our institution. The patients were 33, 18, 5 and 34 years of age. The causes of hepatic failure were one HELLP-syndrome, one paracetamol intoxication and remained undeterminded in the last two cases. All patients were in coma before transplantation. Date of first symptoms before APOLT has been 13, 2, 30 and 20 days. In all cases segment II and III of the recipient's own liver were resected before implanting the auxiliary liver orthotopically. The auxiliary graft consisted of segment II and III in two cases and of II, III and IV in the other two patients. **Results:** The auxiliary graft had an initial function in all cases. In the first 3 patients a full regeneration of the native liver was shown by biopsy. All 4 patients are alive 5 years, 1 1/4 year, 1/2 year and 1 month after transplantation. In the last patient an arterial thrombosis of the graft occured on the 5th postoperative day; the patient underwent successful retransplantation. The immunosuppressive therapy could be stopped in 3 cases and the graft was removed in two patients 15 and 40 days after APOLT. **Conclusion**: This report shows that APOLT represents an effective methode for patients with acute liver failure. This methode enables restoration of native liver function. Thus, APOLT should be considered in every patient with acute liver failure in whom a transplantation is indicated.

### F093

**AUGMENTATION OF DONOR-DERIVED CHIMERISM WITH BONE MARROW INFUSION IN RECIPIENTS OF LIVER TRANSPLANTATION**

**T. Karatzas, C. Ricordi, M. Webb, J. Nery, B. Cirocco, M. Carreno, E. Linetsky, V. Esquenazi, J. Miller and A.G.Tzakis. University of Miami, School of Medicine, Dept. of Surgery, Div. of Transplantation, Miami, Florida.**

**Chimerism is the result of spontaneous cell migration from the graft into the recipient and vice versa, after transplantation (Tx). Augmentation of chimerism in liver allograft recipients was attempted by infusion donor bone marrow cells (DBMC) in order to promote graft tolerance. Sixty-six OLTx were performed between July and December 1994. Twenty-seven recipients received DBMC ($5 \times 10^8$ cells/Kg body weight) and 39 no DBMC (controls). Ten of the 27 recipients received 1 DBMC infusion at the time of liver Tx (day 0) and 17 received 2 DBMC infusions on day 0 and 11. Immunosuppression was based on FK 506 and low dose of steroids. Additionally, 11 of the patients who received DBMC, OKT3 (5 mg, day 0-10) or steroid taper were randomly selected for induction. Chimerism was assessed on peripheral blood by semi-quantitative Polymerase Chain reaction (PCR) to evaluate DNA concentration in donor cells and by flow cytometry analysis to detect specific class II epitopes and to evaluate the concentration of lymphocytes expressing donor HLA-DR.**

**Bone marrow infusion was uneventful in all patients. DBMC increased significantly the chimeric state of all OLTx recipients (See below Figure 1 and 2). In the control group 4 of the 39 patients died (mucor mycosis of the brain (n=1), central pontine myelinolysis (n=1), cardiac failure (n=1), and lymphoma (n=1)). Eight control recipients underwent to retransplantation (PNF (n=2), hepatic artery thrombosis (n=3), accelerated rejection (n=2), and intra-hepatic bilomas (n=1)). In the DBMC group all patients are alive and none required retransplantation. Eleven control recipients developed acute rejection episodes, including 2 with accelerated rejection requiring retransplantation. The severity of rejection episodes in the 27 OLTx-DBMC recipients ranged from mild to severe acute and were successfully treated with steroids (bolus and recycle) and by increasing the dose of FK 506. There was one suspected case of mild GVHD of the skin which responded to steroid bolus.**

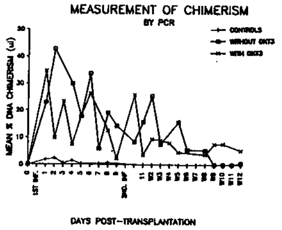

**Figure 1**

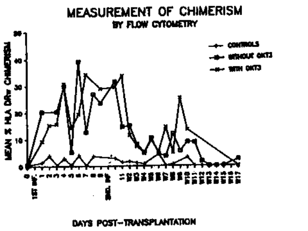

**Figure 2**

**Chimerish measured by PCR (Fig. 1) and flow cytometry (Fig.2) in controls and DBMC patients with and without OKT3.**

**These data suggest that cadaver DBMC infusion is safe and it augments significantly the chimeric state of the recipient. It may improve patient and graft survival.**

### F094

TECHNICAL EVOLUTIONS IN SPLIT LIVER TRANSPLANTATION

X. Rogiers, M. Malagó, K.A. Gawad, J. Schulte am Esch, J. Erhardt*, M. Gundlach and C.E. Broelsch
Departments of Surgery, University Hospital Eppendorf, Hamburg and University Hospital, Essen*, Germany

From 01.01.94 to 13.12.94 sixty-seven orthotopic liver transplantations were performed at the University of Hamburg. Of these, 14 (20.9%) were split liver transplantations (3 shipped from other centers) with a patient survival of 85,7 % (12/14) and a graft survival of 78,6 % (11/14).

Eigth of these transplantations are the result of a new generation of techniques for splitting.

Two livers were splitted using the *in-situ* splitting technique in the heart beating cadaveric donor. All 4 recipients are alive. One recipient of a left lateral segment suffered ischemic damage due to progressive portal steal after auxiliary grafting. After initial recuperation graft function is now progressively deteriorating. The others had an uncomplicated course and have normal liver function.

Two livers were splitted *ex-situ*, using a technique derived from the in-situ technique, allowing to avoid all dissection of the bifurcation of the bile duct and to minimize manipulations to the hilar structures of the right lobe. All four patients had uncomplicated postoperative courses (including one with an auxiliary orthotopic graft and a right graft shipped to Essen).

In summary, with the two new techniques, a 100% patient survival and a 100% graft survival (with probable functional loss of one graft) were obtained.

Conclusions: 1. Split liver transplantation can be performed safely using the in-situ or modified ex-situ techniques.

2. In-situ splitting allows shorter ischemic times and observation of the perfusion and hemostasis on both grafts before transplantation.

3. Shipping of splitted grafts between experienced centers is a valuable option.

### F095

AUXILIARY LIVER TRANSPLANTATION FOR FULMINANT LIVER FAILURE : LIMITS OF AN ATTRACTIVE CONCEPT.

J. Belghiti, R. Noun, F. Zinzindohoue, A. Sauvanet, F. Durand*, J. Bernuau*.
Departments of Digestive Surgery & *Hepatology, Hôpital Beaujon, F - 92110 Clichy.

Auxiliary liver transplantation (ALT) theorically bridges the period of acute liver failure until the native liver (NL) recovers so the graft could be removed and immunosuppression stopped. However, this attractive concept is burdened by technical problems and by the selection of candidates. We report our experience of ALT with special references to circumstances that may contraindicate an ALT and to factors of technical failure.

Patients: From April 1993 to August 1994, among the 14 adults who were candidates for emergent liver transplantation, 8 patients were excluded for ALT and received OLT because of age>60yrs (n=2), hemodynamic instability (n=2), pre-existing liver disease (n=2) and poor neurological status (n=2).Six patients (43%) underwent ALT including 4 with a full size graft (FG) after reduction of the NL (segments 1-4) and 2 patients with a partial graft (PG) with reduction of both the graft and the NL. Characteristics of patients and technical data are listed in the following table.

Results: Graft function, postoperative course and outcome are listed as follows:

| Patients sex/age | Graft size | weight/height D-R | vascular graft | PT day 1/3/10 | vascular complicat° | outcome |
|---|---|---|---|---|---|---|
| M/33 | FG | 61/161-60/183 | P | 10/62/67 | none | alive (19mth) |
| F/57 | FG | 70/175-110/160 | P | 9/46/- | PT | dead (day 6) |
| F/21 | FG | 70/175-51/172 | P+A | 13/41/49 | PT+AR | dead (day10) |
| M/55 | FG | 65/178-72/180 | P+A | 21/51/35 | none | dead (day16) |
| F/24 | PG(2-4) | 62/170-56/165 | none | 6/38/69 | PT* | alive**(10mth) |
| M/16 | PG(5-8) | 50/162-64/185 | none | 29/53/72 | none | alive (4mth) |

* successful thrombectomy, ** graft removal at 7 months.

FG = full-size graft, PG=partial graft, D= donor, R=recipient, P=portal, A=arterial, T= thrombosis, R=rupture.

Conclusion: ALT can provides a rapid liver function support, an immediate patient survival, and may enable native liver recovery. ALT seems inadequate in late stages of hepatic failure as the failing liver should be removed. The use of full-size graft and complex vascular reconstruction seems to be the main factors of poor short-term prognosis.

### F096

EXPERIENCE IN THERAPY OF CHRONIC LIVER ALLOGRAFT REJECTION

R. Charco, C. Ruiz, E. Allende, J Balsells, JL. Lázaro, E. Murio, I. Bilbao, E. Gifre and C. Margarit.
Liver Transplant Unit. Hospital General Universitario Vall d'Hebron. Barcelona, Spain.

From October 1988 to December 1993, 128 liver transplants (OLT) were performed in 118 adult patients. During follow-up, chronic allograft dysfunction was found in 24 cases. Liver biopsy showed graft hepatitis related to HCV reinfection in 16 cases and chronic rejection (CR) in 8, although 4 patients presented both CR and graft hepatitis. The mean time from OLT to presentation of CR was 9.5 months. In most patients, CR was preceded by several episodes of acute rejection.

Standard immunosuppression consisted of cyclosporine A (CsA) and prednisone (P), and FK 506 and P in 17 grafts (No graft developed CR in this group of patients). Rejection episodes were treated with 0.5-1 g bolus of P. If rejection persisted, a 14-day course of 5 mg/d of OKT3 was given. When episodes of acute rejection recurred or chronic rejection was suspected or confirmed by histology, patients were converted to FK 506. Seven of them with CR were converted. The other patient underwent retransplant since FK 506 was not available at the time of diagnosis of CR. Mean values of bilirubin, ALT, FA and GGT in patients with CR were 25.2 mg/dl, 347 UI, 1858 UI and 2718 UI when FK 506 was introduced.

**RESULTS:** Seven of eight allografts result in graft failure. In all grafts, CR or CR with HCV infection was confirmed in explanted livers (dead or retransplanted patients). Arteriolopathy was found in six specimens. One patient who underwent retransplantation died from sepsis a few days after retransplant. Of the patients treated with FK 506, one had an excellent response to FK 506 rescue therapy. Two patients progressed to graft failure despite FK 506 treatment and required retransplantation. One had a successful retransplant and the other died immediately after surgery from unrelated causes. The remaining four patients died: two on the waiting list for retransplant and two from sepsis and multiorgan failure. Overall patient survival was 25%.

**In conclusion**, CR remains an important cause of graft failure. Problems in diagnosis still persist, particularly, at an early stage in patients with HCV recurrence. We recommend early FK 506 conversion in all these patients.

### F097

HUMAN ISLET ISOLATION AND TRANSPLANTATION

L. Bühler, E. Andereggen, S. Deng, R. Mage, C. Bubloz, G. Mentha, Ph. Morel
Transplant Unit, Department of Surgery, University Hospital, Geneva, Switzerland

The recent technical improvements achieved in the field of islet isolation renewed the interest for cell transplantation. However, the isolation of a sufficient number of viable islets is still an important issue. We report hereih our experience of 29 human islet isolations performed during the last 20 months and the results of 5 patients undergoing a subtotal or total pancreatectomy with immediate intra-portal islet autotransplantation. We also performed one islet-lung allotransplantation in a cystic fibrosis patient with end-stage fibrotic lung disease and concomittant diabetes. **Methods:** The pancreases were processed using a modified automated method. After collagenase digestion, the tissue was purified on Euroficoll gradients, using a Cobe cell processor. For autotransplantation, after surgical resection, the pancreas was digested with the modified automated method and no purification was made. Under the control of portal pressure, the preparation was injected into the portal vein. Pre and post-operative metabolic tests were performed including a 24 hours glucose profile, oral and iv glucose tolerance test, glucagon test and the measurement of the glycosylated hemoglobin. For the islet-lung allotransplantation, we processed the pancreatic gland of one multiple-organ donor and injected the cell preparation into a colic vein, performing a small laparotomy just after the double-lung transplantation. **Results:** *Islet yield and purity*: the mean islet number prior to purification was 283'634 (113'400-615'600), representing a mean of 4'304 (1'350-9'618) islet/g pancreatic tissue. The mean purified islet number was 85'937 (13'400-259'000), with an average of 1'278 (206-2'901) islet/g pancreatic tissue. The mean ± SD purity was 59 ± 23%. *Islet autotransplantations*: four of the five autotransplanted patients are actually insulin-independent. One alcoholic patient, which started drinking again, developped a diabetic state and required introduction of insulin therapy 8 months after the autograft. Two patients presented some degree of glucose intolerence, but not necessitating insulin treatment, and two patients had a normal endocrine function, as assessed by the above mentioned metabolic tests, under a normal diet (follow-up 3 and 12 month). *Islet-lung allotransplantation*: The patient insulin requirement decreased significantly after the transplantation with concomittant normalisation of the plasmatic C-peptide values. **Conclusion:** The development of new isolation methods allowed us to perfom human islets auto and allotransplantations without morbidity and with some success considering the insulin requirement of the patients. As the islet number may be a limiting factor in the outcome of islet allotransplantation, we started a cryopreservation programm with the aim of pooling several cell preparations for one recipient.

### F098

USE OF COLOR-CODED DUPLEX SONOGRAPHY AFTER COMBINED PANCREATIC/KIDNEY TRANSPLANTATION

H. Lang, R. Lück, A. Weimann, M. Bartels, H. Bektas, R. Brunkhorst, J. Klempnauer, R. Pichlmayr
Klinik für Abdominal- und Transplantations-chirurgie, Medizinische Hochschule Hannover

In combined pancreas and kidney transplantation (PTX/NTX) graft dysfunction due to rejection or surgical problems may occur in each organ alone or in both organs simultaneously. We evaluated color-coded duplex sonography in the assessment of the postoperative course in pancreatic and kidney transplants.
The charts of nine patients were analysed retrospectively. The results of color-coded duplex ultrasound including measurement of the arterial resistive index (RI) were compared with the clinical course in these patients.
In normal graft function the mean resistive index was 0.69 (range 0.60-0.80) for the kidney and 0.61 (range 0.55-0.70) for the pancreas. Ten episodes of graft dysfunction (kidney n=4; pancreas n=6) were observed. During renal rejection or hemolytic uremic syndrome the RI was above 0.80 while cyclosporine toxicity concurred with normal indices. In pancreatic rejection the RI exceeded 0.80 whereas all other causes of pancreatic dysfunction were not associated with changes in the resistive index.
Color-coded duplex sonography is a reliable noninvasive diagnostic method in the evaluation of the postoperative course after combined PTX/NTX and may contribute to the differential diagnosis of graft dysfunction.

### F099

**ENERGY METABOLISM FAILURE IN EARLY GRAFT INJURY AFTER PANCREATIC TRANSPLANTATION IS IMPROVED BY FUT-175.**

F. Marotta, *P. Safran, +J Wu, +DH. Chui, G.Barbi.
GI Unit, S.Anna Hosp., Como,Italy; *Econum Villeneuve d'Ascq, France; + Int. Med. Dept., N. Bethune Univ., Changchun, China

Following pancreatic transplantation (PTx), metabolic preservation of the graft is a prominent issue. In this study we tested FUT-175 on energy metabolism in the graft. Donor pancreatectomy and PTx on syngeneic rats was done. Rats were given 3 perfusion media via sup. mesenteric artery: A) Saline; B) Eurocollins; C) FUT-175 1.0mg/ml. Sham-operated rats served as control. Rats were sacrificed 1, 3, 6 and 12hr afterwards and malate dehydrogenase (MDH), amylase and trypsin were examined in portal blood. Pancreatic mythocondria-rich pellets and soluble suspensions were tested for MDH-activity, ATP, ADP, AMP and related Energy Charge (EC). A significant ($p<0.01$) time-course increase of portal MDH occurred in group A and B but not in C. The increase of portal amylase and trysin was of lesser degree in group C as compared to A and B ($p<0.05$). Mithocondrial fragility and adenine nucleotides level showed a time-course increase in group A and B and, to a significantly ($p<0.05$) lesser extent in C. However, the overal EC in group C was comparable to control. The present data suggest that FUT-175 added to perfusion mèdium offers a significant protection of energy metabolism of pancreatic graft.

### F100

ENDOSCOPIC TREATMENT OF PANCREATIC AND BILIARY FISTULA

Z.Wajda, M.Dobosz, A.Babicki

II Department of Surgery,Medical University of Gdańsk, Poland

Authors present 16 patients with biliary or pancreatic fistula. In 11 cases, ERCP examination revealed biliary fistula, in 5 patients pancreatic fistula was diagnosed. The causes of biliary fistula were cholecystectomy in 7 patients /4 laparoscopic and 3 classical/, T-tube drainage failure in 2 patients, stab abdominal wound and blunt abdominal trauma in one patient each. Pancreatic fistula was a consequence of necrotizing pancreatitis in 2 patients, and in patients undergone insulinoma enucleation, distal pancreatic resection and after blunt abdominal trauma. In all the patients, endoscopic papillotomy was performed within 9-18 days after the fistula was diagnosed. Eight biliary fistulas and three pancreatic fistulas healed spontaneously, following endoscopic papillotomy only. The bile and pancreatic juice output of the fistulas was reduced for about 75% within 2 days after the papillotomy. Three patients with biliary and two with pancreatic fistula required additionally an endoprosthesis insertion. In these patients, the fistulas were also healed within 2 weeks after the prosthesis implacement. Authors conclude, that endoscopic papillotomy is an effective method in biliary and pancreatic fistula treatment. Consecutive endoprosthesis implacement is necessary in cases, when papillotomy is uneffective in biliary and pancreatic fistula healing.

### F101

ENDOSCOPIC THERAPY OF DIFFICULT BILE DUCT STONES

A. Vezakis, D. Babalis, A. Polydorou
Department of Surgery, Hippocration Hospital, Athens, Greece

Common duct stones can be successfully removed in 85-90% of patients after endoscopic sphincterotomy using standard techniques. The remaining patients usually have large stones (>1,5 cm). A variety of lithotripsy techniques (mechanical, electrohydraulic, laser, extracorporeal) and dissolution therapies have been developed to circumvent the problem.

Between 1990 and 1994, 564 patients (M/W 250/314, mean age 65) with bile duct stones were referred for endoscopic treatment in our Unit. Endoscopic sphincterotomy was successful in 556 (98%). In 482 patients (85%) stones were removed using standard dormia baskets and balloon catheters. The remaining 74 patients (M/W 30/44, mean age 73) had difficult bile duct stones. Mechanical lithotripsy (ML) was applied in 26 patients with success rate 23/26 (88%), extracorporeal lithotripsy in 2 with success 1/2 (50%). In 43 patients with non extractable stones two pig tail stents 7Fr were placed and ursodeoxycholic acid was given orally. Fifteen of them had clearance of their duct after a new procedure 3-6 months later. Twenty-two patients still have the stents and only 4 of them suffered an episode of cholangitis which was managed with stent change. Operation was needed in 13/74 patients (17%) for definite treatment.

Conclusively our results support ML as an efficient method for the treatment of difficult stones while pig tail placement is an attractive alternative when ML is not possible.

### F102

**IS ENDOSCOPIC SPHINCTEROTOMY LEAVING THE GALLBLADDER IN SITU AN ALTERNATIVE TO OPEN SURGERY FOR TREATMENT OF BILE DUCT STONES IN THE HIGH RISK PATIENT?. A PROSPECTIVE AND RANDOMICED TRIAL COMPARING BOTH PROCEDURES.** EM Targarona, RMª Perez Ayuso, JM Bordas. I Pros, J Martinez, E Ros, J Terés, M Trías. Serv. of Surgery, Gastroenterol and Endoscopy . Hosp.Clinic. Univer. of Barcelona

Endoscopic sphincterotomy leaving the gallbladder 'in situ'(EE-GiS) has been proposed as definitive treatment of choledocholithiasis in the elderly or high risk patient, but biliary symptoms up to 30% during the follow up has been reported. In adittion, this option never has been compared with open surgery in a prospective trial. **AIM:** To compare the eficacy of open surgery with EE-GiS for treatment of bile duct stones in the high risk patient. **MATERIAL AND METHODS:** 100 patients suspected to harbour duct stones were randomly allocated to surgery. **Group I**, n: 48 or EE-GiS; **Group II**, n: 50. Criteria for suspicion of the existence of bile duct stones were jaundice, cholangitis or pancreatitis + US showing gallstones + dilated bileduct. High risk were stablished as age > 70 y. or severe disease (cardiac, pulmonary, liver chirrosis or limited mobilization). **RESULTS:** 100 patients entered in the study. 2 were withdrawn for wrong randomization.

| | SURGERY | % | EE-GIS | % | p |
|---|---|---|---|---|---|
| **N** | 48 | | 50 | | |
| **Age** (y) | 80 ± 7 | | 79 ± 9 | | ns |
| **Technical succes** | 45 / 48 | 94 % | 45 / 50 | 90 % | ns |
| **Bile duct stones** | 26 / 48 | 54 % | 25 / 50 | 50 % | ns |
| **Morbidity** | 11 / 48 | 23 % | 7 / 50 | 16 % | ns |
| **Mortality** | 2 / 48 | 4 % | 3 / 50 | 6 % | ns |
| **Postop stay** (d) | 11 ± 8 | | 5 ± 4 | | .001 |
| **FOLLOW UP** (m) | 18 ± 10 | | 15 ± 11 | | ns |
| **N** | 43 | | 46 | | |
| **Biliary morbidity** | 3/46 | 6.5 % | 10 / 47 | 20 % | .002 |
| **Surgical procedures** | 0 / 46 | | 7/ 47 | 15 % | .02 |

**CONCLUSION:**Standard surgical therapy of choledocholithiasis in the high risk patients is more succesful than EE-GiS regarding the prevention of long term biliary complications, without influence on immediate morbimortality or long term survival.

### F103

ENDOSCOPIC SPHINCTEROPLASTY IN PATIENTS WITH BILIARY LEAK AFTER ORTHOTOPIC LIVER TRANSPLANTATION (OLT)

L.S. Leonardi, F. Callejas Neto, G. Berenhauser-Leite, I.F.S.F. Boin
Department of Digestive Diseases Surgery, State University of Campinas, Campinas-SP, Brazil

The biliary leak after OLT occurs in 13 to 40%.

In our Service we performed 17 OLT (16 patients) and 2 patients (choledochocholedochoanastomosis) was realized ERCP to see the place of the leak.

The first case (OLT nº 12) ERCP showed leak around of the T-tube and 1.1 cm of stenosis above and the receptor's biliary tract was normal. In second case (OLT - 17) we observed that the leak was below (1.0 cm) of the T-tube and the receptor tract biliary had 1.5 cm and it was tapering inside the pancreas.

We performed sphincterotomy in two cases with a good solution. The leak disappeared in 5 days.

In these cases the ERCP was effective to diagnose and to treat the biliary leak.

### F104

**THE VALUE OF MINIMALL ACCESS APPROACH IN PATIENTS WITH CHOLEDOCHOLITHIASIS.**

Simutis G., Bubnys A. Clinic of Abdominal Surgery, Vilnius University Santariskiu Hospital, Santariskiu 2, 2600 Vilnius, Lithuania.

**Introduction:** The perioperative diagnosis and modern treatment of choledocholithiasis is controversial. The aim of this report was to evaluate our experience and results of the two-stage procedure (combined endoscopic-laparoscopic approach) in patients (pts) with gallbladder (GB) and concomitant common bile duct (CBD) stones.

**Patients and methods:** A retrospective review was performed of all pts presenting with GB and CBD stones who underwent endoscopic clearance of CBD before laparoscopic cholecystectomy (LC) between December 29,1992 and December 1,1994. We present a single-centre study with 750 consecutive pts with a mean age 50,1 years (range 15-85 years).

**Results:** CBD stones were suspected in 52 (7%) pts preoperatively. They were detected in 38 (5,6%) pts on preoperative endoscopic retrograde cholangiography (ERCP). Stones in CBD on ultrasound and current jaundice had the highest positive predictive value 78,6% and 65%, respectively. The sensitivity was 28,9% and 68,4%, respectively. Endoscopic clearance was achieved in 15 pts after initial endoscopic sphicterotomy (ES), CBD stones passed spontaneously in 11 pts, and in 12 pts CBD stones were extracted after second and third (5 pts) procedure. No serious complications because of ERCP or ES we encountered. LC was performed with confidence within some 3-4 days after stone removal. Four (0,5%) pts underwent ERCP-ES due to jaundice after LC. Silent CBD stones were confirmed.

**Conclusion:** We recommend preoperative ERCP and ES only when we know that a stone is present in bile ducts, i.e., when the patient is jaundiced or a stone has been detected on sonography. Treatment of GB and CBD stones by combined endoscopic-laparoscopic approach is a safe and effective.

### F105

**MAJOR RESECTION AND GRAFTING IN ALVEOLAR ECHINOCOCCOSIS OF THE LIVER**

**S.Gautier, O.Tsiroulnikova, O.Skipenko, J.Kamalov, G.Sorokin and A.Eramishantsev**
**Research Centre of Surgery,**
**Moscow, Russia**

**Curative liver resection in advanced alveolar echinococcosis (AE) similar to primary liver malignancies still remains the method of choice among conservative treatment, palliative surgery and orthotopic liver transplantation (OLT). Large primary liver tumours with 4 to 8 segments involved were evaluated in 38 patients, 13 males and 25 females of 1 to 61 years old (mean age - 35,1 ± 2,5 years) while selecting recipients for OLT during 1990 - 1994 years period. AE was found in 4 (10,5%) patients. Major liver resections (7 right and 2 left extended hepatectomies, 3 right hepatectomies) were performed in 12 (31,6%) patients. Two right extended hepatectomies with biliary reconstruction were made for AE. Eleven (28,9%) patients listed for OLT included 2 with AE (due to Budd-Chiari syndrome with cirrhosis in one and total segmental involvement in the other case). The first one died while waiting because of hepato-renal syndrome. The other was successfully grafted and doing well with triple immunosuppressive protocol. The frequency of advanced AE in some countries including Russia forces to rise the radicality of treatment. OLT must be discussed as more preferable procedure comparatively with palliative surgery in AE when curative resection is impossible.**

### F106

"PERCUTANEOUS ASPIRATION AND DRAINAGE OF HYDATID CYSTS". IS IT HELPFULL FOR DIAGNOSIS AND TREATMENT?
(Preliminary report of a prospective study)

S.Kılıçturgay, Y.Sadıkoğlu, Y.Özen, C.İrgil, N.Korun, H.Bilgel

Uludağ University, School of Medicine, Department of Surgery, Bursa, TÜRKİYE

Although it is thought to be, the surgical terapy is essential for hydatid disease, morbidity rate is very high and local rekürrens and surgical treatment for secondary hydatidosis have mostly been serious problem for surgeons. Also diagnosis of the some suspicious hepatic lesions may be another problem. In this prospective study, the preliminary reports of percutaneous needle aspiration of hydatid cyst which has recently been acepted as a terapotic modalite are presented.

US or CT guided needle aspiration has been applied to the Twentyfive cysts in 22 patients. Cysts have been established radiologically by CT and US, and also serologic studies have been done. 14 of the cases had been operated because of previous hydatid disease and the remaining were thought to be primary hydatid cysts.

In all patients, during the 4 days before the catheter was positioned the patients were given mebendazole at the dosage of 40-50mgr/kg/gün or albendazole at the dosage of 10-15 mgr/kg/gün. The last preoperative dose of the drug was administered 4 h before drainage. Needle (19-22 gauge) positioning was performed with US or CT guidance in a fluoroscopic room. Specimens were submitted for cytologic and microbiologic assessment. Subsequent to percutaneous aspiration, this US or CT image shows that the inner membrane was detached and is seen undulating in the hydatid fluid. According these findings, diagnosis of hydatid cyst was established. In the patients who had hydatid cyst, a 5-F or 8.3 F pigtail catheter was inserted in the cystic cavity. The fluid was then aspirated and a cystogram was obtained to rule out a connection with the bile ducts. Subsequently, the cavity was irrigated hypertonic saline for 5 minutes and 95 % ethanole (a sclerozan agent). Then, the fluid was reaspirated and the catheter was immediately removed from the cyst cavity smaller than 5cm.

In twelve patients who had fifteen hydatid cysts therapeutic drainage procedure were performed. Two patients were referred to the surgery because of cysto-biliary fistule was found. In the one patient who was very old women, some commünication problems were developed after insertion of needle, so we gave up contnue to the procedure. Other 7 cases were not diagnosed as hydatid cysts after the diagnostic aspiration so, therapeutic drainage was not performed for these patients. One of these patients was thought to be primary hydatid cysts and rest of them were thought to be secondary hydatidosis. No severe complication occured after diagnostic aspiration. In 4 patients urticaria and pruritus develeped after terapotic aspiration. All of them recevied conticosteroids. In 2 patients abses were developed 1-2 week after removel of the catheter. And percutaneus aspiration was performed for one case and surgical drainage was used for the other one. There is no mortality.

Patients were followed up with US and CT. Follow up period was 14-24 months and any recurrens have not been established yet. As a conclusion, using this procedure, may not only help to treat of some type of hydatid cysts, but also help to diagnosis of suspicious lesion (ie, postoperative collections or recurrens, Type I hydatid lesions)

### F107

### F108

### Therapeutic indication for small liver cancer

Toru Otani, Hiroshi Kasugai[1)], Makoto Fujita[2)], Yo Sasaki[3)]
Dept. of Gastroenterology[1)], Radiology[2)], and Surgery[3)].
The Center for Adult Diseases, Osaka, Japan

Therapeutic indication for small liver cancer (hepatocellular carcinoma) equal to or smaller than 2 cm in diameter was investigated in relation to their back ground factors and prognosis.

A total of 185 cases with small liver cancer was evaluated. There were 142 males and 43 females, 118 solitary and 67 multiple tumors, and ages distributed 31-85 years old (mean 60 y. o.). Among all, 104 cases (56%) were resected, 49 cases (26%) received transcatheter arterial embolization, 21 cases (11%) were treated with percutaneous ethanol injection and 11 cases (6%) with other therapy. Each of 3-, 5-year's survival rates and 3-, 5- year's recurrence rates was 68%,42%, 61%, 83% in the all cases, 81%, 50%, 54%, 80% in the resected cases, 76%, 38%, 70%, 85% in the ethanol injection cases, and 51%, 33%, 78%, 85% in the embolization cases, respectively. The survival rate of ethanol injection was as good as that of resection, but the recurrence rate was higher in statistical significance. In the all cases, the survival rate in solitary tumor group was higher than in multiple tumor group, but there was no difference in the recurrence rate between the two groups. In the group with good liver function, the survival rate was higher and the recurrence rate was lower than those with poor liver function, respectively. The survival rate of the patients with solitary tumor and good liver function was similar between resection and ethanol injection therapy, but the prognosis of those with solitary tumor and poor liver function was better in ethanol injected group than in resected group. In multiple tumor cases, survival of resection and ethanol injection group were higher than that of embolization cases.

Therapeutic indication of small liver cancer should be considered depending on the back ground factors such as liver function and tumor number.

### F109

**LAPAROSCOPIC MANAGEMENT OF COMMON BILE DUCT (CBD) STONES AND GALLSTONES IN 110 UNSELECTED, CONSECUTIVE PATIENTS**

A. Paganini, *F. Carlei, F. Feliciotti, *D. Lomanto, M. Guerrieri, *M. Nardovino *M. Sottili, E. Lezoche
Istituto di Scienze Chirurgiche, Università di Ancona. *I.N.I. Canistro, L'Aquila. Italy

A prospective study to evaluate feasibility, success rate, safety and short-term results of the laparoscopic management of ductal stones during LC in 110 unselected, consecutive patients was undertaken.
CBD stones were proven at routine intraoperative cholangiography and choledochoscopy in 110 patients out of 992 with gallstones undergoing LC. Unsuspected CBD stones were present in 43 patients (4.3% of 992; 39% of 110). 26 patients were referred for surgery after failed ES performed elsewhere. Laparoscopic trans-cystic duct CBD exploration (LTC-CBDE) was the procedure of first choice. When this was not feasible, laparoscopic choledochotomy and direct CBD exploration (LD-CBDE) was performed. Use of biliary drainages was liberal. Laparoscopic treatment of CBD stones was successful in 106 patients (96.3% success rate), after LTC-CBDE in 72 and after LD-CBDE in 34. Four patients were converted to open surgery (3.6%), 2 of whom were among the first 5 patients treated and were related to inadequate instruments and experience. Retained stones, observed in 6 patients, were treated by ES in 2 cases, percutaneous endoscopic/fluoroscopic lithotripsy in 3, ESWL in 1. Minor morbidity included biloma (2), port site infection (2) and subumbilical hematoma (1). Major morbidity was bile leakage from the cystic duct stump in 2 cases, due to clips (1) and trans-cystic duct drainage displacement (1), respectively. Mortality was observed in 1 elderly, high risk patient referred after several failed attempts of endoscopic clearance, who died from cardiogenic shock 3 days after successful laparoscopic treatment.
Laparoscopic management of CBD stones during LC is feasible and safe with high success rates. Short-term results compare favourably with those of sequential ES/LC reported in the literature.

### F110

LAPAROSCOPIC CHOLECYSTECTOMY.

O.LUTSEVICH,M.D., S.GORDEEV,M.D., Ju.PROCHOROV, A.BRONSTAIN,M.D.
Center of endosurgery & lithotripsy, Moscow, Russia.

Our experience with laparoscopic cholecystectomy (LC) for symptomatic cholelithiasis has involved 1850 patients. Patient age ranged from 15 to 78 years; most were female (1498). 52% had weight more then 80 kg, 79 patients - more then 120 kg. We have no contraindications to LC except terminal patient's condition. 1219 patients had a simple chronic cholecystitis, 631 - complicated cholecystitis, included chronic inflammatory changes of GB (318), acute cholecystitis (259), bilio-digestive fistulas (3), common bile duct stones (44), perivesical abscess (7). To remove CBD-stones we used pre- and postoperative endoscopic sphincterotomy-balloon technique, laparoscopic choledocholithotomy, postoperative extracorporal lithotripsy. 8 patients (0,43%) required conversion to open cholecystectomy because of technical difficulties with the dissection. 28 patients underwent the combined operations: LC and laparoscopic hernioraphy, myomectomy, cystectomy e.s. The complication rate was low: no deaths, 0,27% major and 0,76% minor complications. Average time of operation was 37 min, postoperative hospital stay was 1-2 days. Most patients resumed normal activities within 1 week after discharge. LC is a safe and effective procedure that can be performed with minimal risk.

### F111

LAPAROSCOPIC ULTRASOUND: A REAL ALTERNATIVE TO CHOLANGIOGRAPHY DURING CHOLECYSTECTOMY?

R.Santambrogio, P.Bianchi, E.Opocher, F.Ghelma, A.Mantovani, M.Verga°, L.Federico°, G.P.Spina*
Clinica Chirurgica VI (Prof. G.Vincre) - Università di Milano;
* Chirurgia 2 (Prof. G.P.Spina) - Osp. Fatebenefratelli e Oftalmico;
° Chirurgia 1 (Dr. C.Corsi) - Osp. San Paolo - Milano

**Aim of this study was to evaluate laparoscopic ultrasound (LU) as a method to examine the biliary tree and related structures. We recruited 77 patients with symptomatic cholelithiasis (27 males, 50 females, mean age 47.6 ± 14.8 years). A preoperative ultrasound examination was performed 24 hours before laparoscopic cholecystectomy (LC); preoperative clinical history, biochemical tests and ultrasonography identified 5 pts. at high risk of choledocholithiasis who were submitted to endoscopic retrograde cholangiography: it identified 4 choledocholithiasis; an endoscopic sphyncterotomy solved it. Therefore 72 pts. at low risk of choledocholithiasis were studied. A LU probe (Aloka. Tokyo - Japan) with a 7.5 MHz linear array transducer, passed through 10mm trocars was used: a 10mm trocar was inserted through an umbilical incision and two others through left upper midclavicular and subxyphoid sites. Ultrasound was repeated 30 days after the operation. The scanning time for LU was 8±2 minutes. A satisfactory examination of the common biliary duct was obtained in 68 cases (94%). In 3 patients (4%), LU showed unsuspected choledocholithiasis and a subsequent intraoperative cholangiogram confirmed this. Cystic duct was not visualized in 12 patients (17%) as it was small or short or filled with biliary debris; it was well visualized by longitudinal scanning of hepato-duodenal ligament in 7 patients (10%), by transversal scanning in 39 patients (54%) and by both scanning in 14 patients (19%). During the follow-up period, one patient had an episode of acute pancretitis: ERCP showed a small stone wedged in the sphincter of Oddi with a non-enlarged common bile duct. In conclusion LU may be a real alternative to cholangiography during LC: it is safer and offers a complete examination of the biliary tree in 94% of cases.**

### F112

**LAPAROSCOPIC OPERATIVE CHOLANGIOGRAPHY IS BETTER THAN ENDOSCOPIC ULTRASONOGRAPHY TO DETECT ASYMPTOMATIC COMMON BILE DUCT STONES.**

French Association for Surgical Research (A.R.C.I.F.), Philippe OBERLIN, Thierry MONTARIOL, Claude REY, Alain CHARLIER, Simon MSIKA, Jean Marie HAY. Centre Hospitalier de Villeneuve-Saint-Georges.
94195 - VILLENEUVE-SAINT-GEORGES - France.

A prospective study was conducted to assess the performance of Endoscopic Ultrasonography (EUS) and of Laparoscopic Operative Cholangiography (LOC) for detection of asymptomatic common bile duct (CBD) stones. From October 1992 to October 1994, 227 consecutive patients, in 13 surgical centers, were enrolled. All had symptomatic cholecystolithiasis and were candidates for laparoscopic cholecystectomy. They had a high risk of CBD stones according to Huguier's score (1). EUS and LOC were attempted in all cases. When CBD stones were detected, by either methods, CBD exploration was performed. When EUS and LOC were normal, the CBD was not explored. EUS was successfull in 225 patients (99.1%) and LOC in 205 patients (90.3%). For the 203 patients who had both procedures, sensitivity and specificity were 0.86 and 0.93 for EUS, and 1 and 0.99 for LOC, respectively. Assuming a prevalence of 20.2% of CBD stones, positive and negative predictive values were 0.77 and 0.96 for EUS, and 0.98 and 1 for LOC, respectively. This study suggests that liability of EUS is higher than LOC, but performance values of LOC are better than EUS, on all levels. We believe that LOC has to be attempted in all patients with a high score for choledocolithiasis, undergoing laparoscopic cholecystectomy. Routine preoperative EUS should not be proned in these patients.

1. Huguier M *et al.*: Surg Gynecol Obstet. 1991 ; 172 , 470-4.

### F113

BILIARY ANASTOMOSIS WITHOUT T-TUBE IN LIVER TRANSPLANTATION. THE EFFECT OF COLD ISCHAEMIA.

A.Rafecas, J.Figueras, J.Fabregat, J.Torras, C.Cañas, C.Sancho, A. Montserrat, E.Ramos, E.Jaurrieta.
Liver Transplant Unit. Hospital Bellvitge. University of Barcelona. Spain.

The aim of the study is to asses the frequency and causes of complications in biliary anastomosis in liver transplantation (LT), without T-tube. Since September 1991 to August 1994, 127 LT were carried out in 112 patients, 74 male and 38 female, with a median age of 49.49 ± 11 years. Fifteen patients died during the first three months after LT from unrelated causes and consequently were discarded from the study. Mean ischemia time was 538.31± 211.45 minutes. In 101 case the anastomosis was performed between both biliary ducts, 67 end-to-end, 34 side-to-side. In 11 cases a hepatico-jejunostomy (HY) was performed with Roux-en-Y limb). There were 27 complications in 19 patients (16.9%). Thirteen stenosis, 7 fistulas, 4 lithiasis, 4 ischemic lesion of the biliary tract (in 3 cases associated with artery thrombosis). Thirteen out of 19 patients were reoperated (6 retransplantation, 6 HY and 1 repair of the end-to-end anastomosis). Two complications were managed percutaneously. Two patients were solved without surgical, endoscopic or percutaneous manoeuvres. Three of the 19 complicated patients died, from not related biliary complications. Cold ischaemia was 654±174 minutes in the complicated and 521±205 in the uncomplicated patients (p=0.01).
CONCLUSION.- The biliary anastomosis without T-tube in LT is a safe procedure, with similar complications to the use of the T-tube. Biliary complications are more frequent after prolonged cold ischaemia time.

### F114

INDUCTION OF PROSTAGLANDIN MEDIATED REGULATION OF BLOOD MONONUCLEAR CELL INTERLEUKIN-6 RELEASE IN PATIENTS WITH ACUTE PANCEATITIS

A.C. de Beaux, J.A. Ross, R.G. Molloy, K.C.H. Fearon, D.C. Carter
University Department of Surgery, Royal Infirmary, Edinburgh, Scotland. EH3 9YW

Prostaglandin $E_2$ ($PGE_2$) has been identified as mediating intra-cellular regulatory control to limit tumour necrosis factor release from activated leucocytes. Such control is blocked by cyclo-oxygenase inhibitors (indomethacin) and restored with exogenous $PGE_2$. The regulatory mechanisms for IL-6 release are less well known. We have measured release of IL-6 into the cell culture supernatant of peripheral blood monoculear cells (PBMCs) after 24 hours incubation in the presence or absence of indomethacin (I)($10^{-6}$ M) or I and $PGE_2$, (both $10^{-6}$ M). Cells were isolated from 6 healthy volunteers and from 14 patients with acute pancreatitis (6 severe, 8 mild: Atlanta classification) on the first day of admission. The results are shown in the table as the mean(SEM).

| | Control IL-6 (pg/ml) | Mild IL-6 (pg/ml) | Severe IL-6 (pg/ml) |
|---|---|---|---|
| No I or $PGE_2$ | 791 (168) | 2855 (615) | 3488 (980) |
| I alone | 941 (212) | 7669 (1438) | 8082 (2164) |
| I and $PGE_2$ | 4410 (981) | 8298 (1525) | 11181 (4429) |

Indomethacin had no effect on IL-6 release in the control group (p=0.2 paired t test), while it significantly increased IL-6 release in patients with both mild and severe disease (p=0.001 and 0.02 respectively). In the presence of I, addition of $PGE_2$ increased IL-6 release in the controls (p=0.001) but had no effect in the pancreatitis groups (p=0.62 and 0.27) when compared with I alone. These observations suggest that a product of the cyclo-oxygenase pathway (which does not appear to be $PGE_2$) is induced in PBMCs to minimise the increased release of IL-6 observed from PBMCs in patients with acute pancreatitis. Such regulatory control is inhibited by indomethacin and the use of such agents may adversely influence pro-inflammatory cytokine release in patients with acute pancreatitis.

### F115

TECHNIQUES FOR BLEEDING ESOPHAGEAL VARICES : YAG-LASER COAGULATION VERSUS SCLEROSING THERAPY

A.Severtsev, V.Shugurov, Yu.Malov
Dpts.of Surgery and Endoscopy, Medical Centre for Russian Government (MCRG);Hosp.N51, Moscow, Russia

The number of patients with portal hypertension increased during last years in Russia.YAG-laser coagulation (LC) and endoscopic sclerotherapy (ST)are used in the treatment of acute variceal bleeding.The aim of this study was to compare 2 types of treatment.
Thirty two consecutive cirrhotic patients with a bleeding from esophageal varices were randomly assigned to treatment with LC(10 patients) and ST(22 patients).We used Nd:YAG laser (Medi-Las 4060N;Dornier, FRG) for LC and Polidocanol (Aethoxysklerol, Kreussler, FRG). The protocol for ST was standard and we used laser by repeated short pulses (1-2 s) with the power 40-60 W for LC.
No difference in age, sex, severity of hemorrhage, etc. were found between 2 groups. We had two deaths during emergency endoscopy (1 in ST-group/1 in LC-group). The stable results were after the 2nd procedure for ST and after the 1st procedure for LC. Failures of treatment were at both groups.The process of coagulation was more controlable for ST. The price of LC-treatment was higher then for ST.
Laser irradiation appears to be safer and as effective as endoscopic sclerotherapy in controling of acute bleeding. The limit for Russia is high price of Nd:YAG-laser units then pharmacological sclerosing products.

### F116

**HEPATIC RESECTION WITH VASCULAR ISOLATION AND ROUTINE SUPRACOELIAC AORTIC CLAMPING**

MS Stephen, PJ Gallagher, AGR Sheil*, DM Sheldon, DW Storey.
*Departments of Upper Gastrointestinal Surgery and Liver Transplantation*, Royal Prince Alfred Hospital, Sydney. AUSTRALIA.*

Hepatic resection with total vascular isolation reduces haemorrhage and the addition of supracoeliac aortic clamping (THIS) putatively avoids haemodynamic instability but may increase morbidity.
THIS and division of the hepatic parenchyma with scalpel was used exclusively to resect 18 benign, 25 primary malignant and 56 secondary malignant hepatic lesions. There were 59 hemihepatectomies and 40 segmentectomy(ies) and median results varied accordingly. Clamp time was 18 and 13 mins, operative time was 145 and 110mins, transfusion requirements were 4 and 0 units, ICU stay was one day in both groups and postoperative stays were 11 and 9 days. The only significant biochemical changes were the transaminases which returned to normal by the first postoperative visit. Three patients required return to theatre for bleeding, 5 had subphrenic collections (4 drained percutaneously), 1 had a biliary fistula, 1 had portal vein thrombosis and 2 developed DVTs. Mortality varied according to whether cirrhosis and/or portal hypertension were present. One of 86 patients with normal livers died (acute Budd Chiari syndrome and urinary fistula due to the anterior resection), 0 of 5 with cirrhosis and no portal hypertension died but 5 of 8 with portal hypertension and cirrhosis died (3 with bleeding oesophageal varices and 2 with acute liver failure).
THIS is a safe and expedient method of liver resection provided there is no portal hypertension.

### F117

MODIFICATION OF CYTOKINE PRODUCTION IN SEPTIC CONDITION FOLLOWING NECRITIZING PANCREATITIS

G. Farkas, Y. Mándi°, J. Márton

Department of Surgery, Institute of Microbiology°, A. Szent-Györgyi Medical University, Szeged, Hungary

Sepsis and septic shock are the most frequent complications of extended necrosis following acute pancreatitis. The purpose of this study was to evaluate the role of cytokines in these conditions and to elaborate a new strategy in the treatment. The serum TNF, IL-1, IL-6 and sICAM-1 levels were determined in 25 patients with infected necrosis and multiple pancreatic abscesses. The **in vitro** cytokine-producing capacity of the leukocytes was also checked. Increased TNF and IL-1 serum levels were found in 30% of the patients, while the IL-6 level was elevated in all of them. There was a positive correlation between the serum IL-6 and sICAM-1 levels. The **in vitro** TNF and IL-6 producing capacities were initially higher in the study group, but decreased on subsequent days, especially in fatal cases (n=3). Pentoxifyllin (PTX) inhibited the TNF production of peripheral white blood cells stimulated by either LPS or **Staphylococcus aureus**. Administration of PTX (200 mg/day) to septic patients following necrotizing pancreatitis resulted in TNF and IL-6 production similar to that observed in control donors. The level of sICAM-1 decreased following PTX therapy. It also subsequently led to an improvement of the clinical status as assessed by the APACHE II score. These results suggest that cytokines produced by activated leukocytes are important in the pathogenesis of complicated acute pancreatitis, and their inhibition might be of therapeutic advantage.

Supported by Grant OTKA 2727.

### F118

BRADYKININ-ANTAGONIST HOE 140 PREVENTS MICROCIRCULATORY STASIS AND TISSUE NECROSIS IN TAUROCHOLATE INDUCED PANCREATITIS OF THE RAT.

C. Bloechle, R. Kühn, W.T. Knoefel, K. Kusterer, J.R. Izbicki.
Dept. of Surgery, University of Hamburg, 20251 Hamburg, FRG

The bradykinin-antagonist Hoe 140 was tested for its ability to prevent microcirculatory stasis and tissue necrosis in sodium-taurocholate induced acute pancreatitis of the rat.

Fasted Lewis rats were anesthesized with pentobarbital (40mg / kg BW i.p.) and ketamine (10 mg / kg BW i.p.) allowing spontaneous breathing. Arterial and central venous lines were placed measuring MAP, CVP, and art. $SO_2$ continously. The pancreatic duct was canulated. Acridine orange was injected intraveneously to label leucocyte. The in-vivo pancreatic microcirculation was observed with an epiluminescent microscope and recorded on video tape. Hoe 140 (doses: $10^{-9}$, $10^{-8}$, $10^{-7}$ mol / kg BW, n=7-9 per group) or vehicle (0.9% saline, n=10) was injected subcutaneously 30 min prior to the induction of pancreatitis with sodium-taurocholate (4%, 0.4ml i.d.). Interlobular arterial diameter, number of perfused capillaries, capillary flow (CF: semiquantitative scale: 0=stasis to 4=no particle identification), and leucocyte adherence (percentage of vein cross section) were calculated. 3 hours after induction of pancreatitis the animals were sacrifized and histopathologic evaluation of the pancreas was performed using a well defined scoring system. (Schmidt et al. Ann. Surg. 1992, 215: 44-56).

Hoe 140 did not inhibit the mean arterial constriction of 28% observed in controls. The number of perfused capillaries dose-dependently increased in Hoe 140 treated rats compared to controls (Hoe 140 ($10^{-7}$): 76% vs. 0% in controls, $p<0.001$). While the capillary flow was maintained in Hoe 140 ($10^{-7}$) treated rats (CF: 3) total stasis (CF:0) was observed in controls ($p<0.001$). Mean leucocyte adherence was dose-dependently reduced in Hoe 140 treated rats (Hoe 140 ($10^{-7}$): 23% vs. 73% in controls, $p<0.001$). Histopathologic score was dose-dependently reduced by Hoe 140 pretreatment (Hoe 140 ($10^{-7}$): 3.1 points vs. 8.8 points in controls ($p<0.001$).

Pretreatment with the bradykinin-antagonist Hoe 140 reduces leucocyte adherence, maintains microcirculation, and prevents tissue necrosis in sodium-taurocholate induced acute pancreatitis of the rat.

### F119

EXPERIMENTALLY INDUCED ACUTE PANCREATITIS.MODELS FOR SURGICAL RESEARCH.

K.Kalligianni, D.Papadimitriou, E.Kalabalikis, M.Mikoniatis
Pharmacology Dept.,Athens University School of Medicine, Athens , Greece

Acute pancreatitis from the surgical and biological view is a disease with an evolutionary polymorfism in pathogenesis and therapy.The ethical and practical problems of pancreatic investigations in men,led in developing of various experimental animal models of acute pancreatitis. In order to study some surgical problems in the presence of pancreatic disorders,we performed two experimental models with different severity:(a)Acute hemorrhagic-necrotizing pancreatitis induced in the rat by administration of D-L Ethionine intraperitoneally,and (b)Edematous pancreatitis with minor cell necrosis induced in the rat by successive administrations of Cerulein.The protocols were established after multiple biochemical and histological studies of the pancreas,liver and other tissues,especially for the surgical research.They were first applied in the studies of liver regeneration and the results were discussed with perspective to be widely applicable.

### F120

ISOLATION AND ALLO-TRANSPLANTATION OF PIG PANCREATIC ISLETS. INFLUENCE OF PURITY IN THE ENDOCRINE FUNCTION

A. Papalois[1,3], A. Nikolaou[1], K. Nikolaou[1], B. Papalois[1,4], Ch. Tountas[2], B. Karamanos[2], Th.Pataryas[3], P. Peveretos[1], B.Golematis[1]
[1]1st Department of Propaedeutic Surgery, [2]2nd Department of Medicine, [3]Department of Biology, University of Athens, Athens, Greece and [4]Department of Surgery, University of Minnesota (UM), USA

Reports of insulin-independence in patients with type I diabetes mellitus after islet transplantation (Tx) were encouraged transplant centers to follow this treatment as alternative solution to whole-organ grafts. The aim of the present study was to evaluate the influence of islet purity in the endocrine function of the graft under stable immunosuppressive (two or three immunosuppressive drugs) treatment (IT). Totally 24 female swine (18-21 Kg) were used as donors (12) and recipients (12) of islets. For islet isolation total pancreatectomy was performed and the collagenase (Type XI, Sigma C-7657/1 mg/ml) digestion technique of the UM was used with modifications. Recipients were divided in two groups: Group A (n=6): Toxic induction of diabetes followed 1 week after by intraportal islet Tx. IT: Cyclosporin A (CyA) 5 mg/Kg iv before surgery and the 1st post-operative day and 15 mg/Kg per os days 2-30. Azathioprine (Aza) 5 mg/Kg iv 1st day post-operative and 2 mg/Kg per os days 2-30. Group B (n=6): same as in Group A. In IT the protocol was included also Prednizolone (Pr) 2 mg/Kg iv 1st day post-operative and 2 mg/Kg per os days 2-30. Diabetes in all animals was induced using Streptozotocin (STZ-Sigma S-0130). Total dose 65 mg/Kg iv. Intravenous glucose tolerance test (IVGTT) was performed before Tx (before STZ and after induction of diabetes) and in days 14 and 30 post-operative. All animals were survived for 30 days (euthanasia).All the results were evaluated through the purity of isolated islets (>20,000 to >80,000 islets with purity 70-90% in both groups). Functioning grafts (FG) for 30 days were 4/6 in Group A and 5/6 in Group B but the endocrine function is not related to the purity or the number of isolated cells (Group A-FG: 2/4 purity 70% and 2/4 purity 80% and 87%, Group B-FG: 3/5 purity 70-85% and 2/5 purity 82% and 90%). We conclude that purity of isolated cells is necessary to be higher than 70% but is not related directly to the normoglycemic condition of the animal.

### F121

PERITONEAL EXUDATE AS A SOURCE OF ENDOTOXIN IN SEVERE ACUTE PANCREATITIS (SAP).

S Dolan, H Lewis, G Campbell, P Erwin, M McCaigue, MI Halliday, BJ Rowlands. Dept of Surgery, Queen's University Belfast, N Ireland.

INTRODUCTION: Systemic tumour necrosis factor (TNF) has been demonstrated in experimental SAP yet endotoxin, a potent stimulus for TNF activation has not been detected in the vascular compartment. Our aim was to investigate the peritoneal exudate as a potential source of endotoxin and TNF. The role of TNF in disease pathogenesis was further studied by evaluating the effect of Pentoxifylline on survival.

METHODS: Study 1: SAP was induced in 30 male Wistar rats by the retrograde infusion of 0.2 ml of 5% sodium taurocholate at 30 mmHg into the pancreatic duct. Saline infusion was used in 24 control (CON) rats. Animals were allocated to 3 groups and had sampling of peritoneal exudate and venous blood at 2, 3 and 12 hr. CON rats had lavage of the peritoneal cavity with a volume of saline equal to the exudate in SAP rats at that time point. Samples were assayed for endotoxin (Chromogenic LAL) and TNF (ELISA).

Study 2: The effect of intraperitoneal (ip) PTF on survival of SAP rats was assessed, Group A (n=10) were administered PTF 12mg/100g ip, Group B (n=10) the same volume of saline ip.

RESULTS: expressed as median (inter-quartile range) Per = Peritoneal exudate Ven = Venous Endo = endotoxin ND = not detected.

* = p< 0.05 for SAP v CON at same time point (Mann-Whitney U test).

| pg/ml | SAP 2 | CON 2 | SAP 3 | CON 3 | SAP 12 | CON 12 |
|---|---|---|---|---|---|---|
| Endo Per | 17 (114)* | ND | 127 (120)* | ND | 24 (45)* | ND |
| TNF Ven | 66 (30)* | 27 (16) | 35 (26) | 25 (28) | 72 (53)* | 18 (25) |
| TNF Per | 79 (42)* | 33 (22) | 19 (25) | 11 (25) | 80 (47)* | 23 (24) |

All venous samples were negative for endotoxin.

Survival in Group A at 48 hr was 100% and in Group B 40%.

CONCLUSIONS: The exudate seen in this model of SAP is a significant source of endotoxin and TNF. Pentoxifylline administered intraperitoneally has a beneficial effect on survival suggesting a role for TNF in disease pathogenesis and therapeutic potential.

### F122

***PROLIFERATING CELL NUCLEAR ANTIGEN ( PCNA ) IN PERIAMPULLAR TUMORS***

*Funda YILMAZ*, Ahmet ÇOKER, Ali MENTEŞ, Yıldıray YÜZER, Gül YÜCE**

*The HPB Surgery Unit, Department of Pathology*,Aegean University Faculty of Medicine, Izmir, TURKEY*

Pathologic specimens and operative features of fifteen patients with periampullar tumors were reviewed retrospectively. Tumors were originated from papilla vateri in 10 patients, head of the pancreas in 4 patients and the distal common bile duct in one patient. All patients were evaluated in respect to lymph node involvement, tumor size and the proliferating cell nuclear antigen (PCNA). PCNA was assessed due to the presence ratio in 1000 cell counts. The patients were devided into two groups according to the their PCNA ratio levels as lower and higher than 40%. Both groups were similar in regards to their lymph node and tumor status. The patients have been closely followed postoperatively. Although the median follow-up period is 14,5 months, preliminary observations suggest that PCNA levels higher than 40% have a close relationship to early recurrence and survival. It seems possible that patients with high PCNA ratios necessitate neoadjuvant anticancer treatment modalities along with surgical resection.

### F123

DISORDERED CALCIUM HOMEOSTASIS IN EXPERIMENTAL PANCREATITIS

J. B. Ward, R. Sutton, S. A. Jenkins, O. H. Petersen.
Department of Surgery and the Physiological Laboratory, University of Liverpool, England, U. K.

The pathogenesis of acute pancreatitis is poorly understood but disruption of stimulus-secretion coupling may be an important event. As calcium is a key signal in this process we have examined acinar cell cytosolic calcium ($Ca^{2+}{}_i$) signalling in early experimental pancreatitis.

Mice received hourly injections of caerulein (50mg/kg) or saline. Pancreatic tissue was harvested after injections 1, 3, 5 and 7. Acini were isolated by collagenase digestion, loaded with fura-2, and intracellular calcium responses to acetylcholine (100nM ACh) studied using digital-imaging microfluorimetry. Two sets of experiments were performed at each stage.

The number of cells demonstrating a normal oscillatory response of [$Ca^{2+}{}_i$] to 100nM ACh diminished progressively: 20 of 24, 18 of 20, 5 of 14, 2 of 8 after 1, 3, 5 and 7 injections of caerulein respectively ($X^2_{trend}$=15.09, p<0.001). In controls the proportion of cells demonstrating a normal oscillatory response remained high, notably after injections 5 (38 of 43, vs caerulein: $X^2_Y$=13.07, p<0.001) and 7 (29 of 30, vs caerulein: $X^2_Y$=17.14, p<0.001). These results indicate that there is progressive disturbance of acinar cell calcium homeostasis with repeated injections of caerulein. Further work is required to assess the significance of disordered calcium homeostasis in the pathogenesis of acute pancreatitis.

### F124

**INADEQUATE FAT LEVELS OF METRONIDAZOLE USING TWO DIFFERENT TIMES OF INFUSION IN SURGICAL PROPHYLAXIS**

JM Badia, R de la Torre*, R Gaya*, M Farré*, JJ Sancho*, A Sitges-Serra*.
Departments of Surgery and Clinical Pharmacology. Consorci Sanitari de Mataró and Institut Municipal d'Investigació Mèdica*, Barcelona, Spain.

The efficacy of antibiotic prophylaxis depends on appropriate tissue levels of the drug at the time of potential wound contamination. However, antibiotic prophylaxis has a well documented failure rate and previous studies suggest that adequate levels may not be achieved in subcutaneous fat when the drug is administered by intravenous route. *Aims.* To investigate metronidazole concentration in serum, muscle and subcutaneous fat after a single intravenous dose given at two different preoperative intervals.

*Methods.* Twenty-six patients undergoing abdominal wall procedures were divided into Group A, in which 500 mg of metronidazole was administered two hours before surgery, and Group B in which the drug was administered during induction of anaesthesia.

*Results.* Plasma level at the beginning of the procedure was significantly lower in group A, (mean with 95% confidence intervals) 7.3 (5.7 - 8.9) µg/mL than in group B, 12.3 (8.9 - 15.7) µg/mL (P = 0.01) although in both cases were above the MIC for 90 % of *B. fragilis.* No other statistical difference between the two groups was found. Metronidazole achieved comparable therapeutic concentrations in plasma and muscle in both groups at the end of surgery. However, both groups showed non therapeutic concentrations of metronidazole in subcutaneous fat: 0.9 (0.6 - 1.2) µg/mg in Group A and 1.2 (0.7 - 1.7) µg/mg in Group B at the beginning of operation and 1.2 (0.8 - 1.6) µg/mg and 1.5 (0.9 - 2.1) µg/mg, respectively, at the end of the procedure.

*Conclusions.* Infusion of metronidazole two hours before surgery or during induction of anesthesia allowed adequate plasma and muscle levels but failed to achieve therapeutic levels in the subcutaneous fat tissue.

This study was supported with the grant FIS 90/0673 of the Ministry of Health of Spain.

### F125

**OMEPRAZOLE PROVIDES PROTECTION AGAINST CERULEIN INDUCED PANCREATITIS IN RATS**

A.L. Montagnini, M.S. Kubrusly, K.R.M. Leite, A.M.M. Coelho, N.A.T. Molan, JEM da Cunha, M.C.C. Machado, H.W. Pinotti
São Paulo University Medicine School - São Paulo - Brazil

It has been proposed that trypsinogen activation inside pancreatic acinar cell is the triggering event for acute pancreatitis. Recently we showed that administration of omeprazole to rats decreases pancreatic content of trypsinogen. This study was designed to determinate if previous administration of omeprazole to rats can alter the intensity of acute pancreatitis induced by cerulein.

Fifty eight male Wistar rats (180 - 220 gr.) were divided in 4 groups. Control group (G I) received 3 doses of 0,5 ml saline solution intraduodenally at 24 hs intervals and 2 i.v. injections (0,5ml) of saline 2 Hs after last saline i.d. injection. Omeprazole group (G II) received 3 doses of omeprazole (5 μmol/kg) intraduodenally at 24 Hs intervals and 2 i.v. injections (0,5ml) of saline. Pancreatitis group (G III) was treated like G I but received 2 i.v. injections of cerulein (20μ/kg) instead of saline. Omeprazole plus Pancreatitis group (G IV) was treated like G II but received 2 i.v. injections of cerulein (20μ/kg) instead of saline. All animals were killed 3 hs after last saline or cerulein I.V. injection. Blood was collect for amylase determination and pancreas ware rapidly removed for water, trypsinogen, catepsin B determination and prepared for histopathologic scoring.

RESULTS:

| | G I | G II | G III | G IV |
|---|---|---|---|---|
| **AMYLASE** | 7.6 ± 0.3 ◊ | 5.88 ± 0.27 ◊ | 15.7 ± 1.1 * | 10.1 ± 0.7 * |
| **% of WATER** | 71.6 ± 1.4 ◊ | 69.4 ± 2.9 ◊ | 81.7 ± 1.0 * | 75.91 ± 1.2* |
| **TRYPSINOGEN** | 13.3 ± 1.2 ◊ | 9.27 ± 0.7 ◊ | 12.4 ± 0.8 # | 11.5 ± 0.8 # |
| **CATEPSIN B** | 2.06 ± 0.1 | 1.6 ± 0.3 | 8.0 ± 1.6 # | 3.42 ± 0.4 # |
| **EDEMA** | 0.9 ± 0.3 | 1.0 ± 0.3 | 2.7 ± 0.6 * | 2.0 ± 0.5 * |
| **NECROSIS** | 0 | 0 | 0.9 ± 0.3 * | 0.4 ± 0.7 * |
| **INFLAMMATION** | 1.0 ± 0.6 | 0.3 ± 0.2 | 2.8 ± 0.8 * | 1.5 ± 1.3 * |

results: mean ± s.e.m same symbol in same line *, ◊ = $p < 0,05$ , # = $p < 0,001$

CONCLUSION: Omeprazole administration to rats decreased pancreatic trypsinogen content and intensity of acute pancreatitis induced by cerulein.
We believe that CCK may play a role in this findings.

### F126

CALCIUM-REGULATING SYSTEMS AND EXOCRINE PANCREATIC SECRETION IN CHRIONIC PANCREATITIS

N.Budagov, A.Yagoda, and R.Rekvava
Department of Internal Diseases No 1, Medical Academy, Stavropol, Russia

Impaired calcium metabolism is thought to play a substantial role in pancreatic secretion disturbances and can affect the course of the chronic pancreatitis (CP).

Levels of parathormone (PTH), calcitonine (CT), and 25-hydroxycholecalciferole (25-HCC) were studied in 35 CP patients. The results were compared to the blood levels of trypsin and elastase, and duodenal contents of L-amylase, lypase, and bicarbonates. 12 healthy volunteers served as controls.

In relapse of the disease, PTH and elastase contents were found enhanced, those of CT, 25-HCC and trypsin did not differ from normal values, while output of enzymes and bicarbonates was significantly decreased. In cases of severe relapse, enhanced levels of PTH, 25-HCC, trypsin, and elastase, along with decreased contents of CT were found. Straight correlation between levels of 25-HCC and trypsin ($r = +0.84$), PTH and amylasuria ($r = +0.38$), and reverse correlation between CT levels and blood amylase ($r = -0.36$), and lipase secretion ($r = -0.56$) were detected. Intravenous calcium gluconate led to sharp decrease in PTH levels in CP patients, while in control group it resulted in significant increase of serum CT.

The results point at possible involvement of the calcuim-regulating system in the performance of the pancreatic acinar cells. Overproduction of PTH, along with low reactivity of thyroid C-cells and small blood levels of 25-HCC (increase in the metabolically active fraction of vitamine D3) is characteristic of the impaired performance of the competitive calcium regulation system. PTH and 25-HCC could also participate in the «enzyme deviation» phenomenon, while serum CT could play a protective role.

### F127

IMMUNCYTOCHEMICAL DETECTION OF DISSEMINATED EPITHELIAL TUMOR CELLS IN BONE MARROW OF PATIENTS WITH ADENOCARCINOMA OF THE PANCREAS

St. Thorban[1], J.D.Roder[1], K. Pantel[2], G. Riethmüller[2], J.R. Siewert[1]

[1]Department of Surgery, Technische Universität München, Germany.

[2]Institute of Immunology, Ludwig-Maximilians-Universität, München, Germany.

Minimal residual disease as the major cause of tumor recurrence in patients with resectable adenocarcinoma of the pancreas is frequently missed by current non-invasive tumor staging.

In the present study, epithelial cells in the bone marrow of 25 patients with pancreatic carcinoma were identified immunocytochemically with monoclonal antibodies (mAbs) CK2, KL1 and A45-B/B3 directed to epithelial cytokeratins (CK), using the alkaline phosphatase anti-alkaline phosphatase method. The specifity of these mAbs was demonstrated by negative staining of marrow from 25 non-carcinoma control patients.

Analysis of bone marrow aspirates from cancer patients revealed CK-positive cells in 7 (58.3%) of 12 patients treated with curative intent and 9 (69.2%) of 13 patients with extended disease. After a median follow up of 7.9 months (3-24 months), the occurrence of tumor relapse and the survival in patients who underwent complete surgical resection was significantly associated with CK-positivity in bone marrow ($p < 0.05$).

In conclusion, anti-cytokeratin mAbs are reliable probes for the immunocytochemical detection of early disseminated panreatic cancer cells in bone marrow. The presence of these cells might be indicative of an increased disseminative capability of the primary tumor with poor survival.

### F128

AMPULLOMA (PAPILLAR and PERIAMPOLLARE CARCINOMA).ENDOSCOPIC DIAGNOSIS and SURGERY TREATMENT.

M.Vajo,V.Ambu,S.Arena,G.Campanella,F.Cavuoto,A.Cereser,M.Dugo,M.Fraccalini,C.Pallante*,S.Ponzio,R.Tramontano.

Department of Surgery,Rivoli Hospital,Torino,Italy.

*Endoscopic Service,Rivoli Hospital,Torino,Italy.

Of about 18% of cases of malignant biliary obstruction in our case histories,it present its self as:

a) 2 cases- with a rough out growth,which is also fragile and jelly-like.

b) 2 cases- hard mass,ulcerated on the level of the papilla.

c) 1 case- enlarged papilla covered with mucosa of normal aspect.

Differential diagnosis: clogged stone, edema of the papilla, deformation of the choledoch duct.

Diagnosis: sphincterotomy+biopsy on the edge of the wound(75% of sensibility of ours case histories).

The tumour is extremely crumbly and will brake just if is touched with the probe and consequently you have to insert a endoprosthesis without preliminary papillotomy (1 case).

All the patients still alive after two years of the operation; the survival aspectancy,after five years, is of about 30-40% of the case histories.

The surgical technique used: DCP according to Trasverso-Longmire with nose-pancreatic drain. In all cases the patients have had regular post-operation without infections, pancreatitis,biliary fistula.

### F129

**TREATMENT OF ZOLLINGER-ELLISON SYNDROME. 25-YEARS EXPERIENCE**

C. Pasquali, C. Sperti, ^ G. Liessi, § V. Valmachino, S. Pedrazzoli.
Semeiotica Chirurgica - University of Padua, ^ Radiology and § 2nd Dept. of Medicine - Castelfranco V. Hospital, Italy.

From 1970 to 1994, 53 patients with Zollinger-Ellison syndrome (ZES) were observed. MEN type 1 syndrome was found in 24.5 % of the patients. Only 28 % of the patients had no gastric surgery before the diagnosis of ZES ; the other patients averaging 2.8 gastric operations each before diagnosis. Seven cases (13 %) had distant metastases at time of diagnosis and 2 more cases developed liver metastases during the follow up (15 and 21 years later). Thirty patients (57 %) underwent surgery; 11 had only a "palliative" gastrectomy and 19 had a laparotomy with resective purposes. Out of this 19 patients 13 had a previous pancreatic venous sampling which in 8 cases showed a single source of gastrin hypersecretion; all these 8 patients became normogastrinemic after resection of the tumor. A total of 11/30 surgically treated patients (37 %) had a gastrin fall to normal after surgery (9 of them with a follow up of 5 - 17 years), including one MEN 1 case and one with liver tumor. Two of these "cured" patients had a late recurrence 5 and 14 years later. Out of 30 patients who underwent surgery 23 % had an emergency gastrectomy ( 2 associated with tumor resection) for complication due to ulcer disease and 8 /19 of the patients who had resective surgery had also a gastrectomy associated. Morbidity was 37 % and mortality 20 % (3/6 in emergency ; 5 before 1979) . Twenty-seven patients initially had antisecretory drugs therapy, but 3 escaped to H-2 blockers and needed emergency surgery (after 3 mo.-3 yrs.) while 9 required omeprazole for long term - failure ( 2-14 yrs.) of H-2 antagonist. Two medically treated patients had their primary tumor detected 7 and 14 year after the diagnosis; the first one had than the tumor resected and became normogastrinemic. Five out of nine patients with liver metastases survived more of 5 years after detection of the secondary.
Long-term palliation or "cure" may be achieved by tumor resection in ZES (37 % in our series) ; however life-long follow-up is mandatory for late recurrencies (> 5 yrs.). Re-evaluation of patients without positive imaging is advisable for possible reversion from medical therapy to resective surgery .
Study supported by Italian Ministry of University (MURST) 60%.

### F130

**LEAKAGE OF THE PANCREATIC ANASTOMOSIS IN 224 WHIPPLE'S RESECTIONS. INCIDENCE AND PREVENTION USING SYSTEMIC SOMATOSTATIN OR FIBRIN GLUE FOR TEMPORARY OCCLUSION OF THE PANCREATIC DUCT.**

TM van Gulik, EEE Gabeler, MI van Berge Henegouwen, PCM Verbeek, LT de Wit, H Obertop, DJ Gouma. Dept. of Surgery, Academic Medical Center, Amsterdam, The Netherlands.

Leakage of the pancreatic anastomosis is a potentially lethal complication after Whipple's resection. We evaluated the incidence of pancreatic anastomotic leakage (PA-leakage) in our overall series of 224 Whipple's resections performed between 1983-july1994. In one period (1991-May92), pancreatic exocrine secretion was suppressed by giving somatostatin (SMS, 250mcg/h, iv, during 6 perioperative days) as part of a randomized, double blind, placebo controlled study. In another period (June92-July94), the exocrine secretion was temporarily blocked by occluding the pancreatic duct with fibrin glue (PDO) when the pancreas remnant was soft and found at risk for leakage. 20.000 IU Aprotinin was added to the fibrin glue (Tissucol[R]) before use. ***Results:*** Clinically evident PA-leakage occurred in 24/224 (9.3%) of pats.(see table). Seven pats. (29%) died of this complication, constituting a high proportion of the total mortality (9 pats.=4%) in this group of patients. Not one case of PA-leakage was not noted in the 8 pats. treated with SMS or in the 12 pats. treated with PDO. The occurrence of local complications (abscess, leakage, hemorrhage, pancreatitis, fistula) was comparable in all patient categories.

| | PA-leakage | Mortality | Local complic. |
|---|---|---|---|
| **Period 1983-May1992** | | | |
| **8 pats. SMS** | 0 | 0 | 2 (25%) |
| **7 pats. placebo** | 1 | 1 | 3 (43%) |
| **148 pats. (reference)** | 15 | 5 | 53 (36%) |
| **Period June92- July94** | | | |
| **12 pats. PDO** | 0 | 0 | 3 (25%) |
| **49 pats. (reference)** | 8 | 1 | 19 (36%) |
| **Total group (224 pats.)** | 24 (9.3%) | 7 | 80 (36%) |

***Conclusion:*** The overall incidence of PA-leakage in this series of Whipple's resections was 9.3% and this complication was associated with a high mortality (29%). SMS and PDO seemed both effective in preventing PA-leakage.

### F131

**CYSTIC TUMORS OF THE PANCREAS - WHAT'S NEW IN DIAGNOSIS AND TREATMENT: A MULTICENTER STUDY OF 52 CASES**

G. SPILIOPOULOS *, M.A.C. MACHADO *, P. VOLPE **, A.L. MONTAGNINI**, B. CHARETON*, T. BACCHELLA**, J.E.M. CUNHA**, J.P. CAMPION*, M.C.C. MACHADO**, B. LAUNOIS*.
Department of Surgery and Transplantation Unit.
* - University of Rennes, France. ** - University of São Paulo, Brazil.

Cystic neoplasms are an uncommon group among pancreatic tumors. These lesions are seen more frequently in recent surgical practice, probably because of advances in diagnostic and surgical techniques. The aim of this study is to describe the diagnostic features and therapeutical options in the management of cystic tumor of the pancreas. We report our findings in 52 patients with cystic tumors of the pancreas over a ten-year period. Forty four patients were women and eight were men. The mean age of patients was 55.4 years (range, 21 to 81 years).

Mild abdominal pain was the main symptom in 70 % of patients. Weight loss was found in 30% of the patients and abdominal mass in 25%. The lesion were incidental finding in 10% of patients. CT scan provided the diagnosis of cystic tumor in 94% of patients while ultrasonography provided the same diagnosis in 78% of patients. All patients underwent surgical treatment. The pathological diagnosis was: twenty-one patients with mucinous cystadenoma (40.4%), twenty patients with serous cystadenoma (38.5%), eight patients with mucinous cystadenocarcinoma (15.4%) and three patients with cystadenoma unclassified. The mean size of the cysts was 6.6 cm (range, 2.3 to 13 cm). Twenty-one cystic neoplasms were located in the tail (40.4%), sixteen in the body (30.8%) and fifteen in the head of the pancreas. Thirty-eight patients (73.1%) underwent complete resection of their pancreatic tumor, twenty-seven by distal pancreatectomy, five by pancreaticoduodenectomy, five by enucleation and one by total pancreatectomy. Nine patients underwent biopsy of the mass only, one had local excision of the cystadenoma from the head of pancreas and four had cystjejunostomy. There was no operative mortality. Five of six patients with cystadenocarcinoma ultimately died of the disease while one patient with extended resection is still alive 2 years after surgery without recurrence of the tumor. All patients with cystadenoma (mucinous or serous type) that underwent complete resection are alive or died from other causes.

Only complete resection of the cystic tumors of the pancreas provides certain pathological diagnosis, the best chance of cure and may remove the risk of malign transformation of the cystadenomas, particularly of the mucinous type, with minimum operative risk.

### F132

**FRANTZ TUMOR (CYSTIC AND PAPILLARY EPITHELIAL NEOPLASM OF THE PANCREAS): A STUDY OF 4 CASES**

M.C.C. MACHADO, J.E.M. CUNHA, T. BACCHELLA, J. JUKEMURA, S. PENTEADO, M.A.C. MACHADO.
Department of Surgery - University of São Paulo, Brazil.

Since the first description by Frantz in 1959, there are few reports about this pancreatic tumor. They constitute rare tumors with prevalence in young women and, although malignant, they have good prognosis with benign evolution despite the fact that they present histologically features of aggressive tumor. This is an important fact due to the possibility of cure if completely resected.

We present our findings in four patients with Frantz tumor. Three patients were women ad one was man (range, 14 to 39 years). All patients underwent complete resection of the tumor. In three patients, invasion of the portal vein was present and segmentary resection of this vessel was performed. The outcome was favorable in three cases. One patient is alive five years after the pancreatic resection. One patient was operated on 15 years before with the initial diagnosis of unresectable cancer underwent resection but died two years later with hepatic metastases.

The majority of patients described in the international literature comes from Japan. There are only two cases of Frantz tumor occurring in males, including one of this series. The jaundice is rare even with large tumors. Ultrasonography and computerized tomography demonstrate encapsulated, spherical, hypervascularizated and solid-cystic tumors.

The importance of this rare tumor stays in the fact that it can be cured, if correctly recognized, with total resection.

### F133

**INTRA-DUCTAL PAPILLARY CARCINOMA OF THE PANCREAS - A NEW ENTITY: A STUDY OF 3 CASES**

M.C.C. MACHADO, E.E. ABDO, M.A.C. MACHADO, A.L. MONTAGNINI, P. VOLPE, M.C. ZERBINI, J. JUKEMURA, S. PENTEADO, T. BACCHELLA, J.E.M. CUNHA, H.W. PINOTTI.
Department of Surgery - University of São Paulo, Brazil.

Intra-ductal papillary carcinoma of the pancreas* represents a new and extremely rare pathological entity. The exact histological identification is very important and includes intra-ductal papilloma, *in situ* and diffuse intra-ductal papillary carcinoma. The first being the premalignant condition for the other two histological types. Although these tumors have been described with different denominations, clinico-pathological studies demonstrated that they constitute an unique pathological entity. Because of their favorable prognosis and absence of peripancreatic infiltration and metastases, they are considered a low-grade malignancy.

We report our findings in three patients with intra-ductal papillary carcinoma of the pancreas surgically treated. The first patient presented diffuse papillary carcinoma associated with intra-ductal papilloma suggesting that the latter is a premalignant condition. This patient underwent total pancreatectomy. The second presented intra-ductal papillary carcinoma in the body of the pancreas and underwent distal pancreatectomy. The third patient presented the mucinous type of the intra-ductal papillary carcinoma of the pancreas located in the head of the pancreas. A pancreaticoduodenectomy was then performed.

A clinico-pathological analysis of these patients, the difficulties in the preoperative diagnosis and surgical options are emphasized.

### F134

CARCINOID TUMOURS OF THE PANCREAS.

I.Buriev, A.Vihorev, V.Tsvirkoun, T.Savvina.
A.V.Vishnevsky Institute of Surgery,Moscow,Russia.

This study is about the surgical treatment of carcinoid tumours of the pancreas. It's involved 23 patients with carcinoid tumours of the pancreas and 4 with duodenal carcinoid tumour. Involvement in the carcinoid disease of the pancreas requiring pancreatic resection of various extents. In 18 patients signs of malignant progression of the disease were observed. 16 patients had, earlier on, undergone various forms of non-effective surgical procedures. The age of the patients ranged between 18 and 69 years. In 8 patients a symptomless progression of disease was observed, while 9 patients developed complications: jaundice (5), duodenal obstruction (4). Information obtained from US and CT scans in combination with the clinical presentation permitted the suspicion of carcinoid tumour in 81,5% of cases. Subcutaneouse fine-needle aspiration biopsy revealed signs of carcinoid tumour in 16 patients. 8 patients with distal localisation of tumour underwent distal pancreatectomy, 1 patient underwent total pancreatoduodenectomy while 15 underwent pancreatoduodenal resection - in these the tumours were located in the head of the pancreas (amongst these 5 patients had a gastric-spearing procedure). In 4 patients the tumours were excised by enucleation. The postoperative mortality was 11%. During a follow-up period of up to 5 years tumour recurrence was observed in only one patient. We conclude that radical surgical treatment of carcinoid tumours of the pancreas provides good long-term survival.

### F135

PERCUTANEOUS FINE-NEEDLE ASPIRATION BIOPSY OF THE PANCREAS

S.Linder[1], M.Blåsjö[2], P.Sundelin[2], A von Rosen[3]. Departments of Surgery[1] and Pathology[2], Stockholm Söder Hospital, Stockholm, Department of Surgery[3], Karolinska Hospital, Stockholm, Sweden.

The value of percutaneous fine-needle aspiration biopsy (FNAB) was assessed in the diagnosis for pancreatic carcinoma. Threehundred- thirtyfour consecutive patients were subjected to FNAB of the pancreas during a 13 year period. A 22-gauge needle was used, mainly guided by percutaneous transhepatic cholangiography.
The cytologic preparations were air-dried and stained by the May-Grünwald-Giemsa method. Twohundred-seventy patients were proved to have pancreatic carcinoma, 187 of which were verified cytologically.
There was no false positive cytologic interpretation. Thus, sensitivity, specificity, and overall accuracy was 69%, 100%, and 75%, respectively.
The percentage of positive cytologic samples did not differ between 46 patients operated with pancreaticoduodenal resection and those with other treatments. Positive cytology tended to correlate to tumor size ($p=0.008$), but not to grade or tumor stage.
In univariate analysis a borderline correlation was found between positive cytology and short survival time, which may reflect that advanced tumors are more easily hit. In a multivariate analysis only type of therapy (tumor resection vs. other treatment) ($p<0.001$) and presence of jaundice ($p<0.01$) had influence on survival time. No complications to FNAB were encountered. It was concluded that FNAB is a safe and accurate method in the diagnosis of pancreatic carcinoma. In resectable disease, however, the use of FNAB may be controversial due to a potential risk of seeding, and negative cytology will not change the intention of curative surgery if the suspicion of carcinoma is high. In non-resectable tumors the cytologic material obtained at FNAB may also be used to assess various tumor characteristics e.g. DNA ploidy, morphometric variables. Such data may perhaps serve as a bases for stratification of non-surgical therapy.

### F136

IL-6 RELEASE FROM PERIPHERAL BLOOD MONONUCLEAR CELLS IN PATIENTS WITH ACUTE PANCREATITIS CAN BE DOWN REGULATED BY IL-4

A.C. de Beaux, J.A. Ross, K.C.H. Fearon, D.C. Carter
University Department of Surgery, Royal Infirmary, Edinburgh, Scotland. EH3 9YW

Leucocyte activation and pro-inflammatory cytokine release is thought to contribute to the systemic sequelae of acute pancreatitis. It has been demonstrated that IL-6 release is up-regulated from peripheral blood mononuclear cells (PBMCs) in patients with acute pancreatitis on the first day of admission. Leucocyte resistance to normal down-regulatory signals such as IL-4 (a T lymphocyte cytokine) may contribute to this inflammatory response. To examine this hypothesis, PBMCs were isolated from 6 healthy controls and during the first day of admission in 6 patients with acute pancreatitis and cultured in the absence or presence of lipopolysaccharide (LPS) (5 $\mu$g/ml) and human recombinant IL-4 (dose range 0-50 ng/ml). IL-6 was measured in the cell supernatant after 24 hour incubation by enzyme-linked immunosorbant assay. The results are shown in the table and expressed as a percentage of IL-6 release in the absence of IL-4.

| IL-4 | Spontaneous IL-6 release | | LPS-stimulated IL-6 release | |
|---|---|---|---|---|
| ng/ml | pancreatitis mean±SEM | controls mean±SEM | pancreatitis mean±SEM | controls mean±SEM |
| 0 | 100 (0) | 100 (0) | 100 (0) | 100 (0) |
| 0.05 | 93.3 (12.8) | 83.4 (6.7) | 92.6 (4.9) | 86.5 (6.1) |
| 0.5 | 45.3 (12.9) | 47.2 (6.3) | 59.6 (12.2) | 59.5 (4.6) |
| 5 | 8.7 (3.0) | 13.4 (1.9) | 18.1 (7.9) | 15.8 (3.0) |
| 50 | 5.2 (1.9) | 7.3 (1.3) | 10.1 (3.2) | 14.7 (3.2) |

IL-4 attenuated PBMC IL-6 release in a dose dependent manner in both the patient and control groups. Furthermore, the IL-4 dose response on IL-6 release was not significantly different in the patient group compared with the control group ($p>0.1$; analysis of covariance). This observation may provide a method of biological response modification in acute pancreatitis.

### F137

VALUE OF PROGNOSTIC INDEX IN ACUTE PANCREATITIS

O.Demircan, Ö.Yağmur, H.Sönmez, A.Alparslan, E.U.Erkoçak, Ö.Alabaz, F.C.Özkan

University of Çukurova, School of Medicine, General Surgery Department, Adana-TÜRKİYE

From 1989 to 1993, 42 patients with acute pancreatitis were treated in Çukurova University School of Medicine, General Surgery Department.
In this study results were analyzed according to age, sex, etiologic agent, laboratory and radiologic evaluation, morbidity and mortality retrospectively. Prognosis and severity of acute pancreatitis were investigated with multiple parameters prognostic systems (Ranson, Osborne, Blamey, Bank, Damman) and Baltazar's computer tomography (CT) classification. Of the 42 patients, 21 (50%) were male and 21 (50%) were female and the mean age was 51 years. The most frequent cause of acute pancreatitis was gallstone (62%), followed idiopathic (19%), alcohol (14.5%) and trauma (4.5%). Overall mortality and morbidity rates were; 14.2% (6 patients) and 28.5% (12 patients) sequentially. A highly significant relationship was found between severity of acute pancreatitis and prognostic score systems. A significant increase in mortality and morbidity were seen in the patients who classified D and E level according to Baltazar's CT classification.
It is concluded that prognostic score systems can be used in combination with the CT classification score to estimate better the outcome of acute pancreatitis patients.

### F138

NUTRITIONAL SUPPORT IN SEVERE ACUTE PANCREATITIS

M. Digalakis, G. Dukas, K. Kilakos, A. Rigas
1st Department of Surgery,Tzanion State Hospital of Piraeus.

Last 8 years 87 patients were admitted to our surgical department with a diagnosis of acute pancreatitis. Gallstones were diagnosed to be the aetiological factor in 70 patients, alcohol in 9 patients, lipid abnormalities in 3 patients and there were 5 postoperative acute attacks of the disease. We used the Ranson's multifactor prognostic system to evaluate the severity of the disease.
Thus, acute pancreatitis was classified as severe if 2 or more factors were positive on admission or during the first 48 hours.

Thirty five patients (40,2%)had a mild attack and they received only conventional intensive therapy. Fifty two (59,7%)patients developed severe illness and they were begun on conventional therapy plus total parenteral nutrition(TPN). Caloric requirements were estimated by the Harris-Bebedict equation.

Three patients with pre-existing hyperlipidemia did not receive lipid emulsions as caloric source. Glycose intolerance requring exogenous insulin was seen in 23 patients. After 15 days of TPN therapy, 50 patients (96,1%) achieved positive nitrogen balance and showed improvement in nutritional indices. There were 4 hospital deaths(4,5%) among the 87 patients. One patient with mild pancreatitis and no TPN therapy died due to pulmonary embolism. Three patients (3,4%) with severe acute pancreatitis and TPN therapy died due to sepsis and multiple organ failure.

We believe that patients with severe acute pancreatitis represent a malnourished population and TPN may be an efficient treatment for their nutritional depletion. Our results indicate that lipid emulsions were well tolerated.

### F139

TIME COURSE OF BACTERIAL INFECTION OF THE PANCREAS AND ITS RELATION TO DESEASE SEVERITY

A.Agorogiannis,E. Naoum and C. Sarros.
Surgical Unit.District General Hospital Of Larisa-Greece.

The aim of thiw study is to confirm a finding which suggest s that bacterial infectionof the pancreas requires more severe tissue injury.For the last 16 years we operated on 3,900 patients with benign deseases of the biliary system. 388 patients werte operated for acute pancreatitis (AP).47 patients had the severe hemorrhagic and necrotic AP In the 341 cases of AP the infection was mild,mostly edematous and the bacterial contamination was foundin 18% of the casesThe clinical condition of the patients was mild without sings and symptoms of sepsis.All the 47 cases had bacterial infection,with clinical sings and symptoms of sepsis.The type and incidence of bacteria identified in pasncreatic tissue of patients with necrotizing pancreatitis are:Gam negative bacilli,Gram positive cocci and anaerobic bacteria ,with more common in each category E.Coli,Enterococcus sp,and Bacteroides spp.In 341 patients with edematous mild AP the postoperative mortality was 0%,and the mortality rate for the 47 gravely infected patients was 18%.
Conclusions:As advances in clinical care have reduced early cardiorespiratory complications of AP,infection has emerged as the major cause of morbidity and mortality of the desease.Local and systemic septic complications increasw with the amound of necrosis.The precice route by which the microorganisms reach the pancreas is not completely understood Transmural migration of intestinal bacteria in the major route of bacterial infection of pancreatic necrosis in AP is the main cause of sepsis.These needs the proper recognition and the proper treatment in ICUs.Operation is nessesary after 18-20 days,when absces formation is diagnosed which at this time has made thic walls and can be drainade more safely.In parallel the proper antibiotics are administered ,after cultures of the special bacteria is available Caltures can be done by percutasneous absorpsion of material of necrotic tissue with the guidance of CT.Also enhanced CT finds out the exact location of necrosis andf pus.

### F140

NECROTIZING PANCREATITIS: EXTENSIVE LAVAGE OR LOCAL LAVAGE AFTER SURGERY.

S.Papavramidis, O.Gamvros, I.Dokmetzioglou, N.Deligiannidis, A.P.Aidonopoulos
3rd Dept. of Surgery, A.H.E.P.A. Hospital, Aristotelian University of Thessaloniki

Between January 1981 and December 1994, 37 consecutive "severely ill" patients with necrotizing pancreatitis underwent a standard surgical treatment including necrosectomy, cholecystectomy or choledochostomy and massive intraoperative lavage in an effort to manage their disease. Twenty of them were male and 17 female, aged between 19 and 88 (mean 53) years. According to additional postoperative treatment patients were classified in two groups. In group A were included 22 consecutive patients who underwent postoperative extensive lavage (peritoneal and retroperitoneal) with Ringer solution via 4 draining Argyle No 32 tubes for 3-4 days after surgery. Group B consisted of 17 consecutive patients who were managed by postoperative lesser sac lavage (local lavage) via 2 draining tubes. Operation was performed at a mean of 9,3 days (ranged from 6 hours to 32 days) after the onset of symptoms (group A) and 2,9 days (ranged from 3 hours to 15 days) for the patients of group B, because of non-response to conservative treatment or acute abdomen formation. Fifteen of the patients of group A (68%) and 10 of group B (66,6%) developed simple or multiple organ failure and hypovolemic or septic shock. Reoperations - one or more - were needed in 6 patients (27,2%) at group A and 3 (20%) at group B. Nine patients of this series (24,3%) died because of noncontrolled multiple organ failure. The mortality rate was 18,2% for the patients of group A and 33,3% of group B. It is concluded that postoperative lavage (especially extensive one) is of good value in the treatment of necrotizing pancreatitis.

### F141

### ENDOTOXAEMIA AND SEPSIS IN ACUTE PANCREATITIS

G.Perpyrakis, K.Politi, M.Velegrakis, S.Kandylakis, K.Zervos.
Surgical Department, General Hospital of Rethymnon, Greece.

This study was planned to investigate the occurence of endotoxaemia in acute pancreatitis (A.P) and the effects of endotoxaemia on the course of the disease.

During a 9 years period, we studied 196 patientw with A.P. Besides routine investigations, we screened blood samples in order to detect endotoxins with the Limulus Amoebocyte Lysate test (LAL-test) on the 1st, 2nd and 7th day of illness, along with blood cultures.Patients with more than 3 positive signs according to Ranson or Imrie and one or more positive parameters of the Agarwal system were considered to have serious disease.With these criteria 75 patients (38,25%) were severely ill (Group A) and 121 (61,75%) were moderately ill (Group B). Endotoxin was found in the plasma of 46 patients (61,3%) of Group A, and 4 of these patients had positive blood cultures; 9 patients in Group B (7,4%) were positive for endotoxin and none had positive blood cultures. Of the 46 patients in Group A, 32 (69,5%) developed septic complications, compared to only one patient of 9 in Group B (11%).

Although endotoxaemia is not associated with the occurence of septic complications in all cases, our results show that the detection of endotoxins in the plasma of patients with A.P, evaluated in combination with the signs of Ranson, Imrie and Agarwal as well as the C.T. results, may significantly assist in detecting the cases most susceptible to septic complications.

### F142

### RECONSTRUCTION AFTER DISCONNECTION OF CHOLEDOCHODUODENAL JUNCTION DURING PARTIAL GASTRECTOMY : TWO CASES REPORT

Zoran Matović,Slobodan M.Janković
Surgical clinic,Clinical-Hospital Centre, Kragujevac,Serbia,FR Yugoslavia

High choledochoduodenal junction could be anatomical variety, but it is more often caused by chronic duodenal ulcer when abundant fibrosis pulls this junction upward("crawling of papilla"). During partiall gastrectomy performed by inexperienced surgeon choledochoduodenal junction could be disconnected in an attempt to dissect duodenal stump. We present two cases of choledochoduodenal disconnection during partial gastrectomies because of chronic duodenal ulcer. In both cases reconstruction was performed in the same operation. First we formed Roux-en-Y limb of jejunum 80 cm long,about 30 cm far from ligament of Treitz. We closed duodenal stump blindly, and we made termino-terminal anastomosis between head of the pancreas and the Roux-en-Y limb of jejunum.The choledochus was transected supraduodenally,distal end ligated and proximal end anastomosed termino-laterally with Roux-en-Y limb.Distal two-thirds of stomach were resected,and proximal stump anastomosed termino-laterally with distal part of Roux-en-Y limb.Both patients recovered, and were alive and well after 5years.This type of reconstruction after choledochoduodenal disconnection proved to be successful in our cases,but it is better to prevent such accidents by preoperative simultaneous contrast X-ray studies of stomach,duodenum and biliopancreatic tree.

### F143

### OUTCOME AFTER ACUTE PANCREATITIS

Wig JD, Gupta NM, Kochhar R, Suresh K.
Department of Surgery and Gastroenterology, Postgraduate Institute of Medical Education and Research,Chandigarh-160012,India.

There are conflicting reports regarding the outcome after an attack of acute pancreatitis. This study was designed to evaluate the exocrine, endocrine and morphological abnormalities after acute pancreatitis. Ten patients were studied at periods of 1 to 8 years after a proven episode of acute pancreatitis. They underwent oral glucose tolerance test, Lundh's test, faecal fat estimation and ERCP. One patient developed diabetes mellitus and another patient had steatorrhoea. Nine patient in whom a Lundh test was performed had normal trypic activity. Six patients had morphological abnormalities on ERCP - dilated duct (1), blocked duct (2), nonvisualization of pancreatic duct (1), abnormal side branches (1), and pseudocyst (1). US and CT scan showed a pseudocyst (1) and a pseudocyst with changes of chronic pancreatitis in one patient. CT scan showed atrophy of body and tail of pancreas in one patient. These observations indicate that morphological changes predominate and are probably early changes in the spectrum of chronic pancreatitis.

### F145

### DIAGNOSTIC AND THERAPEUTIC STRATEGIES IN THE MANAGEMENT OF PANCREATIC DUCT HAEMORRHAGE

PJ Gallagher, PC Bornman, JEJ Krige, G McLauchlan, S Beningfield*, J Terblanche.
*Surgical Gastroenterology Groote Schuur Hospital and Departments of Surgery and Radiology*, University of Cape Town, Cape Town, SOUTH AFRICA.*

Haemorrhage via the pancreatic duct (PDH) is a rare cause of upper GI bleeding and is often a diagnostic dilemma. We analysed our experience with 10 patients treated during a 12 year period.
Of the 8 men and 2 women (mean age 44, range 34-62) 8 had previous admissions (mean 3, range 1-5) and 3 surgery for unexplained bleeding. All 10 patients had a history of heavy alcohol intake and presented with major upper GI bleeding requiring a median of 8 units (range 2-40) of blood. 9 had upper abdominal pain and previous admissions for pancreatitis. All had previous gastroduodenal endoscopy, median 4 (range 1-9) which was diagnostic in only 1. ERCP showed bleeding from the pancreatic duct in 7 of 8 patients. Of 9 coeliac angiograms, aneurysms were demonstrated in 7, involving splenic artery in 4 (tail 3, head 1) gastroduodenal artery in 2 and pancreaticoduodenal in 1. Two of 4 selective embolisations were successful. 6 patients had distal pancreatectomy, 1 had ligation of the gastroduodenal artery and 1 had a total pancreatectomy and died of coagulopathy. The remaining 9 have had no further bleeding.
Haemosuccus pancreaticus should be considered in patients with unexplained recurrent upper GI bleeding, epigastric pain, alcohol abuse and chronic pancreatitis. ERCP and selective angiography are essential for diagnosis and management. For bleeding sites in the head of the pancreas embolisation should be attempted to avoid major resection. Distal pancreatectomy is preferred for splenic artery lesions.

### F146

### MASSIVE DELAYED HEMORRHAGE AFTER PANCREATIC AND BILIARY SURGERY.

MI van Berge Henegouwen[1], JH Allema[1], TM van Gulik[1], PCM Verbeek[1], H Obertop[1], DJ Gouma[1]. Dept. of [1]Surgery, Academic Medical Centre, Amsterdam.

Massive delayed hemorrhage is a severe postoperative complication after pancreatic and biliary surgery, with a high mortality. Because of the different causes (i.e. ulcer disease, suture line bleeding, false aneurysm, erosion of the hepatic or splenic arteries) and treatment modalities, the management of these massive bleedings is under discussion. Therefore, in this study, we assessed the incidence, the symptoms, the etiology and the optimal diagnostic and interventional procedures of severe delayed bleeding.

Of the 686 patients that underwent major pancreatic and biliary surgery (from 1983 to 1993), patients with massive hemorrhage (> 6 packed cells within 24 hrs.) in the 'late' postoperative period (> 24 hrs. after initial surgery) were selected. Massive p.o. hemorrhage occurred in 22 patients (3.2%); two sub-groups were formed, according to etiology of bleeding, i.e. bleeding caused by erosion from a major artery (n=12) or bleeding from the (gastro) intestinal suture line (n=10). Both groups were compared with respect to bleeding parameters, symptoms, diagnostic and interventional procedures and management of the bleeding.

Hb level (means 4.4 vs. 5.0) and transfused units of blood (means 15.9 vs. 11.0) were not significantly different between the two groups. Patients with an arterial erosion had a longer interval between initial surgery and hemorrhage (p<0.05), more often septic complications (p<0.05) and had a higher mortality than patients with suture line bleeding, 50% vs. 0%, respectively (p<0.01).

Conclusions: Delayed massive hemorrhage was either due to erosion of a major artery or suture line bleeding, both giving equally severe symptoms. Especially a longer interval and septic complications are suspicious for a bleeding from an erosion of a major artery nearby the pancreatic or biliary anastomosis. Emergency GI endoscopy is the first step in the diagnostic process and is useful to exclude, as well as treat (by means of sclerotherapy) ulcer disease and suture line bleeding. Angiography and, if possible, embolization is the next step. If not successful, early aggressive surgical intervention is mandatory, including thorough exploration of the resectional area, ligated artery stumps and eventually opening of the Roux-Y limb. Oversewing of the bleeding site may lead to recurrent bleeding within 24 hours in most patients.

### F147

### NATURAL HISTORY OF PANCREATODUODENECTOMY.

F Mauvais, A Sauvanet, R. Noun, G Conzo, C Caraco, J Belghiti.
Department of Digestive Surgery, Hôpital Beaujon, F-92110 Clichy.

Biologic diagnosis of pancreatic and biliary complications following pancreaticoduodenectomy (PD) is difficult because changes due to the procedure are not known. The aim of this study was to describe normal biological changes after uncomplicated PD.
Materials and methods: From 1989 to 1993, 67 PD with pancreaticogastrostomy were performed. The postoperative course was considered as complicated in case of reoperation, pancreatic fistula (amylase level in fluid drainage > 5 X the serum level after day 3), pancreatitis with pancreas remnant enlargement on CT scan, abdominal collection, postoperative temperature > 38.5°C, delayed gastric emptying, serum creatinine level > 150 µM/L, and/or perioperative transfusion > 4 units. Thirty(45%) patients with uncomplicated course were analyzed : 22(73%) had a cancer, 18 (60%) were preoperatively jaundiced, and 14(47%) had a sclerotic remnant. Blood biochemical data (mean ± SD) were compared with Student's t-test.
Results :

| | Preop. | Day 1 | Day 3 | Day 5 | Day 8 | Day 10 |
|---|---|---|---|---|---|---|
| Amylase[(a)] | 69±49 | 193±188 | 82±73 | 45±36 | 42±39 | 40±37 |
| Bilirubin[(b)] | 106±139 | 100±120 | 58±78 | 49±55 | 50±62 | 48±53 |
| Alk. Phosph.[(c)] | 340±256 | 148±93 | 140±77 | 142±53 | 188±109 | 246±163 |
| ALT[(d)] | 190±175 | 194±169 | 103±102 | 67±46 | 85±68 | 75±42 |
| Leukocyte[(e)] | 74±31 | 124±42 | 116±42 | 86±24 | 104±28 | 109±38 |

[(a)]N<70U/L [(b)]N<20µM/L [(c)]N<130U/L [(d)]N<40U/L [(e)]X$10^2$/$mm^3$

Amylase serum level was significantly higher in case of normal remnant than in case of sclerotic remnant (284±196 *vs* 89±113, p=0,002) ; thereafter it was comparable to both groups. ALT serum level from day 1 to day 10 was not significantly different according to the presence or absence of preoperative jaundice. Rise in alkaline phophatase level at days 8 and 10 occurred in all patients.
Conclusion: After PD : 1) biochemical changes are normally maximum at day 1 and minimum at day 5 ; 2) rise of serum amylase level exceeding 3 times the normal level is the rule in case of normal remnant ; 3) cholestasis at days 8 and 10 is not due to a complication.

### F148

### RECONSTRUCTIVE OPERATIONS AFTER PANCREATODUODENECTOMY

M.Jeremić,M.Stojiljković,S.Jovanović, P.Stojanović,A.Bogićević,M.Stojanović

Surgical clinic.Clinical center Niš.Serbia Yugoslavia

The aim of this study is to evaluate the results of different recobstructive operations after 37 pancreatoduodenectomies performed in patients with malignant periampulary tumours in a ten-years period/1984-1994/.
All our patients had resectable tumours without sings of distant metastasis.Ex tempore biopsy of the resected part of the pancreas and regional lymph nodes was done in all cases.The reconstructive procedures performed were:Whiple with T-T pancreatojejunostomy, T-L hepaticojejunostomy and intramesocolis gastrojejunostomy with Brown (72,97%),and Longmire-Travers operation with pylorus preservation (27,13%).The most common complication was pancreatic fistula(5 cases-13,51%). In three cases the fistula closed spontaneusly,in the remaining two cases a reoperation was required.The operative mortality was 8,1% (3 patient died).The five-year survival rate in 18 follow-up patients was 7%.
Radical surgical operations with systematic lymphadenectomy,adequate reconstruction in which pylorus preservation had priority(due to less undesireable nutritional sequels)and well-performed telescopic pancreaticojejunostomy are the main factors for optimal results.

### F149

### THE RESULTS OF PANCREATODUODENECTOMY FOR CHRONIC INFLAMMATION OF THE PANCREATIC HEAD

G.N. Stapleton and R.C.N. Williamson
Poyal Postgraduate Medical School, Hammersmith Hospital London U.K.

A personal series of 50 patients underwent proximal pancreato-duodenectomy for severe chronic pancreatitis between 1979 and 1994. There were 13 women and 37 men with a median age of 43.5 years (12-70). Presenting features were chronic pain (n=47), obstructive jaundice (18), and duodenal stenosis (5). Cancer was suspected in 10. In addition, 13 had a pseudocyst, 3 had diabetes mellitus and 20 had exocrine failure. Aetiology was chronic alcohol abuse in 35. Five had had recurrent acute pancreatitis and 10 were idiopathic. Pylorus-preserving proximal pancreatoduodenectomy was performed in 44 patients and 6 had partial gastrectomy. Drainage of a dilated distal pancreatic duct by side-to-side pancreatico-jejunal anastomosis was included in 15 patients. Mean operating time was 6.2 hours (4.5-9.5) and blood loss 2.8L (0.2-13). There were no hospital deaths, but 3 patients required a second operation and 5 had percutaneous drainage of infected collections. During a median follow-up of 25.5 months (1-120) six patients required completion distal pancreatectomy for renewed pain; pain persisted in 4 others. Three required intervention for stricture at the biliary-enteric anastomosis. Five patients have died from unrelated causes. Proximal pancreato-duodenectomy is a relatively safe procedure effectively palliating pain in 80% of patients with chronic pancreatitis.

### F150

### WHAT IS THE REAL RISK OF PANCREATICODUODENECTOMY (PD) IN 1994 ?

A. Sauvanet , F. Mauvais , R. Noun, P. Lévy *, P. Bernades *, J. Belghiti.

Departments of Digestive Surgery and * Gastroenterology, Hôpital Beaujon, F-92110 Clichy.

During the last years, PD was presumed to be a dangerous procedure with a high morbidity rate. However, some teams reported recently large series of PD without mortality. The aim of this study was to describe recent changes in early results of PD with special references to its persisting risks.
Patients and methods : From October 1979 to March 1994, 123 patients (mean age : 55±12 years) underwent a PD, including 120 with a pancreatico-digestive anastomosis. There were 39 pancreatico-jejunal anastomoses (PJA) (1979-87) and 81 pancreatico-gastric anastomoses (PGA) (1988-94). Nine patients (7%) had a pyloric preservation, and 114 (93%) had a distal gastrectomy including 63 (63/114=55%) with a troncular vagotomy. Three successive groups (G1, G2, G3) of 41 patients each were compared to analyze the results evolution.
Results : The evolution of indications, mortality rate, rate of patients with uneventful postoperative course and amount of perioperative transfusion are described as follows :

| | G1 : 1-41 | G2 : 42-82 | G3 : 83-123 | Total |
|---|---|---|---|---|
| Malignant tumors | 28 | 32 | 23 | 83 |
| Benign lesions | 13 | 9 | 18 | 40 |
| Mortality (%) | 4(10%) | 2(5%) | 2(5%) | 8(7%) |
| Uneventful PO course (%) | 46 % | 46 % | 44 % | 46 % |
| Nb of red cell units (m±sd) | 4.8±3.5* | 3.1±3.2*° | 2.2±2.1° | 3.4±3.2 |

*p=0.03 ; °p=0.12 (Student's t-test)

Among the 8 postoperative deaths (due to 2 PJA fistulas, 2 non-pancreatic fistulas and 4 other causes), 3 occurred after PD for a benign lesion. No pancreatic fistula occurred on a sclerotic pancreatic remnant. The rate of pancreatic fistula on normal remnant was 17% (3/18) after PJA and 7% (3/45) after PGA (NS, Chi2-test). Delayed gastric emptying occurred in 2 cases (22%) after pyloric preservation and in 14 cases (12%) after distal gastrectomy (NS) without variation according to the completion or the absence of vagotomy.
Conclusions: this study suggests that mortality and morbidity after PD are persisting problems despite decrease in pancreatic fistula rate. These results lead us to limit indications of PD for a benign lesion.

### F151

### CARCINOMA OF THE PERIAMPULLARY REGION: WHO BENEFITS FROM PORTAL VEIN RESECTION?

J.D. Roder, H.J. Stein, J.R. Siewert.

Department of Surgery, Technische Universität München, Germany

The prognosis of patients with carcinoma of the periampullary region infiltrating the portal vein is dismal. We assessed the morbidity, mortality and prognostic effect of an extended resection including partial portal or superior mesenteric vein resection in these patients.
Patients and Methods: Between 1983 and 1994 a total of 277 partial duodenopancreatectomies were performed for tumors of the periampullary region at our institution with an overall morbidity and postoperative mortality of 37.4% and 3.9%, respectively. Despite an extensive preoperative workup including angiography of the celiac axis, portal vein invasion by the tumor was diagnosed intraoperatively in 31/277 patients. In an attempt to achieve complete tumor removal, a tangential excision (N=22) or a segmental resection (N=9) of the portal vein or superior mesenteric vein was performed in these patients.
Results: There was no postoperative mortality, postoperative morbidity was 41.9% (13/31 patients). Histopathologic work up of the resected specimen showed a ductal adenocarcinoma of the pancreas in 22, carcinoma of the distal bile duct in 7, cystadenocarcinoma in 1 and an acinus cell carcinoma in 1 of these patients. Tumor infiltration of the resected vein could be documented histopathologically in only 19/31 (64.3%) patients. All patients with pancreatic or bile duct carcinoma died within 16 months of the resection (median survival 8 months). In contrast, the patients with cystadenocarcinoma and acinus cell carcinoma are alive with no evidence of recurrence 23 and 54 months after the resection.
Conclusion: Portal vein resection, although save in experienced hands, does not prolong survival in patients undergoing partial duodenopancreatectomy for carcinoma of the pancreas or distal bile duct. Only the occasional patient with cystadenocarcinoma or acinus cell carcinoma may benefit from this approach.

### F152

### PANCREATOGASTROSTOMY : A SAFE DRAINAGE PROCEDURE AFTER PANCREATODUODENECTOMY

JP.Arnaud, R.Bergamaschi, C.Casa, C.Leroux

CHU ANGERS - Department of Visceral Surgery

4, rue Larrey 49033 ANGERS CEDEX 01 - FRANCE

The propensity for leakage and disruption at the site of the pancreaticojejunostomy is a major reason for morbidity and death after pancreaticoduodenal resection.The purpose of this study was to evaluate the role of pancreaticogastrostomy as an alternative method of restoring pancreaticointestinal continuity after pancreaticoduodenectomy.
From January 1989 to November 1994, 76 patients have undergone pancreticogastrostomy after pancreaticoduodenectomy at our institution. 47 patients were men and 29 women. The mean age was 60.7 years (range 42-74 years). Pancreaticoduodenectomy was performed for pancreatic carcinoma (28 patients), ampullary carcinoma (20 patients) biliary carcinoma (7 patients) duodenal carcinoma (6 patients) and chronic pancreatitis (15 patients).
There was two postoperative deaths, for an overall operative mortality rate of 2.6%. There was one pancreatic fistula (1.3%) which recovered with further surgery. The average postoperative stay in the hospital was 16 days.We have observed twelve exocrine insufficiency and ten insulino-dependant diabetes. A endoscopic examination with injection of pancreatic duct was performed in 15 patients at the third month : in 12 cases we had a visualization of the anastomosis between the stomac and the pancreatic duct. Pancreatic secretary studies performed in 15 patients were normal.
These results confirms that pancreatogastrostomy is a safe method of pancreatic drainage after pancreatoduodenectomy and suggests that it may have technical advantages and therefore merits more widespread application.

### F153

**CONCOMITANT, EXTENDED & COMBINED OPERATIONS OF MULTIPLE ABDOMINAL ORGANS BY SINGLE APPROACH.**

Prof.MD.Daskalov M.PhD.II[nd] Surgical Clinic, Sofia,Bulgaria./ Faculty of medicine /.

The author operated 272 pts for concomitant diseases of more than one abdominal organs.I42pts had cholecystitis & cholelithiasis along with diseases of liver and pancreas.Peristalsis resumed im 3-6 post-oper.day,pts got up by 5-6 d, released in I0-25 days.All were cured.Operated were 4Ipts.for gastric diseases along with diseases of hepatobiliary system,liver,pancreas, abdomimal walls,etc.Peristalsis resumed in 4-6 days,pts.got up in 6-I2 days,released in I4-23d. Cured were 39pts.Operated were 2Ipts.for differ. tyoes of concomitant diseases:resumed peristalsis in 3-6days,pts.got up in 4-6 days,released in 9-20 days.28pts.were operated for appendicitis along with diverticulitis,ovariancyst,diseases of adnexa etc.Peristalisis resumed in 3-7 days,pts.got up by I6-25 days.Cured-27 pts. out of 272 pts.270 resumed peristalsis in 2-6 days,got up in 2-14 days and were released in 7-25 days.Cured-272pts.Three pts.developed suppuration.Two pts.had prolonged lethal condition. They had carcinoma of the stomach/T4/with local infiltration in and around the oesophagus,pancreas,liver,colon etc..After 3rd block operation they developed peritonitis.
Our experience has proven that single approach operations on multiple abdominal organs can be successeful.In such cases selection of patients and appropriate steps of approach should be performed for combined operations by single suitable incision,followed by proper anestesia, drainages,post operative care and suitable antibiotics.

### F154

**APPLICABILITY OF LAPAROSCOPIC SURGICAL TECHNIQUES FOR TREATMENT OF BILIARY PANCREATITIS**

EM Targarona, C Balagué, J Martínez, J Rodríguez, JJ Espert, S Pascual, R Perez-Ayuso, E Ros, S Navarro, JM Bordas, J Terés, M Trías.
Serv. of Surgery, Gastroenterology and Endoscopy Unit. Hosp. Clínic. Univ. of Barcelona. Barcelona. SPAIN.

The application of laparoscopy for treatment of acute biliary pancreatitis (ABP) is not well defined. Laparoscopy can be used for diagnosis, treatment of gallstones and in selected cases, it can resolve complications (pseudocysts) **AIM:** To evaluate the applicability of laparoscopic surgery for the management of ABP following a policy of selective preoperative ERCP, intraoperative colangiography and stone retrieval. **MATERIAL & METHODS:** Between Jan /92 and Dec /94, 219 lap cholecystectomies (Group I, CxL) non related to ABP were performed and 62 patients were treated after an episode of ABP (Group II, CxL.ABP). It was evaluated the type of laparoscopic procedure, associated endoscopic manoeuvers, operative time, stay and morbi-mortality comparing both series. **RESULTS:** 71 lap. procedures were performed in 62 patients with previous ABP: 1 exploratory laparoscopy for acute abdomen, 62 lap cholecystectomies with intraoperative cholangiography, 6 transcystic exploration of the bile duct and 2 transgastric cystogastrostomy. Preoperative ERCP was performed in 9 patients with a high suspicion of duct stones (dilated duct, US demonstration, jaundice or sustained elevation of LFT) retrieving 1 calculi. In 3 cases, stones were retrieved perioperiatively via the cystic duct, and two cases were converted. There were no technical or postoperative differences between both groups (Table). Transgastric intraluminal cystogastrostomy was attempted in two cases of ABP and succesfully performed in one.

| | CxL | CxL.ABP | p |
|---|---|---|---|
| N | 219 | 62 | |
| Age | 55±15 | 54±17 | ns |
| Operative time(min) | 95±42 | 99±47 | ns |
| Conversion | 9.7 % | 10 % | ns |
| Morbidity | 13 % | 11 % | ns |
| Reoperation | 1.4 % | 1'6 % | ns |
| Postoperative stay(d) | 3.9±5.4 | 4'6±5'8 | ns |
| Mortality | 0.5 % | 0 % | ns |

**CONCLUSION:** **1.** ABP does not add technical difficulties for laparoscopic cholecystectomy. **2.** A laparoscopic approach with a selective policy of preoperative ERCP, systematic intraoperative cholangiography and laparoscopic retrieval of stones could be applied for elective treatment of ABP. **3.** Selected complications of ABP, as pancreatic seudocyst, can be solved with a minimal invasive therapy.

### F155

**ACUTE RENAL TUBULAR DYSFUNCTION IN ACUTE PANCREATITIS**

**C.P.Driver[+], D.N.Anderson[+], I.J.Broom[*] and P.H.Whiting[*].**
Department of Surgery[+] and Biochemistry[*]
Aberdeen Royal Hospital's NHS Trust,
Aberdeen, Scotland

23 consecutive patients (11 males and 12 females) were admitted as emergency cases with acute pancreatitis. The aetiology of the acute attack was alcohol induced in 8(35%), biliary in 12(52%) and idiopathic in 3(13%). The diagnosis of acute pancreatitis was confirmed by an elevation in the serum amylase at the time of admission. The mean amylase was 1470 U/l (range 213 - 4907 ; upper limit of normal <90 U/l). The severity of the acute attack, and with it the prognosis at the time of admission was assessed by a variant of the Imrie classification and ranged from 0 - 3.

The serum creatinine concentration measured on admission of 81±18 umol/l (Mean ± S.D.), was within the normal range, suggesting no acute renal impairment. The urinary enzyme N-acetyl-B-D-glucosaminidase(NAG) was measured in each patient on admission, and was elevated in 70% of individuals (113±104, range 18-399 U/mmol Creatinine). This elevated NAG level showed no correlation with the serum creatinine (p=0.422) or amylase concentration (p=0.376), nor with the modified Imrie prognostic score (p=0.291).

This study shows that a significant renal insult is present on admission in patients with acute pancreatitis but that this is not being detected by conventional serum parameters.

### F156

SEVERITY OF ACUTE PANCREATITIS AND ITS ETIOLOGICAL CORRELATION

MA.Secchi, W. Sanchi, E. Tagliaferri, S.M. Krupik,G.Raimundo. Surgical Division. Hospital Italiano and Hospital Provincial. Rosario. Argentina.

**INTRODUCTION:** Acute pancreatitis(A.P.) is a disease of variable etiology,being lithiasic pancreatitis the most frequent in our setting (70-80%).The severity and prognosis of A.P. is directly related to the cause that gave rise to the disease. **METHODS:** from April 1987 and November 1994,223 patients suffering from A.P. were prospectively studied.The etiologic cause was determined and they were staged as mild moderate and severe.The prognosis based on this classification was performed by combining our "original index"(9 non morphological parameters and McMahon method. Apache II and Baltazar-Ranson score were also used in the group of Severe A.P. We were evaluated prevalence of severity and mortality and compared with the lithiasic Group. **RESULTS:** 158 patients were lithiasic etiology, 20 idiopathic,12 alcoholic, 8 virus mumps, 7 post operative, 7 dislipemic, 5 inflamatory, 4 drug induced, 1 traumatic, 1 inmunologic. The highest prevalence of severity and mortality corresponded to dislipemic:42% and 14%(p<0.01) and post operative A.P.:28% and 14% (p<0.01). Idiopathic and alcoholic A.P. had a high rate of severity (30% and 25%) and mortality (5% and 8%) in relation to the lithiasic group (p<0.05). Lithiasic A.P. is highly prevalent (71%), but both severity and mortality rates are low (10.7% and 1.8% respectively). **CONCLUSION:** The prognosis of severe A.P. of lithiasic origin is significantly better than those of dislipemic, post operative, idiopathic or alcoholic origin.

### F157

### ACUTE PANCREATITIS OF UNKNOWN AETIOLOGY

P. Vrachnos, L. Papastamatiou, N. Christoforides, A. Kordonis
2nd Dpt. of Surgery, "Apostle Paul" Hosp-KAT. ATHENS-HELLAS

Unclear aetiology of acute pancreatitis presents reduced rates nowadays as progressive knowledge and modern interventional procedures clarify the cause of the disease in most cases. Recent reports show a special interest on acute pancreatitis with unknown aetiology in the elderly concluding that the illness is related with greater compromise of organ function and mortality.

This study, however, presents three cases of young patients with fulminant course of acute necrotizing pancreatitis of unknown aetiology: Two women (aged 23-32y) and a man (aged 42y) presented in admission early organ failure and consisted a diagnostic problem. Interestingly the onset of acute symptoms was only 3-5 hours before. Ultrasonography, CTscan and laboratory findings established the diagnosis. Levels of Ranson's criteria were significantly higher than in cases of known aetiology patients. The overwhelming course of the disease obliged to early intervention (36-48h) in both women, subjected to pancreatic debridement and wide drainage for extended pancreatic and peripancreatic necrosis. The older (32y) died 24 hours postoperatively (multiple organ failure). The man exceeded 4 days after admission, rather suddenly, while his general status was ameliorated. At autopsy peripancreatic necrosis was more extended than the pancreatic per se.

It is concluded that acute pancreatitis of unknown aetiology, presenting short time of symptoms, fulminant course, early multiple organ failure and high mortality rates consists perhaps a separate medical entity. The illumination of its aetiopathogenesis might be in the field of genetics, endocrinology and molecular biology, although undetected sludge and poly-microlithiassis is accused to be the main cause of the disease.

### F158

### ENDOSCOPIC STENTING FOR PANCREATIC DUCT LEAKAGE AFTER NECROSECTOMY FOR ACUTE HAEMORRHAGIC NECROTISING PANCREATITIS.

MJ Boom, W Dickey*, TM van Gulik, PCM Verbeek, EAJ Rauws*, DJ Gouma, H. Obertop. Department of Surgery and Gastroenterology*, Academic Medical Centre, Amsterdam, The Netherlands.

Acute haemorrhagic necrotising pancreatitis is preferably treated conservatively, while necrosectomy and (open) drainage are performed for septic complications. The latter treatment is sometimes associated with leakage of the pancreatic duct and/or formation of a pancreatico-cutaneous fistula. Management of this complication consists of fasting, total parental nutrition and, more recently, somatostatine analoques. This policy eventually will lead to reduction of leakage or closure of most fistulas, but can take up to six months. Stenosis of the pancreatic duct or spasm of the papillary sphincter could impede closure. Therefore, the aim of this study was to evaluate the effect of pancreatic duct stenting on closure of pancreatic duct lesions and reduction of leakage via the open abdomen.
In the period 10/93-5/94 we treated 4 patients with pancreatic duct leakage. They underwent 2-9 surgical debridements for pancreatic necrosis, leading to open laparostomas (and eventually pancreatico-cutaneous fistulas) in 3 patients and a persisting drain production in 1 patient. Production of the fistulae was 120-600 ml/day, with an amylase content of 20,000-190,000 U/L. Existence of the fistulas since the first exploration ranged from 3 weeks to 7 month. Succesful placement of an endoprosthesis was achieved after a mean of two attempts in all patients. There were two normal pancreatic ducts at endoscopy and two with a proximal stenosis. Duct disruption, with leakage, was seen in all. Leakage of pancreatic juice ceased after a maximum of three days. The endoprosthesis was removed after 6 wks without recurrence.
From these preliminary results we conclude that endoscopic stenting of the pancreatic duct can be useful in the management of pancreatico-cutaneous leakage after surgical debridement for acute hemorrhagic necrotising pancreatitis, leading to early closure of pancreatic duct leakage.

### F159

### NUTRITIONAL SUPPORT IN ACUTE PANCREATITIS

C. Papapolychroniadis, *H. Makedou, S. Marcakidis, B. Papadopoulos, *D. Michailidou, N. Harlaftis, C. Katsohis
First Propaedeutic Surgical Clinic, *Microbiology Department of Aristoteles University of Thessaloniki, A.H.E.P.A. Hospital, Greece

This study was performed to assess the efficacy of early institution of parenteral nutrition in patients with acute pancreatitis in relation to nitrogen balance. During the period from 1993 to 1994 , 35 patients were admitted to our I.C.U. with acute pancreatitis. In 20 patients concomitant chololithiasis and/or choledoholithiasis was found. There were 19 males and 16 females with a mean age of 61,8 years. All patients were given TPN. General dosage recommendations were used in parenteral regimens per kg of body weight per day: aminoacids up to 1.5 gr., glucose up to 5.0 gr. and fat up to 1.5 gr. The accurate measurement of the exact nitrogen balance is difficult to be accessed in every day practice. However, we find very useful the approximation:

$N_{bal}$ (g/24 h.) = $N_{in}$ - [UNN + 4 + ($BUN_E$-$BUN_S$ /100 x B.W. X F)]

Twenty patients were subjected to surgical intervention and 15 patients to conservative treatment. Three patients died as a result of sepsis that followed acute necrotising pancreatitis. There has been tremendous interest in investigating the importance of objective nutritional index and the nitrogen balance, in the hope that this knowledge would permit better management of patients with acute pancreatitis.

### F160

### SEVERE ACUTE PANCREATITIS IS ASSOCIATED WITH PROLONGED BLOOD MONONUCULEAR CELL INTERLEUKIN-6 AND INTERLEUKIN-8 RELEASE

A.C. de Beaux, J.A. Ross, K.C.H. Fearon, D.C. Carter
University Department of Surgery, Royal Infirmary, Edinburgh, Scotland. EH3 9YW

Leucocyte activation and pro-inflammatory cytokine release is thought to contribute to the systemic sequelae of acute pancreatitis. We have measured release of IL-6 and IL-8 in the cell culture supernatant of peripheral blood monoculear cells (PBMCs) after 24 hours incubation. Cells were isolated from 6 healthy volunteers and from 14 patients with acute pancreatitis (6 severe, 8 mild: Atlanta classification) on day 1 and 5 of admission. The results are shown in the table as the mean(SEM).

| | | Day 1 | | Day 5 | |
|---|---|---|---|---|---|
| Cytokine | Control | Mild | Severe | Mild | Severe |
| IL-6 | 791 | 2855 | 3488 | 1538 | 6724 |
| pg/ml | (168) | (615) | (980) | (418) | (3093) |
| IL-8 | 35.0 | 85.5 | 174 | 53.8 | 131 |
| ng/ml | (16.5) | (30.9) | (49.7) | (27.8) | (28.7 |

PBMC IL-6 release in patients with both mild and severe disease on day 1 is increased compared with the control group (p<0.02 Mann-Whitney U). However, there was no significant difference in IL-6 release between the two patient groups on day 1 (p=0.15). In contrast, on day 5, IL-6 release was significantly increased in the severe group (p=0.01) but not the mild group (p=0.3) compared with the control group. IL-8 release in the 3 study groups shows a similar pattern. The severity of acute pancreatitis is associated more with the duration of leucocyte activation than the intensity of activation.

### F161

THE SURGICAL MANAGEMENT OF INFECTED PANCREATIC NECROSIS:AN UNRESOLVED PROBLEM

M.Jekić,I.Jekić

Surgical Service,Clinical Hospital Center Zemun-Belgrade,Yugoslavia

Despite a better understanding of the causes of infected pancreatic necrosis,the widespread availability of CT-scan and of supportive care the results after surgical treatment are,to date,deludent.Our series includes 15 patients (mean age 48 years).In our experience aetiology of infected pancreatic necrosis was:alcoholic pancreatitis in 46.6% of cases;infection of a pancreatic collections in 26.6%;postoperative pancreatitis in 13.3%,biliary didease in 6,6% and hydiopatic pancreatitis in the remaining 6.6%of cases.
Surgical procedures suggested are:local drainage only in critical patients or wide drainage (including exploration of retropancreatic spaces taking down hepatic and splenic flexures). After this surgical procedurés,external drainage of all collections is mandatory.
Mortality rates after coventional surgical drainage have approached 50% in different series.Two our patiènts died for recurrent sepsis despite surgery and intensive medical treatment.

### F162

Current therapy of acute pancreatitis.

C.Fotiadis. B.Harrisi.S.Giannakakis. An.Lazaris
Gh.Papanikolopoulou. M.Sechas.

The aim of our study is to present our experience in the treatment of acute pancreatitis. In our Department from 15-2-93 to 15-12-94 we treated nine patients, five of them were men and four women. Mean age of our patients 52 years. In two cases lithiasis of bile truct was present. All patients were treated concervatively except two cases. These two patients suffered from pseudocystis of pancreas as comlication and went under laparotomy and surcgical threatment.

The means by which acute pancreatitis should be treated is depended on the etiology and the prediction of severity of the disease. Is worlwide accepted that the optional treatment is conservative and surgical intervention would be helpful in selected groups of patients withsevere course of the disease complications, gallstones pancreatitis extrapancreatic disease and for diagnostic purposes. Intensive care settings labaratory and radiographic support have been helpful in decreasing the morbidity and mortality.

### F163

CARCINOMA OF THE BODY AND TAIL OF THE PANCREAS.

C. Sperti, C. Pasquali, V. Costantino, S. Pedrazzoli.
Semeiotica Chirurgica - University of Padua, Padova, Italy.

Carcinoma arising in the distal pancreas is generally seen with advanced disease, and is, therefore, less frequently resectable for cure. Although few informations are available regarding the results of curative distal pancreatic resection for pancreatic carcinoma, long-term survivors have only occasionally been reported. The purpose of this study was to review experience of carcinoma of the body and tail of the pancreas, with particular attention to long-term survival. From 1970 to 1992, 145 patients (out of 536 pancreatic cancers; 27 %) were diagnosed with an histologically proven adenocarcinoma of the distal pancreas. 59 patients (41%) underwent exploratory laparotomy only and 27 (19%) palliative procedures, with 7 operative mortality (8%). Median survival time was 4.5 and 5 months after explorative and palliative surgery, respectively. No patient survived more than 17 months. 35 patients with advanced disease were not operated: median survival time was 2.5 months (no patient survived more than 12 months). 24 patients (16%) underwent distal pancreatectomy: 8 patients had non-radical operation for macroscopic tumoral remnant, and 4 procedures involved en-bloc resection of other organs(colon, stomach, and kidney), with one vascular resection.Two patients (8%) died postoperatively. Median disease-free and median survival times were 9 and 11.5 months, respectively. Median survival time was 8.5 months for non-radical and 15 months for radical operations. Three patients survived more than 5 years, and are still alive and disease-free, after 10 years: they had small (T1), localized cancers detected at operation for chronic pancreatitis. In conclusion, most patients with adenocarcinomas of the body and tail of the pancreas are unresectable and survive for only a short period. However, radical resection may offer benefit for some localized tumors, and should be performed whenever possible. Many efforts must be made on early diagnosis in patients with pancreatic adenocarcinoma.

Study supported by Italian National Research Council (CNR), project nr. 93.02236.PF39.

### F164

TECHNICAL ASPECTS OF DIAGNOSTIC LAPAROSCOPY COMBINED WITH LAPAROSCOPIC SONOGRAPHY IN PREOPERATIVE STAGING OF CANCER OF THE PANCREATIC HEAD REGION.

LTh de Wit[1], WA Bemelman[1], OM van Delden[2], NJ Smits[2], H Obertop[1], EAJ Rauws[3], DJ Gouma[1]. Dept. of Surgery[1], Radiology[2] and Gastroenterology. AMC, Amsterdam, The Netherlands

Since the revival of minimally invasive surgery, diagnostic laparoscopy gained ground in the preoperative staging of pancreatic cancer. Small liver and peritoneal metastases can be detected with a high accuracy rate. The development of a laparoscopic sonography probe made it possible to evaluate the solid organs and retroperitoneal space during the same procedure without disturbance of overlying bowel gas or thick abdominal wall. In the past year experience has been obtained with this new diagnostic modality.

This video will show the technique and possibilities of laparoscopy combined with ultrasound in the preoperative staging of pancreatic cancer. Using a three 10/11-mm trocar approach the abdominal cavity is investigated for peritoneal and hepatic deposits and malignant infiltration of mesocolon and Treitz ligament. Laparoscopic exploration of the lesser sac and biopsy of lymph nodes around the coeliac axis will be demonstrated.
Laparoscopic ultrasound of the liver and of the tumor with regards to vascualr involvement (local unresectability) will be shown. Biopsies of suspicious lesions are taken under direct laparoscopic or ultrasound guidance using biopsy forceps or Tru-cut and Rotex biopsy needles.

In 70 patients with a presumed Stage I tumor of the pancreatic head region local vascular involvement was correctly predicted in 93% and laparotomy was avoided in 19%.

### F165

DUODENO-PANCREATECTOMY WITH PYLORUS PRESERVATION: FOR WHICH PATIENTS?

G. Chassot, A. Rohner, P. Morel
Department of abdominal surgery, Geneva Cantonal Hospital, Switzerland.

In 1978, Traverso and Longmire described a modified Whipple procedure with pylorus preservation (PP). The advantage of this procedure is to keep the entire stomach with a functional pylorus and therefore to improve the digestive comfort of the patients with a gain of weight.
Between 1984 and 12.1993, we performed at our Hospital 51 PP: 27 for chronic pancreatitis (the majority of alcoholic origin), 24 for tumors (2 insulinoma, 8 adenocarcinoma of the Vater Ampulla, 7 head of the pancreas adenocarcinoma, 4 duodenal adenocarcinoma and 3 of the low choledocus). We have a complete follow up for 96% of our patients with a mean of 76 months in the first group and 33 months in the second one. We have no per or postoperative mortality and an incidence of 15% immediate postoperative complications. We observed 5 cases (10%) of anastomotic ulceration with 2 cases of perforation into the peritoneal cavity, 4 of them requiring an antrectomy-vagotomy. We prevented this complication in our last 31 patients with a shortening of the distance between the biliary and duodenal anastomosis (10cm) and the preservation of a competent pyloro-duodenal sphincter (2-3cm of duodenum preserved). The short and long term beneficial effects of PP on patients well being and nutritional status were confirmed in patients with pancreatitis, with 22 (82%) pain free and with a mean gain of weight of 8.2 kilos. The average frequency of stools is twice per day. In patients suffering from a tumor this outcome appears less obvious due to the cause of the disease; their mean survival time is of 19 months (6-24 months) with 7 patients still alive with a satisfactory postprandial comfort. This survival is similar to the outcome observed after the Whipple procedure.
In conclusion, when technically possible, the PP is the procedure of choice for cephalic resections in pancreatitis.
Despite the controversy regarding the use of this operation for pancreatic tumors (incomplete oncological resection?), the mean survival time in our series as well as in recent studies, is similar to the classical Whipple procedure for periampullary adenocarcinoma and for resectable carcinomas of the head of the pancreas.

### F166

PROGNOSTIC IMPACT OF INVASION ASSOCIATED PROTEOLYTIC FACTORS IN CARCINOMA OF THE PANCREAS

H. Nekarda[1], J. Roder[1], H. Vogelsang[1], K. Becker[2], ,J.R. Siewert[1]
Department of Surgery and [1]Institut of Pathology, Technische Universität München, Klinikum rechts der Isar, Germany.

Objective: The urokinase-type plasminogen activator uPA, the receptor uPA-R and inhibitor PAI-1 are elevated in cancer tissue extracts and are related to invasion and metastasis. Recently , we showed the prognostic impact of PAI-1 and uPA for gastric cancer patients (Cancer Research 54, 2900-2907, 1994).
Materials and methods: We investigated prospectively 40 patients (1988-1994) who experienced a resection of the pancreas for ductal cancer. 15 patients (38%) belongs to stage I, one patient to stage II and 24 patients (60%) to stage III. Median calculated survival was 10 months. uPA , uPA-R and PAI-1 content were quantified by ELISA in detergent-extracted specimens of pancreatic tumors and normal pancreatic tissue (n=15) which was snaped frozen and microscopally investigated.
Results: Significantly higher median values were determined in tumor tissue extracts compared to normal pancreas tissue (uPA: 4.9 versus 0.3 ng/mg protein, uPA-R: 7.2 versus 0.09 ng/mg protein and PAI-1: 79 versus 9.7 ng/mg protein).
By univariate Cox regression analysis pT-category, uPA and PAI-1 content were significantly correlated to overall survival. By multivariate Cox regression analysis PAI-1 content and pT-category were the only prognostic factors. In the subgroup of nodal negative pancreatic cancer patients uPA-R was the only significant prognostic factor which showed a tendency (p=0.07) in the whole group. Nodal status, size and grading had no prognostic impact.
Conclusion: PAI-1 and uPA-R content of ductal pancreatic cancer are new independent prognostic factors predicting shorter overall survival even in the subgroup of nodal negative patients.
Grant DFG GR 280/4-1

### F167

PROGNOSTIC FACTORS FOR SURVIVAL AFTER PANCREATODUODENECTOMY FOR CARCINOMA OF THE PANCREATIC HEAD REGION

J.H. ALLEMA, M. REINDERS, T.M. v. GULIK, D.J. v. LEEUWEN, D.J. GOUMA
Departments of Surgery and Gastroenterology, Academic Medical Centre, Amsterdam, the Netherlands

Aim of the study was to determine prognostic factors for survival after pancreatoduodenectomy (PD) for carcinoma of the pancreatic head region.
Methods. In the period 1983-1992, 176 patients underwent PD for ampullary carc. (n=67), distal bile duct carc. (n=42) or pancreatic carc. (n=67). First choice for resection was subtotal PD (n=146). Patients with a tumor positive pancreatic margin or a brittle pancreatic duct underwent total PD (n=30).
Results. Mortality was 5% for subtotal PD and 20% for total PD. Overall five year survival was 31%. Five year survival after PD for ampullary carc. (50%) was significantly better than for distal bile duct carc. (24%) and pancreatic carc. (14%). Overall negative prognostic factors for survival were involved resection margins (p<0.001), major vascular involvement (p<0.001) and blood transfusion >4 units (p=0.008). A tumor size of more than 2 cm, lymph node involvement and a poor differentiation grade were negative factors for patients with distal bile duct carc. and pancreatic carc. but not for patients with ampullary carc..
Conclusion. Survival after PD for ampullary carc. was significantly better than for distal bile duct carc. and pancreatic carc.. The strongest overall negative prognostic factors were involvement of resection margins and major vascular involvement. Tumor size, lymph node involvement and differentiation grade were significant factors, but not for the subgroup of ampullary carc..

### F168

CHRONIC PANCREATITIS OR CANCER OF THE HEAD OF THE PANCREAS - THE DIAGNOSTIC DILEMMA

J.D. Roder, J.R. Siewert
Department of Surgery, Technische Universität München, Germany.

The pre- and intraoperative differentiation between a malignant process and chronic pancreatitis in patients with a tumor of the pancreatic head frequently presents a diagnostic dilemma.
Methods: Based on morphologic criteria suggestive of a malignant process on the preoperative diagnostic imaging studies (ERCP, CT, endoscopic sonography, and angiography) we performed a partial pancreato-duodenectomy in 254 patients. Because of the well known diagnostic problems pre- or intraoperative proof of a malignant tumor was not required for resection.
Results: Histopathologic evaluation of the resected specimen showed an adenocarcinoma of the duodenum or the papilla vateri or the distal bile duct in 110/254 patients and a tumor of the head of the pancreas in 144/254 patients (ductal adenocarcinoma in 90, malignant endocrine tumor in 7, cystadenocarcinoma in 8, metastases in 4, sarcoma in 1, and acinus cell carcinoma in 1 patient). Chronic pancreatitis but no malignant tumor was found in 33/144 (22.9 %) patients. The postoperative mortality rate was 1 % in patients with a malignant tumor of the head of the pancreas. None of the patients with chronic pancreatitis died post-operatively.
Conclusion: Despite the use of modern diagnostic techniques the presence of a malignant process in patients with a tumor of the pancreatic head can frequently not be diagnosed pre- or intraoperatively. In experienced centers a partial pancreato-duodenectomy can be safely performed in this situation and can be regarded as the most effective and reliable method to obtain tissue for histopathologic assessment.

### F169

INFLUENCE OF MUCIN CHARACTERISTICS ON SURVIVAL OF AMPULLARY CARCINOMA

Nafas R, Simatos G, Antonopoulos K, Poulantzas J.
4th Surgical Department of "Evangelismos" Hospital Athens Greece

Ampullary carcinoma is associated with a change in the characteristics of mucin secretion within the ampulla of Vater. The aim of this study is to assess if there is any correlation between mucin characteristics and survival in ampullary carcinoma.
The study group consisted of 17 patients operated between Jan 1988 and Jan 1992, for carcinoma arising only within the ampulla of Vater as this was specified by the histopathologic study. They were 14 men and 3 women (mean age:63, 8 years).
Twelve underwent pancreatoduodenectomy and five wide local resection of the tumor. All patients were followed for at least three years. All tumors were adenocarcinomas.
According to the mucin produced carcinomas were divided in to tumors producing predominantly sialomucins (8 cases) and tumors producing predominantly sulphated mucins (9 cases). There was no significant difference in age at operation and size of tumor between the two groups. It was found that tumors producing predominantly sialomucins had a significantly better 3 year survival rate (75%) than tumors producing predominantly sulphated mucins (11%) ($p<0,05$).
If carcinomas were divided into tumors producing only sialomucins (3 cases), tumors producing both sialomucins and sulphated mucins (8 cases) and tumors producing only sulphated mucins (6 cases), the 3 year survival rates were 66,5%, 62,5% and 0% respectively.
Further follow up is necessary to establish mucin characteristics as an independent prognostic factor for ampullary carcinoma.

### F170

**MORBIDITY, MORTALITY AND SURVIVAL AFTER PANCREATODUODENECTOMY**

F. Kalfarentzos+, S. Kakkos+, M. Melachrinou++, G. Peppas+, J. Spiliotis+, J. Androulakis+.

Department of Surgery+, Department of Pathology++ ,Medical School of Patras, Greece.

One hundred and twenty nine consecutive patients with pancreatic tumors were admitted during the period 1987-1994. Twenty seven of those patients underwent pancreatoduodenectomy and were studied retrospectively as far as morbidity, mortality and survival is concerned. The preoperative diagnosis was made on the basis of CT scan and ERCP. Histological confirmation was achieved in most cases intraoperatively. However, in 60 % of cases the decision for pancreatectomy was based on clinical criteria. Pancreatic resection was performed in 27 patients for lesions of the head of pancreas. Twelve patients had adenocarcinoma of the head of pancreas, 7 patients had carcinoma of ampulla of Vater or duodenum, 2 patients distal common bile duct carcinoma, 2 cystadenocarcinomas and 1 malignant endocrine tumor. In three patients (11%), histology showed chronic pancreatitis. Staging of the tumors included 11 patients with stage I and 13 with stage III. Total pancreatectomy was done in 5 patients and proximal pancreatectomy in 22 patients. Pyloric preservation performed in 3 patients and truncal vagotomy in 7. In 22 patients with proximal pancreatectomy, pancreaticojejunal anastomosis was done in 9, pancreatic duct ligation in 12 and pancreatic duct exteriorization in 1. Postoperative morbidity was 63% in general and 48% for major complications (pancreatic fistula 33%, intraabdominal collections/abscesses in 18%). Postoperative mortality was 7.4%. Predicted mean 3-year survival was 50% for all tumors. For adenocarcinomas of the head of pancreas mean 1-year survival was 48%, while for the remaining neoplasms it was 82%.
In conclusion our study shows that pancreaticoduodenectomy currently has an accepted rate of morbidity, low mortality and increased long term survival of patients. On these basis, whenever possible, pancreaticoduodenectomy is the treatment of choice for malignant pancreatic tumors.

### F171

The prognostic value of cytology and the tumor markers CEA and CA 19-9.

CHJ van Eijck, SO Muller, J Jeekel, R. Heide, R. Chin, BG Blijenberg.

**Affiliation**: Dept. of Surgery, Dept. of Chemistry, Dijkzigt Hospital Rotterdam, the Netherlands.

**Purpose**: A. To detect a peroperative tumor spill during resections of gastrointestinal tumors using cytology and tumor markers in peritoneal washings. B. To prove the predictive values of tumor markers in peritoneal washings for intra-abdominal tumor reccurence after resection of the tumor.
**Introduction**: After curative resections of gastrointestinal tumors, the intra-abdominal reccurence comprises about 70-85% of tumor failure. This intra-abdominal tumor reccurence is thought to be the result of a peroperative tumor spill. Tumor cells and other substances are spilled when lacerating the serosa, the intestinal lumen and the lymphatic and venous blood vessels.
**Methods**: A population of 52 patients with a gastrointestinal tumor was used; 22 patients had an esophageal or gastric adenocarcinoma, 17 a pancreatic carcinoma and 13 a colorectal carcinoma. The tumor was resected in 42 patients, while 10 underwent a laparotomy. The first washing was performed immediately after opening of the abdomen; while the second one was performed after tumor resection or exploration, before closing of the abdomen.
**Results**: A pilot study showed the insignificance of general cytology of peritoneal washings. 50% of patients with a normal concentration (<30 U/ml) of CA19-9 in the first washing had raised concentrations in the second washing; while 39% with a normal (<2.0 ng/ml) CEA in the first washing had elevated levels of CEA in the second washing. Since the majority of these patients developed peritoneal metastases, these elevated levels could signify a tumor spill.
Tumor marker levels in the second peritoneal washing showed promising results in relation to intra-abdominal reccurence.
CEA had a negative predictive value of 87% and a positive predictive value of 68%, while CA19-9 had a negative predictive value of 90% and a positive predictive value of 67%. When combining both tumor markers the results were a negative predictive value of 94% and a positive predictive value of 71%.

### F172

PANCREATIC INSOULINOMAS ; COMPARISON OF CT,DSA,MRI MR ANGIOGRAPHY

K. LIBEROPOULOS*, K. KOKKINIS* A. MITRAKOU**, S.KAVVADIAS*, M.TSIRULAS* M.DUSKO*, T.RAPTIS**, K.SIRINGRARIS*, S.RAPTIS**.

*Dpt. of Diagnostic Radiology, Medical Imaging ,AthensGeneral/GREECE

** 1rst dpt. of Pathology, EVANGELISMOS Hospital, Athens /GREECE

Our purpose was to compare the diagnostic value of computed tomography (CT), digital substracion angiography (DSA), magnetic resonance angiography in the detection of pancreatic insoulinomas.

Ten patients with insoulinomas, surgically verified, underwent preoperatively dynamic contrast enhanced CT, DSA MRI at 1.5 T and 3D time of flight MR arteriography of the upper abdomen.
The MR images included T1 WSE, T2 WSE, T1 WSE fat suppression and dynamic pre and post contrast GRE sequences. CT did not depict four tumors that were small ( less than 2.5 cm) or avascular on DSA. DSA was negative in two cases. MRI showed nine of the ten tumors. MRA showed five tumors as bright foci, two of them having been missed on DSA.

We conclude that MRI combined with MRA should be regarded as the most accurate method for detection and localization of the insoulinomas.

### F173

### INSULINOMAS ASSOCIATED WITH MULTIPLE ENDOCRINE NEOPLASIA

Emm. Bandouvas, M. Paraskevopoulou, J. Alexandridis A. Alexandridi, I. Parharidou, E. Papaefthimiou, I. Kapelakis, G. Giannopoulos, D. Tsabrinou, K. Anastasiou.
"Helena Venizelou" Hospital, Athens, Greece

Insulinomas are usually solitary (greater than 90%) benign pancreatic tumors readily cured by enucleation or resection. To determine whether the 4% of insulinomas associated with multiple endocrine neoplasia type I (MEN-I) require a different surgical approach. We analyzed our experience in 2 patients with MEN-I insulinomas treated during the past 30 years, associated with 43 patients reported in the English literature. However, more than 1,000 cases have been reported in the world literature. Both patients with MEN-I insulinomas were associated with an antecedent history of other endocrinopathy of a family history of MEN-I, allowing preoperative identification of these patients. Both patients had hyperparathyroidism and one had pituitary tumors. The insulinomas were usually multiple (median 3, range 1 to 14). Distal subtotal pancreatectomy with enucleation of any tumors identified in the head of the gland was done in both our patients. One is now normoglycemic and one is diabetic.

### F174

### PANCREATIC INSULINOMA - SURGICAL TREATMENT

Z.Wajda, Z.Gruca, E.Boj, M.Dżoga-Litwinowicz, Z.Śledziński, J.Głowacki, Ślisz-Jaromczyk

II Department of Surgery, Medical University of Gdańsk Poland

The authors reports on a study of 13 patients with diagnosis of pancreatic insulinoma. All patients had incidents of hypoglycemia more than 40 mg%. The diagnostic procedures included CT-scan, caeliac arteriography and ultrasound examinations. 3 lesions were localised in head of pancreas, 5 in body and 2 in tail of the gland. In 3 patients hypertrophy or adenomatosis in the tail of pancreas was revealed in post-operative histological examination. Ten patients with palpable tumours underwent local excision of the tumours. In 2 patients left pancreatectomy and in 1 subtotal pancreatectomy were performed. No serious complication was noted due to surgical procedures. After surgery each patients had normal blood glucose level and none incident of hypoglycemia was observed. In conclusion these data confirm that local excision of the tumours or blind resection of the pancreas when the lesion is undetectable are resouable operative procedures in treatment of pancreatic insulinomas.

### F175

### SURGICAL TREATMENT OF INSULINOMAS.

E.Hadjiyannakis, M.Mitrakou, S.Drakopoulos, N.Georgopoulos, A.Poultsidi, S.Mathioulakis, N.Nikitakis, S.Tselios, K.Anastasakou, S.Raptis.

1st Surgical Department and Transplant Unit - "Evagelismos" Hospital of Athens.

Perioperative mortality was 0.

Postoperative follow-up was up to 8 months:

BMI in 4-8 months was 29 ± 3 Kgr/m², HbA1C : 5,6 ± 0,2%.

Fasting plasma glucose levels were 4,9 ± 0,2 mmoles and fasting insulin levels 72 ± 10 pmol.

### F176

### SURGICAL MANAGEMENT OF PANCREATIC PSEUDOCYSTS

Antonopoulos K, Simatos G, Athanasiou M, Staurou A, Dimitracopoulos G, Markidis P, Poulantzas J.

4th Surgical department of "Evangelismos" Hospital, Athens Greece.

During the last 12 years (1982-1993) 41 cases of pancreatic pseudocysts were managed surgically in the 4th Surgical Department of "Evangelismos" Hospital.
They were 21 men and 20 women with a mean age of 55,1 years.
The causes were: acute gallstone pancreatitis (65,8%), acute alcoholic pancreatitis (4,9%) trauma (12,2%), chronic pancreatitis (9,8%), unspecified (7,3%). The mean diameter of the cysts was 7,4 cm. Three patients (7,3%) underwent peripheral pancreatectomy out of whom in one with two cysts external drainage was also performed. Seven patients (17%) were treated with external drainage, fifteen patients (36,6%) with cystogastrostomy and sixteen (39,9%) with cystojejunostomy.
There was one perioperative death (2,44%) from unrelated causes.
Major complications were: for the group of peripheral pancreatectomy pancreatic fistula (for the patient to whom external drainage was also performed), persistent pancreatic fistula for the group of external drainage (2/7 cases 28,5%) and for the cystogastrostomy group gastric haemorrhage (3/15 cases 20%). One of the patients with gastric haemorrhage needed reoperation. All the other complications were managed conservatively. No major complications were observed in the cystojejunostomy group. Follow up data were available for 33 patients for 1-11 years. There was one recurrence for the external drainage group (16,6%). In conclusion gastrojejunostomy is the safest and the most efficient procedure for the surgical management of pancreatic pseudocysts. External drainage procedures should be saved for selected cases especially those complicated with infection.

### F177

SURGICAL TREATMENT OF PANCREATIC PSEUDOCYSTIS- IMPLICATIONS-RESULTS

P.Capsambelis, K.Tsangaropoulos, E.Palli, G.Panagopoulos, D.Aktipis, P.Stravopodis, G.Panopoulos
Department of Surgery ,General Hospital of Zante, Greece.

Our purpose is to introduce to you the results and surgical experience from the surgery of pseudocystis in pancreas as a complication of acute pancreatitis. Our sample is consisted of 8 patients (5M-3W) who we operated in the last decade (1984-1994). The age average was 55 years. Seven pseudocystis were unilocular and one polilocular. The pseudocystis were the result of acute pancreatitis from gall blader lithiasis in 6 patients, chronic alcoholic pancreatitis in one patient, and unknown cause in another. The outcome was satisfactory. In six patients we applied posterior intragastric cysteostomy, in one patient Roux-en Y anastomosis with the jejunum and in one external drainage. We noticed one major post-operating complication(bleading) and re-operation.Concluding we infer that the Internal drainage of pseudocystis, no matter the method, allows radical and secure treatment with no major post-operating problems.-

### F178

OUR EXPERIENCE OF 408 OPERATIONS IN CYSTIC PANCREATIC LEASIONS

V.D.Fedorov, M.V.Danilov, V.P.Glabay, I.M.Buriev
A.V.Vishnevsky Institute of Surgery,Moscow,Russia

408 patients with cystic pancreatic leasions (CPL) were operated on from 1976 to 1994. The true cysts were occured the most rarely (6 patients - 1,5%); the optimal method of operation was distal pancreatectomy (DP) in 5, spleen was preserved in 3, the results of this procedure have prooved to be good. 134 patients (32,8%) were operated on for postnecrotising pseudocysts due tocholangiogenic or posttraumatic pancreatitis without pancreatic duct hypertension (mortality - 0,7%);the DP was used in 17 patients, external cystic drainage in 25, the others 92 operations when cyst was more often found out in the head of the gland were cystojejunostomy (76) and cystogastrostomy (16), the secondary operations for cyst reccurense or late complications were needed in 18 (15,9%). Mostly (in 227 patients-55,6%) the operations for primary chronic pancreatitis with pseudocysts and dilated pancreatic ducts were carried out. Operations included pancreatic ducts drainage in 91 cases: longitudinal pancreatojejunostomy (LPJS) -57, DP+LPJS -25, Whipple operation - 9 with general mortality of 2,2% and secondary operations for reccurence of the disease of 6,8%. 136 patients requared isolated internal cyst drainage (mortality -7,3%,secondary operations - 29,2%).41 patients(10,1%)were operated on for cystic pancreatic tumors (benigh-19,malignant - 22), resect were performed in 39 without fatal outcome,the 5-year survival rate was 100% in benigh and 88,2% in malignant cystic tumors.In conclusion:the favourable results could be achieve by using the different surgical approach depended on type of CPL.

### F179

LONG-TERM FOLLOW UP OF 70 PATIENTS WITH PANCREATIC PSEUDOCYST MANAGEMENT BY SUBTOTAL RESECTION

M.Jesipowicz, S.Rudzki, S.Stettner, J.Jesipowicz
The First Department of General Surgery
Medical Academy in Lublin, Poland

An estimated occurrence of the pancreatic pseudocyst varies from 0,004% to 0,01% in hospitalized patients with a tendency towards growth proportionaly to an increasing occurrance of acute and chronic pancreatitis. Approximately 2% to 10% of the patients with acute pancreatitis develop clinically apparent cyst. The aim of the present study is to analyse, in detail, long-term results of different ways of surgical treatment of the pancreatic cyst in the light of the experience of the First Department of General Surgery in Lublin. Clinical diagnosis and follow up were achieved by using radiological methods, including vascular examination, initial USG and then USG applied repeatedly, and the most precise CT. In our opinion ERCP or intraoperative pancreatography can be chosen to determine the kind of operation. In the years 1975-1994 there were 107 patients with pancreatic pseudocystic operated on. In 18 cases various types of internal anastomosis were performed. In 10 external drainage was applied. In these groups the long-term results were followed up in 9 patients. There were observed: recurrence of cysts in 3 cases, chronic pancreatitis in 2 cases and one fistula pancreatis. There were 79 patients operated on using the method combining both total cystectomy as well as excision of changed pancreatic parenchyma and pathologically changed surroundings. In 70 patients, out of 79, long-term results were assessed. No recurrance of cyst were observed in this group. In 2 cases diabetes was found out. On the basic of the authors experience it can be concluded that the prefered method of resection of pseudocystis together with pathologically changed pancreas has some advantages over the traditional ones.

### F180

LONG-TERM RESULTS IN THE THERAPY OF PANCREATIC PSEUDOCYSTS

E. Yekebas° G. Fröschle°, H. Seiffert*, K. Binmöller*, J. Izbicki°, C.E. Broelsch°
Department of General Surgery(°) and Department of Surgical Endoscopy(*), University of Hamburg, Federal Repulik of Germany

Introduction: Pancreatic pseudocysts are a complication arising from acute or chronic pancreatitis. Post-acute pseudocysts disappear in some cases spontaneously whereas the latter rarely do.

Materials and Methods: In a ten-year period from 1985-1994, a total of 187 patients with pancreatic pseudocysts were treated and followed up either in the department of general surgery or in the department of endoscopic surgery at the University of Hamburg. We investigated the outcome of surgical, endoscopic or percutaneous techniques. The average age of the patients was 46 years (range 17-74). Mean follow-up was 29 months. Post-acute pseudocysts were observed in 41 % (n=77) of the patients, while 59 % suffered from chronic disease. Alcohol was the leading etiology in 53 % (n=99), followed by a preceeding biliary pancreatitis in 35 % (n=65). In 11 % (n=21) the etiology was unclear, including 6 cases of anatomical variations (pancreas divisum, common channel, juxtapapillary diverticula). The localization was in 43 % in the head, in 24 % in the body and in 32% in the tail. The mean size of the pseudocysts was 13 cm (range 1,2-38). All cysts were present for at least 2 months.

Results: 50 % (n=94) of the patients were treated surgically. 37 % underwent interventional endoscopic procedures and in 4 % (n=8) a percutaneous drainage was performed. A spontaneous regression was observed in 8 %. Emergency laparotomies had to be done in 21 patients. Severe complications after surgery occured in 16 %, peri-operative mortality was 6 %. Complications following endoscopic procedures could be observerd in 20 %. Most of them could be treated endoscopically but in 10 patients complications were so severe that the patients had to be operated. 75 % of all patients with post-acute cysts had been free of symptoms after any form of therapy. In the treatment of chronic pseudocysts non-surgical procedures were much less successful. Only 33 % showed a long-term improvement of their clinical symptoms or were free of symptoms as cysts recurred frequently as soon as endoscopic or percutaneous therapy discontinued. In contrast, 62 % of the patients with chronic pseudocysts treated surgically were definitely free of symptoms.

Conclusion.: In post-acute pancreatic pseudocysts there are only small differences in complaints for surgical or non-operative treatments. In contrast, surgical treatment is more successfull in chronic pseudocysts.

### F181

LIVER RETRANSPLANTATION. 67 CASES EXPERIENCE.

E. Moreno-González, A. Alvarado, C. Loinaz, R. Gómez, I. González-Pinto, I. García, C. Jiménez, M. Musella, C. Castellon.

Liver Transplantation Unit. University Hospital "Doce de Octubre", Madrid, SPAIN.

One unoubtedly significant factor in the improved results of liver transplant constitutes the most common indication of retransplantation, since progrssively and severely the graft function worsen, the only chance to save the patient life is liver retransplantation. Report our experience with 67 cases.
PATIENTS AND METHODS. In the period between April 1986 to November 1994, 402 liver transplants heve been performed in the "Doce de Octubre" hospital, 67 were retransplants: 48 adults and 19 children. In 54 cases of second graft-first retransplant (41 adults, 13 children), 12 cases of third graft-second retransplant (6 adults, 6 children), and 1 case of fourth graft-third retransplant (1 adult). Primary non function and chronic rejection represent the 70% of indications of retransplant. From 67 grafts, 12 were reduced size liver and the remainder whole grafts.
RESULTS. The peroperative mortality represent 22,2%, 12 patients (3 intraoperative, 9 in the first 28 days), in the follow up dead 13 patients (24%), for overall mortality of 25 patients (46,29%). The actuarial overall survival, excluding the peroperative mortality are: 85,5% at first year, 71,6% at third y, and 59,4% at fifth y. The survival of first grafts are 35,1% at six months, 20,3% at first y, 11,1% at second y, 3% at third y, and 0% at fourth y. The survival of second grafts are: 53,1% at first y, 46,4% at third y, 42,2% at fifth y.
COMMENTS. The present results suggests that hepatic retransplant is a valid option when is required as only chnace of treatment. The main point is that the indication have to be accurate. A third or fourth transplant can be performed with good results. The long term survival is similar to the patients evolution with one only transplant.

### F183

RESECTION OR LIVER TRANSPLANTATION FOR TREATMENT OF HEPATOCELLULAR CARCINOMA IN CIRRHOTIC LIVER.

J Balsells, V Vargas, R Charco, LL Castells, JE Murio, JL Lázaro, R Esteban, C Margarit.
Liver Transplantation Unit and Hepatology Unit. Hospital Vall d'Hebron. Barcelona. Spain

Partial liver resection (LR) or liver transplant (LTX) are the potential curative treatments of hepatocellular carcinoma (HCC). The aim of the study is to compare the survival and the survival free of tumor between both groups of treatments.
Seventy-eigth cirrhotic patients with HCC were included, 53 treated by LR and 25 by LTX. Alpha-fetoprotein measurements and imaging studies were performed every 4 months in order to detect recurrence of HCC. Actuarial survival and free-tumor survival rates were estimated by the Kaplan-Meier method and the log-rank test.
Postoperative and follow-up deaths occurred in 7 and 29 patients respectively for LR, and in 2 and 6 patients respectively for LTX. Actuarial survival rates at 1 and 2 years were 61.8% and 46.9% respectively for LR, and 73.7% and 50.7% respectively for LTX ($p=0.2$). Tumoral recurrence was detected in 24 from 46 patients treated by LR (52.2%), and in one patient from 23 (4.3%) cases of LTX ($p<0.001$). Free-tumor survival rates at 1 and 2 years were 46.9% and 28.3% respectively for LR, and 67.8% and 50.8% respectively for LTX ($p<0.03$).
Conclusion: LR had a high rate of recurrence. Patients treated by LTX had a higher survival than patients with LR. LTX should be considered as the first choice of treatment of HCC in cirrhotic patients.

### F182

EXTRA-ANATOMIC VENOUS GRAFTS IN PORTAL THROMBOSIS IN LIVER TRANSPLANTATION.

J.Figueras, J. Torras, A.Rafecas, J.Fabregat, E.Ramos, B.de Ramon, A.Montserrat and E. Jaurrieta.
Department Surgery. Hospital Bellvitge. University Barcelona. Spain.

The aim of this study is to analyze the mortality and morbidity of liver transplantation (OLT) in portal vein thrombosis (PVT) with extra-anatomic venous grafts reconstruction. From 1984 to now, 254 OLT were carried in our unit. Venous conduits to reconstruct a chronic PVT were performed in 8 patients during the two last years. Three were retrasplants (ReOLT). The end of the venous graft was brought anteriorly to the pancreas and retrogastric and it was anastomosed to the anterior face of the SMV, at the root of mesentery. Operative data and blood transfusion were compared with 42 simultaneous OLT performed during the same period. Operative time was longer in the OLT with venous grafts: 579±95 vs 495±104 ($p<0.05$). The anhepatic phase: 97.5±49 vs 78±33, and the requeriment of blood transfusion : 20.6±11 vs 15.8±7 P.R.C.U. were similar in both groups. One patient presented early venous graft thrombosis (36 hours) due to compresive haematoma in the conduit route. Thrombectomy and reanastomosis was performed with good outcome. Intraoperative mortality was nul, the 3 ReOLT patients died in the follow-up, one in the 9th postoperative day due to invasive candidemia, and the others died in the 5th and 6th months from unrelated causes. The necropsic studies demonstrated portal and arterial patency. With a follow-up of 2 to 16 months the five survivors maintain good liver function and the portal vein patency has been confirmed.
Conclusions: 1.- Portal thrombosis should not be considered a contraindication for OLT. 2.- In the chronic portal thrombosis, the extra-anatomic venous graft conduit is feasible, no increases the anhepatic phase, nor the requeriments of blood transfusion.

### F184

NEOPTERIN - AN EARLY INDICATOR OF COMPLICATIONS AFTER LIVER TRANSPLANTATION

A.R. Mueller, K.-P. Platz, M. Partow, M. Postels, U. Kaisers, P. Neuhaus.
Department of Surgery, University Clinic Rudolf Virchow, Germany

The monitoring after liver transplantation not always allows a safe diagnosis between various diseases. New parameters which may select patients at risk early after transplantation are desirable. Neopterin an intermediate of the tetrahydrobiopterin pathway is produced by macrophages which were activated by IFN-$\gamma$ and other cytokines.
Between August 1993 and March 1994, neopterin was determined in 55 liver transplant recipients at pre-defined time points: prior to transplantation, prior to hepatectomy, prior to reperfusion, and 15 min, 2, 6, 12, 18, 24, 36, 48 and 72 h after reperfusion, and subsequently on a daily basis until POD 28 or until complications have been resolved (normal range: 6.7±1.2 nmol/l; n=40).
Irrespective of the quality of graft function as assessed by transaminases, bile color and amount of bile production, neopterin levels were low (21.8±1.2 nmol/l; p=n.s. vs normal) in patients with an uneventful postoperative course. Neopterin levels increased significantly within the early time period after reperfusion in patients who subsequently (between POD 6 and 15) developed acute rejections (54.7±1.5 nmol/l; $p\leq0.05$ vs normal). A further increase in neopterin levels was observed within 72 h after reperfusion in patients who subsequently developed serious complications, including life-threatening infections and graft failure (143.9±4.8 nmol/l; $p\leq0.05$ vs patients with an uneventful postoperative course). During rejection episodes, neopterin levels increased to 88.3±3.9 nmol/l ($p\leq0.05$ vs no rejection). However, in patients with serious complications, neopterin levels reached 246.7±6.2 nmol/l ($p\leq0.05$ vs all remaining groups).
We conclude that neopterin is an early indicator for subsequent complications after liver transplantation. Measurement of increased neopterin levels may lead to intensified monitoring in critical patients.

### F185

COMBINED LIVER AND RENAL TRANSPLANTATION FOR POLYCYSTIC LIVER AND KIDNEY DISEASE

E.A. Antoniou, L.J. Buist, Y. Nishimura, J.A.C. Buckels, D.A. Mayer
Liver Unit, Queen Elizabeth Hospital, Birmingham, U.K.

A polycystic liver is found in about half the patients with polycystic kidney disease but usually the renal disease predominates. Occassionally, as in the case presented here, end stage hepatic failure becomes an indication for organ transplantation.

A 55 year old lady with a 20 year history of deteriorating renal function due to polycystic disease and 9 year history of symptomatic multiple liver cysts was referred for transplantation. She had a family history of polycystic disease. Her hepatic disease presented with ascites and spontaneous bacterial peritonitis and symptoms over the following years were of abdominal discomfort from an enlarging liver. On referral she had been jaundiced for 4 weeks with increasing lethargy and generalised weakness. Her abdomen was distended by a grossly enlarged liver. Her bilirubin measurement was 278mmol/l, alkaline phosphatase 2234mmol/l, AST 101mmol/l and albumin 31mmol/l. Renal function was significantly impaired (urea 27.9mmol/l, creatinine 506mmol/l). Radiological investigations revealed the IVC was patent but compressed in the intrahepatic portion with the hepatic veins indented by multiple cysts.

On 19/12/94 she received a liver and kidney transplant. Her removed liver weighed more than 8Kg. and was composed completely of cysts. There was relative sparing of the caudate lobe which was hypertrophied. The right kidney, which was removed to produce space to accommodate a new kidney, measured 15cm in length and was polycystic. The liver graft was placed orthotopically and the kidney implanted retroperitoneally in the right iliac fossa. She made a satisfactory recovery and was discharged home on the 14th postoperative day. At eight weeks post transplant she is well with good renal and hepatic function.

From this experience we advocate combined liver and kidney transplantation for patients with hepatic and renal polycystic disease who develop end stage liver disease with renal impairment.

### F186

PERCUTANEOUS ANGIOPLASTY IN ORTHOTOPIC LIVER TRANSPLANTATION

G. Berenhauser Leite, Valéria, I.F.S.F. Boin, L.S. Leonardi
Unit Liver Transplantation, State University of Campinas, Campinas-SP, Brazil

We showed two cases with stenosis of the anastomosis of the supra hepatic cava vein (SHCV).

Case nº 1: 41 years old, male, HCV ●; 21$^{th}$ after OLT a evaluation of the patient we observed anasarca and renal insufficiency and the inferior cavagram presented 70% stenosis of the SHCV. The treatment was percutaneous angioplasty with two ballons simultaneously (15 and 20mm of diameter). During the follow-up observed reduction in stenosis grade with 15% stenosis residual. The evolution of this patient is very good and one year after OLT is with chronic rejection.

Case nº 2: 21 years old, male, with Budd-Chiari syndrome, his evolution during immediate postoperative period presented graft dysfunction and legs edema and upper digestive hemorrhage on 23th day. The cavogram on 31th day showed stenosis of SHCV (60%), the treatment was percutaneous angioplasty with two balloons simultaneously (18mm/diameter), actually the patient is without symptoms 18 months after OLT.

The percutaneous angioplasty can to resolve stenosis of the vessels anastomosis with injury to the liver graft.

### F187

**INTERCELLULAR ADHESION MOLECULE-I EXPRESSION ON BILE DUCTS:**
**A DIAGNOSTIC MARKER OF ACUTE CELLULAR REJECTION**

**A. Bhargava[1], N. J. Bradley[1], A. K. Burroughs[2], A. P. Dhillon[3] K. Rolles[1], B. R. Davidson[1].**
**[1]University Department of Surgery, [2]Liver Transplantation Unit and [3]Department of Histopathology**
**Royal Free Hospital School of Medicine, Pond Street, London NW3 2QG UK.**

Acute cellular rejection (ACR) affects 75-80% of liver allograft recipients. Persistent episodes of rejection may be associated with chronic rejection and graft loss. Intercellular adhesion molecule-1 (ICAM-1) an inducible cell adhesion molecule involved in leukocyte adhesion pathways.

The aim of this study was to investigate the expression of ICAM-1 liver allografts post-operatively and correlate expression with histolologically diagnosed ACR.

Protocol biopsies usually taken on days 5,10,15 and 20 for routine histology were stained immunohistochemically for ICAM-1. 5μm frozen sections of 83 biopsies from 32 patients were analysed by light microscopy and compared to normal liver. The staining intensity on bile ducts, endothelium, sinusoids and hepatocytes was assessed blindly.

De novo staining of bile ducts was noted and compared to morphological diagnosis. 21/32 patients, were diagnosed as ACR on at least more than one occasion. Positive staining bile ducts with ICAM-1 were seen in 18/21 recipients diagnosed as ACR irrespective of grade.

Five recipients had persistent acute cellular rejection: all five had persistence of bile duct staining for ICAM-1. Patients were treated with 3 pulse doses of methylprednisolone for ACR. Those showing histological improvement had negative staining bile ducts for ICAM-1.

ICAM-1 is a useful marker in the diagnosis of ACR and may be used to ascertain efficacy of treatment. The bile duct is the main target of destruction in ACR. Expression of ICAM-1 on bile ducts may result in lymphocyte homing to the bile duct. Monoclonal antibodies to prevent ICAM-1 expression may be a novel method to reduce the affects of acute cellular rejection especially in those recipients with steroid resistant rejection.

### F188

ORTHOTOPIC LIVER TRANSPLANTATION FOR ALCOHOLIC LIVER CIRRHOSIS.

L BAULIEUX, E. DE LA ROCHE, M. ADHAM, C. DUCERF, M. POUYET.
Service de Chirurgie Digestive et de la Transplantation Hépatique - Hôpital de la Croix Rousse Lyon 69004.

From January 1985 & January 1994, 215 orthotopic liver transplantation (OLT) was performed. Among them, 48 patients (40 men & 8 women) aging 18 - 62 years (mean 46 years) received OLT for alcoholic cirrhosisi (AC). 16 patients had coexisting viral infection B - C - D - E. 3 patients had associated hepatocellular carcinoma (HCC). All patients had been classified "Child C" during their preoperative follow up, at the time of OLT their were : Child A n=5; Child B n=28; Child C n=15. These 48 patients received 53 OLT. 5 retransplantations were performed for : 4 primary nonfunction, 1 arterial thrombosis. Postoperative mortality was 14% (5 cases), late mortality had concerned 5 patients (3 recurrence of HCC, 1 hepatitis B recurrence, 1 chronic rejection). 3 years actuarial survival was 77%. Alcoholic ingestion after OLT was recorded for 12,5%. Social activity was normal in 80% of cases & 50% returned to work within 15 months (mean 7,24 month). OLT for AC could be proposed in selected cases : young patients, severe liver cirrhosis, no alcoholic consumption for 6 months before OLT, favorable socio-familial environment & absence of malignancy.

### F189

### LIVER TRANSPLANTATION FOR ACUTE LIVER FAILURE: ARE PROGNOSTIC FACTORS USEFUL IN THE DECISION MAKING PROCESS ?

*J.Van de Stadt, N.Bourgeois, M.Adler, M.Gelin.*
Department of Digestive Surgery and Gastroenterology, Erasme hospital, Brussels, Belgium.

In acute liver failure, prognostic criteria have been proposed in order to determine in right time which patient will need a liver transplantation. We have reviewed our last 31 patients with acute liver failure and retrospectively tested the London's prognostic factors. The sex ratio was M/F=11/20, and the mean age 38 (14 - 77). The etiology was as currently reported in continental western Europe, with 71% viral hepatitis (A 10%, B 26%, C or non-A non-B 35%), 26% drug- or toxic-induced hepatitis (including 3 cases (10%) of acetaminophen overdose) and 1 acute Wilson disease . Twenty-six patients developed encephalopathy with a fulminant course (< 2 weeks from jaundice) in 16 and a subfulminant course (> 2 weeks from jaundice) in 10. Twenty-four patients developed the risk factors as described by the King's College team in London: 11 were transplanted, 12 died and only 1 survived. Seven patients never developed these risk criteria: 6 survived and 1 died. Out of the 13 patients who died, the risk factors were present >48h before death in 6, 6 were admitted less than 36h before death and 1 did not present the risk criteria. In our experience, the survival rate is 86% (6/7) in patients not considered for liver transplantation, 8% (1/13) in patients considered for transplantation but not transplanted, and 64% (7/11) 1-year survival in liver transplanted patients.
*Conclusions:* Liver transplantation in emergency remains the best treatment for far-advanced acute liver failure. Prognostic indicators are helpful in making the decision for transplantation. In fulminant presentations, quick patient referal, evaluation and treatment is the key to success.

### F190

### APPRAISAL OF THE ORDER OF REVASCULARISATION IN HUMAN LIVER GRAFTING: A CONTROLLED STUDY.

R. Noun, J. Belghiti, F. Durand*, A. Sauvanet
Departments of Digestive Surgery & *Hepatology, Hôpital Beaujon, F - 92110 Clichy.

Although experimental studies showed no detrimental effects of primary arterialisation, this order of revascularisation had neither been frequently used, nor investigated in clinical transplants. We routinely use a technical procedure that allows either initial arterial revascularisation (IAR) or initial portal revascularisation (IPR) under hemodynamic stability. The aim of this controlled study was to compare the effects of graft revascularisation by IAR versus IPR.
Patients: From November 1992 to December 1993, 29 patients undergoing an elective or an emergent liver transplantation were included. During this period, uniform protocols for liver allografts storage and immunosuppression were followed. The procedure included preservation of caval flow in combination with a temporary portacaval shunt. During the anhepatic phase, patients were divided in IAR group (n=15) and IPR group (n=14) and were homogeneous according to type of liver disease, age of donors (37±12 vs 38±13 yrs), preservation time (501± 226 vs 505±248 min) and mean arterial pressure during the anhepatic phase (89±10 vs 87±11mmHg). Post reperfusion syndrome was defined as a fall in mean arterial pressure > 20% below the baseline value for at least 1 min, occurring within the first 5 min after reperfusion.
Results: Post reperfusion syndrome occurred in 4/15 (26%) patients with IAR as compared to 6/15 (42%) in IPR group (NS). Macroscopic perfusion aspect was judged as uniform and diffuse in all the livers that received IAR as compared to 10 (71%) with IPR (p<0.05). Mean operative duration was significantly shorter in the initial arterial group (472±11vs 590±83 min)(p<0.05). Postoperative ASAT and clotting factor V levels in the IAR vs IPR were (722±396 vs 675±529 IU) (46±26 vs 53±20 %) and (311±233 vs 361±333 IU) (84±24 vs 97±24%) at day 1 and 5 respectively (NS).
Conclusion: Although advantages of IAR were limited to the appearance of the liver and to the duration of the procedure, the absense of apparent detrimental effect supports that IAR could be liberally undertaken. Patients with a previous portasystemic shunt would represent the best candidates.

### F191

### CLINICAL FEATURES AND MANAGEMENT OF PORTAL VENOUS OBSTRUCTION FOLLOWING ORTHOTOPIC LIVER TRANSPLANTATION.

**B. Malassagne, O.Soubrane, B.Dousset, Y. Calmus, P.Legmann, O.Bernard, D.Houssin, Y.Chapuis.**
**Clinique Chirurgicale, Hôpital Cochin, Paris, France.**

Even though portal venous obstruction is considered a rare but severe event following liver transplantation, it has been little evaluated. The aim of this work was to assess the clinical features and prognostic factors of this complication and to propose an adequate strategy for therapy.
This report is a retrospective study of 13 adults and 8 children who experienced a portal venous obstruction among 338 liver graft recipients over a 9-year period.
Portal venous obstruction occurred at a median time of 17 (0-1463) days after liver transplantation. The causes of obstruction were: thrombosis (n=12), stenosis (n=6), or twist (n=1) of the portal vein, and a localized portal hypertension (n=2). Pretransplant risk factors identified using univariate analysis were the following: portal vein thrombosis (p<0.01) or hypoplasia (p<0.01) and a portasystemic shunt (p<0.001). Clinical manifestations were related both to the mechanism and date ot occurence of portal venous obstruction. Early after liver transplantation (<30 days), a liver graft failure (n=7) due to portal vein thrombosis predominated over the manifestations of portal hypertension. Later in the post-transplant course, isolated symptoms of portal hypertension without liver dysfunction were observed (n=14) and variceal bleeding was the main life-threatening complication. Therapeutic procedures were: retransplantation (n=3), vascular reconstruction (n=5), left colectomy (n=1), splenectomy (n=1), portal vein dilatation (n=3) or no specific treatment (n=8). Overall mortality rate was 10/21 (47%). Six patients out of seven who experienced early portal vein thrombosis died from graft failure. Four patients out of fourteen whith complications of portal hypertension died. One died from variceal bleeding and three died from causes which were not related to portal hypertension.
In conclusion, this study underlined the high incidence and the severity of portal venous obstruction after liver transplantation. Successful management required an early diagnosis and the precise identification of the mechanism of the portal obstruction. Urgent retransplantation is recommended in case of graft failure whereas in absence of hepatic dysfunction, conservative procedures can be proposed according to the mechanism of portal venous obstruction and to patient's condition.

### F192

### THE USE OF THE RECIPIENT SPLENIC ARTERY FOR ARTERIAL RECONSTRUCTION IN CLINICAL LIVER TRANSPLANTATION.

A. Rafecas, J. Fabregat, J. Figueras, J. Torras, B. Ramon, A. Montserrat, E. Ramos and E. Jaurrieta.
Liver Transplant Unit. Hospital Bellvitge. University of Barcelona. Spain

We report an alternative technique in which the recipient splenic artery (SA) is used for arterialization of hepatic grafts when recipient's hepatic artery (HA) was inadequate.
PATIENTS AND METHODS: Between February 1984 and November 1994, 254 OLT were performed in our unit. In eleven cases, all of them cirrhotic with a large SA and an inadequate HA, arterialization of the graft was achieved by anastomosis to the recipient SA. In 3 cases the HA was inadequate due to anatomic abnormalities, in 6 due to HA thrombosis after OLT and in 2 due to chronic rejection (8 retransplants). The SA was approached through the lesser omentum, dissected free from the upper edge of the pancreas over a distance of 4 cm, starting near its origin. Thereafter, the SA was clamped proximally, ligated at the distal end, and divided. The proximal end of the SA was anastomosed to the donor celiac trunk.
RESULTS: there were two postoperative deaths (one patient died 58 days after OLT due to brain damage and sepsis, and the other one died 60 days after OLT due to CMV disease). One patient died seven months after retransplantation due to recurrence of HCV infection. In the three cases the autopsy showed a patent arterial anastomosis. Eigth patients (73%) are alive with a mean follow-up of 24 months (range 12-48). Normal HA inflow was confirmed by doppler ultrasound in all cases. There were no complications related to the technique, and especially no splenic infarction, clinical pancreatitis or hepatic arterial thrombosis.
CONCLUSIONS: Use of the SA as inflow in OLT is simple, requires only minimal additional dissection, and should be considered an effective alternative in cases where the HA is inadequate for arterialization of the graft. We suggested this technique in the cases with inadequate native HA and where there is an enlargemennt of SA with splenomegaly.

### F193

LIVER TRANSPLANT IN PATIENTS WITH PREVIOUS SURGERY FOR PORTAL HYPERTENSION.

Murio, J.E., Lázaro, J.L., Charco, R., Balsells, J., Bilbao, I., Gifre, E., Ruiz, C., Margarit, C.
Liver Transplant Unit. Hospital General Universitario Vall d'Hebron. Barcelona, SPAIN.

From January 1991 we have performed 14 liver transplants (OLT) in patients with previous surgery for portal hypertension (10 end-to-side portocaval shunt (PC), 2 side-to-side PC, 1 Warren's shunt and 1 proximal splenorenal shunt). Liver transplant was indicated for end-stage liver failure due to postnecrotic liver cirrhosis in 9 cases, liver cirrhosis and hepatoma in 2, alcoholic liver cirrhosis in 2 and criptogenetic liver cirrhosis with portocaval shunt acute thrombosis in 1. Mean interval between shunt and OLT was 82.3 months. In 12 cases the piggy-back technique was utilized. A venovenous by-pass was utilized in the first case as well as when acute thrombosis of PC. Portosystemic shunt made. easier the hepatectomy phase due to abscense of portal hypertension. During dismantling PC a splacnic congestion was noted compeling to close abdominal wall with a prosthetic mesh in one case. Mean transfusion was 6.7 units. There was not operatory mortality. Clinical course was uneventful in 12 cases. A patient presented hyperacute rejection and was retransplanted. Patient with acute shunt thrombosis was technically demanding due to severe portal hypertension. After a mean follow-up of 20 months (range 4-35) 4 patients died secondary to cholangitis and sepsis in 1 case, and hepatitis C virus recurrence in 3.
In our experience previous surgery for portal hypertension is not at all a contraindication to OLT and we believe that piggy-back technique is particularly useful.

### F194

**IS RETRANSPLANTATION AVOIDABLE AFTER ARTERIAL THROMBOSIS OF THE LIVER GRAFT?**

**O.Soubrane, J.Cardoso, B.Dousset, O.Bernard, D.Houssin, Y.Chapuis.**
**Clinique Chirurgicale, Hôpital Cochin, Paris, France.**

Arterial thrombosis of the hepatic graft is one of the most severe complications of liver transplantation (LT) leading to parenchymal and biliary ischemia, often requiring urgent retransplantation (reLT). The aim of this retrospective study was to precise the prognosis of recipients who experienced hepatic artery thrombosis (HAT) and to define the cases where reLT could be avoided.

From 1985 to 1994, 435 LT have been performed in 220 adults and 160 children. 16 HAT occurred in 13 children (8.6%) and 12 HAT occurred in 12 adults (4.7%). Two groups have been identified: group A of 10 patients who underwent 13 reLT and group B of 15 patients who were not retransplanted.

In group A, 5 patients were retransplanted in emergency (8reLT) because of acute hepatic insufficiency after HAT occurring less than 7 days after LT. One of these reLT has been performed 20 hours after total hepatectomy of the primary graft (two stage procedure). The 5 other reLT have been done in elective condition because of diffuse ischemic damage of the liver graft after failure of conservative therapy (partial hepatic resection and/or biliary reconstruction). In group A, patient survival rate was 50%. In group B, 4 adult patients died from sepsis after either partial hepatectomy (n=1), percutaneous (n=2) or surgical (n=2) treatment of ischemic cholangitis. The 11 other patients (8 children, 3 adults) are alive (73%) after a median follow-up of 33 months (range: 4-92). All these patients have been reoperated on : partial hepatic resection for liver abcess (n=5), biliary reconstruction (n=12), percutaneous drainage (n=4) and transhepatic dilatation of biliary stricture (n=1). At the time of last follow-up, 6 patients exhibited marked alteration of liver biochemical tests, 4 had portal fibrosis at histological examination of liver biopsy, 3 still had persistant biliary sepsis, and 1 had portal hypertension.

In conclusion, when HAT occurs after LT, reLT is mandatory in case of acute liver graft failure. On the other hand, when liver graft function is satisfactory, a conservative treatment is recommended. Agressive therapy of parenchymal and biliary ischemia allows either to obtain prolonged survival with a functional graft or to delay reLT which can thus be performed in elective condition.

### F195

**LIVER TRANSPLANTATION FOR BUDD-CHIARI SYNDROME AFTER FAILURE OF PORTOSYSTEMIC SHUNT**

**PP. Massault, B. Dousset, O. Soubrane, Y. Ozier, D. Houssin, Y. Chapuis**
**(Department of Surgery, Hôpital Cochin, Paris, France)**

Liver transplantation (OLT) for Budd-Chiari syndrome (BCS) as a first-step treatment is debatable, in view of the satisfactory results of portosystemic shunt and the risks of disease recurrence. We report our experience of OLT in five patients with BCS after failure of portosystemic shunt..

*Methods.* Among 367 OLT performed between 1985 and 1993, 4 adults (21-52 years) and one child (12 years) received 6 (1.6%) liver allografts for BCS. All patients had undergone a portosystemic shunt 4 to 120 months prior to OLT. Two patients furthermore experienced reoperative abdominal surgery. One patient had a portal thrombosis. In three patients with thrombosed portosystemic shunt, severe portal hypertension and hepatic insufficiency were the reasons for OLT. The portosystemic shunt was patent in the remaining 2 patients and indications for OLT were incapacating chronic encephalopathy in one case and severe hypoxemia secondary to intrapulmonary shunting in the second one. The causal factors of BCS consisted of latent (n=2) or overt (n=1) myeloproliferative disorders, oral contraceptives (n=1) and unknown etiology (n=1). After OLT, all patients were given heparin intravenously and converted to anticoagulants indefinetely.

*Results.* Mean duration of OLT for BCS was 768 ± 345 mn versus 544 ± 186 (other indications, n=362), emphasizing the technical difficulties in this indication. The two patent PSS were divided during OLT. Pathological examination of the explanted liver revealed extensive (predominantly centrolobular) fibrosis (n=4) and cirrhosis (n=1). Post-operative complications were : reoperative surgery for intraperitoneal haemorrhage (n=2), left hepatectomy for partial ischemic necrosis of the liver (n=1), duodenal leak secondary to portosystemic shunt closure (n=1). Two patients died : one adult from portal vein thrombosis after venous graft bypass, one child from acute respiratory distress syndrome. Three patients are currently alive 26, 27, and 54 months, one of whom after regrafting for recurrent BCS secondary to polycythemia rubra vera.

*Conclusions.* 1) Failure of PSS represents a rare and reasonable indication for OLT, despite the risks of SBC recurrence. 2) In this indication, mortality and morbidity of OLT are increased by previous portosystemic shunt.

### F196

BILIARY ANASTOMOSIS WITHOUT BILIARY DRAINAGE IN LIVER TRANSPLANTATION: EXPERIENCE WITH 118 PATIENTS

J. Balsells, J.L. Lázaro, J.E. Murio, R. Charco, I. Bilbao, E. Gifre, C. Ruíz, C. Margarit.
Liver Transplant Unit. Hospital Vall d'Hebron. Barcelona. Spain

The incidence of biliary complications in liver transplant (LTX) ranges from 0% to 35% and mostly are T-tube-related. We present our experience of biliary anastomosis without drainage in 118 LTX.
From June 1991 to November 1994, we performed 128 LTX. Biliary drainage was used in only 10 cases: 4 discrepancies in bile duct size, 3 reLTX, 1 partial LTX and 2 split LTX. In the remaining 118 LTX, 107 end-to-end choledocho-choledochostomies (CC) without T-tube and 11 choledocho-jejunostomies (CJ) without stent were performed. Postoperative mortality (within 30 days) was 10.2%. A further 23 patients died during follow-up.
Biliary complications appeared in 9 patients (7.6%); all complications occurred with CC and none with CJ. Two patients had intrahepatic bilomas, secondary to arterial thrombosis, that required percutaneous biliary drainage and posterior reLTX. Biliary complications directly related to the bile duct occurred in 7 patients: 4 extrinsic compression (2 mucoceles of cystic duct) and 3 anastomotic stenosis. All seven patients underwent surgery: 3 T-tubes were placed in the bile duct and 4 Roux-en-Y CJ were performed. Postoperative mortality was 0%.
Conclusion: Biliary anastomosis without drainage is a safe technique for biliary reconstruction in LTX with a low incidence of complications (7.6%).

### F197

### MEGX TEST IN THE LIVER DONOR
### The Influence of the Preservation Solution

Lamesch P., Ringe B.[1], Oellerich M.[2] Kohlhaw K., Kobes S.

Klinik für Abdominal-,Transplantations- und Gefäßchirurgie, Universität Leipzig;

[1]Abt.für Transplantationschirurgie, [2] Abt.für Klinische Chemie, Universität Göttingen

**INTRODUCTION**
Controverse discussions during the last few years on the prognostic value of the MEGX test as parameter for evaluation of the donor liver has led to question of its final value. The initial results concluding the prognostic efficiency with regard to the 120 day graft survival were drawn from livers preserved in Eurocollins solution. Thereafter UW solution was introduced. The aim of this study was to evaluate the prognostic value of the MEGX test with regard to the preservation solution used.

**MATERIAL AND METHODS**
A total of 204 liver donors were reevaluated. 43 livers were preserved in the Eurocollins solution (Group 1), 161 in the UW solution (Group 2). MEGX test was performed in the donor as described earlier (1 mg/kgBW iv, blood samples drawn at 0 and 15 minutes after injection, fluorescencepolarizationimmuno assay). Statistical analysis was performed by using the U-test for non parametrical evaluation of differences, life table was based on Kaplan-Meier survival analysis (BMDP statistical package).

**RESULTS**
The median values for MEGX formation rate in the Eurocollins and the UW group were similar (99 µg/l vs 106 µg/l), the preservation periods were significantly different (6h22min vs. 12h50 min). There were 9 primary non functioning grafts (PNF), 2 (4.7%) in group 1, 7 (4.9%) in group 2 (ns). In 7 cases of PNF the MEGX formation rate was <90 µg/l, in 2 cases >90 µg/l. Analysis of the 120 day graft survival showed statistical significant differences in the Eurocollins group (p=0.0001), in the UW group these differences were not significant (p=0.7912)(Log rank). The differences remained significant in the whole group.

**DISCUSSION**
The controverse discussion on the value of the MEGX test seems to be partly related to the different preservation solutions used during the various studies performed. This indirectly underlines the preservation quality of the UW solution. The value of the test needs to be put into this context. However, in cases with very low MEGX formation rates (< 50 µg/l) in the donor, OLTX should be performed as an emergency procedure with minimal preservation time if the organ is judged suitable for OLTX.

### F198

### LIVING RELATED LIVER TRANSPLANTATION

M. Malagó, X. Rogiers, L. Fischer, J. Schulte am Esch, M. Gundlach, A Sturm*, M. Burdelski* and C:E Broelsch
Departments of Surgery and Pediatrics*
University Hospital Eppendorf, Hamburg, Germany

The shortage of cadaveric organ donors represents a major obstacle to expansion of liver transplantation programs and expecially in pediatric patients. Living Related Transplantation (LRLT) reprsents one of the techniques available to alleviate the mortality on the list resulting from scarcity of suitable grafts.

From October 1991 to October 1994 40 LRLT were performed at the University of Hmburg in 40 pediatric recipients.

The donors were selected from a pool of 76 relatives. Six of fourty transplantations were performed in emergency conditions.
The waiting time for the elective recipients was 5.3 months.

Thirty eight left lateral segment and 2 full left transplantations were performed. The majority of the recipients (63.4%) were below 10 kg. of weight. Immunosuppression was based on low dose Cyclosporine and Steroids.

There was no primary non function; retransplantation rate was12%. The overall survival is 70 %; in non emergency cases the survival is 77%. The mortality on the list for LRLT was 0% for the last 2 years and the mortality on the cadaveric transplant list was 5.5%.

Conclusions: 1) LRLT is an excellent procedure which yelds good results, expecially in elective settings. 2) It drastically reduces the mortality on the waiting list of a pediatric transplant program and 3) It indirectly improves the results of cadaveric transplantation by shortening waiting times and allowing transplantation earlier in the course of the disease.

### F199

### THERAPEUTIC OPTIONS FOR BENIGN LIVER TUMOURS

G Mangiante, N Nicoli, E Facci, A Acerbi, I Dal Dosso, G Serio
Department of Surgery, University of Verona
Italy

**Therapeutic options for benign liver tumours have changed over the last 20 years. From 1975 to June 1994, we observed 145 liver hemangiomas ( HMG ) ( 57.2 % females-mean age 47.3 years-old, 42.8 %, mean age 50.4 years ). Forty-two symptomatic HMG were resected ( mortality rate was 2.3 % ), while 93 HMG without symptoms were only followed-up: 5 of these increased in size and were resected. Twenty-seven symptomatic cases over 53 focal nodular hyperplasias ( FNH ) were resected, 9 cases were resected and 3 were only biopsed during laparotomy performed for other pathology. Postoperative mortality was nil. Fourteen cases were followed-up after diagnosis performed by imaging techniques and fine needle biopsy. Over a mean period of 23 months no variations of size have been recorded. Increases in GGT and ALP were present respectively in 34% and 22 % of FNH-cases. These values are significantly higher on FNH vs HMG ( p<0.005 ), on FNH vs hepatocellular adenomas ( HCA ) ( p 0.007 ), on FNH vs HMG+HCA ( p< 0.0001 ). Scintigraphic techniques were the most diagnostic accurate tool ( 96.2 % ). All 16 HCA in our series were removed ( 11 females, 5 males ): postoperative mortality was nil. Oestrogen administration was present in 36.4 % of female cases, histological diagnosis vs well differentiated hepatocellular carcinoma ( wd HCC ) was difficult in 2 cases on surgical specimens, whilst 3 cases had spontaneous rupture. Risk of rupture is significantly higher on HCA vs HMG ( p=0.003 ), on HCA vs FNH ( p=0.012 ), and on HCA vs HMG+FNH ( p<0.001 ).**
**Asymptomatic HMG and FNH for their low tendency to increase can be only followed-up by ultrasonography scans, and AFP and CEA values: HCA must be fully resected for risk of spontaneous bleeding, and misdiagnosis vs wd HCC.**

### F200

### ASYMPTOMATIC LESIONS OF THE LIVER - RESULTS OF SURGERY AND OBSERVATION IN BENIGN LIVER TUMORS

A.Weimann, J. Klempnauer, G. Tusch, B. Ringe, R. Pichlmayr
Klinik für Abdominal- und Transplantationschirurgie,
Medizinische Hochschule Hannover/Germany

**Introduction:** Most frequent benign liver tumors are hemangioma, focal nodular hyperplasia (FNH) and hepatocellular adenoma (HCA). In many patients tumor diagnosis is an incidental finding during sonography. While spontaneous rupture for hemangioma and FNH is uncommon and malignant degeneration has been only discussed in single case reports for FNH, these complications are well known for HCA. Therefore, in hemangioma and FNH surgery will be recommanded just in case of symptoms and/or tumor enlargement but should be always performed in HCA. For differentiation of the tumors noninvasively diagnostic imaging procedures can be combined to detect hemangioma and FNH with high accouracy.
**Patients and methods:** Over a period of 13 years (1980-1993) in 236 patients with asymptomatic lesions of the liver diagnosis of a benign tumor could be established. 79 pâtients with a median tumor diameter 8.5 (2-20)cm were operated while 157 patients (hemangioma 104, FNH 53) with a median diameter of 7 (1-22) cm were observed for symptoms and tumor enlargement.
**Results:** In 79 operated patients (hemangioma n=29, FNH n=33, HCA n=17) indication for surgery was unknown dignity of the tumor in 77.2% (n=61), enlargement of tumor size in 15.2% (n=12) and symptoms in 7.6% (n=6). Whenever feasible surgical procedure was tumor enucleation in hemangioma and FNH and resection in HCA. Surgical mortality was 0 and morbidity 20.3% (n=16). During follow up for a median period of 32 (7-132) months asymptomatic patients developed severe symptoms in 5.8% of hemangioma (n=6) and 3.8% of FNH (n=2). Significant increase in tumor size occured in 10.6% (n=11) of hemangioma and 9.4% (n=5) of FNH while decrease was observed in 6.7% (n=7) and 3.8% (n=2) respectively. Neither malignant degeneration, nor tumor rupture could be found.
**Conclusions:** Whenever noninvasive diagnostic imaging fails to exclude hemangioma and FNH in asymptomatic tumor lesions of the liver, it is an indication for surgery because HCA or even HCC cannot be ruled out. For proven hemangioma and FNH observation will be justified. An increase in tumor size has just to be expected in about 10%. In case of tumor enlargement or tumor related symptoms surgery can be performed with low and calculable risk.

### F201

**A RARE CASE OF PSEUDOINFLAMMATORY LIVER TUMOUR: CLINICOPATHOLOGIC FEATURES AND TREATMENT**

**A. Schmid[1], F. Fändrich[1], D. Jänig[2], A. Boluszlavzki[3], D. Henne-Bruns[1]**
**[1]Dept. of General & Thoracic Surgery, [2]Inst. of Pathology and [3]Nuclear Medicine, University of Kiel, Kiel, Germany**

Pseudoinflammatory liver tumours represent an unique event to both, the clinician and the pathologist, as only 30 cases have been reported, so far. These lesions are commonly mistaken for primary hepatocellular carcinomas and usually treated by hepatic resection. The clinicopathologic features are described, as are the implications of accurate diagnosis.

In November 1993, a 32-year-old non-icteric woman was allocated to our University Hospital with the accidentially discovered findings of high elevated blood sedimentation rate (75/115), sideropenia, and gammopathy during pre-operative evaluation for an elective sterilisation. Abdominal ultrasonography, computed tomography (CT) and magnetic resonance tomography (MRT) scans revealed a well circumscribed mass in the anterior segment of the right lobe of the liver. Further evaluation of the tumour dignity included hepato-biliary frequency scintigraphy, albumine, -and colloid scintigraphy which demonstrated a malperfused lesion without radioactive $^{99m}$Tc-colloidal up-take or biliary excretion. Subsequently, further screening tests including gastro-and colonoscopy, ultra-scan of the goiter and mammography were performed to exclude a liver metastasis of different origin. An exploratory laparoscopy disclosed a mass of the right anterior liver with extension to the left-sided third liver segment. Biopsy samples were in consistency with the histologic features of an inflammatory pseudotumour of the liver. These lesions are often observed in connection with traumatic contusions of the liver. Anamnestic inquiry confirmed a car accident 5 years ahead which can be interpreted as the crucial underlying etiologic factor. Under these circumstances it was decided to avoid surgical resection of the liver mass. Instead, regular clinical and radiological examinations to control its growth behaviour ensued, and an uneventful clinical course until now (>1 yr.) can be notified.

We conclude that surgical resections of pseudoinflammatory liver tumours can be avoided if hepatic metabolism and biliary tract function are not impaired by the tumour and if the morphologic evaluation is unequivocal

### F202

SURGERY FOR ADULT POLYCYSTIC LIVER DISEASE

C. Soravia, G. Mentha, Ph. Morel, E. Giostra, A. Rohner
Clinique de Chirurgie digestive, University Hospital,
Geneva, Switzerland

Occasionnally, patients with adult polycystic liver disease (APLD) become symptomatic. For them, surgery may represent the only adequate treatment to relief symptoms. From september 1977 to August 1994, ten female patients were investigated and operated on. Mean age was 49 years (range : 33 - 70 years). All patients were highly symptomatic, complaining of abdominal pain, early satiety, dyspnoea and fatigue. Mean interval time from diagnosis to disabling APLD was 6.2 years (range : 1 - 27 years). Other cystic organ involvment included the kidney in 9 cases and the pancreas in one. There was no hepatic dysfunction, but two patients suffered from renal insufficiency. A family history for APLD was found in 6 patients. For all patients, the surgical procedure consisted in a combination of hepatic resection and cysts fenestration; associated cholecystectomy was performed in 4 instances. One patient died in the immediate postoperative course after developping an acute Budd-Chiari syndrome. Early postoperative complications were : pneumonia (1 case), pancreatitis and deep venous thrombosis (1). Ascites was noted in all patients and treated successfully by diuretics and needle aspiration. Late complications included two cases of incisional hernia, one of acute cholecystitis and one of small bowel obstruction. Mean follow-up time was 6 years (range : 6 months - 17 years). The remaining patients are still alive and symptoms free, but two patients are complaining of residual abdominal disconfort, requiring percutaneous cysts aspiration in one case.

Conclusions : a combined approach of hepatic resection and cyst fenestration has proven feasible for highly symptomatic APLD patients. Extensive fenestration of posterior cysts should be avoided; transverse hepatic resection (frontal hepatectomy) up to the costal margin is proposed. This therapy provides good results at long term follow-up.

### F203

REMARKS AND RESULTS FROM THE MANAGEMENT OF PATIENTS WITH LIVER HAEMANGIOMAS

Z.Plaitakis, C.Lambidis, E.Singelaki, H.Bairaktaris, G.Balanis, I.Tassopoulos
2nd Surgical Department, General State Hospital of Nikea's "St.Panteleimon", Greece

Liver haemangiomas represent the most common benign tumors of the liver, whose incidence varies from 0.4 to 7.3%. We herein present the surgical management and the results of 22 patients with the disease. Over the past 10 years, cavernous liver haemangioma has been diagnosed in 10 men and 12 women, with a mean age of 56 years (30-76). The haemangiomas were located in the right lobe in 17 cases, in the left lobe in 3 cases and the disease was bilobar in 2 cases. In 15 patients, the diagnosis was suggested by an imaging procedure (Group A) whereas in 7 patients the diagnosis was established in the operating room (Group B). From the Group A, 4 patients were managed conservatively and 11 were treated surgically. The operation comprised 7 atypical resections of the right liver lobe or sublobar resection while 4 patients were operated for coexistent diseases. From the Group B, 2 patients were emergently operated because of haemangiomas rupture and intraabdominal haemorrhage which resulted in hypovolemic shock and atypical resection of the right lobe in one, or ligation of the left hepatic artery in the other was performed. In 5 patients the diagnosis of haemangioma was incidentally made while they were operated for coexistent disease and one enucleation of a right lobe haemangioma was performed. The average size of the resected lesions was 7 cm (5 to 10). The pathologic examination showed that the lesions were cavernous haemangiomas. The course after the elective operations was uneventful but one patient with a ruptured haemangioma died during the immediate postoperative period due to myocardial infarction. The mean hospitalization time was 15 days. In conclusion, the majority of the liver haemangiomas are incidentally found at laparotomy or during an imaging procedure. Although the natural history in most cases probably is benign, the indication for resection should be performed selectively.

### F204

SURGICAL TACTICS FOR LIVER HEMANGIOMA

A.Chevokin, S.Karagulyan, N.Kuzovlev, E.Galperin
I.M. Sechenov Moscow Medical Academy, Russia

During 1972-1994 166 consecutive cases of liver hemangioma were observed.

Main complaints: pain, chills and fever had 62 pts. In 15 cases the tumor was palpable. Clinical manifestations were noted at tumor size more then 10 cm and its multiple location. 54 operations were made: hemihepatectomies -16,lobectomies -4, 1-3 segmentectomies and atypical resections -22.

Significant alterations in tumor site (extensive necrosis, stroma sclerosis, and hematoma) were observed in 13 pts and alterations of surrounding liver tissue (from nonspecific hepatitis to necrosis) - in 9, generally in cases of large and giant hemangioma. 3 postoperative deaths occured: 1 of sepsis and 2 of liver failure.

We developed a method of transhepatic finger exposure of lobar and segmentary pedicals. This method together with ultrasonic dissection reduced surgical blood loss to 200-400 ml and shortened the duration of the liver ischemia to 1.5 - 2.0 min. In recent years on 18 hepatectomies were performed without fatal outcome. The number of explorative laparotomies was reduced on acount of sonograpy, computer tomography and angiography.

5 outpatients with hemangioma in both lobes are ander observatin from 3 months to 6 years. They leed an active life style.

Patients with hemangioma sizes less than 5 cm and a negative alpha-fetoprotein reaction go through sonografy every 6 months. For such patients we use sclerotherapy (6) and selective embolisation of hepatic artery branches (5). No more growth of tumor was noticed for 3 months to 3 years in follow-up.

In our opinion, the main indications for surgery for hemangioma are clinical signs as: pain, chills and fever. Unsatisfactory results of hepatectomies depend on surgical blood loss and development of liver failure in the early postoperative period. Using transhepatic selective exposure of hepatic pedicle we were able to reduce the number of early postoperative complications and by means of sclerotherapy and selective embolisation we stopped further tumor growth.

### F205

VIDEO ASSISTED THORACIC SURGERY (VATS) FOR RESECTION OF HETEROTOPIC LIVER, SIMULATING A PULMONARY MASS.

Armando Hernández MD

Policlínica Metropolitana,

Caracas, Venezuela

South America

This is a 69 years old asymptomatic patient who was studied by medical doctor for essential hypertension.
A chest X-Ray was performed, showing a 4 cms. mass, included in lung parenchyma and localized in the medial segment of right lower lobe.
A thoracoscopy was performed, localizing the lession in the apex of the right diaphragm and was resected using a linear cutter ELC35.
The histopathologic studies showed normal hepatic tissue.
The postoperative period was excellent and the patient was discharged 48 hours later.
This is a very uncommon pathology of which I could not find a similar case reported in the world, and for sure the first one resected by VATS.

### F206

RESECTION OF BENIGN LIVER TUMORS: SELECTIVE APPLICATION OF TOTAL VASCULAR ISOLATION M Schwartz MD, S Emre MD, D Kelly MD, P Sheiner MD, S Guy MD, C Miller, MD The Mount Sinai School of Medicine, NY, NY USA

While rarely fatal, large benign liver tumors are a source of significant morbidity, and resection is often indicated. Resection of such lesions can, however, be technically difficult, especially for tumors located centrally or close to major vessels. **METHODS:** Patients undergoing resection of large benign tumors between 9/88 and 9/94 were studied. The preferred approach was enucleation with intermittent inflow occlusion, sparing normal hepatic parenchyma, with anatomic resection applied in only a minority of cases TVI was employed for tumors located adjacent to the cava and/or hepatic veins as well as central tumors; TVI was achieved by clamping the suprahepatic and infrahepatic IVC and the porta hepatis. **RESULTS:** Forty-one patients underwent resection of large benign tumors. Enucleation was performed in 25 cases(61%); anatomic resections performed included L lat segmentectomy (7), L lobectomy (4), R lobectomy (1), and R trisegmentectomy (4). TVI was used in 21 cases (51%); average ischemia time in TVI cases was 18.3 min, (range 7-36 min). Mean tumor diameter was 14.2cm in the TVI group; in non-TVI patients, mean diameter was 10.0cm. Pathologic diagnoses were: hemangioma (11), adenoma (9), focal nodular hyperplasia (8), simple cyst (4), biliary cystadenoma (3), angiomyolipoma (3), schwannoma (1), echinococcus (1), infantile hemangioendothelioma (1). Mean transfusion requirement was .9 U PRBC with TVI, and 3.0 U in non-TVI cases; 24/41 patients (59%) required no transfusion (TVI: 11/21, no TVI: 13/20). All patients survived; liver failure was not seen, and tumor recurrence has not been observed. **CONCLUSIONS:** 1. The relatively bloodless field achieved by TVI facilitates precise dissection of intrahepatic vessels and ducts. 2. Selective application of TVI enables safe resection of large benign tumors with maximal preservation of functioning liver parenchyma.

### F207

SURGICAL TREATMENT OF HEPATIC HEMANGIOMAS.

E.J.Hadjiyannakis, S.Drakopoulos, R.Lakoumenta, N.Georgopoulos, S.Mathioulakis, A.Poultsidi, N.Nikitakis, H.Harisis, G.Mousoulis.

1st Surgical Department and Transplant Unit - "Evagelismos" Hospital of Athens.

From November 1975 to November 1994 we treated 32 hepatic hemangiomas; 9 were operated uponelectively.
The test less than 9 cm did not meet the criteria for Surgical rexection.
The patients 5 males and 4 females had a mean age of 45-50 years old.
4 were located to the left lobe, 4 to the right lobe and one to segments VII, VIII we performed. 4 right extended hepatectomy, one left extended hepatectomy, a left lobectomy and three segmental tesections.

### F208

**ANIMAL EXPERIMENTAL STUDY OF PORTAL HYPERVOLEMIA**

**YT. Huang, WM. Wang, TR. Hang**
**Department of Surgery, First Hospital, Beijing Medical University, China**

**The pathogenic mechanism is a very complicated problem in which some issues are still not well understood. In order to investigate the effect of portal high flow state in the pathogenesis of portal hypertension, an animal model of portal hypervolemia was designed. The procedure was a retrograde portacaval shunt. A regular side-to-side portacaval shunt with large stoma was completed first, followed by ligation of inferior vena cava just proximal to the anastomosis, thus the whole amount of blood from the lower half body turned its flow into the portal system. portal hypervolemic model was made in 11 days. The portal hyperdynamics was studied by using color ultrasonogrphy before operation and 10 days, 20 days and 4 months postoperatively. In the 10th day, the 20th day and 4th month after operation, laparotomy was done again for observing portal pressure, portal colleteral circulation and liver histology. The portal high flow state was maintained constantly and portal pressure elevated in different degrees. The portal collateral circulation and pathological change of the liver was not remarkable. The function of liver was not damaged. The results indicated that simple portal hypervolemia was proved not to cause real clinical portal hypertension, while the elevation of portal resistance was the important initiating for formation of portal hypertension. However portal hypervolemia which frequently associated with the portal hypertension might be the essential factor for main-tance of portal hypertension.**

### F209

THE PATHOPHYSIOLOGY OF HEPATIC ISCHAEMIA - CYTOKINE PROFILES DURING THE REPERFUSION PERIOD

GR Hewitt MI Halliday GR Campbell BJ Rowlands T Diamond
Department of Surgery, The Queen's University, Belfast, N. Ireland

The Kupffer cells of the liver represent the largest fixed mass of macrophages and therefore the potential for release of cytokines when stimulated by ischaemia is considerable. This study was designed to examine the systemic concentrations of IL6 and TNF following 90 and 120min hepatic ischaemia in a rat model.
Eight groups of male Sprague-Dawley rats (n=5 all groups).were subjected to left hemi-hepatic ischaemia. Four groups underwent 90min ischaemia and four groups 120min. Blood was sampled at 0min, 1hr, 3hr, and 5hr following clamp release and assayed for IL6 (bioassay) and TNF (ELISA). Sham animals underwent laparotomy and mobilisation of left hepatic vessels.

Results: Time following clamp release

| | 0 | 1 | 3 | 5 |
|---|---|---|---|---|
| IL6 90 | 226 (37) | 922 (416) | 1727 (487) | 2477 (1602) |
| 120 | 566 (161)* | 1880 (712)* | 7981 (5795)* | 9055 (7874)* |
| TNF 90 | 0 (19.6) | 0 (42) | 0 (49) | 158 (73) |
| 120 | 0 (0) | 232 (198)* | 324 (111)* | 383 (212)** |

Results expressed in pg/ml as median (inter-quartile range). No TNF or IL6 was detected in sham animals.
* = p<0.01 and ** = p<0.05 120 v. 90min ischaemia.
Conclusions: Prolonged hepatic ischaemia was associated with increased systemic concentrations of IL6 and TNF. These concentrations were significantly higher following 120min ischaemia compared to 90min.

### F210

RELATION OF EXPRESSION OF p53, TGF α, EGFr AND Ki 67 IN COLORECTAL LIVER METASTASES TO PROGNOSIS.

K.P. de Jong[1], R. Stellema[1], A. Karrenbeld[2], J. Koudstaal[2], W. Sluiter[3], M.J.H. Slooff[1], E.G.E. de Vries[4]. Departments of Surgery (Division HB Surgery and liver transplantation[1]), Pathology[2], Endocrinology[3] and Medical Oncology[4]. University Hospital Groningen. The Netherlands.

Growth factors and tumor suppressor genes determine prognosis in colorectal cancer. In this study in humans with colorectal liver metastases we investigated the influence of the growth factor TGF α and its receptor (EGFr), the proliferation marker Ki 67 and the protein of tumor suppressor gene p53 on survival. Forty-four patients underwent a laparotomy with the intention to resect one or more colorectal liver metastases. Preoperatively patients were screened for extrahepatic metastases. In 9 patients intraoperative findings precluded performing a curative liver resection. In the remaining 35 patients liver resections were performed: extended hemihepatectomy (n=6), hemihepatectomy (n=20), left lateral segment resection (n=4) or subsegmentectomy (n=5). There was no perioperative mortality. All patients were followed using a regular outpatient follow-up scheme. Mean follow-up is 21.2 months (range 3-65). Materials (either biopsies or resected parts of the liver) were analyzed using monoclonal antibodies (MoAb) for TGF α, EGFr, Ki 67 and p53 protein. Histological slides were scored by two independent observers in a blinded fashion. The percentage of TGF α expression in resected tumors was lower than in non-resectable cases (means (95% C.L.): 43.2 (33.6-52.9) and 86.7 (48.7-124.6) resp. p=0.007, ANOVA). In resected liver metastases no influence of TGF α expression on survival was found. Liver tissue uniformly expressed TGF α to a high extent. Surprisingly only 3 of the liver metastases exhibited positive staining with anti EGFr MoAb. p53 nuclear protein expression was correlated with a significant better survival compared to resected liver metastases without p53 expression (p<0.05). The 3-years disease free survival of p53 positive cases is 47% vs. 17% in p53 negative cases. Expression of the proliferation marker Ki 67 appeared not to be of influence on survival.
Conclusion: TGF α expression in resectable colorectal liver metastases was found to be significantly lower compared to intraoperatively judged irresectable liver metastases but did not have a predictive value on prognosis of patients after partial liver resection. A very strong influence of p53 nuclear protein expression was found on disease free survival after liver resection. p53 expression in liver metastases of colorectal origin is associated with a better prognosis.

### F211

ORAL ARGININE SUPPLEMENTATION IN ACUTE LIVER INJURY

[1]D. Adawi, [1]F.B. Kasravi, [2]G. Molin, [1]B. Jeppsson
[1]Department of surgery, [2]Department of food technology, Lund University, Lund, Sweden

We have studied the effect of oral arginine supplementation on the extend of liver injury and the associated bacterial translocation in an acute liver injury model in rats. In the arginine group, 2% arginine has been supplemented daily through a nasogastric tube for 8 days. Acute liver injury induced in the 8th day by intraperitoneal injection of D-galactosamine (1.1 gm/kg body wt.). Samples were collected 24 hours after the liver injury. In the arginine supplemented group, the Alkaline Phosphatase (ALP), Bilirubin (BIL) and Aspartate Aminotransferase (ASAT) reduced significantly compared to the acute liver injury group. The results of bacterial translocation in the arginine supplemented group show reduction in the number of translocated bacteria in the arterial blood, liver and mesenteric lymph nodes with significant difference in the liver and mesenteric lymph nodes compared to the acute liver injury group. The histologic study of the liver shows in the arginine supplemented group scattered areas of hepatocellular necrosis and inflammatory cell infiltration compared to the acute liver injury group which shows more and widespread hepatocellular necrosis and more inflammatory cell infiltration. These results show that arginine oral supplementation improves significantly the state of the liver injury and reduces the number of the translocated bacteria in the acute liver injury.

### F212

LASER DOPPLER PERFUSION IMAGING-A NEW TECHNIQUE FOR MEASURING LIVER BLOOD FLOW

A.M. Seifalian, L. Horgan, D. Moore, M. Cutress, B.R. Davidson
Department of Hepatobiliary Surgery and Liver Transplantation, Royal Free Hospital School of Medicine, London, U.K.

The objective of the present study was: (1) to establish that liver surface blood flow is homogeneously distributed and (2) to assess the effect on perfusion with occlusion of: a) the splenic artery (SA), b) Hepatic artery (HA) or c) Portal vein (PV) blood flow.
The laser Doppler imager (LDI) is a novel application of Doppler for the measurement of microvascular blood flow in superficial tissues. The major difference to the standard laser Doppler is that the laser scans a large tissue area (120 $mm^2$) creating an overall image of perfusion without the necessity for surface contact. We have investigated the use of the LDI in assessing liver blood flow in six large white/landrace male pigs, average weight(±SD) 27.9±9.1 kg.
There was a 15% regional variation in blood flow on the surface of the liver at normal perfusion. Temporary occlusion of the SA produced a significant increase in liver perfusion in 4 animals (12±3% (mean±SD), p<0.001, versus pre-occlusion) and a reduction in 2 animals (6 and 14%, not significant). When the occlusion was relieved the LDI perfusion returned to normal. Temporary occlusion of the HA produced a significant fall in liver perfusion (20±8%, p<0.001, versus pre-occlusion). Temporary occlusion of the PV caused 62±12% fall in signal from the liver (p<0.001) versus pre-occlusion). The regional variation in surface liver blood flow persisted during clamping. In addition, we have studied two patients following liver transplantation using LDI. In both patients after the revascularisation the tissue perfused well. There was a 10% regional variation in blood flow on the surface of the left lobe of the liver. In one of the patients the SA was clamped which resulted in an increase in mean liver blood flow of 9.3%.

### F213

THE AUGMENTATION OF RAT LIVER REGENERATION BY CYCLOSPORINE (CsA) IS INHIBITED BY A $PGE_2$ RECEPTOR ANTAGONIST.-A FLOW CYTOMETRY STUDY.

I. Fouzas[1], M. Daoudaki[2], Th. Thalhammer[3], Th. Konstandinidis[4], V.Papanikolaou[1], A. Dimitriadou[2], J Graf[3], A Antoniadis[1] and A. Trakatellis[2].Depts of Transplantation[1], Biochemistry[2] and Hygiene[4], School of Medicine, Aristotelian University. Thessaloniki, Greece. Dept of General and Experimental Pathology[3], University of Vienna, Austria.

CsA is known to augment the regenerative responce after 2/3 partial hepatectomy in the rat, by a poorly understood mechanism. CsA is involved in Prostaglandin synthesis by increasing $PGE_2$ production by monocytes. On the other hand $PGE_2$ is reported to increase during liver regeneration and to enhance the regenerative responce by a specific receptor-mediated process. In the present study it was attempted to elucidate the relationship between Cyclosporine and $PGE_2$ by studying the effect of SC-19220, a $PGE_2$ receptor antagonist of the $EP_1$ subtype, on the Cyclosporine augmented rat liver regeneration.**Methods:**Forty male Wistar rats (250-300gr), recieved preoperatively CsA 20mg/kgr BW, in olive oil by gavage.(groups A,C and D), n=10, or only olive oil (group B) and were subjected to either sham operation (group A) or 2/3 partial hepatectomy (groups B, C and D). At the end of the operation an Alzet minipump (2ML1) was implanted IP containing the vehicle for SC-19220 in groups A, B, C, and 20 mg SC-19220 in group D Additionaly, 1 ml of vehicle was injected IP (groups A,B,C) with 5 mg of SC-19220 in group D Postoperatively, all the animals recieved Bromodeoxyuridine (BrdU) 2x50 tab SC at 18 and 42 hours. At 48 hours the liver remnant was excised and perfused in an ex vivo system and lysed with Collagenase. Isolated hepatocytes were stained with an anti BrdU-FITC monoclonal antibody and hepatic regeneration was determined by flow cytometry, measuring the fluorescence of positive cells for BrdU incorporation into the nuclei.**Statistics:**The normal distribution fitting was established by the Kolmogorov-Smirnof test and the groups were compared by the student t test for independent samples.**Results:** CsA increased significantly the regeneration in group C as compared to group B ($p<0.05$). The administration of SC-19220 decreased the regeneration very significantly in group D as compared to group C ($p<0.001$) and group B ($p<0.001$).There was no difference between groups A and D. These findings imply that CsA augments the hepatic regeneration by increasing the $PGE_2$ production in the liver, propably by induction of the Kuppfer cells.

### F214

DEVELOPMENT OF AN EXTRACORPOREAL LIVER ASSIST CIRCUIT

G. Kostopanagiotou, N. Arkadopoulos, J. Theodosopoulos, K.Theodorakis, J. Kapiris, J. Mersinias, J. Papadimitriou
Second Dept. of Surgery, Dept. of Anesthesiology, University of Athens, Areteion Hospital, Athens, Greece

The increasing incidence of fulminant -viral and toxic- hepatic failure (FHF) and the severe shortage of cadaveric liver donors in Greece, have stressed the need for the development of liver support systems, which will be used as a temporary metabolic support of FHF patients until the host liver regenerates or a suitable graft is provided. The aim of this experimental protocol is to develop and extracorporeal liver assist circuit with an incorporated pig liver. The graft liver is obtained from pigs weghing 15-20 Kg. The animals underwent total hepatectomy (under general anesthesia), following cannulation of the portal vein, the infrarenal aorta and the infrahepatic vena cava and perfusion with 2 lit R/L solution with added heparin (10 IU/ml) at 4°C. The circuit consisted of the graft liver connected to a membrane oxygenator, a heater, a centrifuge pump and a fluid reservoir. Inflow to the graft liver goes through the portal vein catheter and outflow comes through the suprahepatic vena cava catheter. Cystic duct ligated and common duct cannulated. The circuit was tested in 15 cases as an isolated liver perfusion system, in order to make the necessary modifications. Bridges were adapted to the circuit to bypass the graft liver when necessary. Mean weight of the graft livers was 780 (610-870) gms and mean total priming volume was 600 ml (R/L and 2% bovine albumin). Oxygenation surface was 1.2 $m^2$ and oxygen flow was set to 3 lit/min. Temperature was maintained at 38°C and inflow pressure (portal vein) was 16 (12-20) mmHg. The flow was increased to 0.5-0.7 ml per gr of graft liver mass per minute. The liver was perfused for a mean of 5.2 (4.5-9) hours. In conclusion the described circuit parameters resulted in satisfactory flow and stable low portal pressure while it enabled the isolated liver to maintain the aforesaid optimal perfusion parameters for a mean duration of 5.2 hours.

### F215

ENDOTHELIN RELEASE IS AUGMENTED WITH CAPTOPRIL IN RAT ISCHEMIA/REPERFUSION INJURY OF THE LIVER

A.Özdemir AKTAN, Bahadır M.GÜLLÜOĞLU, Cumhur YEĞEN, Rıfat YALIN

Marmara University School of Medicine

Department of General Surgery, Istanbul, Turkey

The oxygen-derived free radicals (OFR) and the vascular endothelial factors such as endothelins (ET)s and tromboxane $A_2$ ($TxA_2$) were found to be the mediators of the reperfusion component of ischemia-reperfusion (I/R) injury. Captopril (CPT) which is a sulphydryl (-SH) group containing angiotensin converting enzyme inhibitor has been shown to reverse the I/R injury by its OFR scavenging effect. In this study the effect of CPT and BQ 123, a selective endothelin A receptor blocker, were assesed on liver I/R injury in rats.

The study consisted of four groups of sham-operated, control, CPT,and BQ 123 treated Wistar-Albino rats. The left and lateral hepatic arteries and portal veins were occluded in each group but the sham and the corresponding agents were given to the animals prior to I/R injury. After I/R injury, blood was drawn from the suprahepatic vena cava inferior for endothelin-1 (ET-1) assay and liver tissue samples were obtained for the determination of prostaglandin $E_2$ ($PGE_2$), leukotriene $C_4$ ($LTC_4$) and histopathologic examination.

ET-1 and $PGE_2$ levels were increased significantly in the control group when compared with the sham operated group. In the CPT group, ET-1 and $PGE_2$ levels were significantly increased when compared with the control group while these values were not different than those of the control group in the BQ 123 treated group.

It is concluded that ET-1 release increases in response to I/R injury in rat liver and CPT further increases this release. It also appears that ET-1 release is mediated by $PGE_2$

### F216

EFFECT OF G-CSF ADMINISTRATION ON HEPATOCYTES REGENERATION AFTER PARTIAL HEPATECTOMY IN RATS

S.Theocharis, A.Margeli, S.Skaltsas, M.Horti, N.Goutas, C.Kittas
Department of Histology and Embryology, School of Medicine, University of Athens, Athens, GR 11527, Greece

Granulocyte-colony stimulating factor (G-CSF) is a growth factor capable to stimulate directly and selectively proliferation, differentiation and function of neutrophils and to prime leukocytes for inflammatory stimuli in-vitro. The objective of this study was to examine the effect of G-CSF administration on hepatocytes' regenerative capacity after partial hepatectomy (PH) in rats. Recombinant human G-CSF (Granulokine, Roche, Switzerland) was administered intraperitoneally at doses of 1500, 150, 15 and 0 μg/Kg body weight simultaneously to PH and the hepatocytes' regenerative capacity was examined 24 hours postoperatively. Tritium thymidine incorporation into hepatic DNA, liver thymidine kinase (TK) activity, mitotic index and proliferating cell nuclear antigen (PCNA) immunostaining, were determined as indices of liver regeneration. The simultaneous to PH administration of G-CSF at doses of 1500, 150 and 15 μg/Kg body weight, caused increase of hepatocytes' DNA synthesis, at percentage of 74, 60 and 3% respectively, compared to that observed in simply partially hepatectomized and saline-treated rats (0 μg/Kg). TK activity and mitotic index presented similar alterations. These observations provide evidence for a beneficial effect of G-CSF administration on hepatocytes' regenerative capacity

### F217

### HILAR CHOLANGIOCARCINOMA: RESULTS OF RADICAL RESECTION

M. Gundlach, K.L. Prenzel, W.T. Knoefel, X. Rogiers, J.R. Izbicki, C.E. Brölsch
Dept. of Surgery, University of Hamburg, Germany

**Introduction:** Since the development of surgical techniques has allowed the curative resection of hilar cholangiocarcinoma with reasonably low postoperative mortality, the question that arises is whether radical surgery influences patient survival. In a retrospective study the impact of hilar resection with or without liver resection was evaluated in 63 consecutive patients treated at our institution. **Patients and Results:** According to the Bismuth classification, in 77 % a stage III or IV tumor was found. In 9 patients no surgical procedure was performed because of metastases or age. A total of 54 patients underwent laparotomy. No resection was possible in 21 of these cases (39%) because of irresectability or previously undetected metastases. 19 patients underwent bile duct resection alone and 14 in combination with liver resection. 53% of these resections were curative (R-0). The median survival times were: R-0, 28 months; R-1, 22 months, and R-2, 6 months. There was a significant difference between overall R-0 and R-1/2 resections (0.0001 Log rank). After treatment with a stent alone, the median survival was 9 months after laparotomy and stent 6 months, after bile duct resection alone 17 months and for combined liver and bile duct 18 months (0.0001 Log rank). **Conclusion:** Our results suggest that R-0 resection is imperative to achieve improved survival in hilar cholangiocarcinoma. Bile duct resection alone will usually allow this only in Bismuth stage I or II tumors. In contrast stage II and IV tumors usually require bile duct and liver resection if the tumors are at all resectable.

### F218

### SURGICAL TREATMENT FOR RUPTURED HEPATOCELLULAR CARCINOMA

Miin-Fu Chen M.D., FACS
Department of Surgery, Chang Gung Memorial, Hospital, Chang Gung College of Medicine & Technology, Taipei Taiwan

*Introduction:* Spontaneous ruptured hepatocellular carcinoma (HCC) is an uncommon but fatal complication of this disease. Surgical treatment is aimed at controlling the intraperitoneal hemorrhage; and it could be achieved by hepatectomy, hepatic arterial ligation (HAL), packing and suturing. However, informations on the surgical treatment for spontaneous ruptured HCC were incomplete.

*Materials and Methods:* Clinicopathological features and types of surgical management of 50 patients with ruptured HCC during the past 15 years were reviewed. The morbidity, mortality and survival rates were analyzed.

*Results:* The indications for emergent laparotomy were hemoperitoneum in 26 and unspecified peritonitis in 24. Twenty-two were found to have non-cirrhotic HCC. The incidence of associated cirrhosis was 56.0 percent. Surgical procedures included hepatic resection in 23, hepatic arterial ligation in 17 and packing, suturing, and electro-cauterization in 10. Re-bleeding rate for non-hepatectomized group was high. Fourteen died within one month postoperatively, with a surgical mortality rate of 28 percent. Recently, palliative hepatic resection has been used more frequently. The group of patients who underwent resection have a better prognosis. Cumulative survival rates of 1-, 2-, 3- and 5-year for hepatectomized patients were 60%, 52%, 40.5% and 26.5%, respectively. Long-term survival can be observed in a few patients without recurrence.

*Conclusion:* Hepatic resection is the treatment of choice for uptured HCC. Long-term survival can be observed in a few patients without recurrence.

### F219

### SURGICAL TREATMENT FOR HEPATO CELLULAR CARCINOMA OF DIFFERENT SIZE.

G. Belli, A. D'Agostino, I. Marano*, A. Iannelli, P. Ceccarelli, and M.L. Santangelo.

Department of General Surgery and Organs Trasplantation.

*Department of Radiologic Sciences

University of Naples "Federico II" Medical School, Italy.

Hepatocellular carcinoma (HCC) is worldwide a major cause of cancer mortality. At present an effective therapeutic scheme for this malignancy has not been developed. In recent years TAE, PEI and other non operative procedures have been proposed. Here we attempted to evaluate the indications and the effectiveness for surgical resection in patients with HCC of different sizes.

From December 1984 to December 1994 we observed 76 patients with HCC (69 on cirrhotic liver). Of these patient 27 belonged to the A Child's class, 23 to B class and 19 to C class. Serum level of a fetoprotein (aFP) was increased in 41 patients. Fourty six patients were operated. The size of the lesions (single or multiple nodules) ranged from 0.7 to 13 cm. No statistically significant correlation between Child's class and tumour size was observed. We performed 8 major resection (5 in non cirrhotic patients), 33 minor resections (2 in cirrhotic patients) and in 5 cases only laparotomy for unresectable cancer. Complications occurred in 7 patients (16.6%). The incidence of postoperative death was 9.7% (4 patients). The best results were obtained in patients with single neoplasm < 5 cm or multiple neoplastic nodules < 3 and < 3 cm and adequate liver function reserve. Surgical procedure offers many .advantages such as the abdominal and liver exploration, optimal staging and hystologic examination of the resected specimen to evaluate the predictive factors of recurrence. Thus, surgical resection should still be the best choice of treatment for HCC in selected patients.

### F220

### PALLIATIVE TREATMENT IN KLATSKIN'S TUMOR

A. Principe, M. Lugaresi, A. Mazziotti, F.D'Ovidio, G.Grazi and G. Gozzetti.
2° Department of Surgery - Policlinico S.Orsola - University of Bologna - ITALY

Long-term survival of patients with Klatskin's tumor is now the issue. The late clinical presentation of these lesions is responsible, in the majority of cases (70%), of poor survival due to extensive tumor diffusion. Tumor resecability or palliation parameters are given in a definite way only at laparotomic exploration. Various operative techniques can be performed for palliation of non resectable tumors.
Material and Methods: Since 1981, 59 patients (38M, 21F ; mean age 57 years, range 35-73) with Klatskin's tumor have been observed. Two patients died before surgical intervention for hepato-renal insufficiency and have been excluded from the study. Curative resection of the tumor was possible only in 18 cases (31,6 %). Jaundice was treated in the remaining 39 patients (68,4 %) following three different modalities. Nine patients underwent Intrahepatic Bilio-Digestive-Anastomosis (IBDA). On 17 patients was performed a Surgical Biliary Drainage (SBD), 8 externally and 9 transtumorally. Of the remaining 13 patients in 10 was possible only a Non Surgical Biliary Drainage (NSBD) and 3 were not treated because without jaundice.
Results: There were 4 p-o deaths (mortality 10 %). Mean survival according to the type of biliary derivation was : 7 months for IBDA ( range 1-18 mo.); 9 months for external SBD (range 1-30 mths)and 11 months for internal SBD (range 2-38 mths), 3,5 months for patients with NSBD. (range 1-9 mths).
Conclusions: Only explorative laparatomy permits a valid tumor resecability assessment. Moreover the results of our study seem to show that in non resectable tumors, surgical transtumoral biliary intubation is the better method to improve in these patients survival and quality of life.

### F221

### STAGING AND CURATIVE RESECTION OF KLATSKIN'S TUMORS

A. Principe, M.Lugaresi, A. Mazziotti, F. D'Ovidio, E. Jovine and G. Gozzetti
2° Department of Surgery - Policlinico S.Orsola - University of Bologna - ITALY

Despite recent advances in diagnostic techniques Klatskin's tumor is still frequently observed at an advanced stage. However extensive resection of hilar extra hepatic cholangiocarcinoma combined with liver resection is feasible and, in selected cases, it is able to modify the natural history of the tumor. Adoption of an accurate preoperative diagnostic study is fundamental to clearly define the site of the lesion, but only surgical exploration and intraoperative ultrasonography give precise informations on the resecability.

Material and Methods: Since 1981, 59 patients (38M, 21F; mean age 57 years, range 35-73) have been observed. The preoperative work-up, in all cases, was carried out through US, CT, Cholangiography (PTC or ERCP) and in 16 cases with Arteriography. Two patients with PTBD were excluded from this study because of death before intervention for hepato-renal insufficiency. Surgical exploration modified the preoperative staging in 21 cases (36,8 %). Surgical resection was possible in 18 cases (31,6% ). Four patients were submitted to tumor resection, in 1 of them was associated a WR of the fourth segment. Thirteen patients were treated with liver resection: 7 with a simple Hepatectomy, and 6 with an extended Hepatectomy, associated in 4 cases to the resection of the caudate lobe.

Results: The post-operative hospital stay was regular in 10 patients (mean hospitalization 17 days) and complicated in 7 (mean hospitalization 35 days). There was 1 p.o. death (mortality 5.5%) (stage II). Nine patients (1 stage I; 3 stage II; 5 stage IVa) are currently alive (mean follow-up 41 months.; range 6-144 mths). The remaining 8 pts (6 stage IVa; 2 stage IVb) had a mean follow-up of 19 months.(range 2-55 mths).

Conclusions: Although presentation is at an advanced stage, extensive resection of the bile ducts, especially in early stadiation, combined with liver resection, including the caudate lobe, is feasible and curative with a good mean survival and a good quality of life.

### F223

### RECURRENCE OF RESECTED HEPATOCELLULAR CARCINOMA IN CIRRHOTIC PATIENTS. RISK FACTORS AND DNA PLOIDY STUDY

J. Balsells, I. Caragol, E. Allende, J.L. Lázaro, E. Murio, R. Charco, I. Bilbao, E. Gifre, C. Ruiz, C. Margarit.
Liver Transplant Unit, Biochemistry and Pathology Departments. Hospital Vall d'Hebron. Barcelona. Spain.

The aim of the study is to analize which clinical and pathologic features were risk factors for recurrence of resected hepatocellular carcinoma (HCC) in cirrhosis, and the relationship between tumor DNA ploidy and recurrence or risk factors.

In 7 years, 53 hepatic resections for HCC were performed in 51 cases. The tumor was asymptomatic in 66% of patients. Most patients were treated with a limited resection. Recurrence of HCC was diagnosed in 27 patients (59%). Mean time of recurrence was 11.9±10.6 months. The actuarial survival at 1, 3 and 5 years were 68%, 43% and 36% respectively. The study of tumoral DNA ploidy with flow cytometry was performed in 44 samples of HCC included in paraffin. Tumors were diploid in 27 cases and aneuploid in 17.

Risk factors statistically significant for recurrence were symptomatic patients, more than one nodule, and a tumor with satellite nodules. With Kaplan-Meier survival curves, only symptomatic tumors remained statistically significant for recurrence. The recurrence rates for diploid or aneuploid HCC were 69% and 50% respectively, but the difference was not statistically significant. No relationship was found between tumor ploidy and risk factors for recurrence.

Conclusion: Recurrence rate of HCC in cirrothic patients is high with surgical resection. The only significant risk factor for recurrence was symptomatic tumors. The ploidy of the tumor is not predictive for recurrence.

### F222

### ALCOHOLISATION OF HEPATIC TUMORS

I. Kafetzis, E. Mittari, G. Vlachos, KH Mustafa, S.Dimas, I. Damtsios.
Surgical Department of Athens Polyclinic. Greece

The abblation of the tumor or the hepatic transplantation is radical treatment of primary or secondary hepatocellular carcinoma (HC). Sometimes this procedure is not reliable because of the general condition or the age of the patient or for different other reasons. The embolisation that is an alternative palliative method is not always reliable because of anatomical abnormalities, portal thrombosis or renal and hepatic impairment.In this category of patients we perform an alternative therapeutic technique. The percutaneous alcoholisation(PA) of the tumor under ultrasound quidance.During the last 2 years (1989-1990) we performed with use of ultrasound 8 PA for HC in cirrhosis. Six of them were men and 2 women. Mean age: 67.

The technique was as follows: under neuroliptoanalgesia and with use of ultrasound we do an aspiration biopsy if the histology is not known. Afterwords we perform the PA with 4-5 ml pure alcohol (99%) sterile. Depending on the results we continue the PA at least 4 times with an interval of 3 weeks.In 6 cases the tumor was solitary with 40 diameter. In 2 multifocal. Some of the patients underwent an hepatectomy previously or chemoembolisation. The dimension of the tumor was decreased in 2 cases. In one a total tumor necrosis was observed. In 2 cases the tumor remained unchanged for 6 months and in 2 cases continued to progress.

CONCLUSION: In high risk patients with bad general condition (hepatic or renal failure) that we can't perform an alternative treatment, PA seems to be a reasonable option as a palliative method. The necrosis or the regression of the tumor is observed in some cases.

### F224

### INCORPORATION OF IODISED OIL BY TUMOUR AND ENDOTHELIAL CELLS

S Bhattacharya, AP Dhillon*, BR Davidson, MC Winslet, KEF Hobbs
University Departments of Surgery and Histopathology*
Royal Free Hospital and School of Medicine, London

Lipiodol (iodised oil) administered into the hepatic artery is selectively retained by hepatocellular carcinomas (HCCs) for prolonged periods. Despite its use in diagnosis and therapy, the mechanism of Lipiodol retention has remained unclear so far.

The interaction of Lipiodol with tumour and endothelial cells was studied *in vitro* in cell cultures and *in vivo* in human HCCs. Cultures of HepG2 (a human liver tumour cell line) and HUVECs (human umbilical vein endothelial cells, which share phenotypic markers with the endothelium in HCCs) were exposed to 1%, 2% and 4% Lipiodol in culture medium for 4, 8, 24 and 32 hours. Cell monolayers were stained for Lipiodol by a selective silver nitrate impregnation technique. All tumour and endothelial cultures demonstrated incorporation of Lipiodol by the cells. Cellular Lipiodol uptake was quantitated by computer-assisted image analysis of the optical density of silver nitrate stained monolayers. HepG2 cells demonstrated a slow rate of uptake initially, followed by progressive intracellular accumulation. HUVECs demonstrated rapid initial uptake, but subsequently the optical densities diminished, indicating that the Lipiodol had been excreted or metabolised by the cells. Electron microscopy of HepG2 demonstrated membrane-bound lipid vesicles in the cytoplasm, suggestive of uptake by pinocytosis. Lipiodol had no significant effect on cell viability, cell numbers or protein synthesis.

Histologic assessment of Lipiodol retention in HCCs was performed on 8 surgically resected tumours administered pre-operative arterial Lipiodol-Epirubicin and 10 incidental HCCs discovered in cirrhotic livers removed at transplantation, wherein Lipiodol had been administered at prior arteriography. Light and electron microscopy after silver impregnation confirmed Lipiodol incorporation by tumour cells and by endothelial cells lining tumour vessels.

Liver tumour cells and endothelial cells *in vitro* incorporate Lipiodol by pinocytosis, without suffering adverse effects. Similar mechanisms probably operate *in vivo*. The intracellular penetration of Lipiodol has significant implications for its use as a vehicle for therapeutic agents.

### F225

HEMOSTATIC MEASURES IN LIVER RESECTION

Amr Helmy,M.D.
Professor and Chairman of the Surgery Dept. at the Liver Institute, Menoufiya University

Hemorrhage is one of the main factors of death and morbidity in liver resection. This report defines the different methods used in the "Liver Institute" to guard against intra-operative bleeding. Intraoperative ultra-sonography has helped a lot to accurately define the tumour extent and the venous trunks, thus enabling to position the inter-segmental boundary zones. Ultrasonic dis-sector and aspirator is of reasonable help to define liver tissue and vascular struc-tures. Anatomical approach and complete isolation of the vascular structures, infra and supra hepatic, including the portal triad and the IVC after mobilisation of the liver should preceed attempting to resect. Infrared contact coagulator and Argon beam coagulator are used to control the ooze of blood from the raw surface of the liver. Direct application of biological components like a ready-to-use combination of a collagen carrier coated with fibrin sealant (fibrinogen and thrombin) is effective, when the systemic coagulation is reduced.

### F226

EVALUATION OF THE SPLENECTOMY IN CHRONIC IMMUNOLOGIC THROMBOCYTOPENIC PURPURA (ITP)

I.F.S.F Boin, J.M.A. Bizzachi, G. Berenhauser-Leite, L.S. Leonardi
Department of Digestive Diseases Surgery, State University of Campinas, Campinas-SP, Brazil

We retrospectively examined 32 patients with chronic ITP, treated with splenectomy.
Prognostic clinical variables, like age, sex, interval between diagnosis and splenectomy, type and intensity of bleeding, type of therapies used and responses achieved by these therapies were analysed our date indicate that the chance of prolongade remission after splenectomy in patients with chronic ITP cannot be adequately predicted on the basis of pre-splenectomy clinical variables.
Complete response to splenectomy was achieved in 62% of patients and after one year of follow-up the probability of survival without relapse was 68%

### F227

**MANAGEMENT OF TRAUMATIC LIVER INJURIES**

**O. Yagmur, O. Demircan, H. Sonmez, F.C. Ozkan, E.U. Erkocak**
**Department of General Surgery, Cukurova University School of Medicine, Adana-TURKEY**

From 1980 to 1994, 247 patients with liver injuries were treated at Cukurova University, School of Medicine, Dept. of General Surgery, Adana-Turkey. Patients were analyzed according to age, sex, type of trauma, severity of liver injury, additional organ injury, surgical procedure, mortality and morbidity retrospectively.
Of these 247 patients, 192 were males and 55 were females, ranging from 14 to 80 years of age ( mean, 31.8 years ). The causes of the liver injuries were penetrating trauma in 134 (54%), blunt trauma in 113 (46%). Liver injuries were divided into five classes according to the extent of injury (Moore's classification). Grade-I: 77 (31%), Grade-II: 109 (44%), Grade-III: 34 (14%), Grade-IV: 12 (5%), Grade-V: 15 (6%) patients. 103 patients have had additional organ injury also. All patients underwent operation; primary suture and drainage in 193 (78%), extensive hepatorraphy in 16 (6.7%), segmentary resection in 17 (6.8%), vascular ligation in 5 (2%), resectional debridmant in 4 (1.6%), lobectomy in 6 (2.4%) patients.
Mortality for the entire series of 247 patients was 17%, with 55% (24/44) of all deaths occurring in the perioperative and early postoperative period from shock or transfusion related coagulapathies.

### F228

"GAUZE PACKING" FOR CONTROL OF HEMORRHAGE IN SEVERE LIVER TRAUMA

A. Mazis, S. Rathosis, C. Vlahos, C. Koutos, D. Danikas, P. Myrillas

Department of General Surgery, "Agios Andreas" Gen. Hospital, Patras, Greece.

We present our experience in liver hemostasis using packing technique for severe Liver injuries.
During a ten years period (1980 throu 1990) we operated 4 patients those being 3 males and 1 female with a mean age of 42,1 (range 22 to 71) with Liver injuries (3 class IV and 1 class V)
The technique we used in our cases was the "multiple laparotomy pads technique" because it is most convenient and readily available during crucial hemorrhage. Packs were placed between the liver and the diaphragm, as well as, below the liver, providing compression above and below the sites of hemorrhage.
We were successful in controlling hemorrhage in 3 patients. One male 28 y.o. died in the recovery room due to massive bleeding (rupture of suprahepatic vena cava and intracranial hemorrhage) Reoperation and pack removal took place in another hospital 32 to 64 hours after the previous operation.
During reexploration packs were removed without further complications or significant hemorrhage.
Perihepatic packing for control of hemorrhage, is safe, effective and life saving in many instances. Packing obviously is not recommended for the majority of liver trauma cases. In a community Hospital, the surgeon must assess the liver injury quickly and combine the knowledge with the other factors, that can influence the patients recovery, such as, the surgeon's experience in these type of liver trauma, blood bank supply, ICU availability e.t.c.

### F229

### LIVER TRAUMA: A 10 - YEAR EXPERIENCE

Atilla ÇÖKMEZ, Okay NAZLI, Serhat GÜR, Bilek TAŞKIN, Sümer DENİZ

First Department of Surgery, Atatürk State Hospital, İZMİR TURKEY

The menagement of 67 patients with liver trauma (56 male, 11 female; mean age: 32 (range 15-71) years) presenting from January 1984 to September 1994 is reviewed. There were 28 cases of penetrating injury and 39 of blunt trauma. Any patients was not menaged without operation. Forty-three cases were classified as simple injuries (grade 1 or 2) and were menaged by suture (with drainage) or required no intervention, with two deaths. Twenty-four cases were classified as complex injuries (grade 3 or 4) and underwent one or more of the following; resectional debridement, partial hepatectomy, hepatotomy with direct suture ligation. Twelve of this patients died from uncontrolled haemorrhage. The continued use of suture for simple injuries and of resectional debridement and/or hepatotomy with direct suture ligation for complex injuries is supported. Judicious clinical assessement and radiological monitoring may reduce the number of unnecessary laparotomies.

### F230

### SURGICAL THERAPY OF LIVER TRAUMA

M.Stojiljković,M.Jeremić,M.Stojanović, G.Stanojević,Z.Rančić

Surgical clinic.Clinical center-Niš,Serbia, Yugoslavia

The aim of this study is to analyze the resul ts of 67 cases of liver trauma surgically treated in the last five-year period(1989-1993).There were 41 men and 26 women with the average age of 32,7 yrs.Blunt abdominal injuries due to road accidents were predominant.Associated abdominal and/or extraabdominal injuries were noted in 53 cases(7[illegible]%) The hepatic lesions affected the right lobe in 48 cases,left in 13 and both in 6 cases. Following classification of the AAST there were:Grade I-8 cases; Grade II-36 cases; GradeIII-8 cases; Grade IV-3 cases and Grade V-1 case.Surgical treatment consisted on: drainage alone-5 cases,suture and/or haemostasis-46 cases,debridman and haemostasis -6 cases and liver resection-3 cases.The most cases of complications were:intraabdominal infections in 7 cases,bleeding in 4 cases and pleuropulmonal complications in 4 cases.The mortality rate was 11 patients or 16,41% ,with hepatic lesions being responsible in 3 cases(4,1%).Liver trauma is still associated with high morbidity and mortality. Proper surgical treatment is based on efficient haemostasis and debridman of necrotic tissue.Liver resection should be applied for the greatest distraction of liver parenchima. As mortality in liver trauma depends fundamentally on the seriousness of extrahepatic injuries,multidisciplinary approach with adequate treatment may improve the results.

### F231

### *TREATMENT OF LIVER INJURIES AT THE DEPARTMENT OF TRAUMATOLOGY IN LJUBLJANA IN THE PAST TEN YEARS (1985-1994)*

*J. Prinčič, A. Baraga, M. Kastelec*
*Department of Traumatology, University Medical Centre Ljubljana, Slovenia*

*The authors report on the incidence of liver injuries among their patients, the methods used in the diagnosis and treatment of these injuries, and the final treatment outcome.*
*Diagnosis in the first years was based mainly on clinical evaluation and peritoneal lavage. In the recent years ultrasound has become the principal diagnostic technique. Changes have occurred also in the therapy: cholecystectomy and T-Drainage used initially in the management of liver rupture have been completely abandoned, while packing and subsequent definitive repair are gaining in importance. Thanks to reliable ultrasound diagnosis and follow-up evaluation, a greater proportion of cases of minor liver trauma are now treated conservatively.*
*Of a total of 60.000 injured patients admitted to the Department in the past 10 year, 176 (0,3%) presented with injuries to the liver. These patients were aged on average 33,6 years. The majority were males (126, 76,1%) injured in traffic accidents. As many as 129 patients (73,3%) presented with multiple injuries, isolated liver trauma being present in only 47 cases (26,7%). The mortality rate in the group as a whole was 11,4% (20 deaths), but in the multiply injured patients, it was as high as 19%.*
*In conclusion the authors emphasize the importance of a thorough diagnostic evaluation and a selective approach in the treatment of multiply injured patients.*

### F232

### MORTALITY AND MORBIDITY OF LIVER TRAUMA. ANALYSIS OF 588 CASES

G. Kyratzis, L. Papastamatiou, J. Virlos, N. Mourelatos
2nd Dept.of Surgery "Apostle Paul" Hosp.-KAT. ATHENS HELLAS

Liver trauma still remains a strong challenge for the surgeon because survival and complications are depending on both, early diagnosis and proper treatment.

During the last 20 year period a total number of 588 patients (General Hosp. and Apostle Paul Hosp.) with hepatic trauma were treated. Blunt in 87.6% and open abdominal injury in 12.4% were responsible for liver trauma.

The severity of the trauma was classified in four degrees. A: 99 cases B: 313 cases C: 122 cases and D: 54 cases i.e. complex hepatic injuries (9.2%) including the porta hepatis and/or the juxtahepatic outflow circulation. These cases were treated with temporary occlusion of the inflow vessels and/or internal shunt of the inf. vena cava. Another 13 cases were treated conservatively.

Overal mortality rate was 23.6% correlated to coexistent injuries. Mortality of liver trauma per se did not reach 14% with a wide spectrum from A to D degree (1.1 - 47.8%).

Postoperative complications reached 30% especially in degrees C and D, while haemorrhage and perihepatic collections were the causes of re-exploration in 16 cases.

It is concluded that hepatic trauma allthough appears decreasing mortality rates, due to modern surgical treatment, still remains an injury with expected high morbidity.

### F233

HEPATIC TRAUMA - EXPERIENCE OF A 219 CASES (1984-1993)

G.Ionescu,M.Ciurel,A.Bucur,M.Beuran,S.Jianu,G.Manea, S.Păun,T.Gaspar

Clinic of Surgery,Emergency Hospital,Bucharest,Romania.

This study tried to evaluate the actual condition of the hepatic trauma - etiology,diagnosis,associate injuries, types of interventions,morbidity and mortality - with the purpose to decrease the mortality and to rise the quality of life in these patients.Using a retrospective analysis 219 persons with hepatic trauma hospitalized in the last 10 years,were examined.Etiological,the aggressions and the accidents were prevailed-74%,and the most patients (87%) had multiple injuries.Clinical manifestations and peritoneal prick,wash and drainage were very useful for diagnosis in many cases - only in the last year were performed echography,CT and laparoscopy.Statistically,41% were injuries of first and second degrees,39% third degree 16% fourth degree and 4% fifth degree (in according with Moore's scale).Hepatic sutures were performed in majority of patients-87%,hepatic resections in 6% of cases,great vessels sutures-1%,simple exploratory laparotomy-6%.General mortality was of 34%,65% of the patients died in the first 24 hours by shock.
Concluding,the improvement of the condition in hepatic trauma (with an increased frequency and gravity) it is not only good surgical techniques but even intensive care measures including competent network of traumatology.

### F234

RISKS OF RIGHT HEPATECTOMY IN CIRRHOTIC LIVER

B. Malassagne, R. Noun, A. Sauvanet, J. Belghiti
Department of Digestive Surgery, Hôpital Beaujon, F - 92110 CLICHY.

In order to evaluate the postoperative risks of major hepatectomy in cirrhotic patients, we selected a subgroup of 18 good risk cirrhotic patients (Child-Pugh's score A5 to A7) who underwent an homogeneous resection for hepatocellular carcinoma (HCC).
Patients and Methods : This group of 18 cirrhotic patients was compared with a group of 10 patients with HCC arising in normal liver. Mean age was identical in the two groups (55±9 years vs 54±14 years). In both groups, hepatic resections including resection of segments V, VI, VII and VIII were performed with preliminary vascular control. The duration of continuous pedicular clamping was similar in the two groups (34 ± 12 vs 32 ± 10 min). Blood transfusion was similar in the two groups (1 ± 1 vs 0.5 ± 0.5 units). The weight of the resected specimen was significantly (p<0.01) lower in the cirrhotic group (1069±373 vs 1554±573 g).
Results:

| **Postoperat. complications** | | | |
|---|---|---|---|
| Liver insufficiency[1] | 14 | 5 | p<0.05 |
| - factor V (day) | 35±16 | 52±21 | p<0.05 |
| - ascites | 15 | 1 | p<0.001 |
| - portal vein thrombosis | 3 | 0 | NS |
| **Postoperat. death (cause)** | 3 liver failure | 1 peritonitis | NS |

[1] : liver insufficiency was defined as factor V < 40%, bilirubinemia > 100µmoles/L or encephalopathy

Both rate and duration of postoperative liver insufficiency was significantly higher in the cirrhotic group than in non-cirrhotic group, respectively (77 vs 50%) and (5±7 days vs 1±2 days). Portal thrombosis occurred in 3 patients with cirrhosis. In the cirrhotic group, postoperative death occurred respectively on days 11, 12 and 19.
In conclusion, the delayed period of regeneration of the resected cirrhotic liver requires prevention, prompt diagnosis and specific treatment of factors triggering liver failure at least within the first three weeks.

### F235

**LIVER RESECTIONS WITHOUT BLOOD TRANSFUSIONS**

Gozzetti G., Mazziotti A., Grazi G.L., Jovine E., Principe A., Gallucci A., Gruttadauria S, Morganti M., Ercolani G., Pierangeli F.
2° Department of Surgery, Policlinico S.Orsola, University of Bologna, Italy

Liver resections (LRs) are major procedures usually associated with the use of large amount of blood transfusions (BTs). However the tendency to carry out liver resections without transfusions has constantly increased in recent years, thanks to the improvement of surgical tecniques with less blood loss during the operation and due to the effect of a more careful choice of the timing of the transfusion. A retrospective study was performed on 522 "elective" LRs carried out over the past 13 years to ascertain the feasibility of liver surgery without BTs and the impact impact of transfusions on the immediate post-operative outcome and the long term survival. **PATIENTS AND METHODS**: Indication for surgery was hepatocellular carcinoma in 216 patients, of these 150 were cirrhotics, metastases in 153, benign tumors in 76 and others diseases in the remaining 82. Cancer was present in 369. There were 89 wedge LRs, 281 segmentectomies and 152 major hepatectomies. **RESULTS:** Fifty point six wedge, 42.1% segmentectomies and 19.6% major hepatectomies were carried out without BTs, giving a total of 185 (35.4%) LRs carried out without BTs; this number has increased over the past 5 years from 25 (14.3%) to 160 (49.8%) (p<0.0001). Mortality rate was 6.7% in cirrhotics, 1.1% in non cirrhotics, O% in benign tumors Post-operative mortality and complications were correlated with the transfusions, particulary in cirrhotic patients. In the latter, the linear regression analysis showed how the blood transfusions are the only factor which is significantly correlated with the onset of complications. The transfusions also affected the long term survival of patients operated on for hepatocellular carcinoma and for metastases in univariate analysis and were the only factor correlated with survival in HCC on cirrhotics in multivariate analysis. **CONCLUSIONS**: Liver resections have become safe operations with a minimal operative risk; even major resection are feasible without having to resort to intraoperative transfusions. Liver surgery performed without BTs leads to a better post-operative recovery, expecially in cirrhotics. Long term-survival in patients operated on for liver tumors can also be unfavourably affected by the use of intraoperative transfusions. The advantages of surgery without transfusions are evident in terms of cost, prevention of infectious diseases transmitted with the blood, reduction in post-operative morbidity and improvement in the long-term results in patients operated on for tumors. The use of blood transfusions during liver surgery should be avoided, whenever possible.

### F236

PREDICTIVE FACTORS OF OPERATIVE BLEEDING DURING RIGHT HEPATECTOMY.

P. Jagot, L. Dugué,R. Noun, D. Sommacale, A. Sauvanet, J. Belghiti.
Department of Digestive Surgery, Hôpital Beaujon, F - 92110 Clichy.

Blood transfusion is a main short term prognosis factor after hepatic resection. The purpose of this study was to determine predicting factors of operative bleeding in a homogenous series of 80 right hepatectomies (RH).
Patients and Methods : From 1988 to 1994, 80 patients (49 men and 31 women) with a mean age of 51±16 years underwent a RH. Indications of surgical resection included a malignant lesion (n=57) or a benign lesion (n=23). The remnant liver parenchyma was normal (n= 49), steatotic (>30%) (n=9) or with chronic hepatitis (n=22). Preoperative systemic chemotherapy (5FU-folinic acid) was performed in 11 patients. Liver function tests were normal in all cases. All RH were performed under vascular occlusion including portal triad clamping (n=61) or hepatic vascular exclusion (n=19) with a mean occlusion duration of 32±9 min. The middle hepatic vein was resected in 9 cases. Difficulty during liver mobilisation, resected specimen weight and the number of packed red cells units (PRCU) were noted.
Results: Mean blood transfusion was 1.5± 2.5 PRCU. Forty-nine (62%)patients were not transfused. The number of PCRU operatively transfused were correlated by univariate analysis according to : chronic hepatitis (3.8±8.4 vs 1.3±2.2 PCRU, p=0.04), preoperative systemic chemotherapy (3.2±3vs 1.1±1.1 PCRU, p=0.04), surgical difficulty to mobilise the liver (3.1±3 vs 1.1±1 PCRU, p=0.01) and the weight of the resected liver (p=0.04); whereas, hepatic steatosis, the resection of the middle hepatic vein and the type or duration of vascular occclusion did not influence operative bleeding.
Conclusion : Major liver resection without blood transfusion is realistic. Predictive factors of operative bleeding include chronic hepatitis, preoperative chemotherapy and a difficult liver mobilisation. Resection of the middle hepatic vein do not influence operative transfusion.

### F237

### AUTOGENOUS PERITONEAL PATCHING OF THE INFERIOR VENA CAVA DURING *EN BLOC* HEPATIC RESECTION

PJ Gallagher and MS Stephen

*Department of Upper Gastrointestinal Surgery, Royal Price Alfred Hospital, Sydney. AUSTRALIA.*

When the inferior vena cava .(IVC) is involved with hepatic malignancy the best method of resection and repair is not certain. In 94 consecutive liver resections for cure of hepatic malignancy there were 6 patients that required *en bloc* resection of the IVC with the liver to obtain clearance of tumour. All patients had resection using total hepatic isolation with supracoeliac aortic clamping (THIS) and the resection was performed with scalpel. If the IVC was resected a patch of peritoneum backed by the posterior rectus sheath was harvested whilst the clamps were on from the upper medial myocutaneous flap. It was 'defatted', tailored to match and sutured to the IVC defect with the peritoneum on the 'intimal' aspect of the IVC.

3 resections were for colorectal secondaries, 1 was for hepatocellular carcinoma and 2 were for leiomyosarcoma secondaries. All resections involved the right liver and the amount of IVC to be replaced was a median circumference of 60% (range 30-70) over a median length of 5 cm (range 3-8). In no case was it necesssary to resect the entire IVC. Postoperatively no patient developed deep venous thrombosis or signs of IVC obstruction and 3 patients have had duplex ultrasounds and had no abnormalities detected.

*En bloc* hepatic resection with the IVC is possible and safe using THIS. Harvesting and application of the peritoneal patch is simple and does not greatly extend the procedure. Furthermore, the IVC is not narrowed as is possible with primary closure and the hazards of using heterogenous or prosthetic grafts are avoided

### F238

### OUR TECHNIQUE AND RESULTS IN THE LIVER RESECTIONS.

### F239

### SELECTIVE USE OF VASCULAR OCCLUSIONS IN LIVER RESECTIONS

D. Cherqui, R. Alon, N. Rotman, M. Julien, P.L. Fagniez. Department of Surgery. Université Paris XII-Hôpital Henri Mondor, Créteil, France

Control of intraoperative bleeding is a major prognostic factor for liver resections. Most popular vascular occlusion methods include the Pringle maneuvre (PM), and vascular exclusion of the liver (VE). Selective lobar inflow occlusion (SLO) is another possibility, rarely evaluated. The aim of the present study is to report the results obtained with these 3 methods used selectively according to the lesions in 60 major liver resections.

Methods. From 1989 to 1993, 60 major liver resections (≥ 3 segments) were performed. The method of vascular occlusion was addressed in view of preoperative imaging but the final decision was taken during surgery. VE was used in cases of tumors ≥ 10cm and/or located at the vicinity of the major hepatic veins and IVC. PM was used in cases of peripheral tumors < 10cm. Recently, we have used SLO in the latter type of lesions. Resections included right (29), extended right (12), left (15) and extended left (4) hepatectomies.

Results. Values in the table are means.

| | VE | PM | SLO | p |
|---|---|---|---|---|
| N | 22 | 19 | 19 | |
| extended resections | 10 | 5 | 1 | |
| tumor size | 12 cm * ** | 6,5 cm * | 8 cm ** | * ** 0.002 |
| ischemia time | 39 min | 36 min | | |
| transfusions | 3.8 units | 2.9 units | 1.2 units | NS |
| AST peak | 457 * | 297 ** | 182 * ** | * ** 0.003 |
| minimal PT | 57% | 54% | 52% | |
| no complications | 72% | 74% | 94% | NS |
| mortality | 1 (4.5%) | 0 | 1 (5%) | |

Switch from PM to VE was required in 1 case (5%). SLO used initially in 24 patients had to be switch to PM in 5 cases (20%). Patients and lesions were comparable in the PM and SLO groups. Global mortality was 3%.

Conclusions. These results suggest that adaptation of the type of vascular occlusion to the complexity of the resection is a safe and resonnable attitude. VE can be recommended in dangerous resections (large tumors, vicinity of the major veins). In cases of peripheral tumors 80% of right or left hepatectomies can be performed using SLO, thus avoiding ischemia of the remaining liver parenchyma.

### F240

### SUCCESSFUL RESECTION OF SOLITARY GIANT (>7CM) LIVER TUMOURS WITHOUT OPERATIVE MORTALITY

MN Hartley, A Leach*, J Harper*, R Edwards#, C Garvey#, D Smith+, S Myint+ and GJ Poston

Depts. of Surgery, Anaesthesia* & Radiology#, Royal Liverpool University Hospital and the Clatterbridge Centre for Oncology+

Twenty-five patients with solitary giant liver tumours (>7 [mean 10 (7-30)]cm) were assessed for operability by angiography and MRI. Five patients were initially deemed inoperable, of whom two underwent portal vein embolisation [PVE] to induce preoperative residual lobar hypertrophy. Twenty-two underwent exploratory laparotomy and 1 had an adrenal myolipoma replacing the right lobe of liver. Six were inoperable either due to involvement of portal structures in the uninvolved lobe (5) [including 1 following PVE] and one because of caval infiltration. Fifteen [7 male, 8 female, mean age 58 (39-82) years] [metastases: 7 colorectal; 1 carcinoid, 5 hepatomas (2 cirrhotic),1 FNH, 1 angiolipoma] were successfully resected without mortality. Two tumours were predunculated, 12 underwent conventional hemihepatectomy and 1 required trisegmentectomy. Median operative duration 5 [2-10] hours; median blood loss 1.5 [0.2-10]L; median postoperative stay 9 [6-15] days. Two suffered significant postoperative morbidity including adhesive obstruction and cholestasis, both settling spontaneously. One patient with metastases died 8 months after surgery and 3 with hepatomas died 4, 8 and 12 months later. Median survival is currently 10 [4-30] months.

Most solitary giant liver tumours can be resected safely with minimal morbidity. We recommend that all such patients be referred for assessment at specialist centres.

### F241

THE PROPER ALGORITHM IN TREATMENT OF HEPATOBLASTOMA (CASE STUDY)

Z.Kekić, Z.Barjaktarević, Lj.Maksimović

Zvezdara University Hospital, Belgrade, Yugoslavia

We present a case of a 3.5 year old girl with huge hepatoblastoma.After the laparatomy had been done and the liver tumor biopsy was taken,the diagnosis was established.Tumour was composed of malignant cells of an epithelial and mesenchymal origin fetal and embryonic type.We have established chemotherapy before surgery.Tumour regression was enormous and also followed by decrease of serum alfa-fetoprotein.Resection of the left lateral hepatic segment was performed afterwards.The second chemotherapy cycle was ordered after the surgical treatment had been done. The recovery of the patient was successful.The girl is nowadays, three years after surgery, well and living with no sign of recurrence although this case initially seemed hopeless. We think that the clue for the cure of this patient was the proper timing for performing surgery and the right choice of chemotherapy.The combination of pre and post operative chemotherapy and surgery done at the best possible moment is extremely important for this type of hepatic malignant tumour.

### F242

HEPATIC RESECTION IN THE TREATMENT OF LIVER TUMORS

Gr. Karatzas, E. Misiakos, M. Safioleas, Gr. Kouraklis, G. Eleftheriou, N. Givalos , C. SIMOPOULOS
Second Department of Propedeutic Surgery, University of Athens Medical School, Athens, Greece

The role of hepatic resection in the treatment of liver tumors is well established. During the period 1984-1994, hepatic resections were carried out on 16 patients with liver tumors. There were 8 men and 8 women within an age range of 45-82 years. Ultrasonography, computerized tomography and selective angiography were mainly used for diagnosis. Histologically, there was hepatocellular carcinoma (HCG) in 9 patients, metastatic tumors in 5 patients, cholangiocarcinoma in one patient and adenoma in one patient. Primary focus of metastatic tumors was: lung carcinoma in one case, ovarian carcinoma in one case and colorectal carcinoma in 3 cases. Three patients with HCG had associated liver cirrhosis and one patient had chronic hepatitis. The type of resections performed were: 3 right lobectomies, 5 left lobectomies, 4 segmentectomies and 4 non-anatomical resections. Operative mortality rate within 1 month was 6%. Mean survival in patients with malignant disease was 17.3 months. One patient with a benign adenoma and four patients with malignant tumors are alive: 2 of them have recurrent disease and 2 are disease-free. Our study suggests that hepatic resection in patients with liver neoplasms offers satisfactory local control with an acceptable long-term survival.

### F243

PORTAL HEMODYNAMICS OF PATIENTS WITH ASCITES, TREATED BY PERITONEOVENOUS SHUNT

P.K. Kupcsulik,L.Harsanyi,Gy.Szekely,F.Puskas
1st Dept of Surgery of Semmelweis Univ.of Medicine, Budapest,HUNGARY

75 Patients with intractable ascites underwent peritoneovenous shunt implantation for relief their symptomps.All of them were investigated pre- and postoperatively concerning the portal blood flow,blood velocity,cross section of the main portal vein,Doppler spectra of hepatic vein and artery, estimation of liver and splenic size.

Portal blood flow increased significantly by 38,9 percent in patients with properly functioning shunts and decreased by 7,5 percent in non functioning shunts. During a two year postoperative observation period preoperative portal blood flow, and postoperative increase of it showed a positive correlation to the late survival of patients.Alteration in portal flow were not caused by portal diameter changes.The hepatic venous spectra remained unchanged,and the hepatic artery Doppler spectra showed various alterations. Changes in liver diameter were not significant.Elevation of splenic size seems to be a negative prognostic parameter for postoperative survival.

Preoperative portal flow has no prognostic effect on postoperative shunt occlusion. Peritoneovenous shunt implantation results a singificant increase of portal and, as a consequence of it, that of hepatic blood flow.Significant improvement of laboratory data,and general condition was observed in these cases.

### F244

MULTICENTRE TRIAL OF OCTREOTIDE VERSUS INJECTION SCLEROTHERAPY (IS) FOR ACUTE VARICEAL HAEMORRHAGE

SA Jenkins[1], R Shields[1], R Sutton [1], AN Kingsnorth[1], M Davies[2], E Elias[2], AJ Turnbull[3], MF Bassendine[3], OFW James[3], JP Iredale[4], SK Vyas[4], MJP Arthur[4]
Royal Liverpool University Hospital, Liverpool; Queen Elizabeth Hospital, Birmingham, Freeman Hospital, Newcastle-upon-Tyne; Southampton General Hospital, Southampton

The role of octreotide in the management of variceal haemorrhage remains controversial. We conducted a multicentre trial to assess the use of 50 ug/h intravenous octreotide for 48 h to control variceal haemorrhage. Consecutive patients with endoscopically confirmed variceal haemorrhage were randomised to either octreotide (n=73) or emergency IS (n=77). Overall control of bleeding was not significantly different between octreotide (85%) and IS (82%) over the 48 h trial period (relative risk of rebleeding 0.83; 95% CI 0.38, 1.82). Octreotide was as effective as IS irrespective of the severity of the liver disease, and in those actively bleeding at endoscopy. Mortality during the 48 h trial period was identical in the two groups, but more patients died in the octreotide group during 60 days of follow-up, although this did not reach statistical significance (relative risk of dying at 60 days 1.91; 95% CI 0.97, 3.78; p=0.06). The results of this study indicate that intravenous octreotide is as effective as IS in the control of acute variceal haemorrhage. However, in view of the trend towards an increased 60 day mortality in the octreotide group, further trials are necessary to evaluate its safety in variceal bleeding.

### F245

### REGIONAL PORTAL HYPERTENSION

D.Voros, A.Prachalias, E.Mallas, D.Mourikis, J.Papadimitriou
2nd Dept. of Surgery, University of Athens (Aretaeion Hospital)

The so called Regional or left sided Portal Hypertension became more known during the recent years due to the progress in angiography and haemodynamic studies. It presents with increased portal pressure in the area of splenic circulation. The varices are present in the lower esophagus and very often in the fundus of the stomach.

The causing factors can be divided into two groups a) Diseases causing thromposis of the splenic vein: pancreatitis tumors, trauma and b) conditions leading to hyperdynamic circulation in the area of the spleen: congenital arteriovenous fistulas, splenomegaly secondary to haematologic diseases. In any case regional portal hypertension is characterised by the absence of hepatic disease.

We present 5 cases from a series of 72 patients with portal hypertention operated in our Dept. during the last 10 years (7% of the total number). There were 3 male and 2 female patients, their age was 27-77 years. In 3 cases splenic thrombosis was diagnosed. History of the patients or co-existing diseases included: thrombocyttopenia (1), Myelodysplastic syndrome and splenomegaly (1) Pancreatitis (1), chronic renal failure (1). In one case no other disease was disclosed. All patients had experienced bleeding from esophageal varices and all but one had big gastric varices at endoscopy. Preoperative investigation included angiography of the portal system (U/S, Celiac axis, Panangiography, splenoportography) and liver biopsy.

In 4 of our cases an elective splenectomy was carried out as the treatment of choice with excellent results at a follow up 8 months to 9 years later. In the fifth case a spleenectomy was considered to be dangerous because of perisplenic fibrosis; ligation of esophagogastric veins and transection of the gastric fundus were carried out. The patient died one month later due to the renal disease.

The regional portal hypertension is not so rare as it was thought before the angiographic studies become widely available. Suspicion may arise in every case of bleeding varices in the abscence of liver disease. The confirmation is given by imaging techniques. This form of bleeding is the only one treated successfully by as simple an operation as the elective spleenectomy.

### F246

### BLEEDING FROM OESOPHAGEAL VARICES: IS STILL A PLACE FOR NON-SHUNT SURGICAL TREATMENT?

T.Kościński, S.Malinger, M.Smoczkiewicz
Department of General and Gastrointestinal Surgery, University Hospital, Poznań, Poland

Authors present the results of non-shunt surgical strategy for ruptured oesophageal varices. Endoscopic injection sclerotherapy was the treatment of choice to stem haemorrhage in 158 patients. Variceal haemorrhage was successfully controled in 125 patients. Recurrent bleeding despite of injection sclerotherapy appeared in 33 patients. They had to be operated on. Surgical treatment consisted in three methods of non-shunt operations: devascularisation of lower oesophagus, cardia and gastric fundus (7 patients) Sugiura technique (5 patients) and oesophageal stapler transsections (21 patients).

Results. Endoscopic obliteration of bleeding oesophageal varices was efficacious in 80% patients However in 16 cases some complications were observed: one oesophagus perforation, 5 oesophageal strictures and 10 exudative pleuritis. 20% of patients required a surgical treatment. After oesophageal and gastric fundal devascularisation 6 patients rebleeded and 4 of them died. One death after Sugiura operation was due to an anastomotic leak in the mediastinum. The best results were obtained after stapler transsections of the oesophagus. There were 4 important rebleedings and 2 anastomotic leaks. Five patients died postoperatively. After one year 9 variceal relapses were observed.

Conclusions. 1.Endoscopic injection sclerotherapy is efficacious, first choice treatment for haemorrhage from oesophageal varices. 2. The surgical treatment should be reserved for ineficient endoscopic control of bleading.

### F247

### HIGH CUMULATIVE MORTALITY FROM RECURRENT VERSUS FIRST VARICEAL HAEMORRHAGE UNDERLINES NEED TO PREVENT REBLEEDING

N Howes, A Larman, M Thomas, S Suares, SA Jenkins, *IT Gilmore, *AI Morris, R Shields, R Sutton
Departments of Surgery and *Medicine, Royal Liverpool University Hospital, Liverpool, UK

Variceal haemorrhage carries high risks of rebleeding and mortality that decrease with time. However, the risks of death from first versus subsequent bleeds have not been defined. We studied 220 patients (142 alcoholic cirrhotics) recorded on a prospective database from 1983 to 1992 to determine the risk of death from either first or recurrent variceal haemorrhage. Patients were treated with vasoactive agents and injection sclerotherapy. Preliminary balloon tamponade, subsequent oesophageal transection, or portasystemic shunting were undertaken in a minority with uncontrolled bleeding. There were 166 patients admitted with first variceal haemorrhage, of whom 48 (29%) died during their first hospital episode. There were 213 admission episodes of recurrent variceal haemorrhage in 89 patients, of whom 43 (20% of episodes, versus mortality of first episodes $Chi^2 = 3.78$, $p<0.05$ ; 48% of patients with recurrent haemorrhage, versus mortality of patients with first bleeds $Chi^2 = 9.47$, $p<0.01$) died during these 213 episodes.

These results suggest that although the risk of death from admission episodes for recurrent variceal haemorrhage is lower than for first variceal haemorrhage, treatment to prevent rebleeding should be assiduous, as the cumulative risk of death from all admissions for rebleeding is almost double that of first variceal haemorrhage alone.

### F248

### MESOCAVAL SHUNT VERSUS RADIOLOGICAL INTERVENTION IN THE TREATMENT OF BUDD-CHIARI SYNDROME.

A.E.A.MAHMOUD, D.GAKIS, S.OLIFFE, R.WEST, J.BUCKELS, E.ELIAS
The Liver & Hepatobiliary Unit, Queen Elizabeth Hospital, Birmingham, UK.

Mesocaval shunt (MCS) and radiological intervention methods (RIM) in the form of balloon angioplasty or stent placement, have been reported to successfully treat manifestations of portal hypertension in Budd-Chiari Syndrome(BCS). To compare between these two treatment modalities, we have analyzed the data of 25 patients with BCS admitted between 1984 & 1994. All data were collected at the time of presentation. 11 patients were treated with a RIM (mean age 40.1 years, range 21-65) and 14 patients were treated with a MCS (mean age 33.8 years, range 22-54). Mean LFTs values (RIM v MCS) were; AST 152 v 286, Bilirubin 50.7 v 77.5, Alkaline phosphatase 364 v 503 & Albumin 31 v 32. Clinical presentations, in particular, incidence of encephalopathy was comparable in both groups. Etiologically there was higher incidence of Myeloproliferative in the MCS group. The following table summarizes the outcome in both groups.

| | RIM(%) | MCS(%) |
|---|---|---|
| CLINICAL & BIOCHEMICAL IMPROVEMENT | 9/11(82%) | 7/14(50%) |
| ONE YEAR MORTALITY | 2/11(18%) | 4/14(28%) |
| COMPLICATIONS | 1/11(9%) | 3/14(21%) |
| FOLLOW UP(MEAN) | 2.3 years | 2.5 years |

CONCLUSION: The outcome of radiological intervention is comparable to MCS in the treatment of BCS. Because it is the most physiological treatment, we believe it should be first line treatment in patients with BCS. MCS and liver transplantation should be only used if RIM is unsuccessful.

### F249

IMMUNOHORMONAL CHANGES DURING THE SURGICAL TREATMENT OF PORTAL HYPERTENSION

M.Pavlovsky, S.Chooklin, A.Perejaslov
Department of Surgery, Medical Institute, Lviv, Ukraine

The liver cirrhosis is the most common cause of the portal hypertension. The remote results of the splenorenal anastomosis execution of the patients with liver cirrhosis were observed. Immune and hormonal monitoring, which can judge about the functional condition of the organism, was performed. It was shown, that the put-on splenorenal venosis shunt increased antigen stimulation and loading on the liver. The suppressors control is weakening, with the autoimmune processes activating, the polyclonal immunoglobulinemia and decreasing of the total complement. The suppressors function of the monocytes is significantly weakening (by the prostaglandin E2 synthesis) and accordingly the level of the cAMP in the T-lymphocytes was decreased with the disturbance of the cyclic nucleotides correlation. Requirements of the organism in vasopressin, which regulate arterial and decreased portal pressure, increased. Decrease of prostaglandin E2 synthesis supported the ishemic changes in the liver. The quantity of the thrombocytes does not increase. Decrease of the prostacyclin level intensified the microcirculatory disturbances and stimulated the processes of the thrombocyte aggregation with the intensification of the reaction of liberate. The separating of fibrinopeptide A from fibrinogen is increased. It points out to the danger of thrombohemorrhagic complications' development. Thus, the put-on splenorenal anastomosis on liver cirrhosis promotes the pathological processes' progress and increases the risk of complications and lethal outcome.

### F250

**TRANSJUGULAR LIVER BIOPSY COMBINED WITH HEPATIC VENOUS PRESSURE MEASUREMENTS.**

**K.Malagari*, I.S.Kaskarelis*, A.Pavlopoulou*, P.Koutsogeorgas*, M.Katsarou*,M.Papadaki*, M.Pittaridis**, E.Douzinas**, Roussos Ch**.
*Department of Radiology, **Department of Critical Care Evangelismos Hosp. Athens Greece**

Transjugular liver biopsy (TJLB) is a well-known biopsy technique, advocated mainly in patients with a bleeding tendency in whom the risk for percutaneous biopsy is unacceptable. In addition, the venous approach gives direct access for free and wedged hepatic venous pressure (VP) measurements that represent an index of portal venous pressures.

In this paper 18 cases are presented in which only TJLB was performed and 7 cases in which TJLB was combined with VP measurements. Issues related to the technique and possible complications are addressed.

The mean age of the patients was 48yrs (37-74). All but 2 had bleeding diathesis, 2 had ascites and 2 were also septic. An average of two tissue samplings was performed in each patient and an adequate tissue specimen was obtained in 86% of tissue specimens and in all but one patient. Pressure measurements were obtained in a wedged and free position of the catheter that was connected with the monitor. There were no major complications.

It is concluded that TJLB is a workable, efficient and safe procedure for liver biopsy in high risk for a percutaneous approach pts and that combined with VP measurements can be essential for dedicated departments of hepatology.

### F251

ELECTIVE TREATMENT OF ESOPHAGEAL VARICES: A COMPARATIVE STUDY.

M. ABU ZEID, A. SULTAN, N. EL-GHAWALBY, K. ABU EL-MAGD AND F. EZZAT.
Gastro Enterology Surgery Center, Mansoura, Egypt.

This study was performed to compare different therapeutic modalities for treatment of esophageal varices .
Between March 1990 & June 1991, 60 Child A/B patients were randomly treated by distal splenorenal shunt (DSRS,n=20), splenectomy & devascularization (S&GED,n=20) or sclerotherapy (EVS,n=20). The patients were followed up for 22.1 $\pm$ 5.7 months (DSRS), 20.7 $\pm$ 5.5 months (S&GED) and 25 $\pm$ 5.8 months (EVS) .
The mortality rate was 10% after DSRS, 5% in each of the other 2 groups. Rebleeding occurred in 21% (DSRS), 13.6% (S&GED), and 33% (EVS). Encephalopathy occurred only after DSRS (26.3%). Hepatopetal portal perfusion was maintained in 77.8% after DSRS, 88.9% after S&GED and 100% after EVS. portal vein thrombosis occurred only after surgery, 5.3% after DSRS and 22.2% after S&GED. In conclusion, while DSRS is a good therapeutic modality to treat esophageal varices, S&GED or EVS are satisfactory alternatives for Child A/B patients with esophageal varices.

### F252

SURGICAL MANAGEMENT OF HEPATIC METASTASES FROM COLORECTAL CANCER.

J.Poulantzas,M.Lorentziadis,S.Stylogiannis,S.Prigouris, G.Verikokakis,P.Markidis.
4th Depart. of Surg."Evangelismos"Hosp. & 1st Depart.of Surg."General Hospital" of Athens.

It has been shown that approximately 70% of patients with resected hepatic metastases from colorectal cancer,present recurrences within subsequent 5 years.However,it is recognized that no other method of treatment has a currative potential comparable to that of surgical resection and metastasectomy.Over ten years period,14 patients underwent liver surgery for metastases of colorectal origin at the 1st Depart. of Surgery of the "General Hospital" of Athens and the 4th Depart. of Surgery of "Evangelismos" Hospital. These patients,represent the 3,7% of all cases operated for G.I. tract cancer,the 0,8% of cases operated for colorectal cancer and the 2,3% of patients with hepatic metastases from colorectal origin admited during the 10 years period.There were 8 men and 6 women,the mean age being 62,1 years(range :37-68 years).Operative strategy included:right lobectomy 1,left lateral lobectomy 4,segmentectomy 2,wedge resection 4,enucleation 3.The overall mortality rate was 0% and the morbidity 28.6%.Two and 5 years survival rate was 58,4% and 16.6% respectively.None of the patients received adjuvant treatment.
Conclusions:The results of this Greek study after synchronous or metachronous liver surgery for metastatic colorectal carcinoma are favorably compaired with those reported in earlier European studies and reason the practice of hepatic resection of metastases from cancer of colorectal origin.

### F253

### OILY CHEMOEMBOLIZATION (OCE) OF HEPATIC MALIGNANCIES

A.M.Granov, P.G.Tarazov, D.A.Granov
Department of Surgery and Division of Angio/Interventional, St.Petersburg Research Institute of Roentgenology and Radiation Therapy, Russia

We evaluated long-term results of OCE in unresectable hepatic malignancies. OCE was performed in 120 patients with hepatocellular carcinoma (HCC,35 cases), other primary liver tumors (10), and hepatic metastases (Mts,75). A new liposoluble cytostatic Dioxadet (15-20 mg) or Doxorubicin (30-100 mg) in iodized oil were used. One to seven OCEs were performed followed in 1/3 of pts by injection of the same drugs in oil into the portal vein. Recently, 2 to 4 g of ferromagnetic $Ba_2Fe_2O_6$ were added to embolization mixture in 10 pts and OCE was performed under local magnetic field followed by 1 to 3 sessions of local hyperthermia. The 1-2-3yr survival was 94%,67%,27% for HCC, 47%, 25%,6% for colorectal, 88%,80%,60% for carcinoid, and 60%,30%,10% for other liver Mts. More extensive tumor necrosis was seen in cases treated by OCE+hyperthermia, but long-term results are in the future. The results showed that OCE prolonges survival in at least 1/2 of patients with unresectable hepatic malignancies. Better results are seen in HCC and carcinoid liver Mts. Combination of OCE and ferromagnetic assisted hyperthermia seems to be promising treatment of hepatic tumors.

### F254

### SOMATOSTATIN RECEPTOR SCINTIGRAPHY IN PATIENTS WITH LIVER METASTASES OF NEUROENDOCRINE TUMORS

A. Frilling, X. Rogiers, M. Malago, K. Platz, C.E. Broelsch
Dept. of Surgery, Universitiy Clinic, Hamburg, Germany

**INTRODUCTION**
The decision for resection of hepatic metastases of neuroendocrine (NE)tumors is mainly triggered by severe hormonal symptoms and the absence of extrahepatic tumor spread. In tumors rich on somatostatin receptors somatostatin scintigraphy (SMS) provides a new method in detection of tumor extension.

**PATIENTS AND METHODS**
14 sympomatic patients with hepatic metasteses of NE tumors have been referred to us for liver resection. In all of them standard imaging methodes confirmed liver metastases in absence of extrahepatic tumor lesions. Additionaly SMS scintigraphy was carried out.

**RESULTS**
In 7 patients liver metastases and extrahepatic tumor deposits were demonstrable in SMS scan. 6 of these patients underwent systemic somatostatin therapy. Due to local compression caused by huge tumor masses in one patient tumor debulking was performed. 2 patients were operated for local tumor recurrence detected by SMS scan. One patient with unknown primary tumor side prior to scintigraphy underwent liver resection and short bowel segmentectomy. In 2 patients with isolated liver metasases segmental resection was done. Due to disseminated hepatic tumor spread and severe carcinoid syndrom two patients underwent liver transplantation.

**CONCLUSION**
SMS scintigraphy is highly sensitive in detection of NE tumors. It should be carried out prior to liver surgery for NE metastases in order to detect possible extrahepatic tumor lesions and avoid oncologically not indicated liver surgery.

### F255

### PREOPERATIVE EVALUATION OF LIVER MASSES: ROLE OF SPIO-ENHANCED MR-IMAGING AS A NON-INVASIVE IMAGING TECHNIQUE

C. Stoupis[1,2], P. Vock[1], H. Baer[2], M. Büchler[2], P.R. Ros[3]
1) Department of Diagnostic Radiology and
2) Department of Visceral Surgery, University Hospital of Berne, Switzerland
3) Department of Radiology, University of Florida, USA

Purpose: To assess the efficacy of SPIO (super paramagnetic iron oxide) MR imaging in detecting and characterizing liver lesions before surgery.

Methods: 65 patients with known or suspected liver lesions were examined with enhanced computed tomography (CT) and SPIO-enhanced MR imaging. Qualitative and quantitative analysis were performed. Pathology was available in 50 patients.

Results: Post contrast T2-weighted images demonstrated marked decrease in signal intensity of liver (96%). This sequence provided improved liver lesion detection and diagnostic confidence compared to iodine-enhanced CT (70% and 77% resp.) and pre contrast MR (81% and 86% resp.). Post contrast MR images revealed in 37% of cases lesions not detected on either pre contrast MR or CT scan. SPIO was not seen in malignant lesions but only in benign ones. SPIO MR imaging differentiated focal nodular hyperplasia cases (n=10) and hemangioma cases (n=8) compared to hypervascular metastases.

Conclusion: MR imaging with SPIO-enhancement has the potential to be the non-invasive method of choice in the preoperative evaluation of liver patients due to its efficacy to detect and potentially characterize liver lesions.

### F256

### SURGICAL INDICATIONS TO REPEAT LIVER RESECTIONS FROM COLORECTAL METASTASIS.

Ciferri E., Gazzaniga G.M. , Filauro M.
1st Surgery Dept. - S. Martino Hosp.- Genoa - Italy
Viale Benedetto XV, 10 - 16132 Genoa - Italy

The surgical attitude towards hepatic metastases has radically changed in these last years. A deeper knowledge of the liver's anatomy and of the natural history of the metastatic process have induced many surgeons to repeat resections even on recurrent hepatic metastases.
At the moment surgery is the only solution that offers the patient a 5 ys overall survival rate of 26-30%.
Our experience is reported in Tab.I: 8 cases; average age 60 (range 54-69)

| Tab.I: | age (sex) | prim.tum. (staging) | d.f.i. (mth) | 1st res. | d.f.i. (mth) | 2nd res. | surv. (mth) | state |
|---|---|---|---|---|---|---|---|---|
| 1 BE | 57 (M) | ciecum (B2) | 0 | 1 L wr | 17 | 4 R wr | 124 | ANED |
| 2 PE | 59 (M) | rettum (B2) | 36 | segm 7 | 17 | R lob | 28 | DWD |
| 3 BG | 60 (M) | sigm. (B2) | 36 | L lob | 6 | 2 R wr | 5 | DWD |
| 4 RM | 55 (M) | rettum(C1) | 0 | 1 L wr | 11 | R lob | 14 | DWD |
| 5 NE | 54 (M) | l. colon (C1) | 19 | segm 7+8 | 30 | 2 R wr | 39 | AWD |
| 6 ZE | 64 (M) | sigm. (C2) | 1 | R lob | 17 | 1 R wr | 18 | DWD |
| 7 TG | 69 (F) | sigm. (C1) | 0 | 1 L wr | 19 | 1 R wr | 14 | ANED |
| 8 PL | 59 (F) | sigm. (C1) | 55 | R lob | 7 | 1 L wr | 4 | ANED |

ANED=alive not evidence disease; DWD/AWD= died/alive with disease

All patients were free of symptoms at the diagnosis, which was always made during follow up with blood examinations, testing CEA and CA 19.9, chest X-ray, US/CT/NMR. The preliminary results from a multiinstitutional study (Registry of repeat resections of hepatic metastases) directed by P.H. Sugarbaker, started in 1991, that included 170 patients from 20 institutions, have emphasized although not all the data were statistically significant, the importance of some factors as the numbers of mets (< 4), the limited distribution in the liver and the non infiltration of important vascular structures, the absence of extrahepatic diseases, are intended as distant, local or lymphnodal.
Among these last it's very important to know the status of hepatoduodenal lymphnodes, considered the road of metastatic cells' escape ("mets from mets - cascade phenomenon").
When it 's impossible to perform a radical operation, from the oncological point of view (adequate extension of resection and respect to clear margin), surgery loses every curative capacity. New studies on chemotherapy, after obtaining the first encouraging results, are now trying to extend the possibility of surgery.

### F257

### LIVER RESECTION FOR METACHRONOUS METASTASES OF RENAL CELL CARCINOMA

H. Lang, J. Jähne, C. Stief, R. Raab, R. Pichlmayr
Klinik für Abdominal- und Transplantations-chirurgie, Medizinische Hochschule Hannover

Metastases of renal cell carcinoma are frequent and represent a major therapeutical challange. In a retrospective study we evaluated the results of surgical treatment in metachronous liver metastases.
Overall, the charts of 15 pts. (10 male, 5 female) were analysed. Median time between nephrectomy for the primary tumor and diagnosis of liver metastases was 3.6 years. In 11/15 pts. (73.4%) resection of the metastases was possible. Operations were: wedge excision or segmentectomy (n=3), hemihepatectomy combined with a multivisceral resection if required (n=7), ante-situ liver resection (n=1). A potentially curative resection (R0) was achieved in 9 pts. (81.1%). Postoperative morbidity and mortality were 54.4% (n=6) and 36.4% (n=4). Median survival after explorative laparotomy was 4 months compared with 14 months after resection (range 9-72 months; p<0.05).
Liver resection for metachronous metastases of renal cell carcinoma concurs with a high rate of morbidity and mortality, especially after combined multivisceral resections. Although potentially curative resections lead to a significant longer survival the prognosis remains poor. Further studies are necessary to evaluate the role of multimodality treatment regimens (surgery/tumor-vaccine/interferon) in this entity.

### F258

### SURGICAL TREATMENT OF COLORECTAL HEPATIC METASTASES: SHORT AND LONG TERM RESULTS FOLLOWING 65 LIVER RESECTIONS.

S. Alfieri, G.B.Doglietto, F. Pacelli, C. Carriero, F. Crucitti.
Department of Surgery - Catholic University - Rome - Italy

AIM: Aim of the present study is to retrospectively evaluate our experience with 65 hepatic resections for colorectal liver metastasesy performed in our unit.PATIENTS AND METHODS: sixtyfive liver resections were consecutively performed in our unit between January 1983 and December 1993, upon 59 patients with hepatic metastases from colorectal cancer. Among these patients, four had a second hepatic resection (after 10, 16, 22, and 30 months) and one had a second and, successively, a third hepatic resection for recurrence, (after 16 and 14 months respectively). Surgery was considered radical where no gross residual disease was evident within or outwith the liver, when at least 1 cm of normal parenchyma sorrounded the resected tumor and when microscopic invasion of resected margins as well as lymphnode metastases were not detected histologically. Some 3 patients therefore underwent palliative resection. The lenght of operation, intraoperative blood loss, post-operative morbidity rate, hospital mortality rate and 5-year survival after curative resection were recorded. After discharge from hospital, patients were followed up with the measurement of CEA in serum and US every 3-months. Survival has been calculated as the interval from liver resection to death or last follow-up (May 1994) for alive patients. Only patients undergoing curative resection were included, after exclusior of operative deaths. Therefore, survival after resection was studied in 49 patients. Five patients (8.5%) were lost to follow-up. Survival rates were calculated by the life-table methods of Kaplan-Meier. RESULTS: there were 25 women and 34 men with ages ranging from 30 to 78 years (mean 58.1). The mean operative time was 258.3 min: 293.84 min and 210.6 min for synchronous (n.38) and metachronous (n. 21) lesions respectively. The mean intraoperative blood transfusion requirement was 486.5 ml (range 0 - 3000 ml): 561 ml and 433 ml for sinchronous and metachronous metastases respectively. Twentyeight (43%) patients have not required any blood transfusion. Mean hospital stay was 17.1 days (range 8 to 47), 18.7 days and 15.5 days for patients with synchronous and metachronous lesions respectively. The morbidity and mortality rates were 15.3% and 3% respectively. Overall survival at 5 years was 24.1% and disease-free survival was 20%. CONCLUSION In our series, low mortality and morbidity rates after the first liver resection and the zero mortality and 16.6% morbidity rates in the six reoperation for recurrence confirm to the safety of liver surgery for colorectal liver metastases. Repeated hepatectomy for recurrent liver metastases did not increased the post-operative mortality and morbidity rates, compared with primary liver resection.

### F259

### MORBIDITY OF HEPATIC ARTERIAL CHEMOTHERAPY - IMPACT OF SURGICAL TECHNIQUE

K.-P. Riesener, R. Kasperk, P. Klever, V. Schumpelick
Department of Surgery, Medical Faculty, Rhenish Westfalian University of Technology Aachen, Germany

Port systems for hepatic arterial infusion chemotherapy (HAI) have been developed as a cost-effective alternative towards implantable infusion pumps. This study was performed to evaluate the kind and frequency of complications following the implantation of port systems for HAI.

**Methods:** From 1.1.1986 to 31.12.1993 port systems were placed into the gastroduodenal artery in 111 patients with hepatic metastases. A total of 512 chemotherapeutic courses (six days short term infusion each) were administered using 5-FU and Mitomycin C. The functioning of the port system was checked by contrast radiography prior to each course.

**Results:** 59 complications were recorded in 52 patients (47%). The most frequent were dislocation or leakage of the system (n = 14), port occlusion ( n = 13), and thrombosis of the hepatic artery (n = 8). Reoperations were performed in 29 patients, port function could be reestablished in 11 of these cases. The majority of the technical complications were observed in the beginning of our study, the rate was lowered with increasing experience and improved technique.

**Conclusions:** HAI using implantable port systems is accompanied with a reasonable rate of complications. Increasing experience with the method of implantation is followed by a significant reduction of technical problems.

### F260

### Repeat Hepatic Resections for Colorectal Metastases to the Liver

WT Knoefel, C Brunken, M Gundlach, X Rogiers, JR Izbicki, CE Broelsch
Department of Surgery, University of Hamburg, Hamburg, Germany

**Introduction:** A surgical approach to recurrent colorectal metastases to the liver is rarely taken. To specify a rational approach to these lesions we reviewed our experience of the last 6 years.

**Methods:** 11 repeat hepatectomies were performed in 10 patients over the last 6 years at our department. The median interval since first resection was 12 months. 5 primary resections were anatomic resections, 6 were atypical resections. Exclusion of extrahepatic disease, control of the primary tumor and intraoperative ultrasound to exclude further intrahepatic disease were required in all patients.

**Results:** There was no perioperative mortality. 2 patients developed pleural effusions that were treated by conservative means. Performed operations were: 6 hemihepatectomies, 2 segmentectomies, and 3 wedge resections. 2 of these were extended resections. In 10 patients no residual tumor was left behind whereas in 1 patient the resection margin was microscopically involved. Median postoperative hospital stay was 11 days. A median of 2 transfusions (maximum 4) were required. The median survival is currently 27 months. 3 patients died from tumor relapse. The median disease-free survival is 20 months. 5 patients developed a tumor relapse after a median interval of 13 months. 2 of these relapses were intrahepatic. Relapse was treated by liver resection (n = 1), hepatojejunostomy (n = 1), or chemotherapy (n = 3).

**Conclusions:** Hepatic resection for recurrent colorectal metastases to the liver is safe. Prognosis of this highly selected group is comparable to that of patients undergoing primary resections. Approach to recurrent metastases should therefore be as aggressive as to primary metastases.

### F261

### THE ROLE OF E-CADHERIN IN THE COLONY FORMATION, INVASION, AND DISSOCIATION OF CANCER CELLS

WG Jiang, S Hiscox, MCA Puntis, MB Hallett
University Department of Surgery, University of Wales College of Medicine, Cardiff, UK

E-cadherin is a cell surface adhesion molecule which is involved in cell-cell adhesion. This study was to determine the role of this molecule in the colony formation, *in vitro* invasion, and dissociation of cancer cells. Human cancer cell HT115, HRT18, and HT29 cells were used. Cell surface E-cadherin was determined by flow cytometry, Western blotting, and immunohistochemical studies. All cells showed positive staining for E-cadherin on their surface, particularly on cell-cell junctions. HT115 stained more weakly and others stronger. This was confirmed by Western blotting analysis. The morphology revealed a loose colony with HT115 and much tighter colonies with others. A neutralising anti-E-cadherin antibody caused a complete scattering of HT29 and HRT18 colonies, and made HT115 colonies even looser. Cell dissociation was determined by a Cytodex-2 dissociation assay. Anti-Ecadherin antibody showed a significant promotion of cell dissociation from the carrier beads (dissociation index mean±SEM). E-cadherin antibody also promoted cell invasion into the basement membrane, Matrigel (shown as invasion index).

| | Control | plus anti E-cadherin antibody |
|---|---|---|
| Colony scatter | - | + |
| Dissociation index | 3.7±0.2 | 4.4±0.3* |
| Invasion index | 1.0±0.1 | 4.9±0.2* |

HT115 cells, HECD1 shown as at 100ng/ml, * $p<0.05$ vs control

In experiments designed to test the attachment of these cells to extracellular matrix, anti-E-cadherin antibody failed to show any effects on the attachment of all three cells to Matrigel, a reconstituted basement membrane, indicating E-cadherin play a less important role in the cell-matrix interaction.

We conclude that E-cadherin play a key role in the formation of colon cancer colonies and loss or dysfunction of this molecule promotes motility and dissociation of these cells, which will facilitate the formation of liver metastasis.

### F262

### EFFECTS OF INTERFERON ON SERUM LEVELS OF $\beta_2$ MICROGLOBULIN, C-REACTIVE PROTEIN AND SOLUBLE INTERLEUKIN - 2 RECEPTOR IN PATIENTS WITH CHRONIC HEPATITIS B AND C

Ü.Sarıtaş, K. Dalva, U. Yılmaz, S. Küçükbaş, E. Altıparmak, F. Demirci, T. Şahin

Department of Gastroenterology, Yüksek İhtisas Hospital, TURKEY

Serum $\beta_2$ microglobulin ($\beta_2$MG), C-reactive protein (CRP) and soluble interleukin-2 receptor (SIL-2R) levels were measured in 30 patients diagnosed of chronic active B and C hepatitis (CAH), in 16 chronic persistent hepatitis (CPH) patients or asymptomatic carriers (AC) who were followed-up without any therapy and in 20 healthy people (HP). The relationship between liver pathology and these parameters whether they were predictive factors in respect to interferon therapy and the mechanism of interferon effect were researched. These three parameters were measured before and after therapy and during the time of therapy every month with respect to ALT levels and response to interferon therapy.

In therapy group 14 patients were complete or in complete responsive to interferon therapy while 16 patients were unresponsive.

$\beta_2$MG, CRP and SIL-2R levels were significantly higher in CAH, CPH and AC group than HP group ($p<0.01$).

With respect to three parameters there was no significant difference between responsive and unresponsive patients. However in responsive B hepatitis group the level of $\beta_2$MG and SIL-2R reached to peak levels in the second month of therapy showing parallelism to high ALT levels and returned to normal value in 6 months. No significant difference was observed in chronic active C hepatitis group.

As a result $\beta_2$MG, CRP and SIL-2R levels were closely related with liver damage but they were not predictive factors for interferon therapy. In chronic active B hepatitis group the effect of interferon was basically immunomodulatory whereas in chronic active C hepatitis mostly antiviral.

### F263

### MORPHOLOGICAL STUDY OF FRESH ISOLATED AND CRYOPRESERVED PIG HEPATOCYTES, BEFORE AND AFTER XENOTRANSPLANTATION IN CASES OF TOXIC LIVER FAILURE

A. Papalois, N. Arkadopoulos, M. Demonakou, G. Kostopanagiotou, E. Manolis, B. Golematis, Th. Pataryas, J. Papadimitriou
2nd Department of Surgery, Department of Biology and 1st Department of Propaedeutic Surgery, University of Athens, Athens, Greece

Transplantation (Tx) of hepatocytes (Hcs) increase the survival rate of animals with acute liver failure (ALF) in different experimental models. The development of techniques of Hc isolation from a large mammalian followed by preservation of them have to be investigated because of the many clinical applications where can be used. The aim of this work was to develop a technique for isolation of pig Hcs and to study morphologically fresh isolated (FI) Hcs as well as cryopreserved (Cp) Hc (-20°C for a month) before and after xeno-Tx in rats with ALF. Two female pigs were used as donors of Hcs (20-22 Kg). For Hc isolation the liver was perfused with 10 lt of Lactated-Ringers (4°C) and preserved in University of Wisconsin solution for 10 hours. Then collagenase (Type V, Sigma C-9263) was infused via portal vein (1 liver with 1 mg/ml-case A and 1 with 1.3 mg/ml-case B) and incubated at 37°C (45 min). Cells were filtered and then washed in Hanks' solution. Their viability was assessed by trypan blue (>90%). In the second part of the study ALF was induced in 27 Lewis rats with N-Dimethylnitrosamine (N-DMNA, 20 mg/Kg iv) and transplanted intrasplenically and in kidney capsule after 24 h. Rats were divided in 5 groups: Group 1 (n=3): no treatment. Group 2 (n=6): Tx of Fl Hc (A). Group 3 (n=6): Tx of Cp Hc (A). Group 4 (n=6): Tx of Fl Hc (B) and Group 5 (n=6): Tx of Cp Hc (B). All rats were treated with cyclosporine A 20 mg/Kg/day (days 0-10) and 10 mg/Kg/day (days 10-20). Total experimental period 20 days. The survival rate at 20 days was 0% in Group 1, 33% in Group 2, 50% in Group 3 and 66% in Groups 4 and 5. Before Tx both Fl and Cp Hcs were found morphologically intact but in case A cells were more organized in clusters instead of single cells (case B). After 10 days post Tx cells were observed as cords of Hcs in the kidney capsule and in aggregates in the splenic parenchyma. Both Fl Hcs and Cp are similar effective.

We conclude that collagenase concentration has very important role in the success of Tx and the improvement of survival rate in case B is related directly.

### F264

### BACTERIAL TRANSLOCATION AND ENDOTOXEMIA IN HEPATECTOMIZED RATS

S Koureleas, J Kirkilesis, D Karavias, C Vaglanos, A Arvaniti, J Androulakis

Department of Surgery and Laboratory of Microbiology, University of Patras, Patras, Greece

At hepatectomy, the integrity of the intestinal mucosal barrier may become damaged, allowing bacteria and endotoxins to translocate to remote tissues, promoting a septic process. In this study, we examined endotoxemia and intestinal bacterial translocation (BT) in rats, following hepatectomy.

***Method:*** Male Wistar rats were used, divided into three groups: I (n=21), non-operated controls; II (n=11), sham hepatectomy and III (n=18), 70% hepatectomy. All animals were sacrificed, 48 hours later. Mesenteric lymph nodes (MLN) were cultured aerobically and liver specimens both aerobically and anaerobically. Endotoxin levels were measured in the portal and the systemic blood. By washing the colon with 2 ml of saline, 0.5 ml aliquots were obtained and aerobic gram-positive/gram-negative and total anaerobic population were determined.

***Results:*** All animals survived. Positive MLN cultures were found in 58% of the animals in group III ($p<0.05$ vs groups I and II). 30% of aerobic liver cultures were positive in group III, and none in groups I and II ($p<0.01$). All cultured bacteria were enteric in origin. All anaerobic liver cultures were negative. There was no significant difference in endotoxin concentrations in portal and systemic blood ($p>0.05$). Quantitavive determination of colonoc bacteria, also revealed no significant difference among the three groups ($p>0.05$).

***Conclusion:*** Fourty-eight hours after hepatectomy in rats, bacterial translocation but no endotoxemia was promoted. No difference in colonic enteric contents was found. Bacterial translocation might be explained by the operative stress, the decrease in the capacity of the liver to clear bacteria and the relative decrease in the amount of bile salts, entering the intestinal lumen after hepatectomy.

### F265

ENDOTOXIN (LPS) AND *LACTOBACILLUS* R2LC (LB) PRETREATMENT IN ACUTE LIVER INJURY

F.B. Kasravi, D. Adawi, G. Molin, B. Jeppsson, S. Bengmark
Dept. of Surgery, Lund Univ., Lund, Swden

Occurrence of bacterial translocation, serum endotoxin, liver histology and enzymes, intestinal microflora and mucosal changes, were evaluated in D-galactosamine liver injury, 3, 7, and 14 days after pretreatment with LPS and LB. Liver injury was minimal 3 days after pretreatment with LPS, while it was severe in all other groups, especially LB groups. High levels of serum endotoxin was present 3 days after LPS treatment, while it was absent in all other groups. No mucosal or microfloral change was seen in the experimental groups.

### F266

**E-SELECTIN AS A MARKER OF REPERFUSION INJURY IN HUMAN ORTHOTOPIC LIVER TRANSPLANTATION**

**A. Bhargava[1], N. J. Bradley[1], A. K. Burroughs[2], A. P. Dhillon[3] K. Rolles[1], B. R. Davidson[1].**
**[1]University Department of Surgery, [2]Liver Transplantation Unit and [3]Department of Histopathology**
**Royal Free Hospital School of Medicine, Pond Street, London NW3 2QG UK.**

E-Selectin, (formerly ELAM) is a cytokine inducible adhesion molecule involved in leukocyte/endothelial cell interaction. Reperfusion injury results in endothelial cell damage.

The aim of this study was to elucidate the distribution pattern and intensity of E-selectin on liver allografts after overnight cold storage and reperfusion.

Following cold storage and reperfusion (at 90 mins), liver biopsies from 23 grafts were snap-frozen. 5μm frozen sections were stained immunohistochemically for E-selectin. Intensity and distribution of E-selectin was analysed by light microscopy and by computerised image analysis (IA). Liver from resection margins of benign tumours were used as controls: demonstrating no endothelial cell staining.

Out of 23 grafts biopsied, 10 grafts were biopsied *in situ* during the harvesting procedure. 2/10 had positive staining endothelium. 14/23 grafts were biopsied following overnight storage *and* reperfusion. 10/14 had an increase in E-selectin expression following reperfusion. This was supported by IA. Mean integrated optical density for E-selectin in the stored graft was 1.07 (s,d. = 1.83) compared to 2.69 (s.d. = 2.6) following reperfusion. The difference in intensity of stain was statistically significant ($p < 0.05$ t-test). Expression was limited to large vessel endothelial cells only, with a lack of sinusoidal cell expression.

Cytokine activation of E-selectin occurs after reperfusion. Low expression seen after overnight storage may be because of a short half-life of E-selectin. Induction of E-selectin is likely to contribute to increased adhesiveness of circulating leukocytes to large vessels-blocking its expression may reduce neutrophil mediated reperfusion injury.Lack of sinusoidal cell expression suggests either an absence of certain cytokine or an inhibiting cytokine.

### F267

α-GLUTATHION-S-TRANSFERASE - A SENSITIVE MARKER OF HEPATOCELLULAR INJURY AFTER LIVER TRANSPLANTATION

K.-P. Platz, A.R. Mueller, M. Wenig, M. Postels, U. Kaisers, C. Müller, P. Neuhaus.
Department of Surgery, University Clinic Rudolf Virchow, Germany

α-Glutathion-S-transferase (α-GST) is a cytosolic enzyme of the liver with a short half life which may not only sensitively detect hepatocellular injury, but may also normalize quickly after cessation of liver damage.

α-GST was determined in 55 consecutive liver transplant recipients at pre-defined time points: prior to transplantation, prior to hepatectomy, prior to reperfusion, and 15 min, 2, 6, 12, 18, 24, 36, 48 and 72 h after reperfusion, and subsequently on a daily basis until POD 28 or until complications have been resolved. The normal range (n=40) for α-GST was 7.8±0.5 ng/ml.

In 15 patients with early allograft rejection, α-GST levels increased significantly: 3 days prior to rejection, 30.3±1.1 ng/ml; during rejection, 132.8±4.7 ng/ml ($p \leq 0.05$). After succesfull therapy, α-GST levels decreased quickly within 3 day to 31.7±2.9 ng/ml. In contrast, AST and ALT levels showed only a moderate rise during rejection which required 1-2 weeks for recovery for AST and ALT, respectively. During the first 72 h after reperfusion, α-GST levels increased with increasing reperfusion injury, but the levels were significantly higher in patient with poor graft function who subsequently developed early graft rejection compared with those patients with poor graft function with no signs of rejection ($p \leq 0.05$). A persistent rise in α-GST levels was observed in HCV-patients with recurrent graft hepatitis.

We conclude that α-GST may improve the diagnostic monitoring after liver transplantation. α-GST is not specific for graft rejection, but may have a significant impact on the decision for immunosuppressive management because it early indicates successfull rejection therapy, while AST and ALT failed to do so.

### F268

ARGININE INCREASES KUPFFER CELL TNF SECRETION IN EXPERIMENTAL BILIARY OBSTRUCTION

JA Kennedy, SJ Kirk, WDB Clements, MI Halliday, BJ Rowlands
The Department of Surgery, the Queen's University of Belfast, Ireland

**Introduction** Gram negative sepsis and endotoxaemia occur frequently in biliary obstruction obstructive jaundice. Hepatocyte/Kupffer cell co-culture experiments suggest L-arginine has deleterious effects on hepatic function in the presence of endotoxin. Using *in situ* hepatic perfusion we investigated the effects of L-arginine on Kupffer cell function in experimental obstructive jaundice, a model of chronic endotoxaemia.

**Methodology** Male Wistar rats (n=32, weight 250-300g) underwent bile duct ligation (BDL) or sham operation (S). After 21 days Kupffer cell function was assessed using *in situ* hepatic perfusion. Standard Krebs-Henseleit buffer or buffer supplemented with 1 mM/l L-arginine was used as perfusate. Livers were perfused (30ml/min) with buffer containing fluorescein labelled endotoxin (1.6 μg/ml) for 10 minutes and for a further fifty minutes with endotoxin free perfusate. Kupffer cell clearance (KCC) was determined from the fluorescence ratio of influent and effluent perfusate during the initial ten minute period. Secretory function was evaluated by assaying effluent perfusate for TNF (ELISA), IL6 (bioassay) and nitrite (Greiss reaction) after 60 minutes.

**Results** Data expressed as median (range)

| *I* | *S* | *BDL* | *S-Arginine* | *BDL-Arginine* |
|---|---|---|---|---|
| KCC (%) | 40.9(19.8-49.6) | 22.1(5.8-41)* | 40.7(19.6-51) | 20.3(9.4-32)* |
| TNF (pg/ml) | 108(49.1-182) | 937(38-1731)* | 123(45-276) | 1861(733-3822)*/ |
| IL-6 (pg/ml) | ND | 65(18-255)* | ND | 118(44-273)* |
| Nitrite | ND | ND | ND | ND |

* $p<0.05$ vs respective S, ** $p<0.05$ vs BDL Mann-Whitney *U*

**Conclusions** These results demonstrate that perfusion with L-arginine has no effect on Kupffer cell phagocytic function following BDL. Following exposure to exogenous endotoxin, hepatic secretion of TNF was enhanced by L-arginine after bile duct ligation, but not sham operation. Secretion of IL-6 was unaffected, there was no evidence of NO production. L-arginine may accentuate Kupffer cell secretory responses to experimental obstructive jaundice. TNF - tumour necrosis factor, IL6 - Interleukin 6, NO - nitric oxide, ND - not detected.

### F269

ENVIRONMENT AND MORBIDITY: CAUSE AND EFFECT ?

A. Georgouli [1], L.B. Georgoulis [2]
[1] Athens Medical Centre, Greece, [2] Dept. of Environmental Health, University of Washington, Seattle, WA, U.S.A.

The purpose of this study is to show that in addition to the emphasis placed by the medical community on the early diagnosis and treatment of disease, the existing possibilities for prevention should also be stressed.

A thorough literature search was conducted to find early references on the causes of the so called diseases of civilization (cardiovascular and pulmonary diseases, cancer etc.). In addition, the titles of more than 6500 abstracts in 28 international medical congresses covering the entire spectrum of morbidity were studied. Finally the bulletins from five medical schools in Greece, Switzerland and the United States were analyzed to estimate the time devoted in disease prevention as opposed to diagnosis and treatment.

There are references in the literature dating back to at least 1661, showing the awareness of the scientific community that the pollution of the environment can increase the prevalence of disease causing factors. The results of the abstract title search showed that only 3% of them were actually related to disease prevention. Finally, in the educational programmes of the medical schools studied approximately 15% of the time is devoted to subjects related to disease prevention.

In conclusion, it appears that the notion of disease prevention in medical practice and education is of secondary importance. Moreover, we continue to focus on the effects of high doses of disease causing factors on specific population groups and ignore the fact that the prolonged exposure of the entire population to much smaller doses can be equally or even more harmful. Finally, in addition to the efforts aimed towards the decrease of mortality, emphasis should be placed on the decrease of morbidity as well.

### F270

THE NATURAL HISTORY OF GALLBLADDER DISEASE IN MORBIDLY OBESE PATIENTS

S.Papavramidis, I.Kessissoglou, I.Dokmetzioglou, N.Koulouris, A.P.Aidonopoulos
3rd Dept. of Surgery, A.H.E.P.A. Hospital, Aristotelian University of Thessaloniki

A total of 120 consecutive morbidly obese patients - 29 male and 91 female - who treated by vertical gastroplasty between January 1991 and December 1994 were studied prospectively to determine the gallbladder disease in this group of patients. The mean age was 35,47±8,66 years (range 20-62), the mean percentage excess body weight 125,26±36,55 (range 80-280) and the mean BMI 52,66±9,61 kgr/m$^2$ (range 42-94). Ten patients (8,3%) had undergone cholecystectomy before bariatric surgery because of symptomatic cholelithiasis and the remaining 110 patients underwent cholecystectomy at the time of vertical gastroplasty. Ninety seven percent of the removed gallbladders had gross or histologic abnormalities including cholelithiasis 22,5% (27 patients), cholesterolosis 21,6% (26 patients), sludge 10,8% (13 patients), and chronic cholecystitis alone 41,6% (50 patients). Histologically, chronic cholecystitis was present in all patients with cholelithiasis, cholesterolosis and sludge. Only 4 patients (3,3%) of this series had normal gallbladder. The percentage excess body weight of the patients with cholelithiasis (137,2±38,6%), cholesterolosis (133,4±47,9%), and sludge (134±28,9%) was significantly greater than that of patients with chronic cholecystitis (112,08±25,8%) (p=0,0009, p=0,009, and p=0,01 respectively). The BMI of the patients with cholelithiasis (55,3±9,4 kgr/m$^2$), and sludge (55±9,4 kgr/m$^2$) was also significantly greater than that of patients with chronic cholecystitis (50,24±7,1 kgr/m$^2$) (p=0,005 and p=0,02 respectively).
It is concluded that cholecystectomy should be performed in all morbidly obese patients concomitant with vertical gastroplasty.

### F271

DYSPHAGIA FOLLOWING LAPAROSCOPIC ANTI-REFLUX PROCEDURES. IS SEX A DETERMINING FACTOR?

John K. Edoga, M.D.
Department of Surgery, Columbia University College of Physicians and Surgeons, Morristown Memorial Hospital, 100 Madison Ave., Morristown, NJ 07960

Seventy-nine patients (37 males, 42 females) underwent laparoscopic anti-reflux procedures at Morristown Memorial Hospital over a 24-month period. Sixty-seven patients had Nissen fundoplication. Twelve underwent the toupet procedure.

Dysphagia developed postoperatively in 8 patients. Seven of these 8 patients were female.

Four of the 7 females required one dilatation session for complete relief of symptoms. Two had 2 or more dilatations. One patient refused dilatation. Her swallowing gradually improved over a 12-week period.

The only male in the dysphagia group needed one bougie treatment.

In our series, sex appears to be the only statistically significant distinguishing factor among the patients who developed dysphagia after laparoscopic anti-reflux procedures.

### F272

PIG ISLET ISOLATION AND ALLOTRANSPLANTATION

E. Andereggen, S. Deng, L. Bühler, R. Mage, C. Bubloz, Ph. Morel
Transplant Unit, Department of Surgery, University Hospital, Geneva, Switzerland

Porcine pancreases are considered as a suitable source of islets for future use in xenotransplantation, because of the similarity between human and porcine insulin. However, mass isolation of porcine islets remains a real challenge, because of the important fragility of pig islets. Transplantation in a large animal model should provide an adequate support to better assess the feasibility of pig islet isolation and transplantation. Method: Islets were isolated from pancreases of slaughterhouse pigs (age 5-8 month), using a modified automated method. From one pancreas we obtained a mean ± SD of 115'000 ± 57'107 purified islets. The islets were transplanted by intraportal embolisation into the liver of surgically pancreatectomized pigs. Each recipient was transplanted with islets pooled from 3 donors, which represents a number of purified islets ranging from 205'000 to 667'000 (6'949 to 29'000 islets/kg body weight of the recipient). Study groups: In *group 1 (N=6)*, the recipients did not receive any immunosuppression. In *group 2 (N=4)* an immunosuppressive treatment of CsA (6 mg/kg/d) and Azathioprine (5 mg/kg/d) was administred by intra-venous (N=3) or intra-muscular (N=1) route, until rejection. The daily monitoring consisted of plasma glucose and insulin measurements. Liver biopsies were performed after recurrence of hyperglycemia and analysed for histology and immunohistological staining for insulin. Results: *Group 1:* Insulin secretion was observed for a mean of 4.8 days (range 2 to 11 days) after transplantation. Histological examination of the liver biopsies showed obvious signs of rejection with lymphocyte infiltration of the islets, but immunohistological staining for insulin was still positive in liver biopsies performed on days 4 and 8. *Group 2:* Three recipients, although presenting a slightly increased glycemia, sustained an insulin secretion for 6 to 9 days. At the time of sacrifice, also in this group, liver biopsies showed an important lymphocyte infiltration of the portal spaces with no visible defined islets. Conclusion: We were able to isolate an amount of viable pig islets sufficient to sustain, after intra-portal allotransplantation in pancreatectomized pigs, insulin secretion for several days. However, these results required the pooling of several islet preparations, still emphasizing the difficulties encountered in pig islet isolation. In this transplant model, rejection may represent one factor for early graft failure. Islet allotransplantation in pigs can be considered as a suitable model to assess the results of mass islet isolation, providing that the immunosuppressive regimen will be improved. Other experiments are actually in progress, especially using older pigs as donors, which provide in our experience best islet preparations in terms of yield and quality.

### F273

### COMBINED LIVER AND PANCREAS TRANSPLANTATION.

E.J.Hadjiyannakis, S.Iakoumenta, S.Drakopoulos, E.Plessia, N.Nikitakis, S.Mathioulakis, N.Georgopoulos, A.Poultsidi, S.Raptis.

1st Surgical Department and Transplant Unit - "Evagelismos" Hospital of Athens.

### F274

### HETEROTOPIC PANCREATIC TISSUE IN THE GALLBLADDER. A RARE ENTITY

T. Kotsis, G. Polymeneas, A. Paphiti, A. Antoniou, J. Papadimitriou
Second Department of Surgery, University of Athens Medical School, Athens, Greece

Heterotopic pancreas is a congenital anomaly frequently encountered by surgeons operating in the abdomen. The most common locations in the different series were the stomach, duodenum and the jejunum. Pancreatic heterotopia in the gallbladder is extremely rare.
Three cases with histologically proved heterotopic pancreatic tissue are presented. The patients had diversified clinical manifestations with predominant symptom epigastric discomfort, and after preoperative evaluation which in the two of them revealed lithiasis and polyps underwent cholocystectomy, two of them laparoscopically.
The reported cases of ectopic pancreas in the gallbladder are less than twenty in the world literature. The preoperative diagnosis is difficult and as most of the heterotopic tissues, the ectopic pancreas in the gallbladder tend to be an incidental finding during surgery and definitively detected by the pathologist.

### F275

### THE COMPARISON OF CEFAMANDOL VERSUS AMOXYCILLIN CLAVULANIC ACID FOR SINGLE DOSE PROPHYLAXIS IN ELECTIVE SURGERY OF THE UPPER ABDOMINAL REGION

P.Marosvölgyi,Gy.Ugocsai,K.Kreisz,A.Sárospataki, E.Harasta,S.Bellovicz

At 1st April 1993. - based on the good results with cefamandol (Mandokef)for single dose preoperative prophylaxis - a prospective randomized study was started in order to make comparison between the effect of cefamandol versus amocy - cillin-clavulanic acid (Augmentin) for prophylaxisof the elective surgery of the upper abdominal region.
1o8 patients were randomized into two groups. Till 1st November 1994. 51 patients were administered iv. 1 g of cefamandol, and 53 patients iv. 1.2 g of amoxycillin-clavulanic acid on the induction of the anaesthesia. Four patients were withdrawn.The types of surgical procedures,the types of wound healing, risk factors,skills of operating surgeons and the length of hospital treatment were registrated. Statistical analysis of the data was carriedout using standard procedures by BMDP.
Two wound infections were observed: one in the cefamandol-group,the other after the use of amoxycillin-clavulanic acid.The results - wound infection about 2% - seemed to be in correlation with other studies.The effect of 1 g cefamandol was comparable to that of 1.2 g amoxy - cillin-clavulanic acid for single dose prophylaxis in the elective surgery of the upper abdominal region.The difference between these medicaments has to be defined by economical calculations.

### F276

### EVALUATION OF VIABILITY OF PANCREATIC GRAFT FOR TRANSPLANTATION

E. Brazda, L. Flautner, T. Donáth*, L. Harsányi
1st Dept. of Surgery, *1st Dept. of Anatomy and Histology, Semmelweis University of Medicine, Budapest, Hungary

Introduction
Pancreas is very sensitive to intraoperative handling. Macroscopically the condition of the harvested organ remains questionable in some cases and the pancreas should be discarded. A rapid and reliable procedure to determine the viability of the removed pancreas could decrease the number of the discarded organs. For this reason a new method based on acridine-orange uptake was developed in a pig model to study the effect of ischaemia on the integrity of the cell membranes in the pancreas.

Methods
In twelve pigs weighting about 30 kg abdominal cavity was explored in intratracheal isoflurane narcosis. After removal and flush-out of body and tail of pancreas a) 10 to 60 minutes of warm ischaemia or b) 2 to 24 hours of cold ischaemia in Euro-Collins (EC) perfusion solution or c) 2 to 24 hours of cold ischaemia in Belzer (UW) perfusion solution was applied. Then a small piece of the organ was placed in 0,2‰ acridine-orange solution for 5 minutes. Thick sections (10μm) made by rapid frozen technique were examined by fluorescence microscope. The extent of membrane lesions was estimated by 1) intensity of fluorescence, 2) rate of the fluorescence area and 3) morphology of the cells.

Results

| Type of ischaemia | Slight damage (yellow color) | Serious lesions (orange color) | Disintegration (no cell structure) |
|---|---|---|---|
| Warm (n=8) | 20 min. | 30 min. | 40 min. |
| Cold, EC (n=8) | 3 hours | 4 hours | 6 hours |
| Cold, UW (n=8) | 6 hours | 21 hours | 24 hours |

Conclusion
After adaptation into human model this method could be useful in the determination of viability of removed human pancreas. Our data also confirm the importance of the shortening of warm ischaemia time and the advantage of the use of Belzer-UW solution in the cold storage of the pancreatic grafts.

### F277

REDUCE OF POSTOPERATIVE DYSPEPSIA BY THE USE OF HIGH DOSES OF PANCREATIC ENZYMES (PANZYTRAT)

L. Papastamatiou, G. Tzagarakis, D. Xypolytas, D. Antonopoulos
2nd Dept. of Surgery "Apostle Paul" Hosp-KAT. ATHENS-HELLAS

It is well known that per os administration of pancreatic enzymes ameliorates digestive discomfort. There is a number of patients, with atypical upper abdominal symptomatology, connected or not with pancreatic disfunction, following biliary or pancreatic surgery.

This study deals with administration of high doses of pancreatic enzymes (Panzytrat) in 163 patients during the second semester of 1994. The clinical material is devided in 4 groups:

G1: 100 cases of elderly and/or overweight cholecystomized patients.

G2: 30 cases of eventual pancreatic reaction due to Oddi area manoeuvres.

G3: 25 cases of acute inflammatory pancreatic reaction following acute cholecystitis.

G4: 8 cases of post-traumatic or not acute pancreatitis.

All patients received postoperatively Panzytrat (1-2 drg x 3) for 15 days (G1 and 2), 30 days (G3), 90-180 days (G4).

The results seem to be satisfactory: Only 5 patients of G1, two of G2 and two of G3 complained for symptoms similar to those who did not receive Panzytrat (88 patients out of 127 of the first semester 1994). Follow-up of G4 is still in process.

It is concluded that high doses of pancreatic enzymes reduce postoperative digestive symptomatology in 92-95% of patients, who undergone surgery on extrahepatic biliary tract, especially when connected with pancreatic inflammatory reaction.

It is suggested that following pancreatic surgery (resection, transplantation) Panzytrat administration may be of great assistance in normal digestion.

### F278

The use of the Somatostatine Receptor Scintigraphy (Octreoscan) for differentiation between endocrine and exocrine tumours.

CHJ van Eijck, FT Bosman, L Lemaire, HA Bruining, J Jeekel, EP Krenning, SWJ Lamberts

Somatostatine receptors (SS-R's) are present on most of the island cell tumours of the pancreas, while recently *in vivo* studies demonstrated the absence of these receptors on adenocarcinomas of the pancreas. In this study the value of the *in vivo* localisation with the Octreoscan has been evaluated in 25 patients with an endocrine pancreas tumour and in 25 patients with an adenocarcinoma. In all these patients an Octreoscan was performed pre-operatively when the presence of one of these tumours was expected. In 80% of these cases it was possible to visualize the endocrine tumour; adenocarcinomas of the pancreas could not be demonstrated in any case. The corresponding *in vitro* autoradiography demonstrated that actual SS-R's were visualized. Preoperative differentiation between endocrine and exocrine tumours of the pancreas is important since palliative surgery in patients with an endocrine tumour may not only reduce the clinical symptoms but also because a diminished tumour load enlarges the effect of the medical treatment.
This study indicates that the Octreoscan can be used in preoperative diagnostics of patients with a pancreas tumour of unknown origin to differentiate between an endocrine and exocrine tumour of the pancreas.

### F279

LONG TERM RESULTS OF PYLORIC PRESERVING PANCREATODUODENECTOMY FOR CHRONIC PANCREATITIS (PPPD). R. Martin, R.L. Rossi, K.A. Leslie, J. Buyske. Department of General Surgery, Lahey Clinic, Burlington, MA, USA

Forty five patients that had a PPPD for disabling chronic pancreatitis were reviewed to assess their long term pain improvement and metabolic and nutritional changes. The median follow up was 63 months. The mean preoperative duration of pain was 50 months with 70% of the patients using narcotic daily. PPPD was performed in all cases with 1 having the portal vein replaced by the internal jugular vein. Mean duration of NG suction was 9 days. One patient (2.2%) died within 30 days of the PPPD. 92% of the patients had pain improvement by 5 years. The pain score (0-10) was 9.2 preoperatively and 1.5, 0.8, 1.1 and 1.1 at 6 months, 1 year, 2 year and 5 years respectively. Compared to pre-illness weight, patients lost an average of 16% of their weight prior to PPPD. Following PPPD, patients gained an average of 8% of their pre-illness weight (50% of the weight loss) with 70% of patients increasing their preoperative weight. Diabetes of new onset occurred in 46% of the cases by 5 years. Diabetic complications (hypoglycemia) was the cause of late death in only 1 patient who had a total pancreatectomy. Marginal ulcers developed in 10% of the cases.
In selected patients, resection of the head of the pancreas achieves pain improvement in over 90% of the patients with metabolic complications comparable to other partial resective procedures. Weight gain was superior to our patients with standard pancreatoduodenectomy for chronic pancreatitis and PPPD for malignancies.

### F280

**PATHOLOGY OF CHRONIC IDIOPATHIC DUCT-DESTRUCTIVE PANCREATITIS.**

**B. Maillet, N. Ectors**, K. Geboes**, A. Donner***, F. Borchard***, M. Stolte**** and G. Klöppel*.**

Departments of Pathology, University of Antwerp; Academic Hospital Jette*, Free University of Brussels and UZ-Sint Rafael**, Catholic University Leuven, Belgium.
Institutes of Pathology, City of Bayreuth**** and University of Düsseldorf***, Germany.

In the industrialized countries, chronic pancreatitis is usually associated with alcohol abuse. Other forms such as familial CP, hypercalcemia-associated CP and idiopathic CP are rare.

We recently observed seven patients presenting with an idiopathic CP. The seven patients, four women (17, 19, 24 and 67 years) and three men (29, 59 and 59 years), had the typical symptoms of chronic pancreatitis. One patient had in addition an autoimmune sialadenitis. None of the patients had idiopathic bowel disease or autoimmune liver disease The resection specimens showed dense inflammatory infiltrates surrounding the small to medium sized-ducts. The infiltrates consisted of (mainly B-) lymphocytes, plasma cells, macrophages (some S-100 positive) and occasionally also eosinophils and neutrophils. The inflammatory process led to the destruction of the duct epithelium and initiated periductal fibrosis. These duct obliterating changes caused fibrosis of the acinar tissue upstream of the stenosis. None of the specimens contained calcified protein plugs (calculi) characterizing advanced alcohol-induced chronic pancreatitis.

We conclude that there is a non-alcoholic chronic pancreatitis characterized by primary duct destruction, possibly due to an autodestructive process directed against the duct epithelium.

### F281

PATHOGENETIC OPERATION IN CHRONIC PANCREATITIS

G.Ratner, E.Korymasov, Yu.Gorbunov

Clinic of Faculty Surgery, Samara State Medical University, Samara, Russia

Gastric and pancreatic secretion has common mechanisms of nervous and hormonal regulation. Therefore, we carried out in chronic pancreatitis almost the same treatment as in peptic ulcer. We studied the late results in 80 patients after truncal vagotomy with antrumresection /1 group/ and in 46 patients after truncal vagotomy with pyloroplasty/2 group/, which was carried out in chronic pancreatitis. Clinical, endoscopical, roentgenological and sonographical methods were used.
Excellent result means absence of pain and necessary diet. Good result means pain in diet breach only. Satisfactory result means decrease of frequency and intensity of pain attacks.
In 1 group the late results were excellent in 68,8%, good - in 13,7%, satisfactory-in 13,7%, unsatisfactory-in 3,8%. In 2 group respective results were in 21,7%, 30,4%, 28,3%, 19,6%.
The best results in 1 group were explained by that the main role belongs not to parasympathetic innervation, but to hormonal factors in chronic pancreatitis. The switch of duodenum decreases secretin and pancreozimin stimulation. We make a conclusion that the truncal vagotomy with Hofmeister-Finsterer antrumresection is pathogenetic operation in chronic pancreatitis, because it effects on all three phases of pancreatic secretion.

### F282

GASTRIC ACIDITY FOLLOWING LONGITUDINAL PANCREATICOGASTROSTOMY

I. Kovács, P. Árkosy, Gy. Dauda, Mrs. Mahunka, P. Sápy

2nd Department of Surgery and Laboratory Informatics, University Medical School of Debrecen

34 patients were investigated who were operated for chronic pancreatitis. A longitudinal pancreaticogastrostomy was applied for relief of symptoms in all cases.
The question was, wether the pancreatic juice released into the stomach have any effect on gastric acidity.
A 24 hour gastric pH monitoring was performed on every patient before and 6 weeks after the operation.
Following a complete postoperative check-up we found that 33% of the patients had no digestive problems with pancreatic enzym substitution. The average postoperative weightgain was 3.8 kg. The number of diabetic patient increased from 9 to 12.
According to our statistical evaluations of 24 hour gastric pH monitoring test no alternation was detected in gastric pH level postoperatively.
We have found that the pancreatic pressure which has been the main cause for pain can be relieved by pancreaticogastrostomy.
On the bases of our result we can consider the pancreatico gastrostomy as an operation choise to relieve intractable pain in most patients with chronic pancreatitis associated with duct dilatation.

### F283

### Intraductal and Parenchymatous Pancreatic Pressures Do not Correlate with Pain in Chronic Pancreatitis

JC Limmer, WT Knoefel, E Achilles, C Blöchle, JR Izbicki
Department of Surgery, University of Hamburg, Hamburg, Germany

**Introduction:** Therapeutic options to alleviate pain in chronic pancreatitis are based on drainage or resection. No established rationale exists for either approach. We therefore investigated the relation between pain and pancreatic pressure.

**Methods:** 20 consecutive patients that underwent pancreatic surgery for chronic pancreatitis with intractable pain were assessed by a previously validated pain score before surgery. Parenchymatous pressures in the head, body, and uncinate process, as well as intraductal pressures were measured during surgery using the needle puncture technique. All patients underwent duodenum preserving resection of the pancreas.

**Results:** Pancreatic pressure was elevated significantly in all patients. However, none of the measured pressures correlated with the individual severity of pain. Median pressure was 30, 24, and 33 mm Hg in the head, body, and uncinate process, respectively. Median ductal pressure was 30 mm Hg. The coefficient for the correlation between pressure and pain score was r=0.039, r=0,41, r=0.11, and r=0.14 for the head, body, uncinate process, and duct, respectively (n.s.).

**Conclusions:** We conclude that parenchymatous pressures, reflecting small ductular pressures, as well main duct pressures are elevated in painful chronic pancreatitis. The severity of pain, however, is not directly linked to the pressure. Pain in chronic pancreatitis, therefore has parenchymatous components. This is the rationale for combining resection and drainage.

### F284

### CHRONIC PANCREATITIS AS A CAUSE OF SEVERE RECURRENT GASTROINTESTINAL HEMORRHAGE - HAEMOWIRSUNGIA

Z. Gerzić, T. Randelović, Z. Janković, A. Simić, M. Pavlov

Institute for Digestive Diseases, First Surgical Clinic, Clinical Center of Serbia, Belgrade, YUGOSLAVIA

Chronic pancreatitis with a communication between the main pancreatic duct and aneurysm of the splenic artery followed by massive and recurrent gastrointestinal bleeding is a rare condition but extremely difficult to diagnose.
Authors describe a 31 year old male in whom recurrent gastrointestinal bleeding was found to have chronic fibrous pancreatitis and communication of the splenic artery aneurysm to the main pancreatic duct. The patient required several admissions and diagnostic evaluations in another hospital and there he underwent unnecessary surgery resection of the distal esophagus followed by esophago-gastric anastomosis.
After admission to our hospital because of a severe epigastric pain and melena, clinical evaluation reveled peptic esophagitis with partial incomplete supraanastomotic stricture of the distal esophagus. Peptic stricture of the esophagus was resected with the interposition of the pedunculated jejunal segment according to Merendino but the bleeding did not stop. The possibility of pancreatic source of bleeding was then entertained. CAT scan with oral contrast showed a low density mass in the pancreatic tail and dilated main pancreatic duct. A celiac angiogram reveled a small aneurysm of the distal portion of the splenic artery.
Distal pancreatectomy with splenectomy has been performed with uneventful postoperative course. The operative specimen showed direct communication between aneurismal sac (13 mm) and dilated main pancreatic duct obstructed by clots. Pathologic changes of chronic sclerotic pancreatitis were also found. On the control after two years, the patient had no episodes of gastrointestinal bleeding and he had no complaints.
The author will present the revue of literature and point the problem of diagnostic difficulties in a such case. Also, we have to emphasize the significance of chronic pancreatitis as a cause of this rare condition.

### F285

PANCREATIC TRAUMA - EXPERIENCE OF A 50 CASES (1984-1993)

G.Ionescu,M.Ciurel,A.Bucur,M.Beuran,S.Jianu,S.Păun
Clinic of Surgery,Emergency Hospital,Bucharest,Romania

The purpose of this study is to evaluate the frequency and the gravity of the pancreatic trauma in correlation with visceral associate injuries,surgical interventions types and measures for decrease the morbidity and mortality of these lesions.Using a retrospective analysis,50 patients were examined concerning the etiology,the diagnosis,the lesion's degrees,the associate injuries,the types of the performed operations,the morbidity and the mortality.The final diagnosis was established only intraoperative while the lesion's degree alternated between 52% in lesions of first and second degrees to 38% in third degree and only 10% in fourth degree.Surgical interventions were represented by drainage in almost 50% of cases,pancreatic suture in 15% cases,caudal pancreatectomy in 12% cases and others,including surgical interventions for associate injuries.General mortality was of 42% (21 cases) while 14 patients were died in 24 hours by complex traumatic and haemorrhagic shock.
Concluding,the pancreatic trauma have a small frequency but high gravity,often associated with multiple visceral injuries;the preoperative diagnosis is only probably,the final one is just intraoperative;it is absolutely necessary the lesion degree's evaluation;the surgical interventions must be addapted to the associated visceral injuries,priority for hemostasis and protection against infection and,after that,the therapy for pancreatic lesion in according with minimal intervention;very important for decrease the mortality are the complex measures of intensive care medecine,quick and competent applied.

### F286

REMARKS ON THE MANAGEMENT OF TRAUMA OF PANGREAS AND SPLEEN

Sp. Milingos, Mar. Paraskevopoulou, D. Michaelidis, D. Panagoulis, D. Tsambrinou, V. Kollia
"Evangelismos" General Hospital and "Therap. Kypselis" Clinic

Adhesive substances have been used in many fields of surgery either experimentally or clinically. In the present work an effort was made to estimate the utility of N. Butyl-C-M in the management of trauma of the spleen and pancreas.

Five patients with trauma of the spleen and seven with trauma of the pancreas after biopsy were managed by finger pressure and application of N. Butyl-2-C-M. The patients were followed postoperatively for bleeding and pancreatitis one year later in two of our patients a second look operation was performed and biopsies were taken.

Our results indicate that N. Butyl-2-C-M can be used as an adhesive substance because the postoperative course in our patients was univentful. Bleeding or peritonitis did not occur and second look exploration when performed did not show excessive fibrous reaction and adhesion formation. Healing of the spleen and pancreas was satisfactory and histological examination when performed showed no carcinomatous reaction.

Our results indicate that this substance has high adhesive properties and above all its use is not followed by complications of excessive reaction and we can use it in the management of spleen and pancreatic trauma.

### F287

COMBINED PANCREAS INJURY

M.Pavlovsky, A.Perejaslov, S.Chooklin
Department of Surgery, Lviv Medical Institute, Ukraine

10 patients with acute posttraumatic pancreatitis were observed. 8 of them, had the pancreas damage, which in 6 cases associated with the splenic injury and in 2 cases - with hepatic and splenic damage. 6 patients had pancreas contusion and 2 - superficial trauma of the gland without disruption Wirsung's duct. In 2 patients with multiple damages of organs of abdominal cavity acute pancreatitis developed without macroscopic changes in pancreas. All the patients were hospitalized with clinical symptoms of the different severity of traumatic shock. On this background the symptoms of pancreas damage were not obvious. Laboratory, roentgenological investigations and ultrasonography were used for the diagnosis.
Hyperamylasemia was not pathognomonic symptom of the pancreas damage. Enhanced amylolytic blood activity was observed in damage of the stomach, duodenum and craniocerebral trauma. The severe course of acute pancreatitis, including posttraumatic, is accompanied by depression of the suprarenal cortex function. The leading role in the diagnosis of combined pancreas injuries belonged to ultrasonography. In the focal necrotizing changes of pancreas, in the inflammatory process involved the whole gland, what determined noneffectivness of its resection.
All patients were operated. Taking into consideration the severe accompanying pathology surgical intervention were restricted the adequate drainage of bursa omentalis and retroperitoneal space, with the following infusion of the enzyme inhibitors, cytostatics and antiseptic solutions. External pancreatic fistula formed in the postoperative period in 7 patients. In 5 cases fistula closed spontaneously, in 2 cases the operation was obligatory. Pseudocyst formation was the outcome of the posttraumatic pancreatitis in 3 patients. Ultrasonic guided drainage was used for the treatment of pancreatic pseudocysts. Our experience with the pseudocysts, including posttraumatic ones, showed that the ultrasonic guided drainage was effective in 60% cases.

### F288

TUMOUR NECROSIS FACTOR (TNF) AND SOLUBLE TNF RECEPTORS (TNF-sR) IN SEVERE LIVER DISEASE AND TRANSPLANTATION

P.E. Majno, P. Roux Lombard, J.M. Dayer, P. Morel, G. Mentha
Department of Digestive Surgery and Immunology, University of Geneva, Switzerland

TNF is a cytokine with an important role in the acute phase response and in the process of graft rejection and primary graft non function. The soluble forms of its cellular receptors, known as TNF-sR55 and TNF-sR75, act as "natural circulating inhibitors", potentially able to neutralise its effects. To gain further insight on the biology of TNF and its receptors in liver disease and transplantation we studied the levels of TNF and TNF-sR in a group of 30 patients with end stage cirrhosis before and after transplantation, and in 57 normal volunteers. TNF and TNF-sR were measured with radio- and bio- immuno assays respectively.

While TNF levels were normal (<40 pg/ml) before and after transplantation (32±9 and 28±5), TNF-sR levels were increased before transplantation (TNF-sR55: 5.4±3, and TNF-sR75: 12.3±5.1ng/ml, p<0.001) and returned towards normal once stable graft function had been achieved (TNF-sR55: 2.9±1.3, controls 2.0±1.0; TNF-sR75: 6.2±3.1, controls: 3.5±1.2). TNF-sR were markedly increased during complications such as rejection, primary non-function and ischaemic necrosis of the graft, with no correlation to TNF values, nor to other indeces of inflammation and hepatic function.

TNF-sR behave as independent markers of liver disease, and are present at levels able to neutralise the systemic effects of TNF. This may explain why patients with cirrhosis are often unable to express the signs of the inflammatory response even in the presence of infection. Increased levels of TNF-sR during graft rejection and primary graft non function, independently of TNF levels, do not lend support to the theory that these conditions are due to an imbalance between TNF and its natural inhibitors, nor that increased TNF-sR levels are a mere reflection of TNF activity on target cells. The role of TNF,and the modulating action of TNF-sR appear to be important in the pathophysiology of liver disease, and of relevant clinical events after liver transplantation.

### F289

DYNAMICS OF PLASMA CHOLESTEROL (CHOL) CHANGES AFTER HEPATIC RESECTION.

I. Giovannini, G. Nuzzo, G. Boldrini, F. Giuliante, C. Chiarla.
Dept of Surgery (Chirurgia Geriatrica), Catholic University School of Medicine, Rome, Italy

The evolution of CHOL has been assessed, together with the correlation with other variables, in 130 measurements performed in 40 patients after liver resection. Mean CHOL was 59% of basal (BASCHOL) in the first postop day, 49% in the 2$^{nd}$, 56% in the 3$^{rd}$, 57% in the 4$^{th}$, 64% in the 5$^{th}$. Prompt and uneventful recovery was associated with a rapid reversal of hypocholesterolemia. Slow recovery (with liver failure or sepsis) was associated with persistent hypocholesterolemia. Death was characterized by rapid and profound fall in CHOL. A "Cholesterol Renormalization Index - CRI" was developed to allow a separation of the three postoperative patterns (rapid or slow recovery, and death) by comparison with actual CHOL:

$$CRI = \log(1.03 \cdot CHOL \cdot BASCOL^{-0.91} \cdot POSTOP.DAY^{-0.08})$$

Changes in CHOL were directly correlated to indices of adequacy of hepatic protein sinthesis (TAP, colinesterase), to hematocrit (total $r^2 = 0.5$, $p \ll 0.01$) and, in cholestasis, also to alkaline phosphatase (dCHOL/dALP ≈ 0.07 mg/dl per UI/L; nv of ALP= 98-279). These data show that the simple measurement of CHOL may provide useful information on the severity of illness and on the adequacy of recovery of hepatic function after resection.

### F290

EXPERIMENTAL ALLO-TRANSPLANTATION OF PIG ISLETS IN CASES OF TOXIC INDUCED DIABETES. IMMUNOSUPPRESSION AND ENDOCRINE FUNCTION

A. Nikolaou[1], A. Papalois[1,3], K. Nikolaou[1], B. Papalois[1,4], Ch. Tountas[2], B. Karamanos[2], G. Bonatsos[1], B.Golematis[1]
[1]1st Department of Propaedeutic Surgery, [2]2nd Department of Medicine, [3]Department of Biology, University of Athens, Athens, Greece and [4]Department of Surgery, University of Minnesota, U.S.A.

A topic of current interest in experimental islet transplantation (Tx) is the selection of an optimal immunosuppressive treatment with long-term graft function. The aim of this study was to evaluate the efficacy of two simple immunosuppressive treatments (IT) in islet post-Tx function. Totally 24 female swine (18-21 Kg) were used as donors (12) and recipients (12) of islets. For islet isolation total pancreatectomy was performed and the collagenase (Type XI, Sigma C-7657/1 mg/ml) digestion technique of the University of Minnesota was used with modifications. Recipients were divided in two groups: Group A (n=6): Toxic induction of diabetes followed 1 week after by intraportal islet Tx. IT: Cyclosporin A (CyA) 5 mg/Kg iv before surgery and the 1st post-operative day and 15 mg/Kg per os days 2-30. Azathioprine (Aza) 5 mg/Kg iv 1st day post-operative and 2 mg/Kg per os days 2-30. Group B (n=6): same as in Group A. In IT the protocol was included also Prednizolone (Pr) 2 mg/Kg iv 1st day post-operative and 2 mg/Kg per os days 2-30. Diabetes in all animals was induced using Streptozotocin (STZ-Sigma S-0130). Total dose 65 mg/Kg iv. Intravenous glucose tolerance test (IVGTT) was performed before Tx (before STZ and after induction of diabetes) and in days 14 and 30 post-operative. All animals were survived for 30 days (euthanasia). Functioning grafts for 30 days were 4/6 in Group A and 5/6 in Group B having a range of islets >20,000->80,000 and purity 70-90%. The biochemical data showed more satisfactory function of the grafts in Group B in the early post-operative period compared to Group A. We conclude that triple immunosuppressive therapy support graft function especially in the early post-operative period (0-14 days).

### F291

LIVER CYTOKINE ACTIVATION IN PARTIAL HEPATECTOMY

J. Schröder[1], H. Gallati[2], B. Kremer[1]
Dept. of General Surgery, University of Kiel, Arnold-Heller-Str. 7, 24105 KIEL, Germany[1]
Hoffmann La Roche Inc., 4002 Basel, Switzerland[2]

The liver is known to be an important site of tumor necrosis factor alpha (TNF) and interleukin 6 (IL 6) production. Little is known about local changes in cytokines during and after partial hepatectomy.

TNF levels, using an immunoassay, concentrations of soluble TNF receptors (sTNFR) I and II as naturally occuring TNF antagonists, using an enzyme-linked immunological binding assay and IL 6 bioactivity were measured. Serum levels were determined intravenously before and after operation, before ischemia (Pringle maneuver) and in the reperfusion period in portal and liver vein as well as systemically. In 12 patients predominently with metastasis (9 patients) the mean time for the Pringle maneuver was 33 minutes. Maximum levels of sTNFR I, II (4,6 vs 7,0 ng/ml) and IL 6 (180 pg/ml) were measured on day 2 postoperative and decreased up to day 5. Maximum levels are significantly elevated compared to baseline levels ($p < 0{,}01$). No TNF immunoreactivity could be detected in the circulation. No differences for soluble receptors and IL 6 could be observed between portal and liver vein concentrations before ischemia and in the reperfusion period. A positive correlation was found between levels of IL 6 and sTNFRI ($r = 0{,}4$; $p < 0.001$; $n = 139$), between IL 6 and sTNFRII ($r = 0{,}53$; $p < 0.001$) and between both receptors ($r = 0.6$; $p < 0.001$).

No release of TNF into the circulation and no further increase of sTNFR I and II as well as IL 6 could be induced by liver ischemia and reperfusion. Increasing levels of soluble TNF receptors could be detected throughout the evaluation and may represent the TNF activity during and after partial hepatectomy.

### F292

NEW HEMOCORRECTOR OF COMPLEX ACTION "LACTOSORBAL" IN IRREVERSIBLE HEMORRHAGIC SHOCK (IHS) IN DOGS: EFFECTS ON LIVER FUNCTIONS (LF)

A.N.Oborin
Department of Surgery and Transfusiology, Research Institute of Hematology, Lvov, Ukraine

The study was carried out in 5 dogs in which IHS was induced by momentary jet hemorrhage from a. femoralis (the blood loss volume made 30.1±1.3 ml/kg). LF were estimated by the v. cava caudalis blood contents of common bilirubin (CB), aspartate- and alaninaminotransferase (AsAT, AlAT), acid and alkaline phosphotates (AcPh, AlPh), pseudocholinesterase (PChE) and oligopeptides of middle mass (OPMM). The treatment was begun after 7.5±0.7 hrs of the hemorrhage with arterial blood pressure level of 30 mm Hg. "LACTOSORBAL" which is a 5% albumin solution containing 20% sorbit solution, sodium lactate, $NaHCO_3$, $K^+$, $Na^+$, $Ca^{2+}$ was injected into v. femoralis at the dose of 10.0 ml/kg. It is established that before treatment contents of CB, AsAT, AlAT, AcPh, AlPh and OPMM increased by 65.2%, 209.4%, 119%, 45%, 72.3% and 35.3% ($P<0.001$), while PChE diminished by 3.9% ($P<0.01$), correspondently. The "LACTOSORBAL" transfusions were accompanied by the quick and steady normalization of cardio-hemodynamic functions and breathing. For all this the contents of OPMM in the blood did not statistically differ from initial level on the 3d day of observation, from PChE on the 4th day, from CB on the 5th day, from AcPh on the 6th day, and from AsAT, AlAT, and AlPh on the 7th day. All dogs survived.

The results of present study are experimental ground for use "LACTOSORBAL" in complex therapy of acute liver failure in clinic.

### F293

IN VIVO COMPARATIVE STUDY OF FOUR SCOLICIDAL AGENTS AGAINST E. granulosus

Emm. Kriticos, Per. Tzardis, A. Babionitakis,
Th. Cordosis, D. Vouyouklakis, Emm. Tierris
First Department of Surgery, Red Cross Hospital, Athens, Greece
Tropical Disease Laboratory., University of Athens

The E. granulosus scolices vitality after exposure to four scolicidal agents is evaluated in an experimental study utilising 50 mice allocated to 5 groups. Hydatidosis was induced by intraperitoneal injection of a suspension of scolices. Group A animals received scolices exposed to hypertonic solution NaCl 15%. Group B animals received scolices exposed to Povidone Iodine 10%. Group C scolices were exposed to $AgNO_3$ 0,5% while scolices injected to group D were exposed to $H_2O_2$ 12 Vol. Group E served as control. All mice were sacrified 8 months after the injection.
All control mice developed distinct hydatid cysts up to 8 mm in diameter. Five out of the six mice that survived from group A developed pronounced hydatidosis. One out of seven mice that survived from group B developed hydatidosis: All six mice that survived from group C developed hydatidosis, while from group D only one out of ten survivors developed a small hydatid cyst.
In conclusion, both $H_2O_2$ and Povidone Iodine seem to be more effective than NaCl 15% and $AgNO_3$ and thus are recommended for the sterilisation of the residual hepatic cavity during surgery of hydatid disease.

### F294

**MARMOTTA MONAX : A NATURAL MODEL OF HUMAN HEPATOCELLULAR CARCINOMA (HCC)**

D.Manganas, C.Gouillat, G.Saguier, R.Duque Campos
Département de Chirurgie, Hôtel Dieu Lyon France

Eastern american woodchuck (Marmotta monax), naturally infected with WHV, a virus similar to human hepatitis B virus, develops HCC in a high prevalence. The aim of this work is to assess Marmotta monax as a model of HCC specially to evaluate therapeutic modalities.
Forty four animals were regularly followed by ultrasound examination from the age of 18 months and for a 30 months period.
One or more liver tumors were diagnosed in 31 animals (70 %). Five of them with multifocal tumor or poor general status were not suitable for surgery. The 26 others were operated on. No tumor was found in 3 (11 %). The tumor was managed by tumorectomy in 8, alcoolisation in 7, laser photocoagulation in 1 and simple biopsy in 7.
Ten animals died postoperatively. The 4 survivors in the tumorectomy group died from tumor recurrence between 10 and 18 months after surgery. All liver tumors were HCC grossly and microscopically similar to human hepatoma. The peritumoral parenchyma demonstrated chronic hepatitis without cirrhosis.
In conclusion, Marmotta monax results in a natural model of human HCC which can be used to assess the main therapeutic modalities. The pathology and natural history of WHV-induced tumors are similar to the ones of human hepatoma, including early ultrasonic detection and recurrence after resection. However its routine use is limited by the cost and the fragility of the animal together with its long hibernation period.

### F295

RETICULOENDOTHELIAL SYSTEM (RES) FUNCTION IN ACUTE LIVER INJURY

F.B. Kasravi, W. Guo, B. Jeppsson, S. Bengmark
Dept. of Surgery, Lund Univ., Lund, Sweden

RES function was evaluated in acute D-galactosamine liver injury in rats using radiolabelled bacteria and was compared to changes in 70% liver resection model. Clearance rate (k-value) was decreased in both groups, but there was higher corrected phagocytic index in the remaining cells of the 70% resection group. Pattern of blood flow and organ distribution of radiolabelled bacteria were also different in experimental groups. This may explain the phenomenon of bacterial translocation observed in these groups.

### F296

ACUTE PHASE RESPONSE INDUCTION AND RAT LIVER REGENERATION AFTER PARTIAL HEPATECTOMY

S.Theocharis, S.Skaltsas, A.Margeli, *K.Grigoraki, C.Spiliopoulou, *C.Kittas, A.Koutselinis
Departments of Forensic Medicine and Toxicology and *Histology and Embryology, School of Medicine, University of Athens, Athens, GR 11527, Greece

Extensive alterations of genes'expression in liver occur experimentally during regeneration induced by partial hepatectomy (PH) and in situations of acute phase response due to trauma and inflammation.We evaluated the influence of an acute phase.response induction, that triggers the synthesis of exportable proteins by hepatocytes and the circulation of important cytokines, on liver regeneration after PH in rats.The subcutaneous administration of turpentine oil (TO) in rats causes a release of acute phase reactants reaching peak levels 24 hours after the injection.In partially hepatectomized rats (Group I), an increase of hepatocytes' DNA synthesis and thymidine kinase (TK) activity was observed 24 hours postoperatively, compared to sham operated ones (Group II).In hepatectomized rats with TO pretreatment, 24 hours priorly to PH,(Group III) DNA synthesis and TK activity were significantly enhanced compared to simply partially hepatectomized ones (Group I) ($p<0.001$).Enhancement was not obtained when the TO administration occured simultaneously to PH (Group IV).The mitotic index of hepatocytes and PCNA immunostaining data were also coestimated. The above data suggest that the stimulatory effect of acute phase response induction on hepatocytes' proliferation occurs, when the maximum circulation of acute phase reactants coincides with the time of PH, triggering the hepatic regenerative process.

### F297

### ULTRASOUND GUIDED SCLEROTHERAPY OF LIVER CYSTS

T. Winternitz, P. Kupcsulik, L. Flautner

1st. Surgical Department of Semmelweis Univ. of Medicine
Budapest, Hungary

The recurrence of liver cysts are often observed after surgery and ultrasound guided drainage. It's mainly true at the patients suffered from polycystic liver disease.
For the decreasing of recidivous the post-suction sclerotherapy is a well known method.
This method is used in our Department since 1989.
Six patients with polycystic liver disease where treated 43 times. The total number of treated cysts was 156 (diameter 1-15 cm).
In first step the cysts were punctured and suctioned, after this the sclerotherapy was made with 90 % ethanol. The volume of alcohol injected varied from 10 to 70 ml, depending the size of the cysts.
We observed only minor side effects such as transient pain, temperature elevation and a mild alcoholic state.
Due to the good results of previous patients we treat the solitaire liver cysts with the same method too.
We treated ten patients with 3-20 cm cysts successfully. Over the 3-21 months follow up period there were no recidiv observed.
We recommend this simple but successful method for the treatment of cystic liver disease.

### F298

### PSYCHOLOGICAL ASSESSMENT IN OUT-PATIENTS SUBMITTED TO LIVER TRANSPLANTATION

I.F.S.F. Boin, M.C. Santos, E.Y. Udo, M.R. Banin, G. Berenhauser-Leite, L.S. Leonardi
Unit Liver Transplantation, State University of Campinas, Campinas-SP, Brazil

The objetive point was analysed the general health: (life quality, social reintegration and capacity to return to work) in ten patients with advanced chronic liver disease three months before and six months after liver transplantation.
We were applied a census paper where we asked about work capacity, familiar integration, social integration, practice of sports, physical conditions before and after the liver transplantation.
In all patients the life expectation was poor.
We observed that 8 (80%) patients were working after six months.
In a (90%) patients there were complete social and familiar reintegration. In 7 (70%) patients there were the best life quality than before and 2 (20%) to get arising in life with less intensity and 1 (10%) case there was total dependency of the your family. Only one patient was student and he is studing again.
After the application these scores we concluded that liver transplantation in our country is a therapeutical procedure and the patients to get a rising in your life.

### F299

### RISK FACTORS AND OUTCOME OF PORTAL AND MESENTERIC VEIN THROMBOSIS OF THE ADULT

T. Bemey, M. Morales, P.E. Broquet, G. Mentha, A. Rohner, Ph. Morel
Digestive Surgery, Department of Surgery, University Hospital, Geneva, Switzerland

The aim of this study was to review our experience with portal and superior mesenteric vein thrombosis (PMVT), to identify risk factors that influence the outcome, and, based on these observations, to propose a scheme of management admitted in our departement over a 17-year period was undertaken, and 45 patients were identified. The most frequent etiological factors were cirrhosis (47 %), intra-abdominal malignancy (31 %) and pancreatitis (22 %), one third of patients exhibiting more than one etiological factor. There were 16 % idiopathic cases. Cirrhotic and non-cirrhotic patients had different features of disease. Porto-systemic derivation was present in 95 % of cirrhotics and 63 % of non-cirrhotics ($p < 0.01$), whereas cavernomatous transformation was observed in 43 % of cirrhotic and 63 % of non-cirrhotics ($p = 0.1$). Over the course of the disease, rupture of gastric or esophageal varices was more frequent ($p < 0.005$) in cirrhotics (75 %) than non-cirrhotics 17 % ($p = 0.1$). Moreover, of the 14 cirrhotics who had variceal rupture, 10 bled at presentation, whereas of the 4 non-cirrhotics who bled, 3 did so after a delay (median 2.5 years). Endoscopic sclerotherapy was always used as the first option of treatment for variceal rupture with a success rate of 75 % but a recurrence rate of 56 %. Surgical therapy (shunt or Sugiura procedure) was necessary for 27 % of bleeding patients. Signs and symptoms at presentation were scant and poorly specific, abdominal pain being the only symptom exhibited by a majority (60 %) of patients. Overall actuarial survival rate was 43 % at 5 years, but was significantly influenced by the presence of cirrhosis (23 %, $p < 0.001$) or malignancy (0 %, $p < 0.0001$), or the occurence of hematemesis (18 %, $p < 0.005$). Indeed, gastrointestinal bleeding and terminal malignancy were responsible for 50 % of deaths, and 5-year actuarial survival rate for patients without cirrhosis and cancer reached 78 %.
This study shows that gastrointestinal bleeding is a critical symptom of PMVT, as it has a major influence on the outcome, requires urgent treatment and raises important questions on the management. We have identified a subgroup of patients with a good prognosis (no cancer, no cirrhosis, no variceal bleeding), who may benefit from an anticoagulative therapy, with the aim to prevent extension or recurrence of thrombosis, and thus occurence of delayed gastrointestinal bleeding.

### F300

### IS THE WATERSOLUBLE VITAMINS SERUN LEVEL A SENSITIVE METHOD OF LIVER DISEASE DIAGNOSIS ?

V. Vdovitchenko, A. Podorozny, E. Chodosevicz,

Lviv Medical Institute, Ukraine

A search of the sensetive methods of liver disease diagnosis is continuing and today. The purpose of this investigation was to verify on such role the vitamins level. The basal serum level of vitamin C, PP, $B_1$ and $B_2$, was studied in 27 patients (males 25, females 2, mean age 46 years) with chronic liver desease (alcohol induced liver injury 17, no alkohol 10; hepatitis 17, cirrhosis 10, persistent hepatitis 10, active hepatitis 7). Control group were 15 health donors of the same mean age stadied in winter- spring 1994 also. The average serum levels of vitamins C, PP, $B_1$ and $B_2$ were accordingly 49,4±2, 2 and 54,6±2, 4 mkM/l, 136,7±9,1 and 147,0±7,3 nM/l in health persons but 35,0±1,6 and 38,6±3,0 mkM/l, 73,0±7,8 and 88,5±10,9 nM/l in patients with the liver disease. So, in these patients the vitamins levels were lowered on 29,2-46,4% to the donors indexes. It was not any correlation between the vitamins levels and injured factors, activity of hepatitis and a severity of the disease. These data permit to consider that the level of watersoluble vitamins in serum is a very sensitive test on the liver injury. In the same time this test can not be a specific one and to be used for monitoring in clinical trials.

### F301

RETROSPECTIVE ANALYSIS OF 78 PATIENTS TREATED FOR HCC

C. Brunken, E. Neumann, W.T. Knoefel, V. Nicolas*, M. Malagó, C. Zornig, J.R. Izbicki, X. Rogiers, R. Kuhlencordt, C.E.Broelsch
Departments for General Surgery and Radiology*, University Hospital Eppendorf, Hamburg, Germany

The study was performed to evaluate the treatment of hepatocellular carcinoma at our institution. The files from 78 patients treated in the period from 1986 to 1993 for histological proven HCC were reviewed.
Only 8 tumors (9,7%) were smaller than 5 cm. Twenty-eight patients (35%) had more than 2 nodules. The median tumor size was 10 cm. Twenty patients (26%) had a non-cirrhotic liver; from the remaining patients 17 (22%) had Child A, 23 (29%) Child B and 12 (15%) patients Child C cirrhosis. Fourty-two patients (53%) underwent surgery: 12 LTX; 16 $R_0$ resections; 5 $R_1$ resections; 1 $R_2$ resection; 8 explorative laparatomies. A chemoembolistation with doxyrubicin was performed in 12 patients (15%). Twenty-four patients (30%) recieved a symptomatic therapy.

Results:

| | 1 year survival | 2 year survival | 3 year survival |
|---|---|---|---|
| LTX | 52 % | 26 % | 26% |
| $R_o$ | 61 % | 29 % | 0% |
| $R_x$ | 27 % | 8% | 0% |
| Chemoembol. | 34% | 0% | |
| Sympt. Th. | 14% | 8% | 0% |
| no chirrosis | 35% | 6% | 0% |
| Child A | 33% | 17% | 0% |
| Child B | 52% | 34%* | 0% |
| Child C | 0% | | |

The choice of treatment modality is more defined by the quality of the patients liver than by tumor size.
The best survival results are obtained with surgical resection with curative intent in patients with good liver function or LTX in patients with Child B*.
The results might even suggest that LTX provided better survival than resection independent of the patients liver quality. Larger numbers are however required to confirm the data.

### F302

HEPATITIS CHRONICA C- THERAPY WITH INTERFERON

Lj.Konstantinović, V.Kostić,D.Tasić-Dimov, M.Krstić,Z.Ranković

Clinic for Infectious Diseases,Niš,Yugoskavia

VHC infection frequently leads to chronic liver diseases.The results of treating Hepatitis chronica C with interferon(IFN) are presented in this work.7 patients were treated for chronic hepatitis C with IFN.Three patients had Hepatitis chronica persistens(CPH), while four patients had Hepatitiss chronica activa(CAH).Usual tests of functional liver examination were studied(AST,ALT,billirubin, electrophoresis of proteins,imunoglobulins, liver EHO,scintigrahpy of liver as well as liver biopsy).All of them were HBsAg negative and anti HCV positive.Other serological investigations were not performed(PCR HCV RNA). Anti HCV-Ab were determined by a II generation ELISA Test.All the patients received 3 MU of recombinant IFN alfa-2a thsee times weekly for three monaths.After the treatment of three monaths,in two patients with CPH and two patients with CAH,biochemical values were normalized.During the follow up of six monaths neither of the patienths had relapse of raised of amonotranspherases.Anti HCV-Ab were found by serological examinations.In 3 patients,there was no improvement of biochemical values after the treatment with interferon.Although there was not a great number of the patients treated,the beneficial effect of IFN on chronic HCV infection can be confirmed.

### F303

OUR REMARKS ON THE ROLE OF MICROVASCULAR CIRCULATION OF THE INTESTINAL WALL IN MULTIINJURED PATIENTS WITH LIVER AND PANCREAS TRAUMA.

Mar. Paraskevopoulou, Sp. Milingos, D. Tsambrinou, J. Omirou, D. Michaelidis, D. Panagoulis, V.Kollia
"Evangelismos" General Hospital & Saint Panteleimon General Hospital, Greece

In the present work an effort was made to study the results of regional ischaemia of the intestinal wall in multi injured patients.

After experimental study in dogs in which we produced intestinal ischaemia in experimental animals we study the intestinal ischaemia that we observed in 10 patients after operations for abdominal blunt trauma (with liver and pancreas damage). These patients submitted to an operation (laparotomy) after their wounding. Seven to 15 days after the time of traumatism they present signs of acute abdomen except the clinical findings the alterations of the biochemical and coagulations factors which can be summarised in a slight increase of Ph, P CO2 serum potassium level of Hct, lactate and pyruvate acid, while a slight decrease was noticed on serum bicarbonate sodium and platelet count. Histological tissue and the infection of findings suggest that the changes of the circulation on bowel wall are the causes of the ischaemia.

As a result we conclude that the ischaemia of the intestinal wall after blunt abdominal trauma and the inflammatory reactions are the causes with the above biochemical and coagulation changes.

### F304

LIVER TRANSPLANTATION FOLLOWING COMPLICATIONS OF LAPAROSCOPIC CHOLECYSTECTOMY (LC)

C.S. Skillern, J.M. Sackier
Department of Surgery, School of Medicine, University of California, San Diego

This study was performed to establish the incidence of the necessity for liver transplantation after LC due to bile duct or arterial injury, and to characterize common factors that make injury more likely. A questionnaire was sent to every liver transplant center (n = 105) in the U.S.A. which was designed to evaluate the above question. To ascertain whether there was any difference between LC and open cholecystectomy (OC), the centers were asked to also include data from the pre-laparoscopic era. At the time of submitting this abstract, not all the data has been received. However, results thus far indicate that LC is more likely to result in a bile duct or arterial injury that rquires liver transplantation, placement on a transplant waiting list or death before transplantation could occur. One transplant was performed due to hepatic artery ligation during LC. Certain factors were associated with these injuries: 1) history of prior abdominal surgery; 2) weight greater than 85 kg; and 3) failure to perform an intraoperative cholangiogram. The importance of these results is in the potential for decreasing the morbidity and mortality from LC by recognizing the factors that predispose to bile duct or arterial injuries. Additionally, certain patients may benefit from an OC, or conversion from LC to OC. Further, if an injury should occur, repair should be performed at the appropriate time and by a surgeon experienced in biliary tract reconstruction.

### F305

HYDATIDIC JAUNDICE

M. Gyras, C. Tsolias, N. Sias, A. Papathanasiou.

Therapeutic Hospital Athens

Purpose of this paper is to discuss our view for the management of hydatidic jaundice, according to the new technological developments in medicine.
Our material: we discuss three cases of hydatidosis of the liver that were operated 3 to 5 times for complications and recurrence of the disease among 1960-1985. (Common complication was the development of secondary gall-stones into the bile duct, with nucleus material from the hydatid cyst.)
Discussion: hydatidic jaundice characterised by the triangle of Dene (Harris 1965). [Hepatic colic pain, jaundice-fever, loss of hydatid tissue in the bowel.] In this paper we dispute the older surgical beliefs. (Surgery must be performed within the first 24h - The common bile duct must always be explored - The hydatid cyst must be operated at the same time - Intraoperative cholangiograpfy - Sphincterotomy or biliodigestive by-pass - Cholecystectomy and T-tube placement)
Prospects-proposals for the treatment of hydatidic jaundice: 1. ERCP-spicterotomy 2. Antibiotics - culture of the ruptured cyst 3. Drainage of the cyst with the help of CT or US scans 4. Appropriate medications 5. Review of the situation after reasonable time.
Message: Never emergency operation.

### F306

N. Rayes, G. Blumhardt, N. Kling,
G. Schumacher, W.O. Bechstein, P. Neuhaus
Department of Surgery,
University Clinic Rudolf Virchow, Berlin, Germany

### SURGICAL THERAPY OF ECHINOCOCCUS CYSTICUS-CYSTS IN THE LIVER

**Introduction:**
Echinococcal cysts are an important differential diagnosis of liver cysts. In the literature pericystectomy and cystectomy are described as the treatment of choice. Because of their high risk of complications liver resections should only be performed if pericystectomy is technically difficult or impossible. In this study the two forms of therapy are compared retrospectively.
**Patients and methods:**
Between 1987 and 1994 fifteen patients with Echinococcus cysticus-cysts underwent surgery in our clinic. Their range of age was forty to sixty years (median 36 years), seven patients were male and eight female. Clinical symptoms were abdominal tenderness (n=7), weight loss (n=5) and fatigue (n=4); the diagnosis was established by CT-scan and serological tests. The following operations were performed: pericystectomy (n=4), cystectomy (n=1) and liver resection (n=10). The latter group consisted of eight right hemi-hepatectomies, one left hemi-hepatectomy and one atypical resection. Median hospitalisation time in both groups was twenty days. Postoperatively there were two complications in the group of the five patients after pericystectomy and cystectomy: one patient developed a bilioma that had to be drained and another patient got a recurrence of the disease. None of the ten patients after liver resection had postoperative complications. The follow-up period was six months to eight years.
**Conclusion:**
Liver resection can be a low-risk therapy for echinococcal cysts.

### F307

### SURGICAL TREATMENT OF HEPATIC HYDATIDOSIS; EVALUATION OF 78 PATIENTS

K.Stavridis, P.Skiadas, E.Koniaris, H.Kolimbiris, .T.Kakavoulis, A.E.Ziros
A' and B' Departments of General Surgery, Konstantopoulion General Regional Hospital,"Agia Olga" N.Ionia, Athens, Greece.

**We evaluated 78 patients with hepatic hydatidosis, who were operated during 1986 to 1994. The main clinical manifestations were abdominal pain [80,7%], palpable mass or enlarged liver [51,2%], pyrexia [47,7%], jaundice [10,2%] and only 11 patients [14,1%] were asymptomatic. The diagnosis was established by U/S and C/T or both. The treatment in all patients was surgical and there were no operative or postoperative deaths.
In 34 [43,5%] patients we performed total cystectomy, in 28 [35,8%] wide capsectomy and drainage of the residual cavity, in 13 [16,6%] we performed limited cystectomy, evacuation of the cyst and omentoplasty and in 3 [3,8%] marsupialization of the cyst. In 13 [16,6%] patients with biliary communication of the residual cavity the operation was completed with drainage of the common bile duct with a T tube or choledocho-duodenal anastomosis. The mean hospitalization time in the group of the total and wide cystectomy was 12 days, whilst in the other group was 22 days. The average follow up time in both groups was 26 months [from 6 to 40 months]. There were only 2 recurrences in the cystectomy or wide capsectomy group and 7 in the other group.
Because of the low rate of recurrence and the shorter period of hospitalization, it seems that total or wide cystectomy and drainage of the residual cavity are the most efficient surgical treatment for hepatic hydatidosis.**

### F308

THE ROLE OF POSTOPERATIVE COMPUTED TOMOGRAPHY IN HYDATID CYSTS OF THE LIVER

J.Prousalidis, E. Tzardinoglou, A. Apostolidis,
K. Katsohis, H. Aletras.
Propedeutic Surgical Clinic, ARISTOTELIAN University
AHEPA Hospital Thessaloniki, Greece.

Preoperative diagnosis in hepatic echinococcosis is easy. In contrast the postoperative follow-up copes with difficulties. The aim of this study is the estimation of the value of CT scan in this problem. We present the postoperative findings in 96 patients who underwent, in the period 1983-1993, removal of hydatid cyst of the liver. Seventy were men and 26 women. In 81 cases the disease was single and in 15 multiple, in 79 in the right lobe, in 12 in the left and in 5 in both of the lobes of the liver. Pericystectomy with drainage of the residual cavity in 81 patients and with primary suture in 15 patients, was done. The postoperative CT scan between 15 days and 1 year after the operation, was performed. The findings varied with the technique employed. In 85 patients the cavity was clearly reduced and the recurrence was excluded with one tomogram. In 11, another tomogram was necessary. We conclude that with postoperative CT tomogram the succes of the operation and the restoration of liver anatomy are very well illustrated and the present and following management of the patients is easily done.

### F309

### SURGICAL MANAGEMENT OF THE HYDATID DISEASE OF THE LIVER

T. Pavlidis, I. Spathopoulos, S. M. Jankovic, D. Kouvelas
Second Department of Surgery, Agios Pavlos Hospital, Thessaloniki, Greece

The hydatid disease due to Echinococcus granulosus affects the liver predominantly and seems common in some areas, but with decreasing frequency. In this study we present our experience of the hepatic hydatid disease(HHD), which was more or less common in our country previously. Over the past 10 year period in our department 45 patients with HDD were operated on out of 5556 operations(0.8%). They consist of 19 males(42%) and 26 females(58%) with mean age of 56±8 years(range of 28 to 77). The disease was primary in all but 3 recurrences. The lesion was solitary in 28(62%) and multiple in 17(38%),located on the right lobe in 30(two thirds) including 5 cases posteriorly adjacent to the i.v.c.,on the left lobe in 12 and in 3 it was bilobar. In 11 cases(24%) an extra hepatic spread coexisted. On operation, care for prevention of dissemination was taken. In 10 cases with the disease located in the left lobe atypical lobectomy was done,while in the remaining 35 cases partial pericystectomy, meticulous evacuation and drainage with wide bore silastic tube(s) was performed. In 5 cases omentoplasty of the residual cavity was added. In 10 cases(22%), because of assumed rupture into the biliary tree, cholecystectomy, CBD exploration and T-tube insertion was considered necessary adding sphincteroplasty in two of them. There was not postoperative mortality. The morbidity was 11% including 4 biliary fistulas lasting for 40 to 60 days,and one high biliary stricture requiring operative reconstruction. In conclusion, partial pericystectomy and drainage is a simple and safe procedure and should be the first choice in the management of the HHD.For location on the left lobe lobectomy could be a safe alternative offering radicallity.

### F310

### THE MANAGEMENT OF THE HYDATID CYST OF THE LIVER (ELEVEN YEARS EXPERIENCE)

A. Manouras, S. Papadopoulos, G. Charalambous, D. Panoussopoulos, B. Paizis, N. Apostolidis, B. Golematis
A' Propaedeutic Surgical Clinic of Athens University.

During the last eleven years (1984-1994), 280 patients (142 F, 138 M, of mean age 50,8 years) with echinococcal cysts of the liver were operated in our Department.
52/280 (18,57%) patients, already had been operated once of twice for their disease, in the past. The mean period from their first operation was 6,9 years (1-26 years).
In 257/280 (91,78%) the cyst was located only in the liver. The rest 23/280 (8,21%) had disease in other organs too.
234/280 (82,57%) had a solitary hepatic cyst and the rest 46/280 (16,42%) had multiple foci. The localization in the Rt lobe of the liver observed in 198 patients (70,71%), the Lt, in 46 (16,42%), and in both lobels found in 36 (12,85%) patients.
Wide communication of the cyst and intrahepatic biliary ducts was noticed in 39 (14%) patients. The most frequent symptom was abdominal pain in 202/280 (72,14%) and palpable abdominal mass found in 135/280 (48,2%) cases.
Antiechinococcal Abs were positive in 235/280 (83,9%) whereas eosinophilia, of varying degree, found in 49 (17,5%) cases.
In the majority of the patients limited operations were performed and in only 30/280 (10,71%) pts, the total cystectomy was possible. Atypical hepatectomy was necessary in 5/280 (1,78%) patients.
Simultaneous operations performed in 73/280 (26,07%) pts including cholecystectomy in 40/280 (14,8%) with common bile duct exploration in 26/280 (9,3%). The post-op complications were the usual ones. Post-op cholorrhea (>3 days) occured in 27/280 (9,6%) pts. The hospitalization in days varied between 2-67 (mean time 15,4 days). 4/280 (1,42%) patients died peroperatively. The follow-up included 224/280 (8%) pts, of whom 11 (4,9%) had recurrence of the hydatid disease of the liver.

### F311

### POSTOPERATIVE COMPLICATIONS AFTER SURGERY FOR LIVER HYDATIDOSIS

### F312

### RESULTS OF SURGICAL TREATMENT OF HEPATIC HYDATIDOSIS:CURRENT THERAPEUTIC MODIFICATIONS

I.Jekić,M.Jekić.
Institut of the Digestive Surgery,Medical Faculty Belgrade,and Surgical Service,Clinical Hospital Center Zemun-Belgrade,Yugoslavia

Hepatic hydatidosis is an endemic disease that affects vast segments of the populations of various countries in the Mediterranean region South America,the Pacific ,and temperate zone nations that possess large numbers of sheep. One hundred and ten patients bearing 361 hydatid cysts were treated at major hospitals. our patients were divided into 2 groups:group A corresponded to the period 1974-1984 and group B,corresponded to the period 1984-1994. Since no effective parasiticide agent is available,hepatic hydatidosismust be treated surgically.Todey's better knowledge and advancements in liver surgery have made it possible to extirpate the cyst completely with little risk and improved results;hepatic resection should only be considered in exceptional cases;aspiration,drainage procedures,or partial resections of the cyst yield inferior results.We have had no relapse of the hydatid disease in the liver or in any other abdominal site.

### F313

Diagnostics And Treatment Of Hydatid Disease Of Liver.

F.Todua,N.Grigolia,I.Beridze,T.Ioseliani, N.Samadashvili.

The Research Institute of Radiology and Interventional Diagnostics

70 patients (25 men,45 women)with hydatid disease between the ages of 15-70 have been examined.

### F314

HYDATID CYSTS OF THE LIVER - INCIDENTALOMA IN NONENDEMIC COUNTRIES

E. M. Gadžijev, D. Stanisavljević

Department of Gastroenterologic Surgery, University Medical Center, Ljubljana, Slovenia

The widespread use of ultrasonography caused that more and more hepatic cystis lesions are discovered nowadays.
Hydatid disease is still endemic in some sheepbreeding countries specially in Mediterranean districts, but people travelling and migration are the cause of finding patients with hydatidosis also in countries where the disease has never been present.
We present a series of patients treated surgically at our departement for hydatid cysts of the liver in the period from 1988 to I994. In 52 patients out of 84 treated for liver hydatidosis in that period, the disease was found incidentally at UZ investigation.44 patients (85%) either spent their youth in the region where hydatidosis had been endemic but eradicated in the sheep population 40 years ago or they were still living there. 8 patients had been living for only a shorter time in endemic regions as tourists (3 pts) or had been at millitary service there (5 pts).
Typical findings in those patients were solitary cysts (48 pts - 92%) often with calcinations (20 pts - 38%) and degenerative changes of the cyst contents in 25 cases (48 %). Serologic tests were performed in 38 patients (73 %) and were positive in 20 cases (53% out of 38 cases).
CT was used after US investigation in 12 patients.
Operative procedures performed were mostly total pericystectomy without opening the cyst with low morbidity rate and no mortality.
We conclude that hydatide disease of the liver is often incidental finding in nonendemic countries and that it can be treated radically with total pericystectomy in HPB units.

